# Endothelial Protein C Receptor Function in Murine and Human Breast Cancer Development

**DOI:** 10.1371/journal.pone.0061071

**Published:** 2013-04-09

**Authors:** Florence Schaffner, Naho Yokota, Tatiana Carneiro-Lobo, Maki Kitano, Michael Schaffer, G. Mark Anderson, Barbara M. Mueller, Charles T. Esmon, Wolfram Ruf

**Affiliations:** 1 Department of Immunology and Microbial Science, The Scripps Research Institute, La Jolla, California, United States of America; 2 Biologics Research, Janssen Research and Development, Radnor, Pennsylvania, United States of America; 3 Cancer Research, Torrey Pines Institute for Molecular Studies, San Diego, California, United States of America; 4 Coagulation Biology Laboratory, Oklahoma Medical Research Foundation and Howard Hughes Medical Institute, Oklahoma City, Oklahoma, United States of America; University Medical Center Utrecht, Netherlands

## Abstract

Several markers identify cancer stem cell-like populations, but little is known about the functional roles of stem cell surface receptors in tumor progression. Here, we show that the endothelial protein C receptor (EPCR), a stem cell marker in hematopoietic, neuronal and epithelial cells, is crucial for breast cancer growth in the orthotopic microenvironment of the mammary gland. Mice with a hypomorphic allele of EPCR show reduced tumor growth in the PyMT-model of spontaneous breast cancer development and deletion of EPCR in established PyMT tumor cells significantly attenuates transplanted tumor take and growth. We find expansion of EPCR^+^ cancer stem cell-like populations in aggressive, mammary fat pad-enhanced human triple negative breast cancer cells. In this model, EPCR-expressing cells have markedly increased mammosphere- and tumor-cell initiating activity compared to another stable progenitor-like subpopulation present at comparable frequency. We show that receptor blocking antibodies to EPCR specifically attenuate *in vivo* tumor growth initiated by either EPCR^+^ cells or the heterogenous mixture of EPCR^+^ and EPCR^-^ cells. Furthermore, we have identified tumor associated macrophages as a major source for recognized ligands of EPCR, suggesting a novel mechanism by which cancer stem cell-like populations are regulated by innate immune cells in the tumor microenvironment.

## Introduction

The coagulation cascade is an evolutionary conserved pathway in vertebrates that maintains vascular integrity, protects from infection, and supports regenerative processes after injury. Coagulation is initiated through the intrinsic pathway by polyanionic intrinsic or extrinsic danger signals [1], [2] or through the extrinsic pathway by the cytokine receptor family member tissue factor (TF) that is expressed by vessel wall and innate immune cells [3]. TF binds the serine protease coagulation factor (F) VIIa and the TF-FVIIa complex activates FX to FXa, leading to thrombin generation, fibrin formation and platelet activation that are crucial for hemostatic clot formation and prevention of bleeding. The TF-VIIa complex also regulates angiogenesis through coagulation-independent cell signaling [4] and thereby supports coagulation-dependent mechanisms in wound repair [5].

Activation of the coagulation system is also a characteristic of advanced cancer and thrombotic complications are major contributors to morbidity and mortality in cancer patients [6]. Oncogenic transformations induce TF expression by a variety of cancer types and TF promotes the prothrombotic state of cancer patients and thrombin-dependent activation of the host hemostatic system in metastasis [5]. In addition, TF-FVIIa regulates cancer cell migration and initiates proangiogenic cell signaling by proteolytic cleavage and activation of the G protein-coupled protease activated receptor (PAR) 2, supporting tumor development and growth in orthotopic tumor microenvironments [7]–[11]. Other procoagulant proteases, i.e. thrombin and FXa, as well as matrix metalloproteases have pleiotropic pro-invasive and growth promoting effects on tumor cells and these effects are frequently dependent on activation of the thrombin receptor PAR1 [12], [13].

The procoagulant effects of the TF pathway are counterbalanced by the protein C (PC) anticoagulant pathway to avoid intravascular thrombosis [14]. PC is activated when thrombin binds to endothelial cell-expressed thrombomodulin. In this pathway, a CD1d–like immune receptor, the endothelial protein C receptor (EPCR), binds the γ-carboxyl glutamic acid-rich (Gla) domain of PC and thereby markedly improves PC activation at the endothelial interface. EPCR also serves as the co-receptor for activated PC (aPC) in vascular protective signaling mediated by activation of PAR1 [15]–[17]. Endothelial overexpression of EPCR attenuates metastasis, presumably by dampening thrombin generation that supports metastatic tumor cell survival in vascular niches [18]. EPCR-dependent PAR1 activation by aPC also stimulates cell migration of breast cancer cells or prevents apoptosis of lung cancer cells to enhance metastasis [19], [20]. In addition to aPC, EPCR binds the amino-terminal Gla-domains of FVIIa and FXa and contributes to signaling by these proteases [21], [22], but contributions of these receptor interactions to cancer progression are unknown.

In addition, EPCR is found on hematopoietic, neuronal and epithelial progenitor populations [23]–[26], but functional roles of EPCR in stem cell biology are incompletely understood. EPCR is expressed by highly aggressive basal-like breast cancer subtypes [27]. Clinical cancers contain stem cell-like subpopulations that can be selected by several markers, including a CD44^high^/CD24^−^ surface phenotype [28], expression of aldehyde dehydrogenase (ALDH1) [29], as well as EPCR [30]. However, normal stem cell niches and solid tumors contain multiple stem cell populations that are not organized in a strict hierarchy, but rather bi-directionally interconvert between themselves in the context of cues from their environment [31] and also with non-stem cell compartments, in particular in breast cancer [32].

Large scale genomic data, while supporting a link between the expression of stem cell markers and poor prognosis, have also illustrated the challenges to identify clinically relevant cancer stem cell properties in the context of tumor heterogeneity and plasticity [33]. New insights into the biology of cancer stem cell-like populations can be expected from the identification of pathways that are relevant for the maintenance or expansion of these subpopulations in tumor microenvironments. Initial evidence in glioblastoma indicates that stem cell markers, such as the integrin α6, are indeed critical for tumor progression [34]. While EPCR-selected populations of breast cancer cells grow as non-adherent spheroids and have high tumorigenicity when injected at low cell numbers [35], functional roles of this marker remained elusive. Here, we provide combined genetic and pharmacological evidence that EPCR functions as a crucial regulator and not solely as a surface tag of tumor-initiating, cancer stem cell-like populations *in vivo*.

## Materials and Methods

### Ethics Statement

All experiments were performed under protocols approved by the Scripps Institutional Animal Care and Use Committee in accordance with United States Public Health Policy regarding the care and use of laboratory animals. All tumor growth experiments were monitored at least twice weekly by measurements of tumor sizes. For transplanted tumor growth experiments, all groups were euthanized by overdose inhalation anesthesia when one of the animals had reached the maximally allowed tumor size of 1.5 cm. For cohorts of mice with spontaneous, multifocal breast cancer development, individual mice were euthanized when one of the tumors reached the allowed tumor size of 1.5 cm.

### Tumor models

CB17/SCID and C57BL/6J mice were from Jackson Laboratory or the Scripps rodent breeding colony. PyMT-C57BL/6 mice [36], [37] were crossed with EPCR^Low/Low^ mice [38]. The mice with mutated EPCR alleles were generated by targeting 129-derived ES cells and extensively back-crossed with C57BL/6. Because of the close proximity of the EPCR and the agouti locus, these mice retained a brown fur color. In order to control for this difference from wild-type C57BL/6 mice, we compared EPCR^Low/Low^ mice with littermate-derived EPCR^Low/WT^ mice. Heterozygous PyMT-EPCR^Low/WT^ displayed tumor progression and sizes indistinguishable from PyMT-C57BL/6 mice housed and monitored at the same time in our animal facility (Fig. S1A). Cohorts of PyMT-EPCR^Low/Low^ mice and PyMT-EPCR^Low/WT^ controls were followed for spontaneous tumor progression, as described [7], [37]. Tumor architecture was analyzed by Hematoxylin and Eosin staining (H&E). Tumor vessel density and size as well as macrophage counts were determined by IMARIS 64 (BitPlane Inc) by quantifying at least 3 fields per tumor. As in our previous experiments [7], [8], cells were injected into the 2^nd^ thoracic mammary gland for transplanted tumor growth studies. To generate EPCR-deficient PyMT-cells, EPCR^flox/flox^ mice extensively backcrossed with C57BL/6 mice [39] were crossed with PyMT-C57BL/6 mice. Tumor cells were isolated by outgrowth from minced tumors in L15 with 10% FCS and insulin (1 mg/ml). PyMT-EPCR^flox/flox^ cells were transduced twice on consecutive days with 1000 ppc Ad5 cre recombinase or control virus. EPCR-deletion was confirmed by Western blotting [21] and cells were injected into the mammary fat pad for tumor growth monitoring.

For xenograft tumor growth experiments, 1×10^6^ unsorted human MDA-MB-231 mfp cells [40] were harvested with trypsin and injected in serum-free medium into the 2^nd^ thoracic mammary gland of CB17/SCID mice [8]. For tumor-take experiments, MDA-MB-231 mfp cells were stained with αTF (10H10-Alexa 647) and αEPCR (CD201-PE RCR252, BD Pharmingen) for FACS to isolate EPCR^+^ and EPCR^−^ subpopulations. For comparison of tumor take and growth of EPCR^+^ and EPCR^−^ subpopulations, isolated cells in 50 µl growth factor-reduced matrigel cells were placed bilaterally in the same animals. For FACS analysis, tumors were digested in DMEM with 2 mg/ml collagenase A (Roche), 10 µl DNase I (New England Biolab) with agitation at 37°C for 40 minutes. After FCS quenching and passage through a 70 µm cell strainer, tumor cells were isolated with human integrin β1 specific antibody (AIIB2, University of Iowa Developmental Studies Hybridoma Bank) and viable cells identified by AAD (Life Technologies) were analyzed for expression of TF and EPCR by FACS.

### Microarray analysis

MDA-MB-231 mfp subpopulations were FACS isolated, immediately preserved in RLT buffer (Qiagen) with 10% β-mercapto-ethanol, and mRNA extracted for profiling on Affymetrix HG-U219 chips. Data was normalized with the Robust Multichip Average method in the statistical software package, R (version 2.14.2). Microarray probes were summarized with a custom chip description file based on Entrez Gene identifiers (version 15.0.0) obtained from: http://brainarray.mbni.med.umich.edu. Statistical analysis of differential gene expression was performed with the Limma Bioconductor package. Because the experimental design did not include any technical replicates, a statistical evaluation required selection of two adjacent passages to serve as a replicate pair. Samples from passages 22 and 23 were selected for this purpose as their expression values were most similar based on the principle component analysis, hierarchical clustering, and pairwise expression plots. To identify genes differentially expressed between EPCR^+^ and EPCR^−^ cells, the remaining passages were treated as discrete points in a Limma linear model of the data. Resulting FDR-corrected F-test p-values of 0.01 and fold changes of 2-fold (log2 ratio = 1) were used to select genes of interest. The moderated F-statistic tests if a given gene is differentially expressed between populations in any of the passages. The selected transcripts were further subjected to hierarchical clustering and subgroups were analyzed for pathway enrichment with Ingenuity Pathway Analysis (Ingenuity® Systems, www.ingenuity.com).

### In vitro assays

Mammospheres were grown on low attachment tissue culture plates in DMEM/F12 supplemented with B27 (Invitrogen), 20 ng/ml FGF and 20 ng/ml EGF. Mammospheres were quantified by cell counts. To measure proliferation of adherent cells, cells were seeded at defined densities on untreated dishes or culture plates coated with 5 µg/ml of the integrin α4 specific ligand fibronectin fragment CS-1 (kindly provided by Dr. M. H. Ginsberg), or 1∶2-diluted serum-free supernatant from 805 G cells that secrete extracellular matrix enriched in laminin 5. Antibodies were added at a concentration of 100 µg/ml and replenished at the same dose for incubations exceeding 48 hours, as indicated for the specific experiments. Viable cells were quantified 24–72 hours after seeding by MTT assay to assess proliferation and survival. For FACS staining cells were harvested and stained with αTF (10H10 alexa 647), αEPCR (CD201-PE RCR252, BD Pharmingen), αCD49d (9F10 PE-Cy7, BioLegend), αCD104-biotin (439–98, eBioscience) and streptavidin-Alexa 488 (Molecular Probes) or control IgG1 for 30 minutes on ice. Cells were then washed and analyzed on Aria II cytometer (BD Pharmingen). EPCR positive cells were gated against a non-specific fluorescent channel.

For confocal microscopy, cells on glass coverslips were fixed with 1% formaldehyde and stained with antibodies to TF (10H10-FITC) and EPCR (CD201-PE, Pharmingen). After counterstaining of nuclei with DAPI in PBS, 0.1% Triton ×100, cells were mounted in fluorescent medium (Dako) and analyzed with a Zeiss 710 confocal microscope (objective 40× PlanNeoFluor-1.3na) and image collection with Zen 2009 software.

### Gene expression analysis in the tumor microenvironment

Tumors from PyMT mice were digested as above and macrophages were selected in PBS, 0.5% BSA, 2 mM EDTA, pH 7.2 with either αCD11b or αCD11c paramagnetic beads at 4°C for 15 minutes and separated on LS columns (Miltenyi). Flow-through and eluted fractions were extracted with TRIzol (Life Technologies), further purified by RNeasy Mini Kit columns (QIAGEN), followed by cDNA synthesis with SuperScript III (Life Technologies). Transcript levels were quantified by Real Time PCR using SyBr Green Master Mix (Applied Biosystems) on an Applied Biosystems 7300 System and normalization against β actin with primer pairs shown in Table S3.

### Statistical analysis

Data are presented as mean±SD, unless otherwise stated. We used Prism 5 for unpaired T-test or ANOVA with Bonferroni posttest. Survival curves were analyzed by log rank test. Tumor size differences in PyMT cohorts were analyzed by Mann-Whitney, because the groups were not normally distributed. Two-tailed two-way ANOVA and logistic regression models were used to compare tumor volumes, weights and take between sorted EPCR^+^ and EPCR^−^ cells using R version 2.15.0 (http://www.R-project.org/) with p = 0.05.

## Results

### EPCR-expression defines a distinct subpopulation in mammary fat pad-enhanced MDA-MB-231 mfp cells

**Figure 1:**
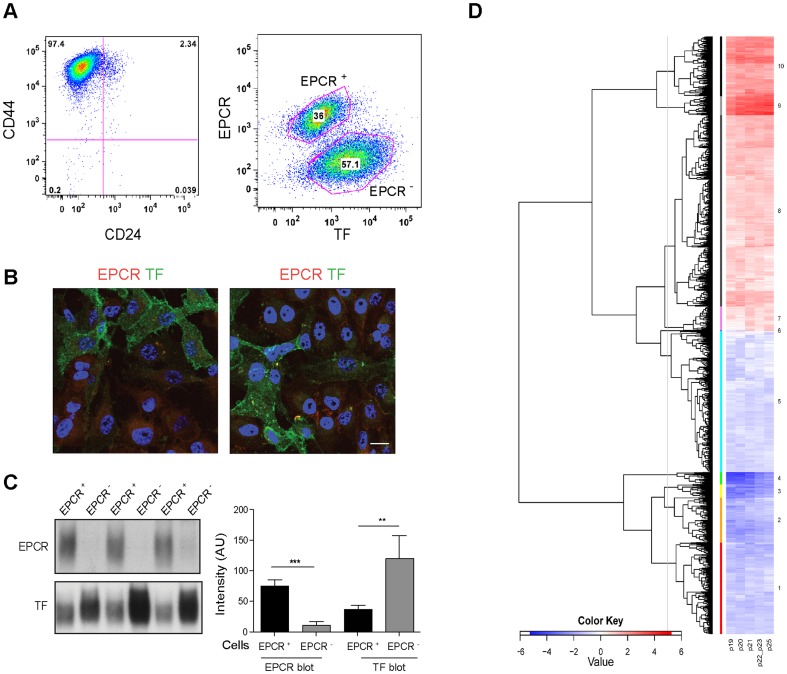
Analysis of MDA-MB-231 mfp cells sorted based on EPCR expression. **A** Cells were double stained for CD44 and CD24, or EPCR and TF using directly labeled antibodies and analyzed by FACS. **B** Non-permeabilized cells were stained for EPCR and TF, confirming the presence of EPCR^+^/TF^low^ and EPCR^−^/TF^high^ subpopulations in adherent MDA-MB-231 mfp cells; two examples of stained unsorted populations are shown (scale bar 15 µm). **C** Western blotting of sorted MDA-MB-231 mfp cells confirmed the EPCR^+^/TF^low^ and EPCR^−^/TF^high^ phenotypes, respectively, and excluded intracellular pools of EPCR in EPCR^−^ cells. A representative blot and quantification of EPCR and TF levels in cells from 4 independent sorts are shown (*** p<0.001, ** p<0.005, T-test; mean±SD). **D** EPCR^+^ and EPCR^−^ populations were isolated in 6 consecutive FACS sorts and profiled for mRNA expression. The sorts of passage 22 and 23 cells were treated as replicates in the data analysis and the clustering for the mean value for these samples and 4 additional earlier and later sorts is shown. *Hierarchical Clustering Subbranches*: The dendrogram was cut at a defined height to yield 10 gene clusters. Of these, 6 had transcripts upregulated in EPCR^+^ cells (Branch 1–6) and 4 had transcripts upregulated in EPCR^−^ cells (Branch 7–10). Gene counts were: Branch 1, n = 405; Branch 2, n = 198; Branch 3, n = 59; Branch 4, n = 54; Branch 5, n = 624; Branch 6, n = 2; Branch 7, n = 107; Branch 8, n = 844; Branch 9, n = 85; Branch 10, n = 264 (see Table S1 for gene lists). The genes for each cluster were used as input into Ingenuity IPA to identify pathway annotations or representation of gene signatures (see Table S2).

Passage through the mammary fat pad can be employed to select more aggressive tumor cell populations for tumor transplantation studies. A mammary fat pad-enhanced derivative of the triple negative breast cancer cell line MDA-MB-231 (MDA-MB-231 mfp) with markedly improved tumor growth properties was previously characterized [40] and used to study contributions of TF-FVIIa-PAR2 coagulation protease signaling pathways to tumor progression [8]. Our more recent data showed that the stem cell receptor EPCR is required for signaling of another TF protease complex, i.e. TF-FVIIa-FXa in which FXa cleaves PAR2 or PAR1 [21], [41]. In order to begin to evaluate potential contributions of EPCR to TF signaling in cancer progression, we determined the co-expression of TF and EPCR by FACS. Tumor initiating populations are enriched in the CD44^high^/CD24^low^ population of breast cancer isolates [28] and MDA-MB-231 mfp cells were predominantly of this phenotype (Fig. 1A, left panel). Surprisingly, double staining for EPCR and TF revealed two distinct subpopulations, EPCR^+^ cells and cells with high TF expression that were negative for EPCR (Fig. 1A, right panel). The differential receptor expression was also demonstrated on adherent cells, excluding artificial receptor release during cell detachment (Fig. 1B).

These subpopulations could be reproducibly isolated by FACS employing double staining and the depicted gates, yielding EPCR^+^/TF^low^ and EPCR^−^/TF^high^ cells. Western blotting of sorted cells confirmed the differential expression of these two receptors, excluding intracellular pools that escaped detection by surface staining (Fig. 1C). In order to determine whether expression of TF or EPCR was associated with a stable phenotype of subpopulations or merely a fluctuation of receptor expression of an otherwise genetically similar total population, we molecularly characterized by microarray mRNA profiling sorted EPCR^+^ and EPCR^−^ subpopulations from 6 consecutive passages. EPCR^+^ and EPCR^−^ subpopulations were remarkably stable in the levels of 2642 differentially expressed transcripts (Fig. 1D, Table S1), consistent with recent large scale genomic analysis demonstrating a phenotypic equilibrium of metastable subpopulations in cancer cell lines in culture [42].

Ingenuity pathway analysis of individual branches of the depicted hierarchical clustering showed that genes upregulated in EPCR^−^/TF^high^ cells (red in Fig. 1D) were related to pathways of TF and cancer progression, pro-angiogenic cytokine induction, integrins and cytoskeletal function (Table S2A). The EPCR^−^ cells not only expressed higher levels of TF mRNA, but also of TF's signaling receptor PAR2. These results were in accord with the previously demonstrated TF-PAR2 mediated IL-8 proangiogenic signaling and reciprocal regulation of TF and integrins [8], [11], [43].

Considering the previously demonstrated expression of EPCR by stem cell populations, we focused our attention on the differential abundance of stem cell markers between these subpopulations. The gene clusters upregulated in EPCR^+^ cells (blue in Fig. 1D) documented expression of markers associated with an aggressive or stem cell-like phenotype, including ALDH1B1 and ALDH1A3 [29], the hematopoietic stem cell marker integrin α4 [44], and the pan stem cell maker integrin α6 [45]. However, EPCR^+^ cells did not clearly match the signatures extracted for embryonic stem cells [46] and for EMT [47], or the core stem cell signature of triple negative, basal-type breast cancer [46] (Table S2B, C). Indeed, transcription factors of the core signature were found to be significantly higher in either EPCR^+^ (TEAD4, MYBL2) or EPCR^−^ (HMGA1, HMGB1, KLF5, NFE2L3) cells, indicating that EPCR^+^ and EPCR^−^ cells are coexisting and relatively stable subpopulations expressing specific subsets of stem cell and EMT markers *in vitro*.

### EPCR deficiency attenuates spontaneous breast cancer growth in the PyMT model

**Figure 2:**
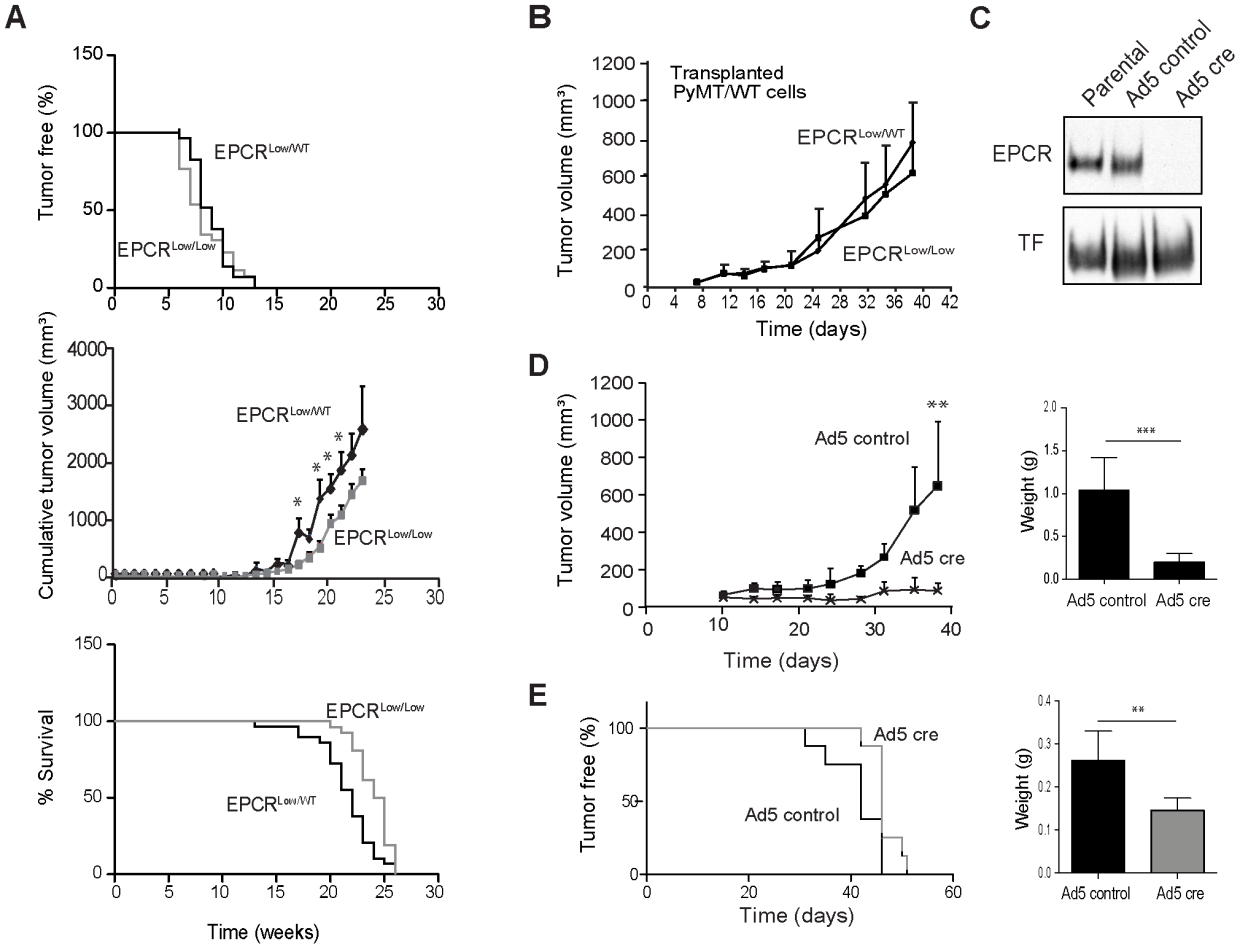
EPCR supports tumor growth in a murine model of spontaneous and carcinoma breast cancer model. **A** Cohorts of PyMT mice expressing very low levels of EPCR (EPCR^Low/Low^, n = 26) and control mice (EPCR^low/WT^, n = 29) were followed weekly for appearance of palpable tumors (upper panel) and tumor growth. Tumor appearance was not different between cohorts, but cumulative tumor volumes were reduced in EPCR^Low/Low^ versus EPCR^low/WT^ mice (middle panel, *p<0.05, Mann-Whitney, mean±SEM). EPCR^low/WT^ heterozygous control mice are eliminated from the cohort due to large tumor sizes earlier, resulting in significantly increased survival for EPCR^Low/Low^ mice (lower panel, ***p<0.001 Log Rank Test). Note that EPCR^low/WT^ mice had similar tumor sizes as C57BL/6 mice followed at the same time in the same facility, indicating that there was no gene dose effect in heterozygous mice. **B** PyMT-WT cells (2×10^6^ cells/mouse) were injected into the mammary gland of EPCR^low/WT^ or EPCR^Low/Low^ mice and a typical experimental outcome for tumor growth is shown (no difference between groups, n = 8 mice/group; mean±SD; confirmed in an independent experiment). **C** Western blotting for EPCR and TF of cell lysates from PyMT-EPCR^flox/flox^ cells treated twice with 1000 particles/cell (ppc) of Ad5 control or Ad5 cre recombinase **D**. Cells depicted in **C** (4×10^6^ cells/mouse) were implanted into the mammary fat pad of heterozygous EPCR^low/WT^ female mice (** p<0.005, T-test, n = 7 mice/group; mean±SD). Final tumor weights (*** p<0.001, T-test; mean±SD). **E** An independently established PyMT-EPCR^flox/flox^ line was selected by passage through the mammary fat pad (mfp), treated with Ad5 control or Ad5 Cre virus, and injected at a dose of 1×10^5^ cells/mouse into the mfp of C57BL/6 female mice for tumor growth monitoring (delayed tumor appearance * p<0.05, Log-rank Test, and reduced tumor weights, **p<0.005, T-test, n = 8; mean±SD).

The segregation of EPCR and TF-PAR2 expression on subpopulation of human breast cancer cells raised the question whether EPCR made a contribution to tumor growth that was distinct and independent of the established pro-angiogenic effect of TF signaling in breast cancer development [7], [8], [37]. We first employed the oncogene-driven polyoma middle T (PyMT) model that mimics important aspects of human breast cancer development, which is dependent on both tumor cell-intrinsic signaling pathways as well as tumor-host cell interactions [48], and has been suitable to define the role of TF-PAR2 signaling in tumor progression [7], [10], [37]. We crossed EPCR hypomorphic (EPCR^Low/Low^) mice [38] with PyMT mice to study spontaneous tumor development in the presence of only very low levels of EPCR. Cohorts of PyMT-EPCR^Low/Low^ or heterozygous PyMT-EPCR^Low/WT^ control mice developed palpable tumors indistinguishably at an average age of 8 or 9 weeks, respectively (Fig. 2A, top panel). The time to tumor appearance, a reflection of tumor progression from the adenoma to carcinoma stage [48], was similar to previously characterized cohorts of PyMT-C57BL/6 mice [7], [37]. However, PyMT-EPCR^Low/Low^ mice had significantly reduced overall tumor burden compared to controls (Fig. 2A, middle panel) and consequently survived longer (24.5 versus 22 weeks, respectively) until institutional tumor size limits required euthanasia (Fig. 2A, bottom panel). Overexpression of EPCR in endothelial cells reduces lung metastasis [18], indicating a role for EPCR in tumor cell survival in vascular niches. However, we found no differences in spontaneous lung metastasis between PyMT-EPCR^Low/Low^ and PyMT-EPCR^Low/WT^ mice (Fig. S1B).

The reduced tumor growth and increased overall survival could have been caused by functions of EPCR expressed by tumor cells or cells of the tumor microenvironment, e.g. macrophages or endothelial cells [14]. Analysis of hematoxylin and eosin staining of EPCR^Low/Low^ and EPCR^Low/WT^ tumors revealed no differences in the overall organization of the tumor mass between genotypes (Fig. S1C). The number of CD34^+^ tumor vessels did not differ between EPCR^Low/Low^ and EPCR^Low/WT^ tumors, although quantification of CD34^+^ areas indicated a somewhat increased vessel size in EPCR^Low/Low^ tumors (Fig. S1D, E). In addition, the density of F4/80^+^ macrophages in late stage tumors was not different between groups (Fig. S1F). In order to directly evaluate potential contributions of EPCR in the host compartment to tumor growth, we transplanted PyMT tumor cells derived from WT mice into either EPCR^Low/Low^ or control heterozygous EPCR^Low/WT^ mice. In the depicted (Fig. 2B) and an independent experiment, PyMT WT tumors grew at the same rate in both hosts, indicating that reduced EPCR expression by the tumor cells was responsible for the observed attenuated tumor growth in the model of spontaneous tumor development.

### EPCR expression by PyMT breast cancer cells supports tumor growth

In order to directly address the role of tumor cell-expressed EPCR, we developed a novel genetic model that enabled the generation of matched pairs of EPCR-expressing and deficient cell populations. We isolated tumor cells from PyMT-EPCR^flox/flox^ mice and treated the established lines with adenovirus (Ad5) control or Ad5 encoding cre recombinase. Transduction of cre recombinase efficiently deleted EPCR without reducing expression of other surface receptors, including TF (Fig. 2C). Deletion of EPCR did not influence proliferation under standard tissue culture conditions or in mammospheres (Figs. S2A, B). With two independent tumor isolates, deletion of EPCR significantly reduced orthotopic tumor growth in the mammary fat pad (Fig. 2D). We confirmed by Western blotting that EPCR remained deleted in the tumors *in vivo* (Fig. S2C), supporting the conclusion that attenuated tumor growth was caused by reduced EPCR expression. H&E stained tumors showed no differences in tumor architecture (Fig. S2D)

In order to assure optimal tumor growth properties and minimize selection artifacts of the initial tumor cell isolation, we passaged one line through the mammary fat pad of C57BL/6J mice followed by re-isolation. EPCR was again deleted using cre recombinase-expressing adenovirus versus control. We implanted into the mammary fat pad a 10-fold lower cell dose than in the previous experiment. EPCR deletion significantly delayed the appearance of tumors (median 46 days for Ad5 cre-treated versus 42 days for Ad5 controls) and reduced final tumor weights (Fig. 2E). The delayed appearance of EPCR^−^ transplanted tumors provided initial evidence that EPCR supported survival or expansion of tumor-initiating populations of fully transformed cancer cells in the orthotopic tumor microenvironment.

### EPCR^+^ cells of the MDA-MB-231 mfp line have stem cell like properties

**Figure 3:**
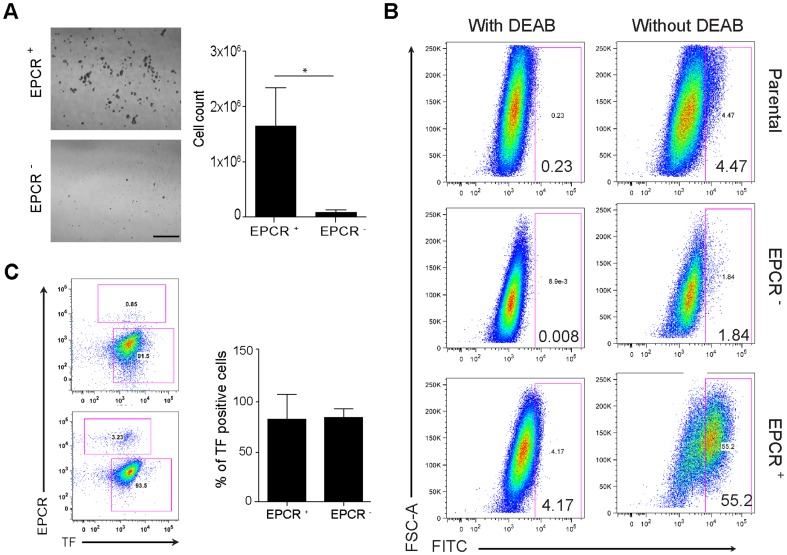
EPCR^+^ MDA-MB-231 mfp cells have stem cell-like properties. **A** EPCR^+^ and EPCR^−^ subpopulations isolated based on the gates in **Fig. 1A** were seeded at 10^4^ cells/well and grown in mammosphere medium for 10 days. Growth was quantified by cell counting **B** MDA-MB-231 mfp cells were sorted based on EPCR. Sorted EPCR^+^ and EPCR^−^ cells, as well as parental cells were cultured for 3 days, harvested and stained with the ALDH kit with DEAB addition as a specificity control, and analyzed by FACS. EPCR^+^ cells had higher ALDH activity than EPCR^−^ or parental cells. At the time of analysis, the parental populations had 8% EPCR^+^ cells. The experiment was repeated with the same outcome. **C** Tumor cells were isolated from freshly harvested tumors and immediately analyzed by FACS for TF and EPCR. A representative FACS profile and quantification for 12 EPCR^+^ cell-derived tumors and 4 EPCR^−^ cell-derived tumors are shown (mean±SD).

EPCR was expressed by a distinct subpopulation in MDA-MB-231 mfp cells (Fig. 1). We first asked whether EPCR marked the mammosphere-forming, stem cell-like subpopulations of the MDA-MB-231 mfp line, as previously shown for other breast cancer lines [30], [35]. Within 10 days, EPCR^+^ cells formed abundant mammospheres, yielding >20-fold the number of cells in comparison to cultures initiated with the same number of FACS-isolated EPCR^−^ cells (Fig. 3A). MDA-MB-231 mfp cells also contained a larger subpopulation of EPCR^+^ cells as compared to the parental line (3–4% for MDA-MB-231 vs >25% for MDA-MB-231 mfp), and produced more mammospheres, when seeded at identical cell densities, suggesting that expansion of the EPCR^+^ cells correlated with the more aggressive phenotype of MDA-MB-231 mfp cells. EPCR^+^ cells also displayed ALDH1 activity (Fig. 3B), considered another marker for cancer stem cell-like populations and poor prognosis [29].

Next we tested the ability of EPCR^+^ cells to form tumors *in vivo*. Viability of sorted EPCR^+^ and EPCR^−^ was similar and expansion of sorted single cells *in vitro* yielded comparable numbers of colonies for both populations (EPCR^+^ 33/500 sorted cells, EPCR^−^ 27/500 sorted cells). Analysis of randomly selected outgrow colonies showed that 11/11 EPCR^+^ sorted cells expressed EPCR. Conversely, 2/10 colonies derived from EPCR^−^ sorted cells expressed EPCR, indicating inefficient EPCR staining of a minor subpopulation during the sorting procedure or, potentially, conversion to the EPCR^+^ population under the tissue culture conditions. Freshly FACS-sorted cells were injected embedded in matrigel into the mammary fat pad of CB17/SCID mice. Injected EPCR^+^ cells formed significantly more tumors at lower numbers of injected tumor cells and average tumor sizes were larger in comparison to tumors developing from injected EPCR^−^ cells in 2 independent experiments (Table 1).

**Table 1:** Tumor-initiating capacity of EPCR^+^ and EPCR^−^ cells in the mfp.

		Injection of EPCR^+^ cells			Injection of EPCR^−^ cells		
	Cell number	Tumor take	Volume (mm^3^)	Weight (g)	Tumor take	Volume (mm^3^)	Weight (g)
**Experiment 1**	50 000	4/4	823±205	1±0.17	2/4	423±62	0.4±0.2
	3000	4/4	756±474	0.9±0.4	0/4		
	1000	3/4	352±203	0.8±0.4	0/4		
**Experiment 2**	10 000	4/4	582±320	0.8±0.4	0/4		
	5 000	4/4	1073±441	1.4±0.5	¼	256	0.3
	3000	3/4	930±80	1.2±0.3	¼	429	0.7
	1000	3/4	757±223	1±0.3	3/4	199±56	0.3±0.1

EPCR-expression was associated with increased tumor take (p<0.001), increased tumor volume (p<0.01), and increased tumor weight (p<0.001) in tumors developing from the same number of injected cells (two-way ANOVA test).

We recovered tumor cells by paramagnetic bead selection for human integrin β1 of dispersed tumors. Analysis for TF and EPCR expression showed that irrespective of the implanted tumor cell population, the majority of outgrown tumor cells presented with a EPCR^−^/TF^high^ phenotype (Fig. 3C), indicating a conversion from EPCR^+^ to EPCR^−^ cells in the tumor microenvironment *in vivo*. Tumor cell populations recovered by outgrow of minced tumor and stained for EPCR and TF using our standard protocol confirmed that cells constituting the majority of the tumor mass were EPCR^–^ and excluded potential problems in receptor detection on freshly isolated tumor cells (data not shown). Thus, EPCR expression was associated with improved tumor take, raising the question whether EPCR directly contributed to breast tumor initiation capacity in the orthotopic microenvironment.

### Blocking EPCR attenuates tumor initiation and growth

**Figure 4:**
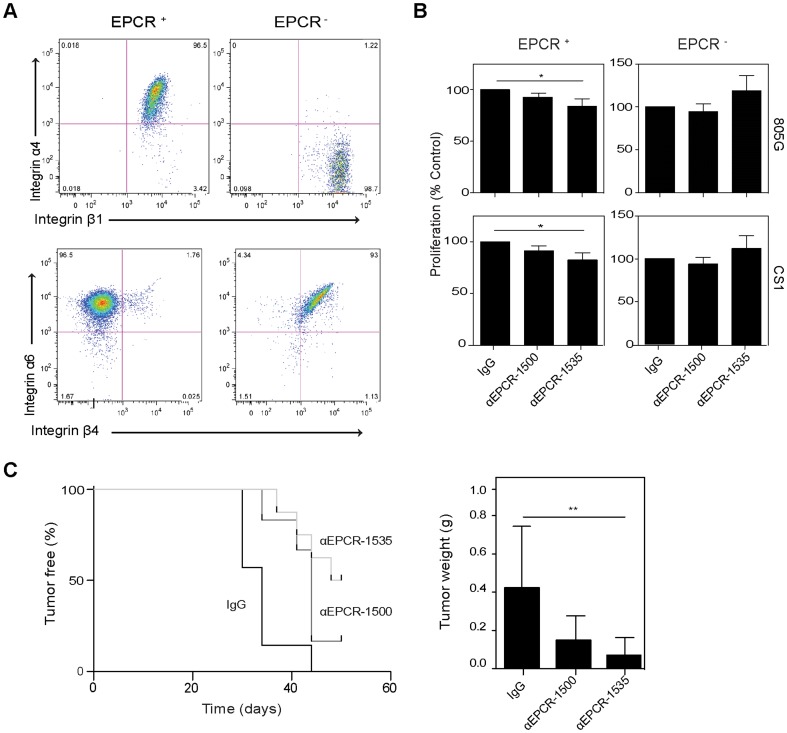
Blocking EPCR alters proliferation *in vitro* and tumor take of MDA-MB-231 mfp cells *in vivo*. **A** Sorted cells were stained for integrin subunits α4, α6, β1 and β4 and analyzed by FACS. EPCR^+^ cells are α4β1 positive whereas EPCR^−^ cells are α4 negative, but express β1. Conversely, EPCR^−^ cells expressed α6β4, and EPCR^+^ expressed α6 but not β4 integrin. **B** FACS-isolated EPCR^+^ or EPCR^−^ cells were seeded in 48-well plates coated with CS-1 (5 µg/ml), or serum-free 805 G cell supernatant diluted 1∶2 with DMEM, and cultured in the presence of 100 µg/ml control IgG, αEPCR-1500, or αEPCR-1535 for 48 hours. Cell numbers were quantified by MTT assay (different between control IgG and αEPCR-1535, *p<0.05 ANOVA, followed by Bonferroni test, n = 3, mean±SD). **C** MDA-MB-231 mfp FACS-isolated EPCR^+^ cells (10^4^/mouse) were injected into the mammary fat pad in matrigel containing 0.5 mg of either control IgG, non-blocking αEPCR-1500 or blocking αEPCR-1535. The blocking antibody significantly reduced tumor take (** p<0.005, Log-rank Test, n = 7 for IgG and αEPCR-1500, n = 8 for αEPCR-1535) and final tumor sizes (** p<0.005 versus control IgG, ANOVA with Bonferroni post test, mean±SD).

Prompted by the microarray data (Fig. 1D) demonstrating preferential expression of certain integrins by EPCR^+^ cells, we investigated integrin expression and function further. It is known from skin carcinoma that additional surface markers can separate a subpopulation with distinct tumor-initiating properties in the stem cell pool defined by high level expression of integrin α6 [45]. Whereas both EPCR^+^ and EPCR^−^ populations expressed comparable levels of integrin α6, EPCR^−^ cells expressed integrin β4 that forms the epithelial laminin receptor α6β4 (Fig. 4A). In addition, EPCR^+^ cells expressed integrin α4 that is involved in hematopoietic stem cell retention in the bone marrow [44]. Considering the distinct integrin profiles of EPCR^+^ and EPCR^−^ cells, we therefore asked whether ligation of defined integrins could uncover functions of EPCR in cell survival and/or proliferation *in vitro*. We used a pair of inhibitory (αEPCR-1535) [21], [49] and non-blocking (αEPCR-1500) [50] antibodies as well as an IgG1 isotype-matched control. These antibodies had no effect on mammosphere growth of unsorted cells (Fig. S3A), excluding non-specific general toxicity. FACS sorted cells were seeded on plates coated with the CS-1 fibronectin fragment that specifically ligates integrin α4, supernatant from 805 G cells that is enriched in the integrin α6 ligand laminin 5. After 48 hours of culture in the presence of antibodies, the inhibitory αEPCR-1535 significantly reduced numbers of viable cells as determined by the MTT assay when EPCR^+^ cells were plated on CS-1 and 805 G matrix, but the antibody had no significant inhibitory effect when added to EPCR^−^ cells (Fig. 4B). These data indicated that engagement of subpopulation-specific integrins expressed by EPCR^+^ cells enhanced proliferation or survival in dependence on EPCR interactions with an unknown or one or more of the coagulation protease ligands present in the serum-containing culture medium.

We next addressed whether blocking EPCR ligand interaction had an effect on the tumor-initiating capacity of EPCR^+^ cells. We injected 10,000 FACS-isolated EPCR^+^ cells admixed with matrigel containing the various antibodies. Whereas >80% of the mice injected with cells in the presence of control IgG or the non-inhibitory αEPCR-1500 developed tumors, tumor-take was observed in only 50% of the animals receiving cells together with the inhibitory αEPCR-1535 (Fig. 4C). The significantly reduced tumor initiation was also reflected in smaller tumor sizes at sacrifice in the group of mice receiving EPCR^+^ cells together with inhibitory antibody.

**Figure 5:**
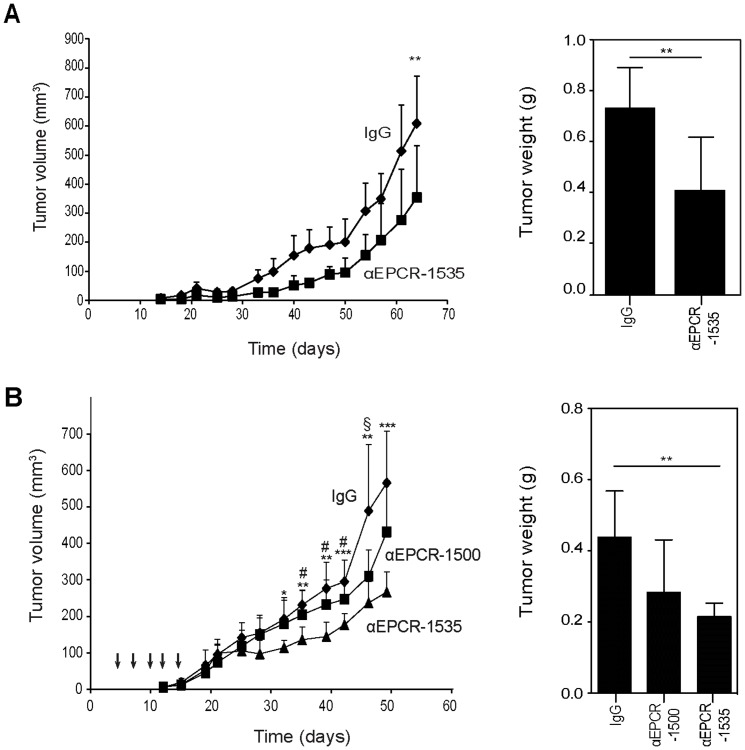
Blocking EPCR decreases tumor growth of human breast cancer cells. **A** MDA-MB-231 mfp cells (1×10^6^ cells/mouse) were mixed with 1 mg control IgG or αEPCR-1535 and injected into the mammary fat pad of SCID mice. Blocking EPCR significantly reduced tumor growth and final tumor weights (**p<0.05, T-test, mean±SD, n = 8 mice/group). **B** MDA-MB-231 mfp cells (1×10^6^ cells/mouse) were injected in the mammary fat pad of SCID mice and mice were treated at days 4, 7 10, 12, and 15 by intraperitoneal injections of 1 mg of control IgG, αEPCR-1500, or αEPCR-1535. Treatment with inhibitory αEPCR-1535 significantly reduced tumor growth and final tumor weights (ANOVA followed by Bonferroni posttest, * p<0.05, ** p<0.005, *** p<0.001 IgG versus αEPCR-1535; # p<0.05 αEPCR-1500 versus αEPCR-1535, § p<0.05 IgG versus αEPCR-1500).

We next asked whether blocking EPCR also reduced tumor growth when the cancer stem cell-like subpopulation was inoculated together with EPCR^−^ cells in the absence of matrigel. Co-injection of MDA-MB-231 mfp cells with αEPCR-1535 into the mammary gland of SCID mice resulted in a significant decrease in tumor growth and final tumor weights relative to control IgG (Fig. 5A). H&E staining of treated tumors showed similar tumor architecture between the groups (Fig. S3B). In order to rule out non-specific effects of co-injecting the antibodies with the tumor cells, we first implanted MDA-MB-231 mfp cells into the mammary glands of CB17/SCID mice and 4 days later began a treatment cycle of 5 intraperitoneal injections of IgG1 control, inhibitory αEPCR-1535, or non-inhibitory αEPCR-1500. We confirmed by FACS analysis of CHO cells overexpressing mouse EPCR that the employed mouse anti-human EPCR antibodies lacked cross-reactivity with mouse EPCR, assuring that the observed *in vivo* effects were due to specific blockade of human tumor cell expressing EPCR (data not shown). Treatment with the function-blocking αEPCR-1535 resulted in significantly slower tumor growth relative to control and non-inhibitory antibody and yielded significantly smaller final tumor sizes and weights versus control at sacrifice (Fig. 5B). Histological analysis of tumors from treated mice showed no difference in tumor architecture (Fig. S3C).

**Figure 6:**
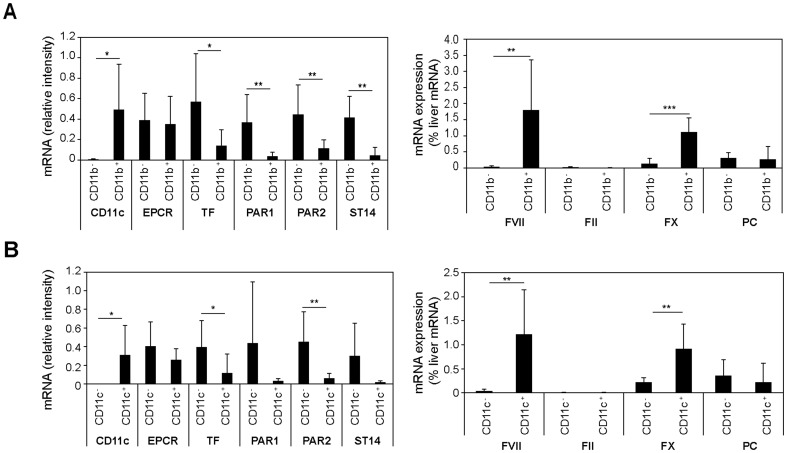
Expression analysis of PyMT tumor macrophages. PyMT tumors from heterozygous PyMT-EPCR^Low/WT^ mice were dispersed and macrophages were separated with αCD11b (A) or αCD11c (B) paramagnetic beads from tumor cells and stromal cells. Tumor macrophages were CD11b^+^/CD11c^+^/F4/80^+^ by FACS and CD11c was used to assess the efficiency of selection. Expression of the indicated mRNAs was determined by RT-PCR. Coagulation factor mRNA was normalized to a standard curve of normal mouse liver mRNA (* p<0.05, ** p<0.005 T-test, mean±SD, n = 5–8).

These data in murine and human breast cancer cells concordantly showed functional roles of EPCR in orthotopic tumor initiation and growth. The inhibition by a blocking antibody furthermore implicated ligand interactions with this receptor in tumor growth in the mammary fat pad. EPCR is known to bind three coagulation proteases, i.e. PC/aPC, FVIIa, and FX/FXa. While coagulation factor ligands for EPCR conceivably might enter the tumor microenvironment due to the hyper-permeability of the tumor vasculature, this pathway was difficult to rationalize during tumor initiation by a low number of injected cancer stem cells. We therefore returned to the PyMT model and asked whether cells in the tumor microenvironment expressed coagulation factors that interact with EPCR. Because tumor cells [51] and macrophages [52] were known to synthesize FVII, we fractionated dissociated tumor cell suspensions by αCD11b or αCD11c paramagnetic bead selection (Fig. 6A and B). Whereas the macrophage-depleted stromal fraction showed high expression of TF, PAR1, PAR2, and matriptase (ST14), a known cancer cell-expressed serine protease [53], EPCR was expressed at similar levels by macrophages and other cells in the tumor microenvironment. Most important, FVII was exclusively and FX was predominantly expressed by tumor associated macrophages. There was no detectable mRNA expression of prothrombin, but PC was expressed at low levels in both compartments. Genetic mouse strains with cell-type specific deletion of these proteases are currently not available for further analysis of relevant counter ligands for EPCR. Nevertheless, these data support the concept that EPCR^+^ cancer stem cell-like populations may be regulated by known protease ligands ectopically synthesized by cells in the tumor microenvironment.

## Discussion

Cancer stem cell-like subpopulations have attracted attention because of their increased chemotherapy and radiation resistance and their potential to be responsible for tumor relapse and metastasis under standard therapy [33], [54]. A better comprehension of cancer stem cell biology is required to develop pertinent new cancer therapies, in particular for highly aggressive basal-like breast cancer with poor prognosis and few therapeutic options after relapse. Here we provide genetic evidence that EPCR, a receptor with important roles in vascular biology and a known stem cell marker in a variety of normal stem cell pools, has a role in tumor progression of breast cancer. Furthermore, we show that an inhibitory antibody to EPCR expressed by human triple negative breast cancer cells attenuates tumor initiation and growth, indicating that ligands for EPCR present in the tumor microenvironment regulate these cancer stem cell-like populations.

Whereas hematopoietic development is predominantly driven by a hierarchical organization of stem and progenitor cells, epithelial stem cell niches frequently consist of co-existing and inter-converting stem cell populations [31]. CD44^high^/CD24^−^ and integrin α6^high^ phenotypes mark cancer stem cell pools and this profile characterizes the majority of cells in our model of highly aggressive human triple negative breast cancer. In this population, EPCR expression defines a distinct functional subpopulation with increased ALDH1 expression, ability to form non-adherent mammospheres, and markedly increased tumor-initiating capacity. However, the EPCR^+^ population was not distinguished from EPCR^-^ cells by established cancer stem cell signatures. Rather, both populations expressed markers associated with cancer stem cell-like phenotypes as well as with EMT that also characterizes breast cancer stem cells [55], [56]. Importantly, tumors initiated with either EPCR^+^ or EPCR^−^ cells yielded mixed populations, indicating plasticity to interconvert. This plasticity is consistent with prior studies that demonstrated conversion of more differentiated cells into basal-like breast cancer stem cells [32], [57], but may also indicate communication between the subpopulations *in vivo*.

Bi-directional crosstalk between the stem cell and its niche is critical in normal survival of stem cells and their differentiation [31]. The EPCR^+^ population expressed integrin α4 and α6 that both are critically important for hematopoietic stem cell homing to bone marrow niches [44]. Progenitors functionally contribute to certain stem cell niches, but hematopoietic stem cell retention and mobilization is regulated by interactions between mesenchymal niche cells and macrophages [31]. The contributions of macrophages to metastasis and tumor progression are increasingly recognized [58] and our data show that tumor-associated macrophages are the primary source for extrahepatic synthesis of the EPCR ligands FVII and FX in the tumor microenvironment. Thus, cancer stem cell expressed EPCR may transmit cues from the innate immune system to regulate stem cell survival, retention, and/or differentiation.

While appropriate genetic mouse models are currently not available to define the relevant ectopically synthesized protease ligands for EPCR in tumor progression, continuing exploration of these signaling events may yield novel therapeutic approaches. Our proof of principle experiments with an inhibitory antibody to EPCR expand the emerging evidence [34] that cancer stem cell markers can be targeted for preclinical therapeutic benefit. While EPCR plays important roles in regulating the host defense to infection [59]–[61], there are no conclusive data to indicate that long term blockade of EPCR or loss of EPCR [38] increases the risk of thrombosis and would limit the application of inhibitory antibodies to EPCR to block tumor growth.

However, the protease ligands and their proteolytic signaling effects on the stem cell populations are similarly attractive targets for cancer therapy. Small molecule anticoagulants targeting specific coagulation proteases are entering the clinic for a variety of thrombosis indications and may have unexpected therapeutic benefit in interrupting the cancer stem cell EPCR signaling pathways that are initiated by the extra-hepatic synthesis of coagulation factors by tumor-associated macrophages. Identifying EPCR's protease ligand will be instrumental to select an optimal anticoagulant strategy that synergizes with conventional cancer therapy by specifically targeting cancer stem cell-like populations as an adjuvant therapy.

## Supporting Information

Figure S1 **Metastasis and tumor analysis in the syngeneic PyMT breast cancer model.** **A** Cohorts of PyMT mice expressing very low levels of EPCR^low/WT^ (n = 29) or PyMT C57BL/6J (n = 15) were followed weekly for tumor growth. **B** Lungs of PyMT EPCR^Low/Low^ (n = 18) versus EPCR^low/WT^ (n = 16) were harvested and fixed in Bouin's solution for counting of visible surface metastases. No significant differences in lung metastatic burden were observed between PyMT-EPCR^Low/Low^ and PyMT-EPCR^Low/WT^ control cohorts (mean shown). **C** Spontaneous PyMT-EPCR^Low/Low^ and PyMT-EPCR^Low/WT^ tumors were stained with H&E in order to detect potential differences in the organization of the tumor (scale bar 50 µm). **D** EPCR^low/WT^ and EPCR^Low/Low^ tumors obtained at sacrifice were stained for CD34. Vessel density and area were quantified with the image analysis software IMARIS. Vessel size, but not density was increased (***p<0.001, Mann-Whitney test, n = 7 tumors/genotype; mean±SD). **E** Representative images of CD34 staining scale bar: 30 µm. **F** F4/80 stained tumor sections were counterstained with DAPI for total cell number to quantify macrophage counts (p = 0.1; mean±SD, n = 5 tumors/genotype).
(TIF)

Figure S2 **Analysis of in vitro growth of EPCR-deleted PyMT cells and tumor histology.** **A**. Proliferation: Cells were plated in 48-well plate for 48 hours and viable cell numbers were quantified by MTT assay (mean±SD, n = 3). **B** Mammosphere growth: 10^4^ PyMT EPCR-expressing or deleted cells/well were seeded in low attachment plates and grown in mammosphere media for 10 days. Mammosphere sizes were quantified from images with Photoshop CS4 (mean±SD, n = 3). Concordant results were obtained with an independent line isolated from a separate animal. **C** EPCR Ad5 control or Cre tumors (100 mg) were harvested and lysed in octylglucoside buffer (50 mM). Lysates were analyzed for EPCR and actin by Western Blotting. **D** Histology of EPCR Ad5 control or Cre tumors. Sections were stained with H&E; representative views from three independent animals are shown.
(TIF)

Figure S3 **Effect of anti-EPCR on mammosphere growth and tumor histology.** **A**. MDA-MB-231 mfp cells (10^4^/well) were seeded in low attachment plates with 100 µg/ml control IgG, αEPCR-1500 or αEPCR-1535 antibody in mammosphere medium, fresh antibody was added every 3 days. Blocking EPCR does not alter mammosphere formation (mean±SD, n = 2,) B. H&E staining of tumors form cells mixed with control or αEPCR-1535 antibody. **C** H&E stained sections of tumors from anti-EPCR or control antibody treated mice.
(TIF)

Table S1 **Gene list of differentially expressed transcripts between EPCR^+^/TF^low^ and EPCR^−^/TF^high^ subpopulations of MDA-MB-231mfp cells.**
(PDF)

Table S2 **Pathway and gene signature analysis of differentially regulated genes in EPCR^+^ and EPCR^−^ subpopulations of MDA-MB-231mfp cells.**
(PDF)

Table S3 **Primers used for RT-PCR analysis of gene expression by cell fractions of murine PyMT tumors.**
(PDF)
