# Supplementary figures and images for: Endothelial Protein C Receptor Function in Murine and Human Breast Cancer Development
Source: PLoS One. 2013 Apr 9;8(4):e61071. doi: 10.1371/journal.pone.0061071 (PMC3621887; doi:10.1371/journal.pone.0061071)

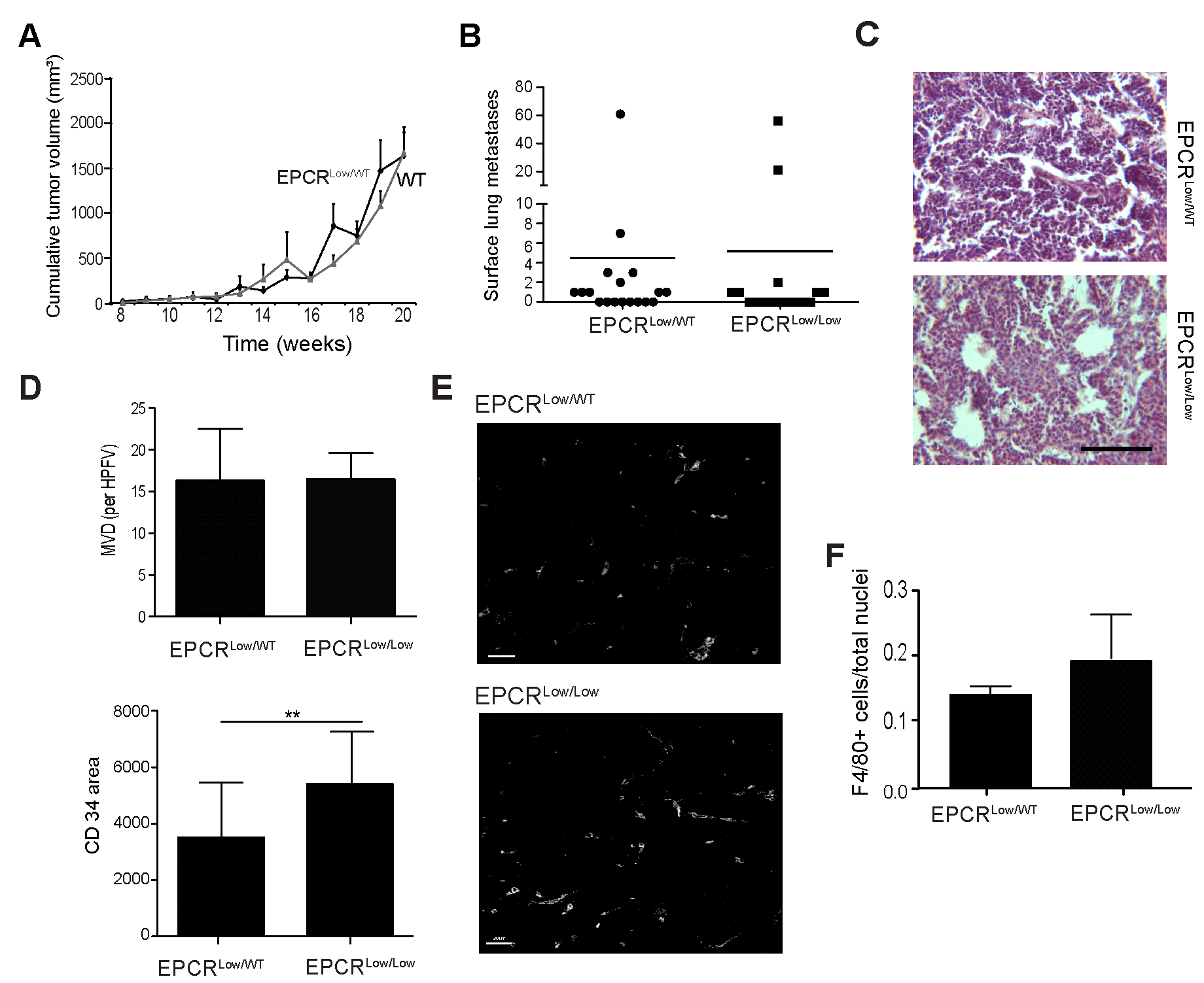

Supplement: Figure S1 — Metastasis and tumor analysis in the syngeneic PyMT breast cancer model. A Cohorts of PyMT mice expressing very low levels of EPCRlow/WT (n = 29) or PyMT C57BL/6J (n = 15) were followed weekly for tumor growth. B Lungs of PyMT EPCRLow/Low (n = 18) versus EPCRlow/WT (n = 16) were harvested and fixed in Bouin's solution for counting of visible surface metastases. No significant differences in lung metastatic burden were observed between PyMT-EPCRLow/Low and PyMT-EPCRLow/WT control cohorts (mean shown). C Spontaneous PyMT-EPCRLow/Low and PyMT-EPCRLow/WT tumors were stained with H&E in order to detect potential differences in the organization of the tumor (scale bar 50 µm). D EPCRlow/WT and EPCRLow/Low tumors obtained at sacrifice were stained for CD34. Vessel density and area were quantified with the image analysis software IMARIS. Vessel size, but not density was increased (***p<0.001, Mann-Whitney test, n = 7 tumors/genotype; mean±SD). E Representative images of CD34 staining scale bar: 30 µm. F F4/80 stained tumor sections were counterstained with DAPI for total cell number to quantify macrophage counts (p = 0.1; mean±SD, n = 5 tumors/genotype). (TIF) [file pone.0061071.s001.tif]

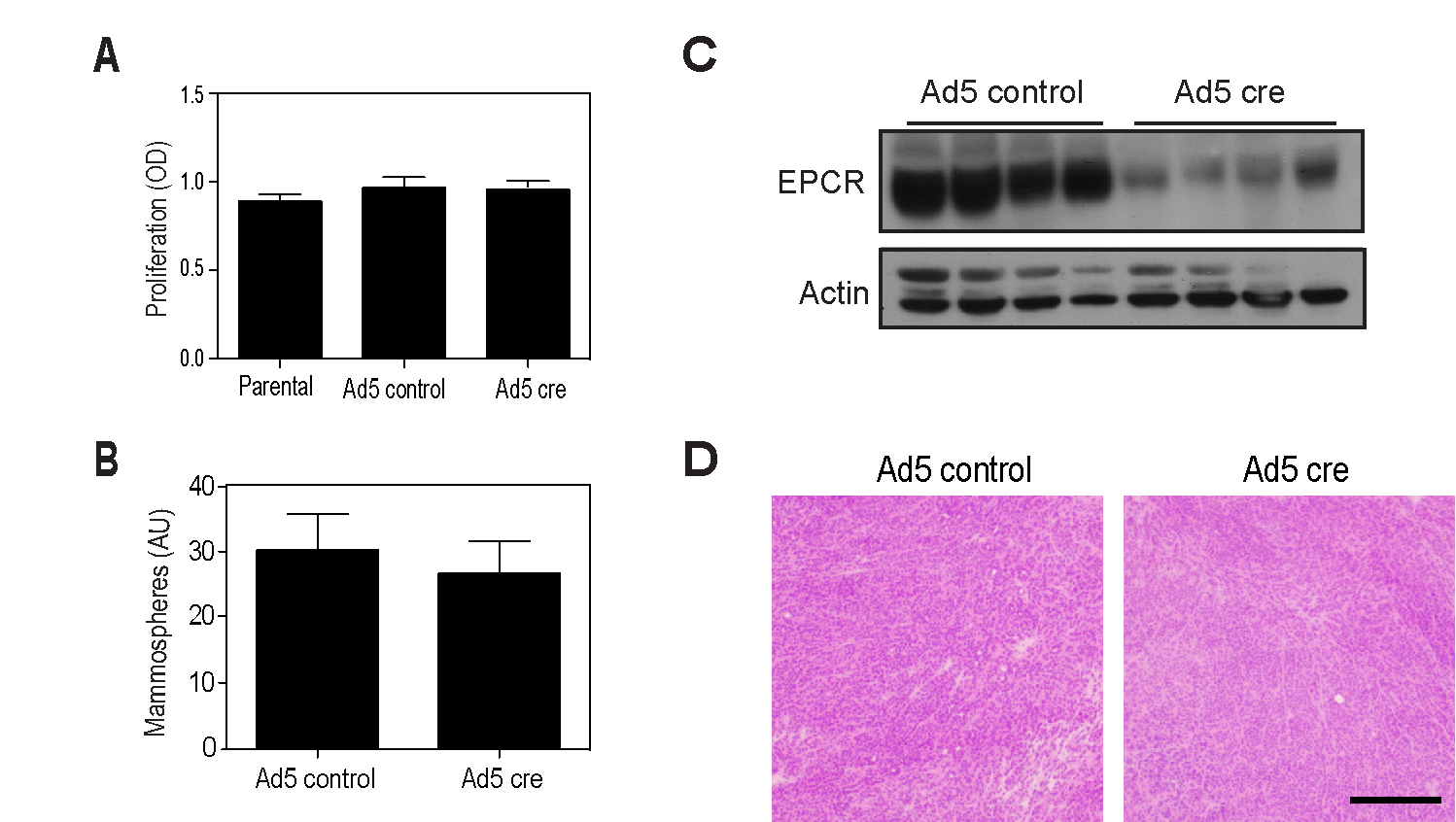

Supplement: Figure S2 — Analysis of in vitro growth of EPCR-deleted PyMT cells and tumor histology. A. Proliferation: Cells were plated in 48-well plate for 48 hours and viable cell numbers were quantified by MTT assay (mean±SD, n = 3). B Mammosphere growth: 104 PyMT EPCR-expressing or deleted cells/well were seeded in low attachment plates and grown in mammosphere media for 10 days. Mammosphere sizes were quantified from images with Photoshop CS4 (mean±SD, n = 3). Concordant results were obtained with an independent line isolated from a separate animal. C EPCR Ad5 control or Cre tumors (100 mg) were harvested and lysed in octylglucoside buffer (50 mM). Lysates were analyzed for EPCR and actin by Western Blotting. D Histology of EPCR Ad5 control or Cre tumors. Sections were stained with H&E; representative views from three independent animals are shown. (TIF) [file pone.0061071.s002.tif]

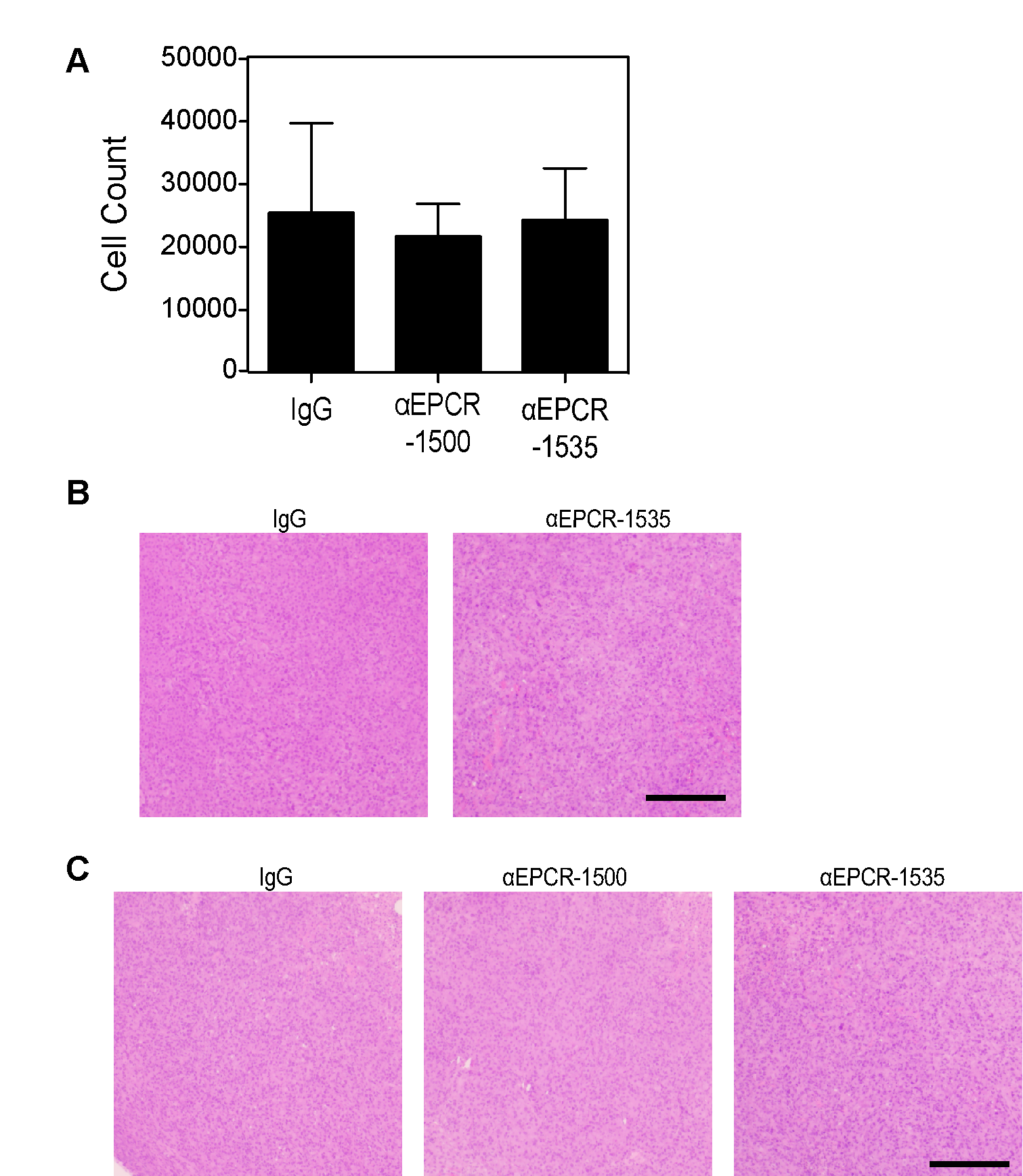

Supplement: Figure S3 — Effect of anti-EPCR on mammosphere growth and tumor histology. A. MDA-MB-231 mfp cells (104/well) were seeded in low attachment plates with 100 µg/ml control IgG, αEPCR-1500 or αEPCR-1535 antibody in mammosphere medium, fresh antibody was added every 3 days. Blocking EPCR does not alter mammosphere formation (mean±SD, n = 2,) B. H&E staining of tumors form cells mixed with control or αEPCR-1535 antibody. C H&E stained sections of tumors from anti-EPCR or control antibody treated mice. (TIF) [file pone.0061071.s003.tif]
