# Supplementary material for: Endothelial Protein C Receptor Function in Murine and Human Breast Cancer Development
Source: PLoS One. 2013 Apr 9;8(4):e61071. doi: 10.1371/journal.pone.0061071 (PMC3621887; doi:10.1371/journal.pone.0061071)
Supplement: Table S1 — Gene list of differentially expressed transcripts between EPCR+/TFlow and EPCR−/TFhigh subpopulations of MDA-MB-231mfp cells. (PDF) [file pone.0061071.s004.pdf]

Table S1: Hierarchical Clustering of differentially regulated Genes of MDA-MB-231mfp Subpopulations

idx	ENTREZID	ENSEMBL	SYMBOL	GENENAM	GENEFAMI	INICalls	PROBENUM	EPCR+ p19	EPCR+ p20	EPCR+ p21	EPCR+ p22	EPCR+ p25	AveExpr	F	P.Value	adj.P.Val	Branch ID
2878_at	2878	ENSG00000(	GPX3	glutathione	Secreted	I	21	1.882051	1.976069	2.450996	2.284756	2.068606	5.352009	249.748	4.30E-08	3.23E-06	10
23331_at	23331	ENSG00000(	TTC28	tetratricop	Secreted	I	22	2.101657	1.95521	2.399771	2.175753	1.978505	4.969621	140.6354	3.56E-07	1.05E-05	10
4217_at	4217	ENSG00000(	MAP3K5	mitogen-ac	kinase	I	20	1.894096	1.948599	2.394588	2.127352	2.280811	4.736338	353.2894	1.19E-08	1.71E-06	10
5552_at	5552	ENSG00000(	SRGN	serglycin		I	11	1.652524	2.000519	2.295417	2.10926	2.120165	10.66335	99.70631	1.25E-06	2.37E-05	10
54509_at	54509	ENSG00000(	RHOF	ras homolo	GTPase	I	31	1.877342	2.406128	2.400662	2.358891	1.900386	5.431256	176.4253	1.55E-07	6.55E-06	10
9123_at	9123	ENSG00000(	SLC16A3	solute carri	transporter	I	33	1.752751	2.291857	2.38003	2.276583	1.967203	7.826786	370.1627	1.00E-08	1.58E-06	10
23708_at	23708	ENSG00000(	GSPT2	G1 to S pha	GTPase	I	33	1.799493	2.224381	2.518536	2.374728	2.150831	4.865189	619.5156	1.49E-09	9.95E-07	10
57722_at	57722	ENSG00000(	IGDCC4	immunoglobulin super		I	22	1.879672	1.822554	2.18965	2.427787	1.897253	5.773273	54.79024	1.09E-05	0.000105	10
3909_at	3909	ENSG00000(	LAMA3	laminin, al	Secreted	I	10	1.819348	1.945491	2.093124	2.301039	2.019042	5.206345	310.0272	1.93E-08	1.99E-06	10
1028_at	1028	ENSG00000(	CDKN1C	cyclin-dependent kinas		I	28	1.553174	1.85132	2.182578	2.457416	2.071522	5.538969	181.9266	1.38E-07	6.18E-06	10
9663_at	9663	ENSG00000(	LPIN2	lipin 2		I	31	1.772101	1.899806	2.357335	2.462232	2.51464	7.000569	253.3469	4.08E-08	3.11E-06	10
1848_at	1848	ENSG00000(	DUSP6	dual specif	phosphopr	I	43	1.620365	1.801551	2.419639	2.365645	2.436243	10.56327	201.8622	9.42E-08	5.13E-06	10
54855_at	54855	ENSG00000(	FAM46C	family with sequence s		I	32	1.83125	1.829013	2.634877	2.402335	2.486316	5.505438	109.9032	8.77E-07	1.92E-05	10
9976_at	9976	ENSG00000(	CLEC2B	C-type lecti	Secreted	I	20	2.099911	1.993932	2.400581	2.349066	2.503854	5.29656	207.0912	8.57E-08	4.79E-06	10
627_at	627	ENSG00000(	BDNF	brain-deriv	Secreted	I	55	2.219673	2.067497	2.394558	2.196875	2.292128	5.028747	335.2863	1.45E-08	1.76E-06	10
27086_at	27086	ENSG00000(	FOXP1	forkhead box P1		I	123	1.761258	2.117966	2.114186	2.194834	2.532741	6.217665	433.1439	5.62E-09	1.23E-06	10
5318_at	5318	ENSG00000(	PKP2	plakophilin 2		I	18	1.675382	2.110726	2.252942	2.103568	2.434656	4.518268	160.4993	2.19E-07	7.98E-06	10
9240_at	9240	ENSG00000(	PNMA1	paraneoplastic antigen		I	11	1.665183	2.135962	2.286508	2.40652	2.61731	7.997405	205.17	8.87E-08	4.89E-06	10
126433_at	126433	ENSG00000(	FBXO27	F-box prote	Secreted	I	22	1.916148	2.221166	2.407663	2.259382	2.413008	6.711434	303.8475	2.08E-08	2.10E-06	10
58528_at	58528	ENSG00000(	RRAGD	Ras-related GTP bindir		I	33	1.939038	2.239463	2.244922	2.29448	2.401833	6.264175	134.4854	4.19E-07	1.17E-05	10
221061_at	221061	ENSG00000(	FAM171A1	family with sequence s		I	33	1.600653	2.403814	2.200798	2.236771	2.386088	5.762334	225.2701	6.29E-08	4.03E-06	10
56952_at	56952	ENSG00000(	PRTFDC1	phosphoribosyl transfe		I	44	1.651997	2.33971	1.985588	2.237005	2.529441	5.05494	197.7387	1.02E-07	5.22E-06	10
10668_at	10668	ENSG00000(	CGRRF1	cell growth regulator w		I	20	1.666881	2.195649	1.945687	2.104004	2.224983	6.845102	104.6493	1.05E-06	2.13E-05	10
5029_at	5029	ENSG00000(	P2RY2	purinergic	G-protein c	I	11	1.725137	1.979237	1.91744	2.13363	2.356481	4.481364	134.561	4.18E-07	1.17E-05	10
2295_at	2295	ENSG00000(	FOXF2	forkhead box F2		I	11	1.943984	2.444394	2.040063	1.986379	2.321482	6.412728	472.7667	4.06E-09	1.09E-06	10
26996_at	26996	ENSG00000(	GPR160	G protein-c	G-protein c	I	11	1.844608	2.196468	2.099374	1.905014	2.191081	4.883521	232.2173	5.62E-08	3.83E-06	10
29108_at	29108	ENSG00000(	PYCARD	PYD and CA	peptidase	I	22	2.244903	2.009276	2.058257	2.029988	2.25708	5.174709	95.59264	1.46E-06	2.61E-05	10
3775_at	3775	ENSG00000(	KCNK1	potassium	ion channe	I	21	2.444569	2.29563	2.424552	2.707055	2.257702	5.841322	167.1796	1.88E-07	7.28E-06	10
3486_at	3486	ENSG00000(	IGFBP3	insulin-like	Secreted	I	18	2.398283	2.334988	2.437672	2.577558	2.364452	5.478988	596.0205	1.72E-09	9.95E-07	10
286319_at	286319	ENSG00000(	TUSC1	tumor suppressor canc		I	11	2.280096	2.408763	2.359254	2.710998	2.125266	5.838743	209.7668	8.18E-08	4.71E-06	10
9435_at	9435	ENSG00000(	CHST2	carbohydrate (N-acety		I	22	2.110368	2.31632	2.373515	2.541459	2.261917	5.189652	185.2346	1.29E-07	5.92E-06	10
91752_at	91752	ENSG00000(	ZNF804A	zinc finger protein 804		I	11	2.469431	2.365898	2.006588	2.454768	1.957748	5.536932	212.8361	7.75E-08	4.54E-06	10
822_at	822	ENSG00000(	CAPG	capping protein (actin		I	25	2.06882	2.665556	2.477321	2.790684	2.163086	8.262298	714.435	8.80E-10	9.95E-07	10
140606_at	140606	ENSG00000(	SELM	selenoprot	Secreted	I	9	1.952356	2.538518	2.514561	2.569316	2.131118	5.166477	128.8994	4.89E-07	1.30E-05	10
23641_at	23641	ENSG00000(	LDOC1	leucine zipper, down-r		I	11	1.965517	2.711812	2.166099	2.896473	2.101171	5.408447	202.976	9.23E-08	5.04E-06	10
1183_at	1183	ENSG00000(	CLCN4	chloride ch	ion channe	I	44	2.065301	2.461356	2.163757	2.494663	2.36047	5.952704	164.5667	2.00E-07	7.50E-06	10
1305_at	1305	ENSG00000(	COL13A1	collagen, ty	Secreted	I	30	2.014986	2.344808	2.208682	2.400076	2.451935	10.09902	329.202	1.55E-08	1.80E-06	10
2152_at	2152	ENSG00000(	F3	coagulation factor III (t		I	31	1.944798	2.535307	2.205483	2.325356	2.29207	9.256703	441.1653	5.25E-09	1.23E-06	10
55841_at	55841	ENSG00000(	WWC3	WWC family member 3		I	27	1.87469	2.456341	2.25652	2.653195	2.442639	6.660052	113.9878	7.67E-07	1.76E-05	10
9749_at	9749	ENSG00000(	PHACTR2	phosphatase and actin		I	73	2.287502	2.455	2.159025	2.342699	2.549989	5.582131	131.0081	4.61E-07	1.25E-05	10
2770_at	2770	ENSG00000(	GNAI1	guanine nu	GTPase	I	32	2.288564	2.39625	2.17763	2.367922	2.350062	4.751797	231.3108	5.70E-08	3.86E-06	10
8738_at	8738	ENSG00000(	CRADD	CASP2 and	peptidase	I	31	2.111767	2.574581	2.01708	2.496131	2.22385	8.558798	245.0253	4.61E-08	3.37E-06	10
57636_at	57636	NA	ARHGAP23	Rho GTPase activating		I	13	2.154279	2.128725	2.129912	2.661571	2.736149	8.078439	521.0293	2.83E-09	1.03E-06	10
54996_at	54996	ENSG00000(	MOSC2	MOCO sulphurase C-te		I	10	2.279447	2.324966	2.625295	2.526791	2.46522	5.917101	303.2407	2.10E-08	2.10E-06	10
1009_at	1009	ENSG00000(	CDH11	cadherin 11, type 2, OI		I	32	2.2388	2.197274	2.588615	2.584793	2.585934	6.095524	408.0246	7.01E-09	1.26E-06	10
3899_at	3899	ENSG00000(	AFF3	AF4/FMR2 family, mer		I	40	2.177913	2.024298	2.746461	2.449543	2.56774	5.000227	473.4991	4.04E-09	1.09E-06	10
51700_at	51700	ENSG00000(	CYB5R2	cytochrome b5 reducta		I	11	2.106177	2.211324	2.595878	2.90898	2.593538	5.937367	530.5118	2.65E-09	1.03E-06	10
23564_at	23564	NA	DDAH2	dimethylarginine dime		I	9	2.127543	2.308223	2.780863	2.74348	2.358044	7.658115	204.8646	8.92E-08	4.89E-06	10
286499_at	286499	ENSG00000(	FAM133A	family with sequence s		I	22	2.341649	2.405141	2.834712	2.477142	2.577798	6.541085	372.2757	9.84E-09	1.57E-06	10
55536_at	55536	ENSG00000(	CDCA7L	cell division cycle asso		I	18	2.196589	2.592046	2.595884	2.48188	2.558022	8.404083	146.2198	3.08E-07	9.69E-06	10
203328_at	203328	ENSG00000(	SUSD3	sushi domain containir		I	11	2.505183	2.418458	2.535936	2.313929	2.489438	5.055084	71.00387	4.29E-06	5.42E-05	10
8706_at	8706	ENSG00000(	B3GALNT1	beta-1,3-N-acetylgalac		I	33	1.745328	2.365235	1.96189	2.099097	1.974744	5.794603	226.4137	6.17E-08	4.01E-06	10
2260_at	2260	ENSG00000(	FGFR1	fibroblast g	kinase	I	64	1.778525	2.317401	1.917389	2.031755	1.989685	5.602274	366.5751	1.04E-08	1.60E-06	10
120114_at	120114	ENSG00000(	FAT3	FAT tumor suppressor		I	11	1.760888	2.600863	1.848031	2.184021	2.013271	4.609644	131.0515	4.60E-07	1.25E-05	10
51228_at	51228	ENSG00000(	GLTP	glycolipid t	transporter	I	10	1.647401	2.429961	1.708872	1.94917	1.918417	8.637708	349.8706	1.24E-08	1.73E-06	10
57153_at	57153	ENSG00000(	SLC44A2	solute carri	transporter	I	33	1.678711	2.406421	1.784844	1.957232	1.703398	6.030219	179.5708	1.45E-07	6.27E-06	10
23352_at	23352	ENSG00000(	UBR4	ubiquitin p	Secreted	I	8	1.891976	2.458963	1.669124	2.102439	1.679521	9.475874	193.5112	1.10E-07	5.41E-06	10
63910_at	63910	ENSG00000(	SLC17A9	solute carri	transporter	I	11	1.837112	2.516254	2.199938	2.386197	1.874205	5.971372	246.1455	4.53E-08	3.34E-06	10
4005_at	4005	ENSG00000(	LMO2	LIM domain only 2 (rho		I	11	1.797737	2.468545	2.058403	2.335912	1.777072	5.010912	94.73938	1.51E-06	2.67E-05	10
6447_at	6447	ENSG00000(	SCG5	secretogra	Secreted	I	10	1.820014	2.289638	1.95339	2.509058	1.864112	6.294283	113.3744	7.82E-07	1.77E-05	10
64789_at	64789	ENSG00000(	DEM1	defects in morphology		I	22	1.972002	2.702404	2.240567	2.198419	1.52468	4.575603	359.6727	1.12E-08	1.67E-06	10
54566_at	54566	ENSG00000(	EPB41L4B	erythrocyte membrane		I	37	2.187963	2.579106	1.98022	2.085453	1.584369	5.093561	424.3063	6.06E-09	1.23E-06	10
4015_at	4015	ENSG00000(	LOX	lysyl oxidas	Secreted	I	57	2.02083	2.297965	2.106234	2.047313	1.256266	5.036931	150.9391	2.74E-07	8.95E-06	10
1293_at	1293	ENSG00000(	COL6A3	collagen, ty	Secreted	I	33	1.858483	2.330808	2.854969	2.243861	1.923814	6.15757	176.7064	1.54E-07	6.53E-06	10
8793_at	8793	ENSG00000(	TNFRSF10D	tumor necrosis factor r		I	21	2.05268	2.406412	2.756307	2.140238	1.78094	4.83446	77.76342	3.09E-06	4.36E-05	10
1491_at	1491	ENSG00000(	CTH	cystathionase (cystath		I	10	1.689909	2.583783	2.695932	2.253318	2.023439	6.680799	115.005	7.43E-07	1.71E-05	10
11199_at	11199	ENSG00000(	ANXA10	annexin A10		I	10	1.841719	2.693118	2.687295	2.13785	1.734112	4.715073	198.5036	1.00E-07	5.18E-06	10
389336_at	389336	ENSG00000(	C5orf46	chromosome 5 open re		I	9	1.918723	3.031279	2.752098	2.220195	1.961772	5.648314	183.8829	1.33E-07	6.04E-06	10
196051_at	196051	ENSG00000(	PPAPDC1A	phosphatidic acid phos		I	11	1.275649	2.582809	2.476705	2.390932	1.684719	5.986639	99.39453	1.26E-06	2.38E-05	10
3569_at	3569	ENSG00000(	IL6	interleukin	Secreted	I	11	1.644688	2.182089	2.615712	2.328886	1.538838	5.800776	12.6614	0.00168	0.005552	10
84171_at	84171	ENSG00000(	LOXL4	lysyl oxidas	Secreted	I	21	2.407432	2.047655	2.197495	1.888867	1.665514	5.202012	141.9508	3.44E-07	1.03E-05	10
64097_at	64097	ENSG00000(	EPB41L4A	erythrocyte membrane		I	11	2.317845	1.83488	2.206957	1.836935	1.664842	4.539606	121.5917	6.06E-07	1.51E-05	10
1474_at	1474	ENSG00000(	CST6	cystatin E/I	Secreted	I	11	2.072439	2.068479	1.992305	1.987305	1.61527	5.172088	37.71271	4.13E-05	0.000285	10
445_at	445	ENSG00000(	ASS1	argininosuccinate synt		I	4	2.04991	2.050052	2.018562	1.734324	1.68673	6.387168	41.3302	2.99E-05	0.000223	10
10873_at	10873	ENSG00000(	ME3	malic enzyme 3, NADP		I	19	2.242579	2.374873	2.146252	1.740385	1.741695	5.607863	191.0186	1.15E-07	5.52E-06	10
51316_at	51316	ENSG00000(	PLAC8	placenta-sp	Secreted	I	34	2.085474	2.239003	2.218575	1.795283	1.575615	5.980728	158.5886	2.29E-07	8.07E-06	10
132430_at	132430	NA	PABPC4L	poly(A) binding protein		I	11	2.325506	2.313024	2.084965	1.994617	1.814217	5.071716	286.3769	2.59E-08	2.34E-06	10
7220_at	7220	ENSG00000(	TRPC1	transient re	ion channe	I	11	2.349942	1.891756	2.238972	2.259559	1.554431	4.693701	364.8792	1.06E-08	1.61E-06	10
316_at	316	ENSG00000(	AOX1	aldehyde oxidase 1		I	11	2.518443	2.628296	2.746408	1.897001	1.620555	7.322787	182.8153	1.36E-07	6.13E-06	10
3589_at	3589	ENSG00000(	IL11	interleukin	Secreted	I	31	2.718477	2.708765	2.241238	2.028264	1.3943	5.478972	230.7556	5.75E-08	3.86E-06	10

4489_at	4489	ENSG0000(	MT1A	metallothionein 1A		I	16	2.532907	2.112365	2.827908	1.992546	1.796112	5.731385	155.4188	2.46E-07	8.39E-06	10
84985_at	84985	ENSG0000(	FAM83A	family with sequence s		I	22	2.496242	2.105033	2.44945	1.766923	1.924367	5.754253	340.7237	1.36E-08	1.75E-06	10
56935_at	56935	ENSG0000(	C11orf75	chromosome 11 open		I	27	2.378647	2.673088	2.427008	2.361344	2.310233	5.298667	209.9769	8.15E-08	4.71E-06	10
284406_at	284406	ENSG0000(	ZFP82	zinc finger protein 82 h		I	11	2.280076	2.522976	2.29086	2.315699	2.146753	5.370483	128.7389	4.91E-07	1.31E-05	10
53358_at	53358	ENSG0000(	SHC3	SHC (Src homology 2 d		I	10	2.268492	2.601766	2.681687	2.276866	2.146727	5.328384	92.40584	1.65E-06	2.85E-05	10
9353_at	9353	ENSG0000(	SLIT2	slit homolc	G-protein c	I	10	2.318689	2.972271	2.22825	2.42144	1.75591	6.327417	41.70165	2.90E-05	0.000218	10
23286_at	23286	ENSG0000(	WWC1	WW and C2 domain cc		I	10	2.717546	2.763234	2.044193	2.159061	2.359371	5.21475	271.0963	3.18E-08	2.63E-06	10
90952_at	90952	ENSG0000(	ESAM	endothelia	Secreted	I	11	2.239165	2.648708	2.140234	1.720359	2.253392	7.665703	481.5934	3.79E-09	1.07E-06	10
995_at	995	ENSG0000(	CDC25C	cell divisior	phosphopr	I	21	1.908521	2.686796	1.80918	1.701104	2.296074	5.506459	309.1828	1.95E-08	1.99E-06	10
1366_at	1366	ENSG0000(	CLDN7	claudin 7		I	11	2.42252	3.057707	2.733755	2.999842	2.970088	6.153215	172.0234	1.70E-07	6.91E-06	10
1902_at	1902	ENSG0000(	LPAR1	lysophospl	G-protein c	I	44	2.349236	2.974738	2.654074	3.110148	2.985056	6.838745	340.7988	1.36E-08	1.75E-06	10
154_at	154	ENSG0000(	ADRB2	adrenergic,	G-protein c	I	11	2.305056	3.21701	2.734328	3.165672	3.12905	8.870318	122.8424	5.84E-07	1.47E-05	10
730755_at	730755	NA	LOC73075!	keratin associated prot		I	11	2.510878	3.234097	2.585206	3.046239	2.913146	7.155724	185.7741	1.28E-07	5.89E-06	10
9060_at	9060	ENSG0000(	PAPSS2	3'-phosphc	kinase	I	42	2.737338	3.077666	2.620948	3.029169	2.678809	6.322276	159.7974	2.22E-07	8.02E-06	10
147372_at	147372	ENSG0000(	CCBE1	collagen and calcium b		I	22	2.772541	2.92163	2.595653	2.799355	2.540211	5.062028	327.3268	1.58E-08	1.80E-06	10
652_at	652	ENSG0000(	BMP4	bone morp	Secreted	I	11	2.639677	2.950663	2.848247	2.826472	2.918045	7.625035	370.9011	9.97E-09	1.58E-06	10
3814_at	3814	ENSG0000(	KISS1	KiSS-1 met:	Secreted	I	20	2.632259	3.026951	2.530188	2.629317	2.756486	6.508865	102.627	1.13E-06	2.23E-05	10
5764_at	5764	ENSG0000(	PTN	pleiotrophi	Secreted	I	17	2.532552	2.73335	3.082735	2.511406	3.008821	5.977024	149.2929	2.86E-07	9.19E-06	10
3487_at	3487	ENSG0000(	IGFBP4	insulin-like	Secreted	I	42	2.355514	2.58103	3.081358	2.580004	2.802589	6.128376	78.12946	3.04E-06	4.31E-05	10
7923_at	7923	NA	HSD17B8	hydroxyste	Secreted	I	10	2.587209	2.887106	3.144279	2.544821	2.681902	6.467067	497.2406	3.37E-09	1.03E-06	10
131408_at	131408	ENSG0000(	FAM131A	family with sequence s		I	11	2.463861	3.147959	2.68665	3.02356	2.318016	5.598735	191.9363	1.13E-07	5.51E-06	10
28986_at	28986	ENSG0000(	MAGEH1	melanoma antigen fan		I	11	2.259113	3.188713	2.840024	2.995109	2.354562	5.602454	277.002	2.93E-08	2.53E-06	10
23180_at	23180	ENSG0000(	RFTN1	raftlin, lipid raft linker		I	22	2.36017	3.049612	2.38119	2.781432	2.400178	6.665372	375.7845	9.50E-09	1.56E-06	10
10468_at	10468	ENSG0000(	FST	follistatin	Secreted	I	31	2.137171	3.054033	2.466842	2.746093	2.281468	7.734078	94.98391	1.49E-06	2.65E-05	10
4016_at	4016	ENSG0000(	LOXL1	lysyl oxida:	Secreted	I	7	2.549538	3.299522	2.836823	2.454615	2.240316	4.971397	101.5156	1.17E-06	2.28E-05	10
201799_at	201799	ENSG0000(	TMEM154	transmeml	Secreted	I	43	2.082737	2.68754	2.412123	2.91687	2.903634	6.49042	515.7413	2.94E-09	1.03E-06	10
397_at	397	ENSG0000(	ARHGDIB	Rho GDP dissociation i		I	22	2.060733	2.583117	2.412438	2.901671	2.942602	8.506669	535.2764	2.56E-09	1.03E-06	10
3429_at	3429	ENSG0000(	IFI27	interferon, alpha-indu		I	14	2.02006	2.532159	2.569783	2.995821	2.779739	9.460491	19.97641	0.000373	0.001604	10
7580_at	7580	ENSG0000(	ZNF32	zinc finger protein 32		I	11	1.800057	2.757522	2.435806	2.756316	2.981347	6.998893	299.3708	2.20E-08	2.13E-06	10
2810_at	2810	ENSG0000(	SFN	stratifin	Secreted	I	11	2.088555	3.003533	2.364713	2.881913	2.758613	7.614127	386.3009	8.58E-09	1.47E-06	10
10581_at	10581	ENSG0000(	IFITM2	interferon induced trai		I	22	2.163685	2.88174	2.307699	2.805265	2.680453	8.95059	328.4792	1.56E-08	1.80E-06	10
5054_at	5054	ENSG0000(	SERPINE1	serpin pept	peptidase	I	30	2.201691	2.739968	2.288157	2.771195	2.961191	8.513335	350.9215	1.22E-08	1.73E-06	10
2621_at	2621	ENSG0000(	GAS6	growth arr	Secreted	I	22	2.064627	2.632835	2.42069	2.738814	2.543177	8.942278	423.8548	6.09E-09	1.23E-06	10
4199_at	4199	ENSG0000(	ME1	malic enzyme 1, NADP		I	22	2.030997	2.578712	2.38932	2.817078	2.453065	5.849157	377.8064	9.31E-09	1.56E-06	10
7805_at	7805	ENSG0000(	LAPTM5	lysosomal protein tran		I	21	1.959874	2.561656	2.527386	2.960515	2.467961	7.419736	225.7236	6.24E-08	4.03E-06	10
11167_at	11167	ENSG0000(	FSTL1	follistatin-l	Secreted	I	22	1.801306	2.483897	2.4735	2.658103	2.63131	6.405987	550.6025	2.31E-09	1.03E-06	10
54541_at	54541	ENSG0000(	DDIT4	DNA-damage-inducible		I	11	1.772624	2.425286	2.512547	2.543053	2.678639	8.709324	581.7727	1.88E-09	9.95E-07	10
64663_at	64663	ENSG0000(	SPANXC	SPANX family, membe		I	4	2.342604	2.4351	2.576253	3.127598	2.675533	6.074158	129.6572	4.79E-07	1.29E-05	10
51097_at	51097	ENSG0000(	SCCPDH	saccharopine dehydro		I	17	2.475079	2.565393	2.56413	2.741656	2.600691	5.873336	790.3239	6.05E-10	9.95E-07	10
1002_at	1002	ENSG0000(	CDH4	cadherin 4, type 1, R-c		I	9	2.55538	3.422837	1.655367	2.189787	2.816057	5.948013	235.4355	5.34E-08	3.72E-06	10
586_at	586	ENSG0000(	BCAT1	branched chain amino		I	53	1.415958	1.932823	1.539551	1.995204	1.591213	4.597398	108.3487	9.23E-07	1.99E-05	10
285440_at	285440	ENSG0000(	CYP4V2	cytochrome P450, fam		I	22	1.300751	1.770808	1.460776	1.86576	1.567145	5.115025	173.2661	1.65E-07	6.81E-06	10
2217_at	2217	ENSG0000(	FCGRT	Fc fragment of IgG, rec		I	34	1.529589	1.762852	1.525853	1.855798	1.47086	7.266198	286.2063	2.60E-08	2.34E-06	10
5532_at	5532	ENSG0000(	PPP3CB	protein ph	phosphopr	I	22	1.418369	1.837717	1.685962	1.785121	1.691471	7.305341	98.77462	1.29E-06	2.41E-05	10
8507_at	8507	ENSG0000(	ENC1	ectodermal-neural cor		I	22	1.497736	1.811343	1.473575	1.772801	1.742102	8.374233	229.37	5.88E-08	3.90E-06	10
1364_at	1364	ENSG0000(	CLDN4	claudin 4		I	33	1.629275	1.794638	1.615881	2.216297	1.899469	5.887257	170.1891	1.76E-07	7.03E-06	10
54681_at	54681	ENSG0000(	P4HTM	prolyl 4-hydroxylase, t		I	30	1.51163	1.635819	1.586073	2.124913	1.791502	5.067462	297.8959	2.24E-08	2.15E-06	10
6286_at	6286	ENSG0000(	S100P	S100 calcium binding p		I	11	1.816518	1.951182	1.709337	2.167933	1.24681	5.178219	20.26795	0.000356	0.00154	10
64838_at	64838	ENSG0000(	FNDC4	fibronectin type III dor		I	11	1.828137	1.880294	1.834301	1.930835	1.28525	5.583785	148.1797	2.93E-07	9.35E-06	10
10810_at	10810	ENSG0000(	WASF3	WAS protein family, m		I	31	1.842822	2.108636	1.88498	2.053154	1.471389	6.338954	161.4307	2.14E-07	7.88E-06	10
1662_at	1662	ENSG0000(	DDX10	DEAD (Asp-Glu-Ala-As		I	5	1.720086	1.777404	1.793269	2.064526	1.70511	9.811626	180.5717	1.42E-07	6.21E-06	10
4609_at	4609	ENSG0000(	MYC	v-myc myelocytomato:		I	11	1.762738	1.600127	1.753629	1.876925	1.697734	10.71957	50.89067	1.42E-05	0.000128	10
5327_at	5327	ENSG0000(	PLAT	plasminoge	peptidase	I	75	1.472443	1.697218	1.96765	1.915745	1.445105	9.374472	239.7895	4.99E-08	3.54E-06	10
6304_at	6304	ENSG0000(	SATB1	SATB homeobox 1		I	32	1.308841	1.721929	1.643333	1.739883	1.425774	5.149793	183.1439	1.35E-07	6.10E-06	10
25803_at	25803	ENSG0000(	SPDEF	SAM pointed domain c		I	32	1.201035	1.79371	1.63533	1.824321	1.326737	5.390917	79.8271	2.81E-06	4.08E-05	10
2919_at	2919	ENSG0000(	CXCL1	chemokine	Secreted	I	27	1.20915	1.772341	1.680816	2.013927	1.286327	6.531462	11.09201	0.002558	0.007899	10
6494_at	6494	ENSG0000(	SIPA1	signal-induced prolifer		I	11	1.257468	2.199127	1.611869	1.818467	1.339425	5.551759	155.941	2.43E-07	8.31E-06	10
3689_at	3689	ENSG0000(	ITGB2	integrin, be	Secreted	I	10	1.05276	1.988813	1.886476	1.945456	1.284045	4.409273	42.68351	2.67E-05	0.000205	10
1472_at	1472	ENSG0000(	CST4	cystatin S	Secreted	I	11	1.512001	1.725985	1.920689	2.327565	1.088706	6.693116	350.0218	1.24E-08	1.73E-06	10
10124_at	10124	ENSG0000(	ARL4A	ADP-ribosy	GTPase	I	18	1.464368	1.674634	1.772284	1.611916	1.646856	7.006523	142.0452	3.43E-07	1.02E-05	10
26579_at	26579	ENSG0000(	MYEOV	myeloma overexpress		I	22	1.407121	1.77618	1.821885	1.516852	1.634948	7.907007	105.111	1.03E-06	2.11E-05	10
5819_at	5819	ENSG0000(	PVRL2	poliovirus receptor-rel		I	13	1.285826	1.65538	1.852703	1.6651	1.601475	7.525393	218.0629	7.09E-08	4.30E-06	10
23048_at	23048	ENSG0000(	FNBP1	formin binding protein		I	20	1.508305	1.538723	1.731494	1.382895	1.599983	6.113788	163.8532	2.03E-07	7.59E-06	10
771_at	771	ENSG0000(	CA12	carbonic ar	Secreted	I	44	1.438569	1.545349	2.060787	1.544101	1.927583	4.86164	118.8294	6.59E-07	1.58E-05	10
55612_at	55612	ENSG0000(	FERMT1	fermitin family membe		I	33	1.417813	1.662847	2.05986	1.621613	1.746446	4.571013	299.9337	2.19E-08	2.13E-06	10
9447_at	9447	ENSG0000(	AIM2	absent in melanoma 2		I	21	1.560396	1.406641	1.765103	1.794018	1.643484	5.574922	109.2064	8.97E-07	1.96E-05	10
122060_at	122060	ENSG0000(	SLAIN1	SLAIN motif family, me		I	32	1.438339	1.395333	1.805263	1.698051	1.645502	4.074553	133.0463	4.36E-07	1.21E-05	10
399818_at	399818	ENSG0000(	METTL10	methyltransferase like		I	32	1.541618	1.449581	2.023672	1.745599	1.689144	5.232097	139.2341	3.69E-07	1.07E-05	10
9532_at	9532	ENSG0000(	BAG2	BCL2-associated athan		I	22	1.406397	1.352725	1.890383	1.719808	1.816466	8.199632	154.6711	2.51E-07	8.48E-06	10
65981_at	65981	ENSG0000(	CAPRIN2	caprin family member		I	28	1.373876	1.400908	1.765938	1.555217	1.74595	6.181018	209.461	8.22E-08	4.71E-06	10
5522_at	5522	ENSG0000(	PPP2R2C	protein phosphatase 2		I	27	1.775863	1.255954	2.150291	1.669388	1.550383	4.415186	87.19759	2.04E-06	3.29E-05	10
55332_at	55332	ENSG0000(	DRAM1	DNA-damage regulate		I	20	1.713071	1.352245	1.965652	1.580896	1.759944	5.747029	89.75002	1.83E-06	3.05E-05	10
8638_at	8638	ENSG0000(	OASL	2'-5'-oligoadenylate sy		I	11	1.679057	1.143264	1.708414	1.727401	1.546327	6.775522	15.38491	0.000892	0.00328	10
2651_at	2651	ENSG0000(	GCNT2	glucosamir	Secreted	I	52	1.368056	1.667016	1.799619	1.673176	1.333163	5.543856	149.5518	2.84E-07	9.18E-06	10
968_at	968	ENSG0000(	CD68	CD68 molecule		I	21	1.30263	1.644491	1.783253	1.546803	1.285593	6.328842	75.23765	3.48E-06	4.73E-05	10
118429_at	118429	ENSG0000(	ANTXR2	anthrax to:	Secreted	I	42	1.512214	1.576968	1.801036	1.677411	1.109265	7.282549	239.0511	5.05E-08	3.57E-06	10
24147_at	24147	ENSG0000(	FJX1	four jointe	Secreted	I	11	1.312839	1.37379	2.047451	1.265065	1.381212	7.573581	158.1183	2.31E-07	8.09E-06	10
22943_at	22943	ENSG0000(	DKK1	dickkopf hc	Secreted	I	10	1.219957	1.508045	1.804836	1.327898	1.407591	11.90104	36.27416	4.74E-05	0.000316	10
55780_at	55780	ENSG0000(	C6orf70	chromosome 6 open r		I	8	1.581532	1.363808	2.231062	1.648528	1.045027	6.241448	38.6648	3.79E-05	0.000267	10
55454_at	55454	ENSG0000(	CSGALNAC	chondroitir	Secreted	I	3	1.616263	1.563678	1.464763	1.673741	1.578368	9.368215	57.56208	9.15E-06	9.27E-05	10
6920_at	6920	ENSG0000(	TCEA3	transcription elongatic		I	20	1.614609	1.585174	1.443575	1.693506	1.505723	6.340388	323.6942	1.65E-08	1.86E-06	10
4124_at	4124	ENSG0000(	MAN2A1	mannosidase, alpha, cl		I	11	1.522874	1.733583	1.371182	1.530482	1.562113	8.900079	139.2932	3.68E-07	1.07E-05	10

25796_at	25796	ENSG0000(	PGLS	6-phospho	Secreted	I	9	1.584062	1.780502	1.373854	1.657389	1.274672	9.630291	255.6374	3.94E-08	3.05E-06	10
2534_at	2534	ENSG0000(	FYN	FYN oncog	kinase	I	8	1.764065	1.690045	1.548573	1.615391	1.461001	5.862003	107.0611	9.65E-07	2.03E-05	10
84260_at	84260	ENSG0000(	TCHP	trichoplein, keratin fila		I	32	1.698073	1.702833	1.498259	1.513064	1.612453	6.36122	204.9613	8.90E-08	4.89E-06	10
1956_at	1956	ENSG0000(	EGFR	epidermal ¡	kinase	I	69	1.877883	1.845954	1.57775	1.53397	1.621534	4.614529	232.8723	5.56E-08	3.81E-06	10
3384_at	3384	ENSG0000(	ICAM2	intercellular adhesion		I	40	1.78604	1.998611	1.113581	1.639011	1.338134	5.013995	173.2427	1.65E-07	6.81E-06	10
3037_at	3037	ENSG0000(	HAS2	hyaluronan synthase 2		I	32	1.691824	1.9411	1.138059	1.425304	1.583913	4.294039	181.5713	1.39E-07	6.18E-06	10
389432_at	389432	ENSG0000(	SAMD5	sterile alpha motif don		I	11	1.499806	2.081109	1.826842	1.548623	1.45029	4.737739	48.10449	1.74E-05	0.00015	10
22936_at	22936	ENSG0000(	ELL2	elongation factor, RNA		I	31	1.569894	1.894679	1.734971	1.391167	1.306988	6.477457	293.6822	2.36E-08	2.22E-06	10
23268_at	23268	ENSG0000(	DNMBP	dynamin bi	Secreted	I	21	1.555498	1.787883	1.810427	1.649665	1.449245	6.513716	117.3587	6.90E-07	1.63E-05	10
2272_at	2272	ENSG0000(	FHIT	fragile histidine triad g		I	20	1.423287	1.803885	1.773914	1.566716	1.413466	5.254741	137.8415	3.83E-07	1.10E-05	10
5468_at	5468	ENSG0000(	PPARG	peroxisom	G-protein c	I	21	1.721781	2.041694	1.658748	1.704905	1.582559	7.142045	139.0821	3.70E-07	1.07E-05	10
282974_at	282974	ENSG0000(	STK32C	serine/thre	kinase	I	53	1.808699	2.122677	1.598906	1.626841	1.340681	5.65336	152.9059	2.62E-07	8.70E-06	10
55897_at	55897	ENSG0000(	MESP1	mesoderm posterior 1		I	11	1.662149	2.06789	1.580736	1.533352	0.994429	5.87975	227.2552	6.09E-08	3.98E-06	10
3233_at	3233	ENSG0000(	HOXD4	homeobox D4		I	11	1.525313	2.065141	1.505672	1.297042	1.107005	4.590711	83.74451	2.36E-06	3.64E-05	10
89894_at	89894	ENSG0000(	TMEM116	transmembrane protei		I	11	1.742112	2.108247	1.542978	1.954762	1.56056	6.992421	241.6054	4.86E-08	3.45E-06	10
79041_at	79041	ENSG0000(	TMEM38A	transmemb	ion channe	I	20	1.726621	2.066135	1.357762	1.966661	1.644948	5.982724	283.2701	2.70E-08	2.41E-06	10
9241_at	9241	ENSG0000(	NOG	noggin	Secreted	I	11	1.534577	2.090683	1.393096	2.032381	1.532741	6.189582	85.16211	2.22E-06	3.50E-05	10
1438_at	1438	ENSG0000(	CSF2RA	colony stimulating fact		I	35	1.667302	2.315255	1.122078	1.878912	1.304381	4.536527	37.95701	4.04E-05	0.00028	10
337876_at	337876	ENSG0000(	CHSY3	chondroitin sulfate syr		I	22	1.785051	2.154882	2.169502	1.617673	1.419416	4.509002	333.5895	1.48E-08	1.76E-06	10
3110_at	3110	ENSG0000(	MNX1	motor neuron and pan		I	11	1.713077	2.223811	2.018695	1.716483	1.403946	5.074718	63.60734	6.39E-06	7.19E-05	10
1847_at	1847	ENSG0000(	DUSP5	dual specif	phosphopr	I	10	1.851631	2.109564	2.108631	1.796571	1.554798	8.913163	159.3019	2.25E-07	8.07E-06	10
64399_at	64399	ENSG0000(	HHIP	hedgehog i	Secreted	I	31	1.862381	2.448402	2.241413	1.811543	1.444494	4.913937	373.1275	9.75E-09	1.57E-06	10
5603_at	5603	ENSG0000(	MAPK13	mitogen-ac	kinase	I	32	1.593789	2.43117	1.934705	1.781661	1.354123	5.280654	121.8311	6.01E-07	1.50E-05	10
256979_at	256979	ENSG0000(	SUN3	Sad1 and UNC84 doma		I	11	1.420785	2.363018	1.930617	2.026712	1.155444	4.354247	57.41427	9.24E-06	9.32E-05	10
6781_at	6781	ENSG0000(	STC1	stanniocalc	Secreted	I	44	1.656607	2.008293	2.26156	1.496223	0.833436	7.849447	195.0867	1.07E-07	5.35E-06	10
9034_at	9034	ENSG0000(	CCRL2	chemokine	G-protein c	I	22	1.722181	2.05089	1.983529	1.432595	0.862091	7.14971	270.9279	3.18E-08	2.63E-06	10
3552_at	3552	ENSG0000(	IL1A	interleukin 1, alpha		I	11	1.048263	2.421025	1.573068	2.277593	1.10515	5.121143	34.62033	5.59E-05	0.000359	10
64750_at	64750	ENSG0000(	SMURF2	SMAD specific E3 ubiq		I	30	1.740292	1.802602	1.945854	2.04649	2.119774	8.305685	425.6629	5.99E-09	1.23E-06	10
22875_at	22875	ENSG0000(	ENPP4	ectonucleo	Secreted	I	33	1.733802	1.776171	1.900558	2.093434	1.90759	4.738567	52.63839	1.26E-05	0.000117	10
11259_at	11259	ENSG0000(	FILIP1L	filamin A interacting pı		I	55	1.541917	1.826883	1.951447	2.005951	2.083064	4.950873	159.0223	2.26E-07	8.07E-06	10
11230_at	11230	ENSG0000(	PRAF2	PRA1 domain family, n		I	11	1.578524	1.872257	1.875795	2.047192	1.980291	5.012094	243.9273	4.69E-08	3.39E-06	10
6782_at	6782	ENSG0000(	HSPA13	heat shock protein 70k		I	44	1.493854	1.563728	1.922655	2.018225	2.048885	6.683033	153.649	2.57E-07	8.63E-06	10
10157_at	10157	ENSG0000(	AASS	aminoadipate-semiald		I	40	1.561085	1.809916	1.893716	1.772852	2.081131	5.663735	245.9478	4.55E-08	3.34E-06	10
10614_at	10614	ENSG0000(	HEXIM1	hexamethylene bis-ace		I	53	1.473719	1.670829	1.831574	1.814013	2.103036	7.564332	260.7122	3.67E-08	2.93E-06	10
5074_at	5074	ENSG0000(	PAWR	PRKC, apoptosis, WT1,		I	10	1.517651	1.79443	2.020929	1.948237	2.243536	8.289544	236.8517	5.23E-08	3.68E-06	10
214_at	214	ENSG0000(	ALCAM	activated lε	Secreted	I	76	1.758704	1.693354	1.897466	1.748331	2.237996	7.123149	173.4839	1.64E-07	6.81E-06	10
51804_at	51804	ENSG0000(	SIX4	SIX homeobox 4		I	11	1.760758	1.73684	1.955513	2.03453	2.684085	7.155414	235.5032	5.34E-08	3.72E-06	10
4131_at	4131	ENSG0000(	MAP1B	microtubule-associate		I	54	1.789465	1.948316	1.661386	1.854657	2.443306	8.000476	282.2454	2.74E-08	2.43E-06	10
1687_at	1687	ENSG0000(	DFNA5	deafness, autosomal d		I	11	1.349678	1.669974	1.842069	1.763228	1.962558	7.596715	155.1898	2.48E-07	8.41E-06	10
11329_at	11329	ENSG0000(	STK38	serine/thre	kinase	I	11	1.350949	1.595935	1.802561	1.750132	1.947767	9.412633	103.0195	1.11E-06	2.22E-05	10
182_at	182	ENSG0000(	JAG1	jagged 1		I	64	1.255509	1.662511	1.830476	1.804908	1.946888	5.747831	175.63	1.57E-07	6.65E-06	10
6495_at	6495	ENSG0000(	SIX1	SIX homeobox 1		I	33	1.135756	1.622115	1.89485	1.685943	2.104002	6.295378	156.922	2.38E-07	8.18E-06	10
84962_at	84962	ENSG0000(	JUB	jub, ajuba homolog (Xε		I	33	1.28849	1.964855	1.931223	1.956085	2.002734	7.736347	196.3953	1.04E-07	5.29E-06	10
51088_at	51088	ENSG0000(	KLHL5	kelch-like 5 (Drosophil		I	26	1.380609	1.769793	1.91443	1.940904	2.058743	6.695932	169.2535	1.80E-07	7.08E-06	10
9510_at	9510	ENSG0000(	ADAMTS1	ADAM met	peptidase	I	22	1.11192	1.69826	1.600553	2.002279	2.067562	7.100321	20.46548	0.000344	0.0015	10
10252_at	10252	ENSG0000(	SPRY1	sprouty homolog 1, an		I	33	0.767234	1.5419	1.726672	2.061413	2.204564	6.670013	172.4015	1.68E-07	6.87E-06	10
85453_at	85453	ENSG0000(	TSPYL5	TSPY-like 5		I	11	0.992471	1.440161	1.981593	2.298762	2.13577	4.405606	346.0467	1.29E-08	1.73E-06	10
7056_at	7056	ENSG0000(	THBD	thrombomodulin		I	22	1.249407	2.091246	1.953078	2.391508	2.006792	5.302502	77.34165	3.15E-06	4.41E-05	10
8900_at	8900	ENSG0000(	CCNA1	cyclin A1		I	9	1.071365	2.031782	1.911876	2.111131	1.875847	4.952249	84.05543	2.33E-06	3.63E-05	10
3601_at	3601	ENSG0000(	IL15RA	interleukin	Secreted	I	27	1.811796	1.897713	2.068878	1.909493	1.679157	5.341852	165.2948	1.96E-07	7.44E-06	10
7464_at	7464	ENSG0000(	CORO2A	coronin, actin binding		I	42	1.863561	1.859731	1.924203	1.837951	1.693204	4.859317	114.8876	7.45E-07	1.72E-05	10
23327_at	23327	ENSG0000(	NEDD4L	neural precursor cell e		I	70	1.67734	1.811216	1.994229	1.843886	1.749876	7.873722	59.47733	8.13E-06	8.48E-05	10
344558_at	344558	ENSG0000(	SH3RF3	SH3 domain containing		I	11	1.617324	1.950518	1.979373	1.844327	1.551151	4.550454	177.0766	1.53E-07	6.53E-06	10
64283_at	64283	ENSG0000(	RGNEF	190 kDa guanine nucle		I	45	1.733322	2.000526	1.806848	1.98973	1.753363	6.781911	152.2458	2.66E-07	8.79E-06	10
2250_at	2250	ENSG0000(	FGF5	fibroblast ¿	Secreted	I	66	1.724054	1.91896	1.840364	1.868198	1.843287	4.433799	168.6574	1.82E-07	7.13E-06	10
399474_at	399474	ENSG0000(	TMEM200I	transmembrane protei		I	11	1.647213	2.210576	1.965605	1.785474	1.830309	6.36117	415.2341	6.57E-09	1.25E-06	10
55809_at	55809	ENSG0000(	TRERF1	transcriptional regulati		I	33	1.723955	2.014671	1.849167	1.667713	1.898885	6.599497	335.2018	1.45E-08	1.76E-06	10
9536_at	9536	ENSG0000(	PTGES	prostaglandin E syntha		I	32	1.723431	2.142566	1.940541	2.134993	1.870658	6.316672	194.581	1.08E-07	5.35E-06	10
54749_at	54749	ENSG0000(	EPDR1	ependymir	Secreted	I	32	1.793545	2.101174	2.052277	2.025924	1.834084	5.090016	409.329	6.92E-09	1.26E-06	10
56034_at	56034	ENSG0000(	PDGFC	platelet de	Secreted	I	30	1.744734	2.112732	1.964986	1.979375	1.688916	7.101835	338.539	1.40E-08	1.76E-06	10
285704_at	285704	ENSG0000(	RGMB	RGM domain family, m		I	22	1.897623	2.199917	2.165354	1.84331	1.887717	8.194036	283.6681	2.69E-08	2.41E-06	10
4329_at	4329	ENSG0000(	ALDH6A1	aldehyde dehydrogena		I	11	1.748744	2.230302	2.090462	1.604432	2.019347	5.953552	156.0421	2.43E-07	8.30E-06	10
9957_at	9957	ENSG0000(	HS3ST1	heparan sulfate (gluco		I	20	2.229189	1.929981	1.796703	1.653829	1.598148	4.850414	60.36152	7.71E-06	8.17E-05	10
55286_at	55286	ENSG0000(	C4orf19	chromosome 4 open re		I	33	2.147056	2.056656	1.809425	1.609034	1.513022	5.177363	115.9489	7.21E-07	1.68E-05	10
688_at	688	ENSG0000(	KLF5	Kruppel-like factor 5 (i		I	31	1.961287	1.670896	1.857254	1.678146	1.84673	4.665585	213.1705	7.71E-08	4.53E-06	10
3231_at	3231	ENSG0000(	HOXD1	homeobox D1		I	11	1.908791	2.357788	1.452497	2.209229	2.235808	5.204096	189.4939	1.19E-07	5.63E-06	10
5307_at	5307	ENSG0000(	PITX1	paired-like homeodom		I	22	1.958163	2.159089	1.481668	1.873497	1.826476	5.94992	69.02728	4.75E-06	5.83E-05	10
339512_at	339512	ENSG0000(	C1orf110	chromosome 1 open re		I	11	1.836334	2.199732	0.990916	1.745918	2.171532	4.972488	46.98705	1.89E-05	0.000158	10
147463_at	147463	ENSG0000(	ANKRD29	ankyrin repeat domain		I	11	1.819513	1.911274	2.232446	1.923469	1.981098	5.562294	185.6924	1.28E-07	5.89E-06	10
23586_at	23586	ENSG0000(	DDX58	DEAD (Asp-Glu-Ala-As		I	32	1.975936	1.769044	2.166517	1.829012	1.84941	7.098393	112.215	8.12E-07	1.82E-05	10
10267_at	10267	ENSG0000(	RAMP1	receptor (G	transporter	I	11	2.010109	1.457667	2.345759	1.944572	1.891767	5.492976	56.518	9.77E-06	9.73E-05	10
57458_at	57458	ENSG0000(	TMCC3	transmembrane and cc		I	33	1.775601	1.587415	2.252847	1.777686	2.168485	5.551981	303.6312	2.09E-08	2.10E-06	10
404636_at	404636	ENSG0000(	FAM45A	family with sequence s		I	3	1.709322	1.696785	2.581912	1.975792	1.578602	6.936862	143.4048	3.31E-07	1.00E-05	10
1843_at	1843	ENSG0000(	DUSP1	dual specif	phosphopr	I	38	1.745808	1.683677	2.324698	1.84635	1.314316	5.119435	39.98674	3.36E-05	0.000244	10
1289_at	1289	ENSG0000(	COL5A1	collagen, ty	Secreted	I	44	1.674488	1.950694	2.677333	1.567386	1.797173	5.236943	230.5522	5.77E-08	3.86E-06	10
4907_at	4907	ENSG0000(	NT5E	5'-nucleoti	Secreted	I	30	1.428498	1.453669	2.547787	1.429751	1.629459	8.30113	200.9422	9.58E-08	5.15E-06	10
340542_at	340542	ENSG0000(	BEX5	brain expressed, X-link		I	11	1.41486	2.077742	2.737143	2.17486	2.090815	4.701033	161.2525	2.15E-07	7.90E-06	10
1545_at	1545	ENSG0000(	CYP1B1	cytochrome P450, fam		I	55	1.373603	1.627734	2.483296	2.360557	2.200571	4.941039	29.26205	0.000101	0.000568	10
25974_at	25974	ENSG0000(	MMACHC	methylmalonic aciduri		I	11	1.368521	1.956007	2.410542	1.97923	1.851435	4.978519	247.4247	4.45E-08	3.30E-06	10
10769_at	10769	ENSG0000(	PLK2	polo-like ki	kinase	I	42	1.354196	1.991952	2.140418	2.05182	2.002775	6.288399	65.48404	5.75E-06	6.69E-05	10
1363_at	1363	ENSG0000(	CPE	carboxypep	peptidase	I	19	1.920685	1.342159	2.616123	2.148572	2.505036	4.507778	254.0317	4.04E-08	3.10E-06	10
80823_at	80823	ENSG0000(	BHLHB9	basic helix-loop-helix c		I	7	1.783422	1.03418	2.312966	1.916564	2.028988	4.882311	310.2985	1.93E-08	1.99E-06	10

1645_at	1645	ENSG0000(	AKR1C1	aldo-keto reductase fa		I	22	1.463415	0.724386	2.807692	1.987196	2.327769	5.599958	177.7881	1.50E-07	6.45E-06	10
4072_at	4072	ENSG0000(	EPCAM	epithelial cell adhesior		I	28	1.287291	1.417615	2.531916	1.687855	2.158347	4.368708	137.0118	3.91E-07	1.12E-05	10
9603_at	9603	ENSG0000(	NFE2L3	nuclear fac	Secreted	I	12	1.27635	1.266809	2.407089	1.874425	2.024383	6.83419	246.5077	4.51E-08	3.34E-06	10
5920_at	5920	ENSG0000(	RARRES3	retinoic acid receptor r		I	22	1.517701	1.424327	2.628807	1.773292	1.887597	6.291219	64.99178	5.91E-06	6.84E-05	10
1839_at	1839	ENSG0000(	HBEGF	heparin-bi	Secreted	I	21	2.128572	2.317931	2.93646	2.271769	2.352652	5.799469	578.9511	1.92E-09	9.95E-07	10
84419_at	84419	ENSG0000(	C15orf48	chromosor	Secreted	I	7	1.969381	2.261396	2.942491	2.62209	2.025713	6.049874	69.93631	4.53E-06	5.64E-05	10
8743_at	8743	ENSG0000(	TNFSF10	tumor necrosis factor (		I	33	1.885662	1.88458	3.217073	2.289913	2.504551	6.177248	200.0413	9.74E-08	5.17E-06	10
5997_at	5997	ENSG0000(	RGS2	regulator of G-protein		I	6	1.66397	2.216503	3.123382	3.125769	1.992393	6.089054	178.3735	1.48E-07	6.41E-06	10
5205_at	5205	ENSG0000(	ATP8B1	ATPase, an	transporte	I	22	1.883414	2.23712	2.713694	2.515601	2.806252	4.948599	223.312	6.49E-08	4.11E-06	10
79710_at	79710	ENSG0000(	MORC4	MORC family CW-type		I	55	1.934632	2.013933	2.695819	2.505455	2.66774	7.115609	139.0075	3.71E-07	1.07E-05	10
10974_at	10974	ENSG0000(	C10orf116	chromosome 10 open		I	20	1.803165	2.142428	2.640043	2.340547	2.502325	7.22484	165.9484	1.94E-07	7.40E-06	10
9411_at	9411	ENSG0000(	ARHGAP29	Rho GTPase activating		I	55	1.744485	2.219549	2.542097	2.575874	3.038989	7.16233	315.6385	1.81E-08	1.93E-06	10
5366_at	5366	ENSG0000(	PMAIP1	phorbol-12-myristate-		I	22	1.631223	1.94254	2.475737	2.574501	2.954921	9.128215	225.3113	6.28E-08	4.03E-06	10
10461_at	10461	ENSG0000(	MERTK	c-mer prot	kinase	I	11	1.55774	1.835542	1.817103	2.699127	2.700707	4.888868	333.1773	1.48E-08	1.76E-06	10
22998_at	22998	ENSG0000(	LIMCH1	LIM and calponin hom		I	91	1.413359	1.887782	1.820074	2.400153	2.68328	7.261604	56.08763	1.00E-05	9.92E-05	10
1490_at	1490	ENSG0000(	CTGF	connective	Secreted	I	32	1.703094	2.273717	2.335935	2.989737	3.376233	10.68539	97.36288	1.36E-06	2.50E-05	10
5052_at	5052	ENSG0000(	PRDX1	peroxiredo	Secreted	I	5	1.576191	2.465339	2.563583	2.948934	3.105367	11.41657	173.9359	1.63E-07	6.81E-06	10
10371_at	10371	ENSG0000(	SEMA3A	sema dom	Secreted	I	22	1.496562	1.876511	2.466825	2.611614	3.560283	7.011568	56.50603	9.78E-06	9.73E-05	10
306_at	306	ENSG0000(	ANXA3	annexin A3		I	19	1.08368	1.391415	2.191808	2.199731	3.347699	9.295219	104.7349	1.05E-06	2.13E-05	10
51655_at	51655	ENSG0000(	RASD1	RAS, dexan	GTPase	I	11	0.647403	1.434457	2.296731	2.398807	2.940051	4.863088	306.0047	2.03E-08	2.06E-06	10
8673_at	8673	ENSG0000(	VAMP8	vesicle-associated mer		I	9	1.959468	2.65894	2.704326	3.10107	3.110434	10.8641	200.6029	9.64E-08	5.16E-06	9
10551_at	10551	ENSG0000(	AGR2	anterior gr	Secreted	I	14	1.871778	2.673402	2.765025	3.082451	3.082488	7.753647	29.08561	0.000103	0.000578	9
84617_at	84617	ENSG0000(	TUBB6	tubulin, be	GTPase	I	22	1.9392	2.714273	2.671188	3.075373	3.241518	10.3777	403.0241	7.33E-09	1.31E-06	9
284293_at	284293	ENSG0000(	HMSD	histocompatibility (mir		I	9	2.019779	2.823373	2.677102	2.826367	3.132941	5.32202	233.3139	5.52E-08	3.80E-06	9
3880_at	3880	ENSG0000(	KRT19	keratin 19		I	7	2.232891	2.903116	2.671721	3.199351	3.093262	11.19089	521.196	2.83E-09	1.03E-06	9
5328_at	5328	ENSG0000(	PLAU	plasminog	kinase	I	31	2.33416	2.76602	2.798384	3.106286	3.031368	8.208259	413.5626	6.67E-09	1.25E-06	9
83641_at	83641	ENSG0000(	FAM107B	family with sequence s		I	49	2.359227	2.780844	2.724611	2.804412	3.026799	6.392804	191.9667	1.13E-07	5.51E-06	9
3087_at	3087	ENSG0000(	HHEX	hematopoietically expr		I	11	2.249009	2.991225	2.826884	2.731803	2.783857	4.984548	346.9979	1.28E-08	1.73E-06	9
124220_at	124220	ENSG0000(	ZG16B	zymogen g	Secreted	I	11	1.927346	2.695939	2.886698	2.977584	2.720656	6.185076	725.4468	8.31E-10	9.95E-07	9
1514_at	1514	ENSG0000(	CTSL1	cathepsin L	peptidase	I	10	2.219948	2.910331	3.026806	3.289818	2.961653	7.782147	341.0806	1.36E-08	1.75E-06	9
100131187	1E+08	ENSG0000(	TSTD1	thiosulfate sulfurtrans		I	11	2.243706	2.943621	3.010931	3.123121	2.886928	5.992403	735.9071	7.88E-10	9.95E-07	9
2920_at	2920	ENSG0000(	CXCL2	chemokine	Secreted	I	21	2.038225	2.784281	3.095815	3.179219	3.143548	7.127174	18.53954	0.00048	0.001973	9
2122_at	2122	ENSG0000(	MECOM	MDS1 and EVI1 comple		I	28	2.420888	2.755596	3.249392	2.993391	3.037757	6.18666	251.5393	4.19E-08	3.17E-06	9
6662_at	6662	ENSG0000(	SOX9	SRY (sex determining r		I	33	2.272155	2.802511	3.165385	3.016469	3.189607	6.163484	207.2136	8.55E-08	4.79E-06	9
3606_at	3606	ENSG0000(	IL18	interleukin	Secreted	I	10	2.039517	3.072851	3.275489	2.989142	3.213378	6.135723	360.3116	1.11E-08	1.67E-06	9
203413_at	203413	ENSG0000(	CXorf61	chromosome X open r		I	10	2.031887	3.100698	2.888079	2.973345	2.999018	5.549939	333.4725	1.48E-08	1.76E-06	9
64866_at	64866	ENSG0000(	CDCP1	CUB domai	Secreted	I	32	2.500427	2.594892	2.865904	3.250129	3.512214	6.649106	552.6652	2.28E-09	1.03E-06	9
217_at	217	ENSG0000(	ALDH2	aldehyde dehydrogena		I	15	2.281711	2.620351	2.850275	3.422208	3.419302	6.826413	407.9406	7.01E-09	1.26E-06	9
253152_at	253152	ENSG0000(	EPHX4	epoxide hydrolase 4		I	10	2.24743	2.768181	2.957911	2.972619	3.462597	5.718639	389.9828	8.28E-09	1.44E-06	9
4070_at	4070	ENSG0000(	TACSTD2	tumor-associated calci		I	19	2.076051	2.826632	2.815299	3.095871	3.479457	8.328051	127.5444	5.09E-07	1.34E-05	9
8644_at	8644	ENSG0000(	AKR1C3	aldo-keto reductase fa		I	9	2.315877	2.419975	3.141757	3.273928	3.390575	8.706746	842.3231	4.78E-10	9.95E-07	9
1469_at	1469	ENSG0000(	CST1	cystatin SN	Secreted	I	20	2.156623	2.143122	2.984272	3.170059	3.33834	9.751439	601.8466	1.66E-09	9.95E-07	9
3730_at	3730	ENSG0000(	KAL1	Kallmann s	Secreted	I	30	2.350735	2.394065	3.027764	2.809003	3.071217	6.735724	432.1343	5.67E-09	1.23E-06	9
115207_at	115207	ENSG0000(	KCTD12	potassium	ion channe	I	55	2.1202	2.165591	3.06315	2.604637	3.116694	5.79001	358.7912	1.81E-08	1.93E-06	9
7980_at	7980	ENSG0000(	TFPI2	tissue factc	Secreted	I	21	1.861525	2.375537	3.009077	2.721512	3.355601	6.744314	71.12236	4.27E-06	5.41E-05	9
2318_at	2318	ENSG0000(	FLNC	filamin C, gamma		I	10	2.775759	3.026529	2.063488	3.230394	3.413934	6.745669	277.5584	2.91E-08	2.53E-06	9
8436_at	8436	ENSG0000(	SDPR	serum deprivation resp		I	22	2.373032	3.161217	2.372033	3.010516	3.4753	6.066997	300.7626	2.16E-08	2.13E-06	9
3625_at	3625	ENSG0000(	INHBB	inhibin, be	Secreted	I	11	2.820166	3.048747	2.644456	3.518882	3.629602	6.143265	144.1634	3.25E-07	9.92E-06	9
7325_at	7325	ENSG0000(	UBE2E2	ubiquitin-conjugating (		I	11	2.32044	2.966303	3.029567	3.337066	3.635105	6.731678	250.4059	4.26E-08	3.21E-06	9
10653_at	10653	ENSG0000(	SPINT2	serine peptidase inhibi		I	22	2.23154	2.968955	2.992759	3.417065	3.567836	9.443787	491.0139	3.53E-09	1.06E-06	9
5646_at	5646	ENSG0000(	PRSS3	protease, s	peptidase	I	11	2.349103	2.979605	2.953122	3.526875	3.730371	6.288171	374.1038	9.66E-09	1.57E-06	9
5167_at	5167	ENSG0000(	ENPP1	ectonucleo	Secreted	I	32	2.252631	2.725484	3.044513	3.380536	3.63703	6.952322	330.875	1.52E-08	1.80E-06	9
54863_at	54863	ENSG0000(	C9orf167	chromosome 9 open r		I	10	2.640522	2.932811	3.103855	3.317482	3.565167	5.755483	98.36714	1.31E-06	2.43E-05	9
23266_at	23266	ENSG0000(	LPHN2	latrophilin	G-protein c	I	22	2.675378	2.773882	3.216225	3.23077	3.598772	5.744977	243.075	4.75E-08	3.42E-06	9
50486_at	50486	ENSG0000(	G0S2	G0/G1swit	Secreted	I	25	2.533425	3.258073	3.250068	3.552155	3.708109	8.731754	276.4286	2.96E-08	2.54E-06	9
1906_at	1906	ENSG0000(	EDN1	endothelin	Secreted	I	22	2.509929	3.419125	3.240826	3.412959	3.673648	7.280589	123.545	5.71E-07	1.45E-05	9
1535_at	1535	ENSG0000(	CYBA	cytochrome b-245, alp		I	11	2.433523	3.374382	3.004344	3.426816	3.573764	8.28901	232.0691	5.63E-08	3.83E-06	9
7837_at	7837	ENSG0000(	PXDN	peroxidasin homolog (		I	22	2.520559	3.222238	3.00559	3.644309	3.897408	9.391944	318.5342	1.75E-08	1.92E-06	9
2150_at	2150	ENSG0000(	F2RL1	coagulatior	G-protein c	I	16	2.522752	3.135287	3.07989	3.576879	3.633118	6.615476	504.0809	3.20E-09	1.03E-06	9
3484_at	3484	ENSG0000(	IGFBP1	insulin-like	Secreted	I	11	2.154399	2.689258	3.510275	3.405638	3.952974	9.099388	54.50748	1.11E-05	0.000107	9
137835_at	137835	ENSG0000(	TMEM71	transmembrane protei		I	11	2.232369	2.608189	3.283936	3.61987	3.965887	6.104667	710.3593	8.98E-10	9.95E-07	9
221395_at	221395	ENSG0000(	GPR116	G protein-c	G-protein c	I	27	2.505618	2.753177	3.236779	3.659387	4.4453	8.224266	169.7352	1.78E-07	7.03E-06	9
84707_at	84707	ENSG0000(	BEX2	brain expressed X-link		I	11	2.07514	2.882413	3.517019	3.068896	3.566491	7.304559	49.76258	1.54E-05	0.000136	9
9823_at	9823	ENSG0000(	ARMCX2	armadillo repeat conta		I	10	2.134152	2.553876	3.216452	3.078417	3.763889	7.657666	271.2938	3.17E-08	2.63E-06	9
5806_at	5806	ENSG0000(	PTX3	pentraxin 3	Secreted	I	11	1.416479	2.716985	3.207149	3.532305	3.646293	9.126458	97.12617	1.38E-06	2.51E-05	9
54102_at	54102	ENSG0000(	CLIC6	chloride in	ion channe	I	11	2.569198	2.904606	3.611757	3.368288	3.203175	5.436916	127.0277	5.16E-07	1.35E-05	9
54504_at	54504	ENSG0000(	CPVL	carboxype	peptidase	I	11	2.660533	2.892547	3.271163	3.420783	3.103695	6.421536	172.794	1.67E-07	6.84E-06	9
196792_at	196792	ENSG0000(	FAM24B	family with	Secreted	I	8	2.471226	2.819146	3.250468	3.413385	3.263706	7.256153	367.4497	1.03E-08	1.60E-06	9
1047_at	1047	ENSG0000(	CLGN	calmegin		I	54	2.376717	2.829097	3.272527	3.270317	3.333219	5.865271	288.1625	2.53E-08	2.34E-06	9
10874_at	10874	ENSG0000(	NMU	neuromedi	Secreted	I	15	2.339658	3.092183	3.236797	3.439795	3.521258	5.998443	182.0387	1.38E-07	6.18E-06	9
2591_at	2591	ENSG0000(	GALNT3	UDP-N-acetyl-alpha-D-		I	22	2.250113	3.124057	3.139743	3.454673	3.288181	5.643632	309.862	1.94E-08	1.99E-06	9
64073_at	64073	ENSG0000(	C19orf33	chromosome 19 open		I	9	2.400687	3.236283	3.392545	3.628934	3.379336	9.390285	384.872	8.70E-09	1.48E-06	9
254427_at	254427	ENSG0000(	C10orf47	chromosome 10 open		I	22	2.505587	3.257323	3.302083	3.131693	3.31715	6.93266	312.9992	1.87E-08	1.98E-06	9
84230_at	84230	ENSG0000(	LRRC8C	leucine rich repeat con		I	22	2.557141	3.353106	3.162779	2.941191	3.445728	5.792353	383.0041	8.85E-09	1.49E-06	9
6935_at	6935	ENSG0000(	ZEB1	zinc finger E-box bindi		I	77	2.476115	3.102582	2.977252	3.195031	3.425384	6.187461	591.7904	1.77E-09	9.95E-07	9
7103_at	7103	ENSG0000(	TSPAN8	tetraspanin 8		I	10	2.869989	3.538276	3.314426	3.393581	3.106629	6.015158	498.6233	3.33E-09	1.03E-06	9
54518_at	54518	ENSG0000(	APBB1IP	amyloid beta (A4) prec		I	11	3.021877	3.277972	3.247412	3.393592	3.247516	7.210238	77.37997	3.14E-06	4.41E-05	9
440498_at	440498	ENSG0000(	HSBP1L1	heat shock factor bindi		I	11	2.838058	3.431113	3.399218	3.679757	3.261591	6.52621	255.6887	3.94E-08	3.05E-06	9
7570_at	7570	ENSG0000(	ZNF22	zinc finger protein 22 (		I	33	2.708518	3.31181	3.077313	3.448253	3.448345	7.29333	514.2799	2.97E-09	1.03E-06	9
3856_at	3856	ENSG0000(	KRT8	keratin 8		I	30	2.83865	3.182349	2.997543	3.349597	3.319813	6.83629	390.4888	8.24E-09	1.44E-06	9
10098_at	10098	ENSG0000(	TSPAN5	tetraspanin 5		I	19	3.042606	3.20722	2.9744	2.746583	2.9702	6.460198	425.6879	5.99E-09	1.23E-06	9
978_at	978	ENSG0000(	CDA	cytidine deaminase		I	18	2.783751	3.296927	2.985244	2.876734	2.70062	7.064345	413.0357	6.70E-09	1.25E-06	9

10053_at	10053	ENSG0000(	AP1M2	adaptor-related protei	I	7	3.052396	3.382334	3.079545	2.978076	3.26036	5.751808	627.9871	1.42E-09	9.95E-07	9
4688_at	4688	ENSG0000(	NCF2	neutrophil cytosolic fa	I	11	3.058543	2.882183	3.169403	3.093625	2.766256	5.354091	208.6023	8.35E-08	4.75E-06	9
56938_at	56938	ENSG0000(	ARNTL2	aryl hydrocarbon recep	I	11	2.684395	2.720946	3.149093	3.025471	2.917807	5.183059	156.8854	2.38E-07	8.18E-06	9
55228_at	55228	ENSG0000(	PNMAL1	PNMA-like Secreted	I	11	2.782727	3.495684	3.673978	3.593309	3.478044	6.575711	603.2322	1.65E-09	9.95E-07	9
79746_at	79746	ENSG0000(	ECHDC3	enoyl CoA hydratase d	I	11	2.847387	3.356432	3.545718	3.580035	3.392382	7.141538	274.9518	3.01E-08	2.57E-06	9
26499_at	26499	ENSG0000(	PLEK2	pleckstrin 2	I	11	2.565974	3.407024	3.654112	3.546106	3.426505	7.249385	497.4858	3.36E-09	1.03E-06	9
56925_at	56925	ENSG0000(	LXN	latexin	I	20	2.375801	3.520952	3.779015	3.525839	3.175714	5.814048	424.4966	6.05E-09	1.23E-06	9
28234_at	28234	NA	SLCO1B3	solute carri transporter	I	9	2.72309	3.014857	4.13671	3.489151	3.219278	6.053568	339.8025	1.38E-08	1.75E-06	9
5867_at	5867	ENSG0000(	RAB4A	RAB4A, me GTPase	I	11	2.506062	3.238536	3.550832	3.739659	4.167241	6.991444	206.8629	8.61E-08	4.80E-06	9
1832_at	1832	ENSG0000(	DSP	desmoplakin	I	22	2.383509	3.217644	3.485353	3.721763	4.084318	7.606345	219.8117	6.88E-08	4.27E-06	9
94234_at	94234	ENSG0000(	FOXQ1	forkhead box Q1	I	22	2.50487	3.401061	3.353199	3.867385	4.183252	7.340278	295.4671	2.31E-08	2.20E-06	9
5654_at	5654	ENSG0000(	HTRA1	HtrA serine peptidase	I	21	2.496287	3.140725	3.360894	3.649195	4.011483	7.231121	442.1103	5.21E-09	1.23E-06	9
28984_at	28984	ENSG0000(	C13orf15	chromosome 13 open	I	11	2.510907	3.065773	3.168969	3.71567	3.950423	7.368611	129.0407	4.87E-07	1.30E-05	9
170712_at	170712	ENSG0000(	COX7B2	cytochrom transporter	I	11	2.580559	3.437197	3.192105	3.728804	3.921506	8.257798	441.2193	5.25E-09	1.23E-06	9
143903_at	143903	ENSG0000(	LAYN	layilin	I	22	2.523124	3.411567	3.339105	3.599146	3.869305	6.874855	160.6073	2.18E-07	7.98E-06	9
2202_at	2202	ENSG0000(	EFEMP1	EGF contai Secreted	I	28	2.540309	3.475062	3.321888	3.847915	3.795568	7.830021	823.3675	5.20E-10	9.95E-07	9
27063_at	27063	ENSG0000(	ANKRD1	ankyrin repeat domain	I	11	2.870781	3.602656	2.989719	3.747122	4.581503	9.184807	77.81907	3.08E-06	4.35E-05	9
80008_at	80008	ENSG0000(	TMEM156	transmembrane protei	I	11	2.851389	3.424246	3.795771	3.580815	3.935139	6.828588	145.3507	3.15E-07	9.80E-06	9
347744_at	347744	ENSG0000(	C6orf52	chromosome 6 open re	I	11	2.812828	3.359404	3.666663	3.600372	3.96973	6.472882	1021.224	2.34E-10	9.95E-07	9
201161_at	201161	ENSG0000(	CENPV	centromere protein V	I	8	3.183474	3.633103	3.532453	3.220954	3.712922	6.584946	324.0945	1.64E-08	1.86E-06	9
7852_at	7852	ENSG0000(	CXCR4	chemokine G-protein c	I	44	2.534862	3.708487	3.672144	3.696025	3.758449	7.204409	439.8321	5.31E-09	1.23E-06	9
725_at	725	ENSG0000(	C4BPB	complemei Secreted	I	9	2.748001	3.791249	3.542737	3.743312	3.356686	6.743411	419.8588	6.30E-09	1.24E-06	9
2203_at	2203	ENSG0000(	FBP1	fructose-1,6-bisphospl	I	8	2.636205	4.143734	3.372977	3.878106	3.594201	7.397449	469.7131	4.16E-09	1.10E-06	9
51422_at	51422	ENSG0000(	PRKAG2	protein kinase, AMP-a	I	43	1.39829	1.487847	1.562301	1.349022	1.478608	7.219796	99.94471	1.24E-06	2.36E-05	8
29103_at	29103	ENSG0000(	DNAJC15	DnaJ (Hsp40) homolog	I	19	1.409396	1.491206	1.518195	1.257252	1.281465	7.347124	99.63314	1.25E-06	2.37E-05	8
81848_at	81848	ENSG0000(	SPRY4	sprouty homolog 4 (Dr	I	22	1.185742	1.430553	1.571862	1.361643	1.273368	5.363039	123.6696	5.69E-07	1.45E-05	8
7099_at	7099	ENSG0000(	TLR4	toll-like receptor 4	I	33	1.190265	1.498071	1.519107	1.416026	1.192228	6.111875	77.95578	3.06E-06	4.33E-05	8
89927_at	89927	ENSG0000(	C16orf45	chromosome 16 open	I	22	1.093803	1.451785	1.538792	1.280513	1.349367	4.378716	83.87179	2.35E-06	3.63E-05	8
132720_at	132720	ENSG0000(	C4orf32	chromosome 4 open re	I	11	1.089308	1.434307	1.508557	1.213101	1.334528	6.375249	57.71467	9.06E-06	9.21E-05	8
283459_at	283459	ENSG0000(	GATC	glutamyl-tRNA(Gln) an	I	7	1.153825	1.455662	1.532741	1.134421	1.435887	4.554789	32.7325	6.81E-05	0.000417	8
8321_at	8321	ENSG0000(	FZD1	frizzled fan G-protein c	I	22	1.258199	1.552125	1.46016	1.390526	1.59202	5.028443	158.2897	2.30E-07	8.08E-06	8
4331_at	4331	ENSG0000(	MNAT1	menage a trois homolc	I	11	1.104973	1.480451	1.467639	1.438951	1.478238	8.030237	167.1102	1.89E-07	7.28E-06	8
22824_at	22824	ENSG0000(	HSPA4L	heat shock 70kDa prot	I	33	1.306696	1.206624	1.705174	1.428221	1.4384	6.299606	130.6702	4.65E-07	1.26E-05	8
4048_at	4048	ENSG0000(	LTA4H	leukotriene peptidase	I	10	1.228387	1.221361	1.494773	1.506909	1.448454	9.69464	233.5073	5.51E-08	3.80E-06	8
266812_at	266812	ENSG0000(	NAP1L5	nucleosome assembly	I	33	1.26665	1.330604	1.519028	1.359234	1.467507	3.945699	126.4364	5.25E-07	1.36E-05	8
259230_at	259230	ENSG0000(	SGMS1	sphingomy kinase	I	22	1.332144	1.238296	1.524333	1.273908	1.417603	7.210862	96.8051	1.39E-06	2.54E-05	8
50804_at	50804	ENSG0000(	MYEF2	myelin expression fact	I	55	1.441604	1.278049	1.453366	1.545408	1.322245	4.245214	89.30469	1.87E-06	3.07E-05	8
81603_at	81603	ENSG0000(	TRIM8	tripartite motif contair	I	30	1.076263	1.365557	1.611241	1.498431	1.379459	8.048435	165.8413	1.94E-07	7.40E-06	8
558_at	558	ENSG0000(	AXL	AXL recept kinase	I	22	0.991457	1.322468	1.586467	1.508462	1.34022	9.357245	199.0072	9.93E-08	5.18E-06	8
84138_at	84138	ENSG0000(	SLC7A6OS	solute carrier family 7,	I	11	0.952738	1.281705	1.537224	1.600577	1.355738	5.55701	120.9022	6.19E-07	1.52E-05	8
84909_at	84909	ENSG0000(	C9orf3	chromosor peptidase	I	40	0.819531	1.506234	1.41442	1.465062	1.434177	5.755692	120.3004	6.30E-07	1.53E-05	8
441150_at	441150	ENSG0000(	C6orf226	chromosome 6 open re	I	11	1.047061	1.41958	1.460272	1.629707	1.316205	7.543093	69.47046	4.65E-06	5.73E-05	8
2745_at	2745	ENSG0000(	GLRX	glutaredoxin (thioltran	I	14	1.044019	1.354784	1.341298	1.699131	1.27528	8.471149	69.44375	4.65E-06	5.74E-05	8
9878_at	9878	ENSG0000(	TOX4	TOX high mobility grou	I	30	1.20212	1.501029	1.305788	1.533133	1.30841	5.670705	160.9228	2.17E-07	7.94E-06	8
56951_at	56951	ENSG0000(	C5orf15	chromosome 5 open re	I	17	0.994647	1.35713	1.448946	1.651878	1.524206	7.570902	165.7607	1.94E-07	7.40E-06	8
9891_at	9891	ENSG0000(	NUAK1	NUAK fami kinase	I	33	1.019665	1.22874	1.409341	1.51988	1.551649	6.257421	100.012	1.24E-06	2.36E-05	8
144165_at	144165	ENSG0000(	PRICKLE1	prickle hon Secreted	I	33	0.962182	1.394498	1.178748	1.543904	1.581859	5.252922	76.30422	3.31E-06	4.57E-05	8
144455_at	144455	ENSG0000(	E2F7	E2F transcription facto	I	22	0.960404	1.425131	1.290552	1.433345	1.481538	6.202932	167.0602	1.89E-07	7.28E-06	8
122786_at	122786	ENSG0000(	FRMD6	FERM domain containi	I	22	0.997452	1.485538	1.3721	1.655838	1.709391	7.854773	73.23974	3.84E-06	5.03E-05	8
5813_at	5813	ENSG0000(	PURA	purine-rich element bi	I	31	1.080688	1.238482	1.169166	1.195451	1.47507	8.560925	146.916	3.03E-07	9.61E-06	8
3667_at	3667	ENSG0000(	IRS1	insulin receptor substr	I	33	0.997827	1.211777	1.184309	1.30546	1.444007	5.728465	147.1327	3.01E-07	9.58E-06	8
91851_at	91851	ENSG0000(	CHRDL1	chordin-lik Secreted	I	31	1.137717	1.203622	1.284971	1.250373	1.353665	4.065727	80.00735	2.79E-06	4.06E-05	8
30001_at	30001	ENSG0000(	ERO1L	ERO1-like (Secreted	I	19	1.046056	1.10185	1.190317	1.293093	1.351772	7.656357	157.2499	2.36E-07	8.17E-06	8
60485_at	60485	ENSG0000(	SAV1	salvador homolog 1 (D	I	31	1.135706	1.331897	1.236111	1.392812	1.401657	6.384215	81.79592	2.57E-06	3.84E-05	8
1032_at	1032	ENSG0000(	CDKN2D	cyclin-dependent kinas	I	10	0.970816	1.316741	1.33033	1.318975	1.351138	8.545129	115.2816	7.36E-07	1.70E-05	8
118987_at	118987	ENSG0000(	PDZD8	PDZ domai Secreted	I	11	1.11649	1.191106	1.25789	1.289082	1.58846	5.292471	100.0536	1.23E-06	2.36E-05	8
64398_at	64398	ENSG0000(	MPP5	membrane protein, pa	I	54	1.011429	1.218006	1.414806	1.264172	1.52629	7.354615	159.7035	2.23E-07	8.02E-06	8
221035_at	221035	ENSG0000(	REEP3	receptor accessory prc	I	31	0.810267	0.998942	1.296742	1.150579	1.538437	5.044754	120.5783	6.25E-07	1.52E-05	8
57325_at	57325	ENSG0000(	CSRP2BP	CSRP2 binding protein	NI	22	0.893171	1.05285	1.284706	1.019624	1.424715	5.848225	51.23897	1.39E-05	0.000125	8
4900_at	4900	ENSG0000(	NRGN	neurogranin (protein k	I	11	0.93328	1.281028	1.32909	1.044549	1.460922	4.2985	77.86687	3.07E-06	4.34E-05	8
10924_at	10924	ENSG0000(	SMPDL3A	sphingomyelin phosph	I	11	0.835128	1.216486	1.717066	1.330614	1.511463	6.554894	40.50353	3.21E-05	0.000236	8
26128_at	26128	ENSG0000(	KIAA1279	KIAA1279	I	22	0.825513	1.074513	1.672401	1.191567	1.212115	8.435087	162.8076	2.08E-07	7.71E-06	8
29950_at	29950	ENSG0000(	SERTAD1	SERTA domain contain	I	11	0.810846	1.266891	1.428213	1.264812	1.510016	7.329001	157.7047	2.33E-07	8.12E-06	8
9585_at	9585	ENSG0000(	KIF20B	kinesin family member	I	9	0.665299	1.336862	1.493229	1.137409	1.35131	5.39565	20.60001	0.000337	0.001474	8
166815_at	166815	ENSG0000(	TIGD2	tigger transposable ele	I	11	1.061468	1.150177	1.562311	1.342901	1.689728	6.991329	79.09625	2.90E-06	4.17E-05	8
64430_at	64430	ENSG0000(	C14orf135	chromosome 14 open	I	33	1.149986	1.081507	1.495415	1.295044	1.664548	8.774241	124.7598	5.51E-07	1.42E-05	8
55591_at	55591	ENSG0000(	VEZT	vezatin, adherens junc	I	41	0.944653	1.15564	1.483495	1.408336	1.647223	6.50643	100.9735	1.19E-06	2.30E-05	8
80318_at	80318	ENSG0000(	GKAP1	G kinase ar kinase	I	20	1.021688	1.380532	1.596205	1.214182	1.697163	5.344279	141.0565	3.52E-07	1.04E-05	8
128434_at	128434	ENSG0000(	VSTM2L	V-set and t Secreted	I	11	1.003545	1.258799	1.592999	1.291053	1.759604	5.461961	155.3447	2.47E-07	8.39E-06	8
23443_at	23443	ENSG0000(	SLC35A3	solute carri transporter	I	11	1.191057	1.443856	1.409144	1.475666	1.942756	5.717794	222.9119	6.54E-08	4.11E-06	8
84458_at	84458	ENSG0000(	LCOR	ligand dependent nucl	I	11	1.149291	1.322176	1.213179	1.39844	1.970759	7.437997	145.973	3.10E-07	9.71E-06	8
5728_at	5728	ENSG0000(	PTEN	phosphata: phosphopr	I	27	1.47188	1.326289	1.648755	1.686341	1.724816	6.798674	189.0747	1.20E-07	5.63E-06	8
2589_at	2589	ENSG0000(	GALNT1	UDP-N-acetyl-alpha-D-	I	40	1.454012	1.334236	1.530423	1.587001	1.748225	8.206308	256.1358	3.92E-08	3.05E-06	8
285527_at	285527	ENSG0000(	FRYL	FRY-like	I	33	1.457344	1.487082	1.4793	1.465659	1.686644	6.673439	117.4993	6.87E-07	1.62E-05	8
10048_at	10048	ENSG0000(	RANBP9	RAN bindir Secreted	I	11	1.325919	1.337343	1.326217	1.557002	1.6916	10.31146	162.4213	2.10E-07	7.73E-06	8
84803_at	84803	ENSG0000(	AGPAT9	1-acylglycerol-3-phosp	I	11	1.184182	1.191943	1.691986	1.529909	1.917378	8.66757	151.8241	2.68E-07	8.87E-06	8
2195_at	2195	ENSG0000(	FAT1	FAT tumor suppressor	I	11	1.184582	1.121486	1.521361	1.591197	1.907194	10.79441	162.0343	2.11E-07	7.79E-06	8
1958_at	1958	ENSG0000(	EGR1	early growth response	I	65	0.921127	1.163383	1.535008	1.578538	1.943195	5.017136	191.434	1.14E-07	5.51E-06	8
387914_at	387914	ENSG0000(	SHISA2	shisa homolog 2 (Xeno	I	22	1.019999	0.949777	1.701868	1.52735	1.768715	4.885696	136.5709	3.96E-07	1.12E-05	8
131583_at	131583	ENSG0000(	FAM43A	family with sequence s	I	11	1.019203	1.032543	1.643977	1.425992	1.826232	6.151175	70.16923	4.48E-06	5.59E-05	8
1407_at	1407	ENSG0000(	CRY1	cryptochro G-protein c	I	11	0.976756	1.076899	1.856336	1.61972	1.769667	8.4155	194.3111	1.08E-07	5.36E-06	8
3433_at	3433	ENSG0000(	IFIT2	interferon-induced prc	I	22	1.264666	0.959539	1.796301	1.344987	1.665377	6.070571	217.0552	7.21E-08	4.33E-06	8

56062_at	56062	ENSG0000(	KLHL4	kelch-like 4 (Drosophil	I	33	1.225744	0.852217	1.647318	1.325014	1.863838	4.686931	46.55017	1.96E-05	0.000161	8
25816_at	25816	ENSG0000(	TNFAIP8	tumor necrosis factor,	I	22	1.240044	1.156511	1.945909	1.377086	2.058902	4.752061	87.09011	2.05E-06	3.30E-05	8
5089_at	5089	NA	PBX2	pre-B-cell leukemia ho	I	8	1.31336	1.082066	1.622639	1.228893	1.380332	6.053232	82.19516	2.53E-06	3.79E-05	8
84142_at	84142	ENSG0000(	FAM175A	family with sequence s	I	15	1.232932	0.937666	1.720159	1.239299	1.369565	5.072837	98.85727	1.29E-06	2.41E-05	8
9456_at	9456	ENSG0000(	HOMER1	homer homolog 1 (Drc	I	37	1.363178	0.966752	1.574698	1.219611	1.191993	5.49875	119.5687	6.44E-07	1.56E-05	8
121441_at	121441	ENSG0000(	NEDD1	neural precursor cell e	I	44	1.081186	0.941878	1.593312	1.136443	1.469099	6.868826	188.5096	1.21E-07	5.68E-06	8
2764_at	2764	ENSG0000(	GMFB	glia maturation factor,	I	21	1.138999	0.99962	1.509979	1.04302	1.370693	7.534892	83.09679	2.43E-06	3.69E-05	8
113263_at	113263	ENSG0000(	GLCCI1	glucocorticoid induced	I	22	1.071608	0.797805	1.613675	1.040075	1.277533	5.734882	140.3511	3.58E-07	1.05E-05	8
79627_at	79627	ENSG0000(	OGFRL1	opioid growth factor re	I	17	1.206822	1.184228	1.745987	1.14295	1.355796	6.198186	78.13272	3.04E-06	4.31E-05	8
79633_at	79633	ENSG0000(	FAT4	FAT tumor suppressor	I	22	1.075111	1.253406	1.596284	1.202091	1.40491	4.201544	108.9211	9.06E-07	1.97E-05	8
3673_at	3673	ENSG0000(	ITGA2	integrin, alpha 2 (CD49	I	22	1.10013	0.991923	1.709478	1.36279	1.335239	9.395028	168.6859	1.82E-07	7.13E-06	8
22822_at	22822	ENSG0000(	PHLDA1	pleckstrin homology-li	I	44	1.113742	1.157468	1.587166	1.183292	1.165599	10.47756	107.1014	9.63E-07	2.03E-05	8
7048_at	7048	ENSG0000(	TGFBR2	transformi kinase	I	22	1.027345	1.179446	1.435264	1.203406	1.157101	6.775135	113.5397	7.78E-07	1.77E-05	8
3720_at	3720	ENSG0000(	JARID2	jumonji, AT rich intera	I	52	1.150873	1.237493	1.417874	1.297042	1.220049	5.334791	179.7724	1.44E-07	6.27E-06	8
29940_at	29940	ENSG0000(	DSE	dermatan sulfate epim	I	40	1.177414	1.291381	1.554793	1.16734	1.100781	4.266918	176.7452	1.54E-07	6.53E-06	8
84282_at	84282	ENSG0000(	RNF135	ring finger protein 135	I	22	1.210921	1.18872	1.538262	1.07083	1.070486	6.816507	151.0093	2.74E-07	8.95E-06	8
4651_at	4651	ENSG0000(	MYO10	myosin X	I	36	0.990942	0.975577	1.444846	1.430894	1.125277	5.220121	123.481	5.73E-07	1.45E-05	8
10049_at	10049	ENSG0000(	DNAJB6	DnaJ (Hsp40) homolog	I	34	1.13179	1.070899	1.337238	1.024851	1.227324	8.041116	72.86807	3.91E-06	5.10E-05	8
222389_at	222389	ENSG0000(	BEND7	BEN domain containin	I	22	1.190039	1.030916	1.386395	0.982694	1.13502	4.052986	124.5004	5.56E-07	1.42E-05	8
132_at	132	ENSG0000(	ADK	adenosine kinase	I	20	1.053009	0.968563	1.476769	0.966896	1.184442	7.753999	97.24361	1.37E-06	2.51E-05	8
2123_at	2123	ENSG0000(	EVI2A	ecotropic viral integrat	I	11	1.281612	1.047167	1.437534	0.870943	1.224693	7.501895	49.16889	1.61E-05	0.000141	8
7026_at	7026	ENSG0000(	NR2F2	nuclear rec nuclear rec	I	33	1.265039	1.050045	1.254936	1.050421	1.272868	9.140245	125.0004	5.47E-07	1.41E-05	8
1432_at	1432	ENSG0000(	MAPK14	mitogen-ac kinase	I	50	1.165689	1.017156	1.184928	0.988632	1.272119	5.575797	121.1747	6.13E-07	1.51E-05	8
5228_at	5228	ENSG0000(	PGF	placental g Secreted	NI	11	1.440884	1.288835	1.551595	0.973143	1.382577	4.611533	54.70561	1.10E-05	0.000106	8
5272_at	5272	ENSG0000(	SERPINB9	serpin peptidase inhib	I	33	1.576761	1.066201	1.444703	0.968264	1.22326	4.18732	102.788	1.12E-06	2.22E-05	8
23232_at	23232	ENSG0000(	TBC1D12	TBC1 domain family, m	I	11	1.354021	1.335112	1.383934	1.158058	1.539773	6.629979	106.6531	9.78E-07	2.04E-05	8
55752_at	55752	ENSG0000(	11-Sep	septin 11	I	44	1.209289	1.210432	1.456075	1.228958	1.5887	6.803133	157.7951	2.33E-07	8.12E-06	8
7112_at	7112	ENSG0000(	TMPO	thymopoie Secreted	I	62	1.318876	1.108248	1.305071	1.171067	1.710441	4.875241	102.2005	1.14E-06	2.24E-05	8
51363_at	51363	ENSG0000(	CHST15	carbohydrate (N-acety	I	31	1.345487	1.067512	1.502742	1.094954	1.536744	4.354018	72.41433	4.00E-06	5.18E-05	8
199704_at	199704	ENSG0000(	ZNF585A	zinc finger protein 585	I	22	1.435459	1.119543	1.499778	1.335348	1.36985	4.787691	209.6819	8.19E-08	4.71E-06	8
6591_at	6591	ENSG0000(	SNAI2	snail homolog 2 (Drosc	I	11	1.377655	0.934752	1.437884	1.303743	1.349773	8.735433	75.01787	3.52E-06	4.76E-05	8
8187_at	8187	ENSG0000(	ZNF239	zinc finger protein 239	I	11	1.338855	1.022833	1.254603	1.252422	1.402935	4.053652	29.13064	0.000102	0.000575	8
5783_at	5783	ENSG0000(	PTPN13	protein tyr phosphopr	I	22	1.05299	1.007786	1.263969	1.178871	1.338415	4.239608	97.73828	1.34E-06	2.47E-05	8
81539_at	81539	ENSG0000(	SLC38A1	solute carri transporte	I	54	1.007711	1.011191	1.198436	1.17739	1.331956	6.638065	36.51581	4.63E-05	0.00031	8
55860_at	55860	ENSG0000(	ACTR10	actin-related protein 1	I	22	1.078938	0.930699	1.173806	1.144472	1.417922	6.279461	151.0863	2.73E-07	8.95E-06	8
9263_at	9263	ENSG0000(	STK17A	serine/thre kinase	I	33	1.050135	0.979979	1.314111	1.228478	1.451838	6.330891	115.2743	7.36E-07	1.70E-05	8
1948_at	1948	ENSG0000(	EFNB2	ephrin-B2	NI	42	1.098458	0.994453	1.066409	1.311708	1.371818	4.788574	105.9104	1.00E-06	2.08E-05	8
51015_at	51015	ENSG0000(	ISOC1	isochorismatase doma	I	11	1.025771	0.933359	1.153228	1.295663	1.418478	7.830814	123.0551	5.80E-07	1.47E-05	8
51278_at	51278	ENSG0000(	IER5	immediate early respo	I	22	1.21252	0.86308	1.110607	1.294194	1.345589	6.024789	135.5843	4.07E-07	1.15E-05	8
79623_at	79623	ENSG0000(	GALNT14	UDP-N-ace Secreted	I	19	1.199084	0.752516	1.305978	1.096395	1.653891	4.018737	118.8384	6.59E-07	1.58E-05	8
120227_at	120227	ENSG0000(	CYP2R1	cytochrome P450, fam	I	11	1.150769	0.744135	1.368245	1.112732	1.497093	6.735252	136.6156	3.95E-07	1.12E-05	8
51191_at	51191	ENSG0000(	HERC5	hect domain and RLD 5	I	10	1.182953	0.882081	1.436568	1.413507	1.617927	8.335034	55.43062	1.05E-05	0.000102	8
2004_at	2004	ENSG0000(	ELK3	ELK3, ETS-domain prot	I	11	1.173008	0.963738	1.319776	1.30159	1.893904	6.418787	66.36029	5.48E-06	6.47E-05	8
653121_at	653121	ENSG0000(	ZBTB8A	zinc finger Secreted	I	22	1.184129	1.020417	1.146788	1.095283	1.875576	5.315273	110.2635	8.66E-07	1.90E-05	8
8701_at	8701	ENSG0000(	DNAH11	dynein, axc peptidase	I	10	1.375206	1.275295	0.94543	1.190897	1.852638	4.906392	129.4854	4.81E-07	1.29E-05	8
23405_at	23405	ENSG0000(	DICER1	dicer 1, ribonuclease ty	I	25	1.381395	1.281006	0.915123	0.918932	1.671444	6.189538	64.81527	5.97E-06	6.87E-05	8
7082_at	7082	ENSG0000(	TJP1	tight junction protein 1	I	19	0.913749	1.107125	1.078238	1.2094	1.385726	7.542371	73.7118	3.75E-06	4.95E-05	8
6594_at	6594	ENSG0000(	SMARCA1	SWI/SNF related, matr	I	55	0.86722	0.994702	1.032438	1.220349	1.439174	8.927785	105.3074	1.02E-06	2.10E-05	8
9867_at	9867	ENSG0000(	PJA2	praja ring finger 2	I	33	0.873597	0.912357	1.050643	1.230968	1.573403	7.287751	144.5367	3.22E-07	9.91E-06	8
3490_at	3490	ENSG0000(	IGFBP7	insulin-like Secreted	I	15	0.680945	0.964282	1.185351	1.27305	1.424477	12.04215	74.20729	3.66E-06	4.88E-05	8
2700_at	2700	ENSG0000(	GJA3	gap junction protein, a	I	11	0.583987	0.970068	1.10717	1.310569	1.48754	3.827217	120.6676	6.23E-07	1.52E-05	8
10970_at	10970	ENSG0000(	CKAP4	cytoskeleton-associate	I	22	0.741864	1.198272	1.175858	1.258957	1.328597	10.15544	140.0257	3.61E-07	1.06E-05	8
8204_at	8204	ENSG0000(	NRIP1	nuclear receptor intera	I	22	0.874217	1.167067	1.174725	1.531982	1.769925	4.268794	49.89024	1.53E-05	0.000135	8
8660_at	8660	ENSG0000(	IRS2	insulin receptor substr	I	22	0.908604	1.045244	1.279856	1.420317	1.766751	5.949641	31.38314	7.89E-05	0.000469	8
4312_at	4312	ENSG0000(	MMP1	matrix met peptidase	I	19	0.738752	0.93888	1.514897	1.231324	1.646971	12.72889	30.34034	8.87E-05	0.000514	8
79940_at	79940	NA	LINC00472	long intergenic non-pr	I	11	0.605306	1.007847	1.332683	1.105486	1.882225	4.427073	90.28732	1.80E-06	3.01E-05	8
51315_at	51315	ENSG0000(	KRCC1	lysine-rich coiled-coil 1	I	22	0.720663	0.816219	1.308924	1.498959	1.685107	5.101323	38.76498	3.75E-05	0.000265	8
57045_at	57045	ENSG0000(	TWSG1	twisted gas Secreted	I	32	0.591874	0.682907	1.387115	1.275017	1.649078	7.315277	72.33332	4.01E-06	5.19E-05	8
84547_at	84547	ENSG0000(	PGBD1	piggyBac transposable	I	21	0.909143	1.283126	1.653542	1.531372	1.629495	6.485333	85.76906	2.16E-06	3.43E-05	8
11066_at	11066	ENSG0000(	SNRNP35	small nuclear ribonucle	I	11	0.932351	1.248497	1.677057	1.539757	1.53573	7.638244	205.1114	8.88E-08	4.89E-06	8
157567_at	157567	ENSG0000(	ANKRD46	ankyrin repeat domain	I	11	0.9548	1.273566	1.803723	1.425822	1.603384	6.079895	191.1385	1.15E-07	5.52E-06	8
5420_at	5420	ENSG0000(	PODXL	podocalyxi Secreted	I	32	0.941618	1.381254	1.84318	1.55327	1.516296	7.529162	83.1449	2.42E-06	3.69E-05	8
4052_at	4052	ENSG0000(	LTBP1	latent trans kinase	I	53	1.097259	1.362575	1.777336	1.693328	1.624355	7.654133	173.7746	1.63E-07	6.81E-06	8
4675_at	4675	ENSG0000(	NAP1L3	nucleosome assembly	I	22	1.099742	1.34777	1.711543	1.584783	1.606407	4.500438	173.2534	1.65E-07	6.81E-06	8
1362_at	1362	ENSG0000(	CPD	carboxypep peptidase	I	41	0.936416	1.391888	1.733665	1.677545	1.625186	7.402589	260.6315	3.67E-08	2.93E-06	8
57182_at	57182	ENSG0000(	ANKRD50	ankyrin repeat domain	I	22	1.079065	1.415682	1.828743	1.540548	1.905405	6.629518	114.2775	7.60E-07	1.74E-05	8
492311_at	492311	ENSG0000(	C5orf53	chromosome 5 open re	I	11	1.032266	1.349394	1.744896	1.716654	1.888924	5.849335	100.794	1.20E-06	2.31E-05	8
282809_at	282809	ENSG0000(	POC1B	POC1 centriolar protei	I	32	1.201863	1.345862	1.754538	1.422579	1.773905	6.321924	120.1831	6.32E-07	1.54E-05	8
26298_at	26298	ENSG0000(	EHF	ets homologous factor	I	82	1.1806	1.355406	1.602362	1.575615	1.682254	4.71046	157.2618	2.36E-07	8.17E-06	8
2069_at	2069	ENSG0000(	EREG	epiregulin Secreted	I	31	1.195892	1.490935	1.753442	1.716241	1.656151	4.678084	51.06823	1.41E-05	0.000126	8
29761_at	29761	ENSG0000(	USP25	ubiquitin sp peptidase	I	32	1.127897	1.66996	1.686869	1.696191	1.586785	5.160996	126.7422	5.20E-07	1.35E-05	8
2530_at	2530	ENSG0000(	FUT8	fucosyltransferase 8 (a	I	21	1.260029	1.408276	1.720295	1.718261	1.484481	9.040227	99.18527	1.27E-06	2.39E-05	8
157285_at	157285	ENSG0000(	SGK223	homolog o kinase	I	11	1.209468	1.383823	1.566405	1.64791	1.45673	7.760227	153.2862	2.59E-07	8.65E-06	8
6857_at	6857	ENSG0000(	SYT1	synaptotag transporte	I	33	1.063872	1.615861	1.48894	1.684191	1.986804	5.021005	46.09354	2.03E-05	0.000166	8
27154_at	27154	ENSG0000(	BRPF3	bromodomain and PHI	I	11	1.056058	1.638258	1.538454	1.566748	1.956492	5.967753	148.9521	2.88E-07	9.25E-06	8
6617_at	6617	ENSG0000(	SNAPC1	small nuclear RNA acti	I	22	1.100194	1.634561	1.650735	1.743526	1.948644	7.618225	103.2344	1.10E-06	2.20E-05	8
100170841	1E+08	ENSG0000(	C17orf96	chromosome 17 open	I	11	1.156547	1.508999	1.45202	1.707734	2.212042	5.655261	146.1848	3.08E-07	9.69E-06	8
3491_at	3491	ENSG0000(	CYR61	cysteine-ric Secreted	I	22	1.019449	1.735682	1.228309	1.715778	1.871548	11.54634	47.07816	1.88E-05	0.000157	8
6038_at	6038	ENSG0000(	RNASE4	ribonuclea: Secreted	I	22	0.665389	1.172658	1.357006	1.918912	1.993281	5.358494	61.78987	7.09E-06	7.73E-05	8
59277_at	59277	ENSG0000(	NTN4	netrin 4 Secreted	I	20	0.506774	1.540561	1.451245	1.734329	1.540071	5.857903	34.56036	5.62E-05	0.000361	8
283078_at	283078	ENSG0000(	MKX	mohawk homeobox	I	11	0.637962	1.568568	1.224889	1.936274	1.368927	4.134604	32.10241	7.28E-05	0.000441	8
3728_at	3728	NA	JUP	junction plakoglobin	I	11	0.824533	1.206913	1.3947	1.827317	1.486541	6.670181	84.85731	2.25E-06	3.54E-05	8

163126_at	163126	ENSG00000(	EID2	EP300 interacting inhit	I	11	0.926478	1.420061	2.164007	1.45003	1.796591	7.416643	142.5678	3.38E-07	1.02E-05	8
7754_at	7754	NA	ZNF204P	zinc finger protein 204	I	11	0.859429	1.228537	2.143098	1.622056	1.812291	4.305485	99.62498	1.25E-06	2.37E-05	8
5274_at	5274	ENSG00000(	SERPINI1	serpin pept Secreted	I	9	0.657522	1.951703	2.0582	1.599866	1.630251	5.182295	66.06916	5.57E-06	6.54E-05	8
2512_at	2512	ENSG00000(	FTL	ferritin, light polypepti	I	17	1.151402	1.321303	1.224547	1.108953	1.172874	12.64325	56.1363	1.00E-05	9.90E-05	8
222068_at	222068	ENSG00000(	TMED4	transmemt Secreted	I	19	1.206679	1.332775	1.149078	1.085555	1.032556	7.933929	133.4874	4.30E-07	1.20E-05	8
8910_at	8910	ENSG00000(	SGCE	sarcoglycan, epsilon	I	31	1.070578	1.306038	1.127889	1.293493	1.077165	8.784752	139.9432	3.62E-07	1.06E-05	8
55770_at	55770	ENSG00000(	EXOC2	exocyst complex comp	I	29	1.07463	1.213264	1.144951	1.204263	0.969406	7.487772	103.3281	1.10E-06	2.20E-05	8
84792_at	84792	ENSG00000(	C7orf70	chromosome 7 open re	NI	11	1.125108	1.204637	1.296642	1.176893	1.044298	5.846888	145.7899	3.12E-07	9.71E-06	8
6903_at	6903	ENSG00000(	TBCC	tubulin folding cofacto	I	22	1.093715	1.124667	1.256322	1.165582	1.023564	7.986551	64.91814	5.93E-06	6.85E-05	8
9477_at	9477	ENSG00000(	MED20	mediator complex sub	I	33	1.273987	1.158055	1.140152	1.2088	0.976855	6.462353	156.7308	2.39E-07	8.20E-06	8
4774_at	4774	ENSG00000(	NFIA	nuclear factor I/A	I	75	1.120713	1.085208	1.195884	1.040924	1.041223	3.953744	59.46549	8.14E-06	8.48E-05	8
88455_at	88455	ENSG00000(	ANKRD13A	ankyrin repeat domain	I	32	1.170121	1.042134	1.203072	1.024603	1.038377	7.994627	83.71812	2.36E-06	3.64E-05	8
9989_at	9989	ENSG00000(	PPP4R1	protein phosphatase 4	I	42	1.050794	1.066694	1.157719	1.10365	1.081101	7.63703	98.39484	1.31E-06	2.43E-05	8
5069_at	5069	ENSG00000(	PAPPA	pregnancy- peptidase	I	81	1.065216	1.020964	1.14727	1.009655	1.062576	4.373963	139.9881	3.62E-07	1.06E-05	8
642_at	642	ENSG00000(	BLMH	bleomycin peptidase	I	21	1.031514	1.184396	1.191704	1.087346	1.244266	8.133547	144.033	3.26E-07	9.92E-06	8
57639_at	57639	ENSG00000(	CCDC146	coiled-coil domain con	I	10	0.985725	1.070556	1.283324	1.210978	1.15833	4.271496	47.62018	1.81E-05	0.000153	8
23370_at	23370	ENSG00000(	ARHGEF18	Rho/Rac guanine nucle	I	11	1.002467	1.105839	0.997222	1.075069	1.072451	9.382493	89.77809	1.83E-06	3.05E-05	8
64928_at	64928	ENSG00000(	MRPL14	mitochondrial ribosom	I	9	0.934909	1.116332	0.979328	1.066273	1.040382	10.69105	106.8373	9.72E-07	2.04E-05	8
51645_at	51645	ENSG00000(	PPIL1	peptidylprolyl isomera	I	29	0.920741	1.10426	1.016513	1.018058	1.126622	10.41322	121.5435	6.07E-07	1.51E-05	8
23787_at	23787	ENSG00000(	MTCH1	mitochondrial carrier 1	I	18	1.034081	1.202979	0.973212	1.116316	1.016117	10.95224	127.9307	5.03E-07	1.33E-05	8
153527_at	153527	ENSG00000(	ZMAT2	zinc finger, matrin-type	I	11	0.972186	1.189279	0.857095	1.107501	1.129804	7.110919	72.01306	4.08E-06	5.24E-05	8
9369_at	9369	ENSG00000(	NRXN3	neurexin 3 Secreted	I	22	1.157952	1.138018	0.852039	1.119468	1.229777	3.994921	65.55244	5.73E-06	6.68E-05	8
55209_at	55209	ENSG00000(	SETD5	SET domain containing	I	20	1.036739	1.095105	0.893956	1.033788	1.279049	6.210928	144.002	3.26E-07	9.92E-06	8
9521_at	9521	ENSG00000(	EEF1E1	eukaryotic translation	I	4	1.220679	1.172687	1.076573	1.097328	1.063923	11.01945	131.8949	4.50E-07	1.23E-05	8
1778_at	1778	ENSG00000(	DYNC1H1	dynein, cytoplasmic 1,	I	34	1.267203	1.162258	1.047694	1.02271	1.079593	9.085244	62.10125	6.96E-06	7.64E-05	8
79642_at	79642	ENSG00000(	ARSJ	arylsulfatase family, m	I	44	1.149673	1.131341	1.057251	1.084921	1.122083	6.74591	123.3293	5.75E-07	1.46E-05	8
7468_at	7468	ENSG00000(	WHSC1	Wolf-Hirschhorn syndr	I	118	1.127347	1.266736	1.037065	0.91645	1.116123	5.339292	102.1201	1.15E-06	2.25E-05	8
81847_at	81847	ENSG00000(	RNF146	ring finger protein 146	I	11	0.971543	1.121163	1.146251	1.14329	1.05695	7.521027	143.2378	3.32E-07	1.01E-05	8
52_at	52	ENSG00000(	ACP1	acid phosp phosphopr	I	32	0.933846	1.137556	1.092797	1.166428	0.994761	8.353857	127.7439	5.06E-07	1.33E-05	8
382_at	382	ENSG00000(	ARF6	ADP-ribosy GTPase	I	9	0.898734	1.051219	1.13694	1.182245	1.128637	10.32568	132.5814	4.41E-07	1.22E-05	8
55596_at	55596	ENSG00000(	ZCCHC8	zinc finger, CCHC doma	I	11	0.968827	1.120058	1.26517	1.077231	1.052644	7.274317	48.03695	1.75E-05	0.00015	8
387882_at	387882	ENSG00000(	C12orf75	chromosome 12 open	I	11	0.938707	1.166719	1.186903	1.051923	1.133982	10.03166	113.4959	7.79E-07	1.77E-05	8
129642_at	129642	ENSG00000(	MBOAT2	membrane bound O-ac	I	31	0.900439	1.208884	1.268123	1.232968	1.05274	6.682012	107.5357	9.49E-07	2.02E-05	8
135293_at	135293	ENSG00000(	PM20D2	peptidase I peptidase	I	30	0.798595	1.130565	1.206352	1.237497	1.109632	4.163651	58.9495	8.40E-06	8.70E-05	8
3622_at	3622	ENSG00000(	ING2	inhibitor of growth fan	I	11	0.968854	1.236166	1.180676	0.849694	1.205358	8.086816	91.79217	1.69E-06	2.89E-05	8
6319_at	6319	ENSG00000(	SCD	stearoyl-CoA desatura:	I	17	0.808263	1.410371	1.120425	0.934282	1.065132	7.015454	78.13426	3.04E-06	4.31E-05	8
221662_at	221662	ENSG00000(	RBM24	RNA binding motif pro	I	51	1.317168	1.417009	1.226385	1.199868	0.757283	3.844814	53.81351	1.17E-05	0.00011	8
11145_at	11145	ENSG00000(	PLA2G16	phospholipase A2, gro	I	21	1.38184	1.322708	1.206448	1.198423	0.783732	5.79221	93.86034	1.56E-06	2.72E-05	8
57228_at	57228	ENSG00000(	SMAGP	small cell adhesion gly	I	22	1.265308	1.458944	1.183125	1.178386	0.84673	6.735326	163.5673	2.04E-07	7.63E-06	8
4953_at	4953	ENSG00000(	ODC1	ornithine decarboxylas	I	10	1.234963	1.349689	1.20675	1.360939	0.800423	10.11997	62.70854	6.72E-06	7.46E-05	8
26031_at	26031	ENSG00000(	OSBPL3	oxysterol binding prote	I	43	1.156761	1.306123	1.225462	1.159741	0.820541	7.641795	128.8347	4.90E-07	1.30E-05	8
55049_at	55049	ENSG00000(	C19orf60	chromosome 19 open	I	19	1.242486	1.180189	1.154916	1.130099	0.756399	8.605424	145.9338	3.10E-07	9.71E-06	8
54830_at	54830	ENSG00000(	NUP62CL	nucleoporin 62kDa C-t	NI	11	1.083879	1.482323	1.334684	1.046087	0.980149	4.187732	69.42439	4.66E-06	5.74E-05	8
60685_at	60685	ENSG00000(	ZFAND3	zinc finger, AN1-type d	I	22	0.987479	1.484442	1.261791	1.128515	0.959034	8.106489	144.2785	3.24E-07	9.92E-06	8
90637_at	90637	ENSG00000(	ZFAND2A	zinc finger, AN1-type d	I	9	1.039952	1.634556	1.179503	1.219018	0.995117	7.595096	115.2914	7.36E-07	1.70E-05	8
10400_at	10400	ENSG00000(	PEMT	phosphatidylethanolar	I	6	1.022209	1.513618	1.005628	1.221399	0.913646	7.976755	81.78265	2.57E-06	3.84E-05	8
65018_at	65018	ENSG00000(	PINK1	PTEN induc kinase	I	20	1.26814	1.435586	1.110703	1.217056	1.045494	7.92489	144.0215	3.26E-07	9.92E-06	8
1054_at	1054	ENSG00000(	CEBPG	CCAAT/enhancer bindi	I	22	1.161615	1.474289	0.974157	1.23771	1.031112	7.569382	99.25548	1.27E-06	2.39E-05	8
2314_at	2314	ENSG00000(	FLII	flightless I homolog (D	I	43	1.310444	1.584218	1.162273	1.304065	1.033762	9.269948	191.6198	1.14E-07	5.51E-06	8
55644_at	55644	ENSG00000(	OSGEP	O-sialoglyc peptidase	I	10	1.475828	1.412585	1.014528	1.281203	0.871157	7.887043	68.94003	4.78E-06	5.85E-05	8
9124_at	9124	ENSG00000(	PDLIM1	PDZ and LIM domain 1	I	21	1.387328	1.294745	0.993398	1.265397	1.097812	7.918346	107.9137	9.37E-07	2.00E-05	8
5137_at	5137	ENSG00000(	PDE1C	phosphodiesterase 1C,	I	20	1.441773	1.474787	0.932753	1.065076	0.882665	4.841398	57.61662	9.12E-06	9.25E-05	8
23129_at	23129	ENSG00000(	PLXND1	plexin D1	I	22	1.380376	1.496629	0.833532	1.025589	0.970573	4.316259	118.4688	6.66E-07	1.59E-05	8
65989_at	65989	ENSG00000(	DLK2	delta-like 2 homolog (I	I	11	1.332284	1.374669	0.885222	1.015703	0.900587	5.260092	23.73327	0.000208	0.000998	8
9517_at	9517	ENSG00000(	SPTLC2	serine palmitoyltransfe	I	30	1.16021	1.516186	0.799856	1.102344	1.012144	7.096775	120.9795	6.17E-07	1.52E-05	8
50619_at	50619	ENSG00000(	DEF6	differentially expresse	NI	9	0.804506	1.571442	0.981974	1.368105	0.90725	4.0593	41.20735	3.02E-05	0.000225	8
441094_at	441094	NA	FLJ42709	hypothetical LOC44109	I	8	0.798902	1.656487	0.954599	1.184853	0.679728	7.415985	50.40523	1.47E-05	0.000131	8
340719_at	340719	ENSG00000(	NANOS1	nanos homolog 1 (Dro	I	21	0.788656	1.520377	1.278021	1.290196	0.623656	4.544686	24.7314	0.00018	0.000891	8
84830_at	84830	ENSG00000(	C6orf105	chromosome 6 open re	I	21	0.83683	1.565816	1.191635	1.254533	0.55269	4.84467	76.90031	3.22E-06	4.48E-05	8
7466_at	7466	ENSG00000(	WFS1	Wolfram syndrome 1 (	NI	11	1.261271	1.557552	0.945071	1.176324	0.782734	5.277951	39.62322	3.47E-05	0.00025	8
80323_at	80323	ENSG00000(	CCDC68	coiled-coil domain con	I	29	1.283908	1.509288	0.729848	1.242281	0.628834	4.508719	36.57384	4.61E-05	0.000309	8
16_at	16	ENSG00000(	AARS	alanyl-tRNA synthetase	I	21	1.091695	1.408463	0.947908	1.149599	0.600311	7.321665	50.98883	1.41E-05	0.000127	8
10050743(	1.01E+08	NA	MICA	MHC class I polypeptid	I	21	1.048246	1.405639	0.721493	1.07136	0.553982	9.306788	53.49689	1.19E-05	0.000112	8
705_at	705	ENSG00000(	BYSL	bystin-like	I	10	1.217767	1.224735	0.769796	1.001976	0.944212	8.981488	107.7353	9.43E-07	2.01E-05	8
9117_at	9117	ENSG00000(	SEC22C	SEC22 vesicle traffickir	I	33	1.129653	1.187261	0.806694	1.009513	1.024793	6.480133	99.29225	1.27E-06	2.39E-05	8
51251_at	51251	ENSG00000(	NT5C3	5'-nucleoti Secreted	I	7	1.232063	1.142529	0.842297	0.885567	1.048546	9.948638	79.79374	2.81E-06	4.08E-05	8
8225_at	8225	ENSG00000(	GTPBP6	GTP binding protein 6	I	21	1.054547	1.275176	1.005755	0.997192	0.897593	7.338069	108.5131	9.18E-07	1.98E-05	8
201294_at	201294	ENSG00000(	UNC13D	unc-13 homolog D (C. (	I	41	1.092147	1.240537	1.055147	0.943338	0.860446	4.292799	67.77809	5.08E-06	6.12E-05	8
6416_at	6416	ENSG00000(	MAP2K4	mitogen-ac kinase	I	30	1.114154	1.265514	0.937934	0.791288	0.906182	5.338489	48.75077	1.66E-05	0.000144	8
79649_at	79649	ENSG00000(	MAP7D3	MAP7 domain containi	I	38	1.331099	1.11313	0.924008	0.956944	0.928997	8.087734	96.25665	1.42E-06	2.57E-05	8
27295_at	27295	ENSG00000(	PDLIM3	PDZ and LIM domain 3	NI	22	1.351244	1.146968	0.900924	0.94723	0.766113	3.877052	37.89178	4.07E-05	0.000281	8
50865_at	50865	ENSG00000(	HEBP1	heme binding protein :	I	11	1.129987	1.143651	0.995158	1.079305	0.770439	8.42973	78.81983	2.94E-06	4.21E-05	8
54963_at	54963	ENSG00000(	UCKL1	uridine-cyt kinase	I	18	1.22412	1.063861	1.025097	0.989129	0.814909	6.530702	68.12413	4.99E-06	6.03E-05	8
57552_at	57552	ENSG00000(	NCEH1	neutral cholesterol est	I	33	1.115984	1.163056	1.258408	0.835823	0.964631	5.704142	48.18603	1.73E-05	0.000149	8
286151_at	286151	ENSG00000(	FBXO43	F-box protein 43	NI	11	1.116346	1.262764	1.203766	0.821871	0.915492	4.950649	47.83713	1.78E-05	0.000151	8
11031_at	11031	ENSG00000(	RAB31	RAB31, me GTPase	I	40	1.143626	1.135661	1.343074	0.978087	0.921318	8.394709	146.7482	3.04E-07	9.64E-06	8
22898_at	22898	ENSG00000(	DENND3	DENN/MADD domain (	NI	42	1.419811	1.257014	1.137696	0.822286	0.839911	4.565239	104.7425	1.04E-06	2.13E-05	8
55024_at	55024	ENSG00000(	BANK1	B-cell scaffold protein	I	28	1.525458	0.939088	1.030927	1.063428	0.996903	4.114213	85.29281	2.21E-06	3.49E-05	8
57688_at	57688	ENSG00000(	ZSWIM6	zinc finger, SWIM-type	I	22	1.303233	1.147255	0.998703	0.737219	1.214464	8.501808	105.6388	1.01E-06	2.09E-05	8
1604_at	1604	ENSG00000(	CD55	CD55 mole Secreted	I	51	1.202646	0.903846	1.357851	1.136676	1.179115	9.367941	19.42977	0.00041	0.001732	8
317762_at	317762	ENSG00000(	CCDC85C	coiled-coil domain con	I	18	1.232749	0.873104	1.302467	0.999872	1.048412	5.033561	41.856	2.86E-05	0.000216	8
149111_at	149111	ENSG00000(	CNIH3	cornichon homolog 3 (	I	11	1.356029	0.920667	1.359426	1.238846	1.024621	5.000773	10.64033	0.002914	0.008803	8

55786_at	55786	ENSG0000(	ZNF415	zinc finger protein 415		NI	10	1.075632	0.825587	1.224507	0.981735	1.046487	3.703182	31.63545	7.67E-05	0.000459	8
147015_at	147015	ENSG0000(	DHRS13	dehydrogenase/reduct		I	11	1.069386	0.694201	1.27537	1.067427	1.005316	5.034858	40.24132	3.29E-05	0.000239	8
55975_at	55975	ENSG0000(	KLHL7	kelch-like 7 (Drosophil		I	42	1.050518	0.798662	1.25224	1.260259	1.003703	6.033478	78.60231	2.97E-06	4.24E-05	8
5125_at	5125	ENSG0000(	PCSK5	proprotein	peptidase	NI	10	1.185841	0.605084	1.215158	1.036012	0.908987	4.230316	51.4015	1.37E-05	0.000125	8
80303_at	80303	ENSG0000(	EFHD1	EF-hand domain family		I	20	1.200215	0.684385	1.209987	0.988106	0.8308	4.375013	34.43489	5.70E-05	0.000364	8
3600_at	3600	ENSG0000(	IL15	interleukin	Secreted	I	27	1.270614	0.637546	1.266435	0.811846	0.810893	3.905634	68.12171	4.99E-06	6.03E-05	8
63898_at	63898	ENSG0000(	SH2D4A	SH2 domain containing		NI	21	1.15123	0.664545	1.144156	0.788883	0.955404	4.543581	36.98001	4.43E-05	0.0003	8
2968_at	2968	NA	GTF2H4	general transcription fa		I	17	1.134009	1.303161	1.16843	1.061978	0.664709	6.915271	57.06264	9.44E-06	9.49E-05	8
2048_at	2048	ENSG0000(	EPHB2	EPH recept	kinase	I	43	1.21925	1.249814	1.212608	0.937313	0.655713	4.229177	74.34082	3.64E-06	4.87E-05	8
8061_at	8061	ENSG0000(	FOSL1	FOS-like antigen 1		I	16	1.205497	1.40112	1.170204	0.833338	0.613456	7.467194	83.29911	2.41E-06	3.67E-05	8
81029_at	81029	ENSG0000(	WNT5B	wingless-ty	Secreted	NI	32	1.235916	1.400192	1.391725	1.065975	0.535392	4.378876	79.67095	2.83E-06	4.10E-05	8
11226_at	11226	ENSG0000(	GALNT6	UDP-N-ace	Secreted	I	20	1.062785	1.241337	1.272556	1.211798	0.565298	3.99076	38.23721	3.94E-05	0.000275	8
84293_at	84293	ENSG0000(	C10orf58	chromosor	Secreted	I	8	1.010451	1.122443	1.330716	1.064081	0.673496	9.268504	124.4552	5.56E-07	1.42E-05	8
79661_at	79661	ENSG0000(	NEIL1	nei endonuclease VIII-l		NI	10	0.976026	1.153671	1.298405	0.890013	0.461064	3.988217	41.09449	3.05E-05	0.000227	8
5768_at	5768	ENSG0000(	QSOX1	quiescin Q	Secreted	I	11	1.13615	1.221966	0.993484	0.896511	0.565073	8.910469	57.73003	9.06E-06	9.20E-05	8
3911_at	3911	ENSG0000(	LAMA5	laminin, alp	Secreted	I	15	1.246771	1.191941	0.993042	0.864503	0.481861	4.05436	30.26603	8.95E-05	0.000517	8
3892_at	3892	ENSG0000(	KRT86	keratin 86		I	11	1.194754	1.355118	1.061512	0.8842	0.424018	5.112263	19.82793	0.000383	0.001639	8
25827_at	25827	ENSG0000(	FBXL2	F-box and l	Secreted	I	33	1.233751	1.175922	1.00772	1.159514	0.597847	4.26461	36.27303	4.74E-05	0.000316	8
9578_at	9578	ENSG0000(	CDC42BPB	CDC42 binc	kinase	I	11	1.22506	1.056919	1.131533	1.188083	0.504832	6.039242	24.66276	0.000182	0.000898	8
23645_at	23645	ENSG0000(	PPP1R15A	protein phosphatase 1		I	10	1.383188	0.967465	0.789592	1.237403	0.335731	7.056175	29.73589	9.52E-05	0.000543	8
10370_at	10370	ENSG0000(	CITED2	Cbp/p300-interacting t		I	22	1.438128	1.303749	1.578319	1.141852	0.928106	8.140675	18.83212	0.000456	0.00189	8
9424_at	9424	ENSG0000(	KCNK6	potassium	ion channe	I	31	1.345765	1.249544	1.515984	1.19261	0.863716	4.647943	157.1331	2.37E-07	8.18E-06	8
53407_at	53407	ENSG0000(	STX18	syntaxin 18		I	10	1.420174	1.170775	1.500618	1.132657	1.058987	6.366322	198.4138	1.00E-07	5.18E-06	8
3437_at	3437	ENSG0000(	IFIT3	interferon-induced prc		I	11	1.356123	1.017184	1.512021	1.0788	0.876004	7.845781	62.24699	6.91E-06	7.60E-05	8
151887_at	151887	ENSG0000(	CCDC80	coiled-coil	Secreted	I	57	1.533689	1.300813	1.595761	0.977265	0.861898	4.8234	112.443	8.06E-07	1.81E-05	8
389840_at	389840	ENSG0000(	MAP3K15	mitogen-ac	kinase	I	11	1.305145	1.199268	1.722887	0.949955	0.900925	4.175354	65.67416	5.69E-06	6.65E-05	8
51205_at	51205	ENSG0000(	ACP6	acid phosp	Secreted	I	11	1.321541	1.325841	1.822874	1.212734	1.171198	7.977831	90.27191	1.80E-06	3.01E-05	8
2669_at	2669	ENSG0000(	GEM	GTP binding protein ov		I	11	1.434554	1.292309	1.718169	1.263205	1.047564	7.980077	106.968	9.68E-07	2.04E-05	8
7078_at	7078	ENSG0000(	TIMP3	TIMP meta	Secreted	I	31	1.326985	1.108817	1.797471	1.074687	1.095715	8.630238	101.6393	1.17E-06	2.27E-05	8
3397_at	3397	ENSG0000(	ID1	inhibitor of DNA bindir		I	33	1.489227	1.00417	1.784686	0.972349	1.107768	8.044272	21.61346	0.000286	0.00129	8
374378_at	374378	ENSG0000(	GALNTL4	UDP-N-acetyl-alpha-D-		NI	11	1.758425	1.405457	1.782404	0.940051	1.018955	4.834538	33.87153	6.04E-05	0.00038	8
50861_at	50861	ENSG0000(	STMN3	stathmin-like 3		I	25	1.759047	0.924558	1.802894	1.080861	0.856147	4.770166	69.19234	4.71E-06	5.80E-05	8
9966_at	9966	ENSG0000(	TNFSF15	tumor necrosis factor (		I	21	1.441325	1.134122	1.764559	1.122106	0.290945	4.160017	120.8894	6.19E-07	1.52E-05	8
1969_at	1969	ENSG0000(	EPHA2	EPH recept	kinase	I	20	1.194048	1.6331	1.736213	1.429654	0.937793	7.940457	158.2134	2.31E-07	8.08E-06	8
598_at	598	ENSG0000(	BCL2L1	BCL2-like 1		I	22	1.292448	1.450156	1.669649	1.329542	0.930592	6.525897	125.7379	5.36E-07	1.39E-05	8
7032_at	7032	ENSG0000(	TFF2	trefoil factc	Secreted	I	9	1.063446	1.339649	1.517241	1.477795	0.766907	5.54333	41.62826	2.91E-05	0.000218	8
28227_at	28227	ENSG0000(	PPP2R3B	protein phc	phosphopr	I	9	1.036104	1.562387	1.714453	1.651355	1.019622	4.954802	187.2468	1.24E-07	5.79E-06	8
400451_at	400451	ENSG0000(	FAM174B	family with sequence s		I	9	1.178578	1.500497	1.64397	1.593354	0.9374	4.114238	46.18711	2.01E-05	0.000165	8
79887_at	79887	ENSG0000(	PLBD1	phospholipase B doma		NI	10	1.105142	1.885595	1.692953	1.060836	0.804646	4.168468	160.4572	2.19E-07	7.98E-06	8
56474_at	56474	ENSG0000(	CTPS2	CTP synthase II		I	17	1.491444	0.693603	1.126992	0.983698	0.558751	6.626851	91.7556	1.69E-06	2.89E-05	8
9022_at	9022	ENSG0000(	CLIC3	chloride int	ion channe	I	10	1.479955	0.309944	1.294616	1.089438	0.657589	3.969292	17.08306	0.000631	0.00246	8
2644_at	2644	ENSG0000(	GCHFR	GTP cyclohydrolase I fe		NI	9	1.938661	0.504392	1.090347	0.798519	1.265965	4.08888	23.8904	0.000203	0.00098	8
23452_at	23452	ENSG0000(	ANGPTL2	angiopoiet	Secreted	I	32	1.182409	0.773071	1.951153	1.53243	1.503705	4.500011	19.5531	0.000401	0.001701	8
90594_at	90594	ENSG0000(	ZNF439	zinc finger protein 439		I	11	1.578058	0.389417	1.778529	1.542087	1.259504	3.894764	74.04582	3.69E-06	4.90E-05	8
2317_at	2317	ENSG0000(	FLNB	filamin B, beta		I	22	0.675334	1.199725	0.868853	1.10399	0.836494	8.685189	111.7639	8.24E-07	1.84E-05	8
23593_at	23593	ENSG0000(	HEBP2	heme binding protein 2		I	10	0.682731	1.092865	0.724081	1.051193	0.89993	8.130914	59.71473	8.02E-06	8.39E-05	8
10490_at	10490	ENSG0000(	VTI1B	vesicle transport throu		I	6	0.539998	1.236118	0.741408	0.960803	0.815378	9.654161	75.07002	3.51E-06	4.75E-05	8
1318_at	1318	ENSG0000(	SLC31A2	solute carri	transporte	I	27	0.560448	1.105825	0.991543	1.082446	0.640102	6.186488	83.2642	2.41E-06	3.67E-05	8
9415_at	9415	ENSG0000(	FADS2	fatty acid desaturase 2		I	41	0.461545	1.259907	0.892126	1.10343	0.630004	4.514393	92.64911	1.63E-06	2.84E-05	8
11020_at	11020	ENSG0000(	IFT27	intraflagellar transport		NI	10	0.265943	1.257513	0.687952	1.084596	0.633926	5.075701	36.13532	4.81E-05	0.000319	8
90701_at	90701	ENSG0000(	SEC11C	SEC11 hom	peptidase	I	9	0.426992	1.468809	1.126496	1.424101	0.759658	7.194903	24.20379	0.000194	0.000944	8
7436_at	7436	ENSG0000(	VLDLR	very low de	Secreted	NI	11	0.255092	1.331983	1.101446	1.147321	0.988893	4.568279	34.15125	5.86E-05	0.000372	8
26037_at	26037	ENSG0000(	SIPA1L1	signal-induced prolifer		I	11	0.766263	1.178429	0.80988	1.423366	1.311153	6.508598	55.00858	1.08E-05	0.000104	8
65267_at	65267	ENSG0000(	WNK3	WNK lysine	kinase	I	11	0.433002	1.17501	0.603489	1.411564	1.450187	3.595957	76.57327	3.27E-06	4.53E-05	8
84101_at	84101	ENSG0000(	USP44	ubiquitin s	peptidase	I	22	0.855791	0.833769	0.808113	1.403505	1.150744	3.757692	61.54936	7.19E-06	7.79E-05	8
80144_at	80144	ENSG0000(	FRAS1	Fraser syndrome 1		I	20	0.587598	0.912146	0.571006	1.47524	1.147044	4.122558	45.13174	2.19E-05	0.000176	8
129790_at	129790	NA	C7orf13	chromosome 7 open re		I	22	0.8504	1.446118	0.881229	1.129993	1.216667	4.930618	143.0314	3.34E-07	1.01E-05	8
133022_at	133022	ENSG0000(	TRAM1L1	translocation associate		NI	11	0.765652	1.394333	0.746974	1.275702	1.071518	4.244005	66.40645	5.47E-06	6.47E-05	8
55855_at	55855	NA	FAM45B	family with sequence s		I	3	0.935716	1.315394	0.460888	1.027555	1.176629	7.48044	37.73315	4.13E-05	0.000285	8
55222_at	55222	ENSG0000(	LRRC20	leucine rich repeat con		I	11	1.061859	1.878242	0.516823	1.876855	1.108351	5.701921	71.30739	4.23E-06	5.38E-05	8
254295_at	254295	ENSG0000(	PHYHD1	phytanoyl-CoA dioxyge		I	10	0.982046	1.506598	0.58265	1.986506	1.215409	4.756035	37.21133	4.33E-05	0.000295	8
51184_at	51184	ENSG0000(	GPN3	GPN-loop GTPase 3		I	29	0.593587	0.810391	1.015951	0.975497	1.275588	8.723481	106.8334	9.72E-07	2.04E-05	8
5427_at	5427	ENSG0000(	POLE2	polymerase (DNA direc		I	7	0.614322	0.762409	0.957389	0.998271	1.264188	8.570542	75.68851	3.41E-06	4.66E-05	8
3091_at	3091	ENSG0000(	HIF1A	hypoxia inducible facto		I	77	0.658665	0.713715	1.034218	0.975909	1.184543	9.170491	55.0784	1.07E-05	0.000104	8
5911_at	5911	ENSG0000(	RAP2A	RAP2A, me	GTPase	I	22	0.576655	0.662347	1.078721	0.943607	1.227134	6.465959	28.04224	0.000117	0.000636	8
6558_at	6558	ENSG0000(	SLC12A2	solute carri	transporte	NI	31	0.625535	0.849683	1.004014	0.816109	1.232413	4.905187	38.8394	3.73E-05	0.000264	8
9184_at	9184	ENSG0000(	BUB3	budding uninhibited by		I	24	0.668318	0.795753	0.978236	0.839021	1.164881	9.256979	61.77937	7.10E-06	7.73E-05	8
5827_at	5827	ENSG0000(	PXMP2	peroxisomal membran		I	28	0.575798	0.735831	1.047483	0.862435	1.116393	7.394193	34.16942	5.85E-05	0.000372	8
201725_at	201725	ENSG0000(	C4orf46	chromosome 4 open re		I	20	0.551165	0.738601	1.014488	0.800741	1.172134	7.135694	33.3776	6.35E-05	0.000396	8
120_at	120	ENSG0000(	ADD3	adducin 3 (gamma)		I	55	0.646703	0.826772	1.126362	0.872213	1.097058	7.862794	62.26166	6.90E-06	7.60E-05	8
9364_at	9364	ENSG0000(	RAB28	RAB28, me	GTPase	I	30	0.626258	0.838112	1.128798	0.914988	1.032699	7.710521	75.43206	3.45E-06	4.70E-05	8
84333_at	84333	ENSG0000(	PCGF5	polycomb group ring fi		I	42	0.600242	0.885134	1.130742	0.938277	1.127408	6.838231	79.02699	2.91E-06	4.18E-05	8
5358_at	5358	ENSG0000(	PLS3	plastin 3		I	21	0.773087	0.73265	1.069525	0.924761	1.130596	10.86761	105.5112	1.02E-06	2.09E-05	8
9972_at	9972	ENSG0000(	NUP153	nucleopori	transporte	I	21	0.772906	0.879508	1.004615	0.879332	1.089877	8.263519	79.07213	2.91E-06	4.17E-05	8
10393_at	10393	ENSG0000(	ANAPC10	anaphase promoting c		I	11	0.573598	0.792735	0.924276	0.885346	1.043883	8.540199	50.91574	1.42E-05	0.000128	8
5747_at	5747	ENSG0000(	PTK2	PTK2 prote	kinase	I	33	0.533353	0.741327	0.855292	0.874746	1.018323	6.232988	41.87094	2.85E-05	0.000215	8
7374_at	7374	ENSG0000(	UNG	uracil-DNA glycosylase		I	19	0.496419	0.73858	0.93536	0.79293	1.043166	8.47928	68.87876	4.79E-06	5.86E-05	8
122553_at	122553	ENSG0000(	TRAPPC6B	trafficking protein part		NI	21	0.496426	0.855813	0.906559	1.042285	1.069355	6.108628	67.8818	5.05E-06	6.10E-05	8
10724_at	10724	ENSG0000(	MGEA5	meningioma expressed		I	43	0.504357	0.763283	0.886533	1.028772	1.011412	5.90635	35.35293	5.19E-05	0.000339	8
51053_at	51053	ENSG0000(	GMNN	geminin, DNA replicati		I	10	0.707974	0.939946	1.006502	1.039638	1.077341	10.66546	59.84771	7.96E-06	8.36E-05	8
400_at	400	ENSG0000(	ARL1	ADP-ribosy	GTPase	I	84	0.775359	0.970477	0.969096	1.026458	1.024951	8.349137	77.42944	3.14E-06	4.41E-05	8
51699_at	51699	ENSG0000(	VPS29	vacuolar protein sortin		I	55	0.803961	0.850896	1.095206	0.956455	1.070901	10.10517	85.12367	2.22E-06	3.50E-05	8

94239_at	94239	ENSG00000(	H2AFV	H2A histone family, m(	I	34	0.74446	0.870092	1.053145	1.040211	1.020685	7.894015	107.4524	9.52E-07	2.02E-05	8
5884_at	5884	ENSG00000(	RAD17	RAD17 homolog (S. po	I	18	0.652033	0.785849	1.126051	1.08096	0.973252	7.912414	92.55978	1.64E-06	2.84E-05	8
79609_at	79609	ENSG00000(	METTL21D	methyltransferase like	NI	14	0.729203	0.806184	0.960786	1.119438	0.959921	5.489356	97.89009	1.34E-06	2.47E-05	8
121457_at	121457	ENSG00000(	IKBIP	IKBKB interacting prot(	I	33	0.773736	0.997117	1.291291	1.064575	1.235619	7.774908	80.92937	2.67E-06	3.95E-05	8
166929_at	166929	ENSG00000(	SGMS2	sphingomy kinase	I	33	0.802177	0.94524	1.247191	1.016502	1.258713	4.397947	33.95672	5.98E-05	0.000379	8
26115_at	26115	ENSG00000(	TANC2	tetratricopeptide repe	I	11	0.733104	1.029918	1.22422	0.937247	1.105706	7.440389	33.52967	6.25E-05	0.000392	8
5530_at	5530	ENSG00000(	PPP3CA	protein ph(phosphopr	I	43	0.837385	0.882969	1.153303	0.984722	1.352778	8.381359	111.2581	8.38E-07	1.86E-05	8
7913_at	7913	ENSG00000(	DEK	DEK oncogene	I	69	0.825542	0.844386	1.144911	0.988081	1.367418	10.63629	117.8754	6.79E-07	1.61E-05	8
1871_at	1871	ENSG00000(	E2F3	E2F transcr Secreted	I	13	0.690882	0.889395	1.153146	0.888783	1.268016	7.605943	63.61749	6.38E-06	7.19E-05	8
27106_at	27106	ENSG00000(	ARRDC2	arrestin domain contai	I	11	0.562783	1.034959	0.995215	1.159269	1.203756	6.845991	69.21782	4.71E-06	5.79E-05	8
84527_at	84527	ENSG00000(	ZNF559	zinc finger protein 559	I	33	0.597852	0.981764	0.947121	1.091691	1.216013	6.23277	108.8392	9.08E-07	1.97E-05	8
28966_at	28966	ENSG00000(	SNX24	sorting nexin 24	I	42	0.676983	0.985059	0.995949	1.070863	1.26338	7.090809	55.51802	1.04E-05	0.000102	8
8148_at	8148	ENSG00000(	TAF15	TAF15 RNA polymeras(	I	15	0.741524	1.073086	1.00644	0.898382	1.2609	10.02397	70.99045	4.30E-06	5.42E-05	8
54453_at	54453	ENSG00000(	RIN2	Ras and Rab interactor	I	22	0.65987	1.039166	0.999909	0.894181	1.112075	7.139633	107.546	9.49E-07	2.02E-05	8
9397_at	9397	ENSG00000(	NMT2	N-myristoyltransferase	I	41	0.932041	0.919976	1.268398	1.138113	1.181813	7.056003	54.7088	1.10E-05	0.000106	8
81537_at	81537	ENSG00000(	SGPP1	sphingosine-1-phosph	I	22	0.894861	0.913297	1.246072	1.034026	1.200947	7.94349	94.01969	1.55E-06	2.72E-05	8
2908_at	2908	ENSG00000(	NR3C1	nuclear rec nuclear rec	I	106	0.927786	0.86434	1.241596	1.072667	1.298086	7.91317	127.2929	5.12E-07	1.34E-05	8
79768_at	79768	ENSG00000(	C15orf29	chromosome 15 open	NI	11	0.890912	0.865364	1.339076	0.93914	1.27231	6.057246	112.0911	8.16E-07	1.82E-05	8
4659_at	4659	ENSG00000(	PPP1R12A	protein phosphatase 1	I	47	1.024616	0.811607	1.250757	0.920065	1.219992	7.778552	118.2464	6.71E-07	1.60E-05	8
114882_at	114882	ENSG00000(	OSBPL8	oxysterol binding prot(	I	52	1.074354	0.880715	1.187784	1.051408	1.393883	6.051774	54.95881	1.08E-05	0.000105	8
6732_at	6732	ENSG00000(	SRPK1	SRSF prote kinase	I	25	0.995458	0.86184	1.17903	1.015269	1.371709	8.43325	86.66954	2.08E-06	3.33E-05	8
3075_at	3075	ENSG00000(	CFH	compleme Secreted	I	12	1.092149	0.90666	1.291818	1.058616	1.346986	4.132656	52.36994	1.28E-05	0.000119	8
79745_at	79745	ENSG00000(	CLIP4	CAP-GLY domain conta	I	41	0.828577	1.005141	1.415963	1.191832	1.274564	5.684324	136.0733	4.01E-07	1.13E-05	8
24138_at	24138	ENSG00000(	IFIT5	interferon-induced prc	I	20	0.868753	0.983397	1.342882	1.100435	1.138318	6.776014	73.78422	3.74E-06	4.94E-05	8
285331_at	285331	ENSG00000(	CCDC66	coiled-coil domain con	I	11	0.73549	0.852856	1.4422	0.997529	1.227845	5.834885	40.01468	3.35E-05	0.000243	8
51185_at	51185	ENSG00000(	CRBN	cereblon peptidase	I	29	0.850395	0.810676	1.19509	0.969177	1.119907	6.604153	75.29572	3.47E-06	4.72E-05	8
29896_at	29896	ENSG00000(	TRA2A	transformer 2 alpha hc	I	31	0.77945	0.747054	1.162261	0.981397	1.121166	7.466083	42.89846	2.62E-05	0.000202	8
56650_at	56650	ENSG00000(	CLDND1	claudin domain contai	I	19	0.786741	0.739586	1.253181	1.063131	1.109851	9.515343	105.6221	1.01E-06	2.09E-05	8
5515_at	5515	ENSG00000(	PPP2CA	protein ph(phosphopr	I	26	0.935192	0.658066	1.292226	1.080382	1.107927	8.555141	67.88111	5.05E-06	6.10E-05	8
54664_at	54664	ENSG00000(	TMEM106I	transmembrane protei	I	55	0.843446	0.619505	1.265681	0.972458	1.102765	7.331104	108.2434	9.27E-07	1.99E-05	8
6653_at	6653	ENSG00000(	SORL1	sortilin-rel transporte	I	22	0.836113	0.671285	1.331432	1.128651	1.322404	4.89975	26.65507	0.000139	0.000729	8
145482_at	145482	ENSG00000(	PTGR2	prostaglandin reducta	I	20	0.952815	0.663721	1.331834	1.044554	1.232828	4.364518	51.92115	1.33E-05	0.000122	8
57205_at	57205	ENSG00000(	ATP10D	ATPase, cla transporte	I	33	0.887702	0.742425	1.136309	1.084679	1.252199	7.870632	87.70837	1.99E-06	3.23E-05	8
55914_at	55914	ENSG00000(	ERBB2IP	erbb2 interacting prot(	I	54	0.850225	0.562141	1.079847	1.135429	1.223492	4.77394	125.2078	5.44E-07	1.40E-05	8
2296_at	2296	ENSG00000(	FOXC1	forkhead box C1	I	33	0.646456	0.570527	1.106977	1.126451	1.241759	7.201312	26.39514	0.000144	0.000748	8
3977_at	3977	ENSG00000(	LIFR	leukemia ir Secreted	NI	55	0.690198	0.626592	0.998723	1.114089	1.238408	4.427552	51.75657	1.34E-05	0.000122	8
55727_at	55727	ENSG00000(	BTBD7	BTB (POZ) domain con	I	54	1.107918	0.910437	0.889167	0.878319	1.195175	5.081841	89.4994	1.85E-06	3.07E-05	8
160418_at	160418	ENSG00000(	TMTC3	transmembrane and te	I	44	1.051797	0.826834	0.934254	0.859252	1.184673	6.049469	88.93964	1.90E-06	3.11E-05	8
64897_at	64897	ENSG00000(	C12orf43	chromosome 12 open	NI	22	1.082873	0.893013	0.93356	0.805427	1.108487	4.829253	112.7822	7.98E-07	1.80E-05	8
221830_at	221830	ENSG00000(	TWISTNB	TWIST neighbor	I	33	1.009056	0.96017	0.877851	0.873839	1.069717	7.636516	84.48326	2.29E-06	3.57E-05	8
6867_at	6867	ENSG00000(	TACC1	transforming, acidic co	I	88	1.00696	0.936146	0.801753	0.834252	1.113714	7.443506	68.34367	4.93E-06	5.98E-05	8
222658_at	222658	ENSG00000(	KCTD20	potassium ion channe	NI	33	1.043403	0.953312	0.806023	0.918837	1.202509	4.11039	43.52324	2.49E-05	0.000194	8
51306_at	51306	ENSG00000(	FAM13B	family with sequence s	I	32	0.861358	1.006561	0.779593	0.854053	1.032092	6.30756	35.06959	5.34E-05	0.000346	8
23118_at	23118	ENSG00000(	TAB2	TGF-beta activated kin	NI	40	0.913967	0.954746	0.799835	0.802042	1.032816	5.085743	92.88795	1.62E-06	2.81E-05	8
79646_at	79646	ENSG00000(	PANK3	pantothen kinase	NI	29	0.820525	0.963086	0.829563	0.882632	1.09944	5.523055	79.8856	2.80E-06	4.07E-05	8
57606_at	57606	ENSG00000(	SLAIN2	SLAIN motif family, me	I	41	0.932517	0.909855	0.91253	0.800553	1.178511	7.052969	77.34224	3.15E-06	4.41E-05	8
5576_at	5576	ENSG00000(	PRKAR2A	protein kin kinase	NI	31	0.92289	0.912328	0.793157	0.706443	1.060582	4.301382	48.05978	1.75E-05	0.00015	8
9352_at	9352	ENSG00000(	TXNL1	thioredoxin-like 1	I	15	0.891243	0.7755	1.000998	1.010669	0.930678	10.5458	75.09881	3.50E-06	4.75E-05	8
839_at	839	ENSG00000(	CASP6	caspase 6, peptidase	I	11	0.95781	0.707848	1.040966	0.941407	0.930472	8.020004	89.92678	1.82E-06	3.04E-05	8
64983_at	64983	ENSG00000(	MRPL32	mitochond Secreted	I	7	1.014404	0.657445	0.879623	0.939017	1.037113	9.129105	72.90054	3.90E-06	5.10E-05	8
90693_at	90693	ENSG00000(	CCDC126	coiled-coil domain con	NI	33	0.856919	0.619296	0.842837	0.867028	1.095913	5.329756	62.00596	7.00E-06	7.67E-05	8
801_at	801	NA	CALM1	calmodulin 1 (phosphc	I	33	0.983861	0.926469	0.998342	1.005401	1.012085	10.35407	77.95919	3.06E-06	4.33E-05	8
256471_at	256471	ENSG00000(	MFSD8	major facili transporte	NI	42	0.984327	0.868251	1.038786	0.971714	1.007253	5.462696	82.61205	2.48E-06	3.74E-05	8
51000_at	51000	ENSG00000(	SLC35B3	solute carrier family 3!	I	11	1.093743	0.983779	1.03735	1.042254	1.093118	8.543495	45.63465	2.10E-05	0.000171	8
5754_at	5754	ENSG00000(	PTK7	PTK7 prote kinase	I	42	1.005105	1.038009	1.107174	0.927324	1.086836	4.463946	41.03715	3.07E-05	0.000228	8
11157_at	11157	ENSG00000(	LSM6	LSM6 homolog, U6 sm	I	16	0.913766	1.037915	1.066547	0.994356	1.030561	10.11762	115.5618	7.30E-07	1.69E-05	8
51637_at	51637	ENSG00000(	C14orf166	chromosome 14 open	I	5	0.940215	1.057023	1.02649	0.824651	1.039678	11.40939	74.00496	3.70E-06	4.90E-05	8
7430_at	7430	ENSG00000(	EZR	ezrin	I	46	0.989354	0.993429	1.050698	0.793566	0.974996	7.429537	97.77534	1.34E-06	2.47E-05	8
23389_at	23389	ENSG00000(	MED13L	mediator complex sub	I	20	1.015881	1.090604	0.975412	0.835073	1.140913	6.931676	59.18862	8.28E-06	8.60E-05	8
286148_at	286148	ENSG00000(	DPY19L4	dpy-19-like 4 (C. elega	I	16	1.110157	0.925296	1.006762	0.925285	0.843461	6.181341	51.38453	1.38E-05	0.000125	8
23392_at	23392	ENSG00000(	KIAA0368	KIAA0368	I	20	1.031863	0.882019	1.009018	0.902845	0.826398	6.179934	31.28534	7.97E-05	0.000473	8
3017_at	3017	ENSG00000(	HIST1H2BD	histone cluster 1, H2b(	I	32	1.108433	0.800595	1.05306	1.015877	0.916844	7.797991	62.79122	6.69E-06	7.44E-05	8
84671_at	84671	ENSG00000(	ZNF347	zinc finger protein 347	I	22	1.134382	0.756508	0.86284	0.90342	0.961551	3.507077	74.18201	3.66E-06	4.89E-05	8
23228_at	23228	ENSG00000(	PLCL2	phospholipase C-like 2	NI	11	1.143741	0.739576	1.004999	0.835569	0.892497	3.620338	23.18319	0.000225	0.001066	8
91351_at	91351	ENSG00000(	DDX60L	DEAD (Asp-Glu-Ala-As	I	11	1.162985	0.967614	0.942507	0.833052	0.975213	6.944076	43.42745	2.51E-05	0.000195	8
26127_at	26127	ENSG00000(	FGFR1OP2	FGFR1 oncogene partn	I	33	0.959862	0.865667	1.192767	0.863424	0.892429	6.700824	86.73164	2.08E-06	3.33E-05	8
91298_at	91298	ENSG00000(	C12orf29	chromosome 12 open	I	14	1.032294	0.905727	1.153329	0.814346	0.857692	9.098284	82.22692	2.52E-06	3.79E-05	8
8091_at	8091	ENSG00000(	HMGA2	high mobility group AT	I	24	0.960964	0.788719	1.176142	0.801549	0.815734	5.62575	53.66749	1.18E-05	0.000111	8
57380_at	57380	ENSG00000(	MRS2	MRS2 mag Secreted	I	55	0.921815	0.866036	1.039154	0.86735	0.813648	6.678156	66.38654	5.47E-06	6.47E-05	8
6138_at	6138	ENSG00000(	RPL15	ribosomal protein L15	I	27	0.943227	0.797272	1.019386	0.849349	0.889773	10.75395	77.05035	3.19E-06	4.46E-05	8
56160_at	56160	ENSG00000(	NDNL2	necdin-like 2	I	11	0.884323	0.913373	1.17809	0.962311	0.924092	7.847745	71.32005	4.22E-06	5.38E-05	8
1846_at	1846	ENSG00000(	DUSP4	dual specif phosphopr	I	11	0.965785	0.804015	1.195609	0.929178	0.967545	11.69175	75.72853	3.40E-06	4.66E-05	8
54887_at	54887	ENSG00000(	UHRF1BP1	UHRF1 binding protein	I	18	0.835409	0.994406	1.24817	0.820015	1.006079	5.8892	106.5908	9.80E-07	2.04E-05	8
8573_at	8573	ENSG00000(	CASK	calcium/ca kinase	I	41	0.850766	0.854144	1.18571	0.870301	1.081114	6.071611	60.93352	7.46E-06	7.99E-05	8
51076_at	51076	ENSG00000(	CUTC	cutC copper transport(	I	20	1.027895	0.977539	1.247531	0.87576	1.078639	6.164857	64.88243	5.95E-06	6.85E-05	8
10892_at	10892	ENSG00000(	MALT1	mucosa as peptidase	I	44	0.84816	1.169504	0.954681	0.813142	0.930367	6.035697	68.74496	4.83E-06	5.89E-05	8
384_at	384	ENSG00000(	ARG2	arginase, type II	I	20	0.8993	1.118249	0.904578	0.862941	0.923281	6.615692	56.36111	9.87E-06	9.79E-05	8
51406_at	51406	ENSG00000(	NOL7	nucleolar protein 7, 27	I	7	0.753614	1.029841	1.042478	0.884233	0.955914	11.32163	100.2895	1.22E-06	2.34E-05	8
118_at	118	ENSG00000(	ADD1	adducin 1 (alpha)	I	49	0.77361	1.020814	0.986724	0.76913	0.899909	5.159777	89.36567	1.86E-06	3.07E-05	8
84874_at	84874	ENSG00000(	ZNF514	zinc finger protein 514	NI	33	0.766361	1.149893	1.191876	0.814181	0.965478	4.33722	56.67107	9.68E-06	9.68E-05	8
55051_at	55051	ENSG00000(	C14orf102	chromosome 14 open	NI	10	1.005671	0.925563	1.351997	0.758013	0.770063	5.639635	82.99751	2.44E-06	3.69E-05	8
2581_at	2581	ENSG00000(	GALC	galactosylc Secreted	NI	38	1.097121	0.879537	1.180206	0.7509	0.765499	3.863592	60.81421	7.51E-06	8.01E-05	8

55844_at	55844	ENSG0000(	PPP2R2D	protein phosphatase 2	I	9	0.902805	0.942937	1.064666	0.76472	0.669794	7.217276	33.93505	6.00E-05	0.000379	8
51143_at	51143	ENSG0000(	DYNC1LI1	dynein, cyt Secreted	I	18	0.863812	0.842888	1.090305	0.823471	0.677152	7.962499	59.86632	7.95E-06	8.35E-05	8
57619_at	57619	ENSG0000(	SHROOM3	shroom family membe	NI	55	0.867244	0.900193	1.067528	0.714297	1.047465	5.281615	25.38971	0.000165	0.000829	8
3099_at	3099	ENSG0000(	HK2	hexokinase kinase	I	10	0.826862	0.931672	1.110972	0.686703	0.902315	8.369398	26.01929	0.000151	0.000777	8
122769_at	122769	ENSG0000(	LRR1	leucine rich repeat pro	I	11	0.68872	0.781992	0.992738	0.619065	1.02844	8.692427	51.34938	1.38E-05	0.000125	8
132949_at	132949	ENSG0000(	AASDH	aminoadipate-semiald	NI	30	0.845268	0.925609	1.257422	0.665522	1.117129	6.029962	72.64036	3.95E-06	5.14E-05	8
63932_at	63932	ENSG0000(	CXorf56	chromosome X open re	NI	9	0.617323	0.950011	1.17348	0.576669	1.100449	5.007666	22.92349	0.000234	0.001099	8
163_at	163	ENSG0000(	AP2B1	adaptor-related protei	I	15	0.750015	0.94494	1.037728	1.031884	0.868546	9.007544	61.68325	7.14E-06	7.75E-05	8
334_at	334	ENSG0000(	APLP2	amyloid beta (A4) prec	I	43	0.79476	0.956897	1.028262	1.014519	0.77053	10.3996	47.11907	1.87E-05	0.000157	8
9592_at	9592	ENSG0000(	IER2	immediate early respo	I	8	0.642126	1.022071	1.141483	1.035184	0.744405	9.66464	88.71617	1.91E-06	3.12E-05	8
4323_at	4323	ENSG0000(	MMP14	matrix met peptidase	I	42	0.737507	1.041597	1.03155	1.018891	0.727143	7.327641	54.31341	1.13E-05	0.000108	8
51123_at	51123	ENSG0000(	ZNF706	zinc finger protein 706	I	13	0.603656	0.942634	1.037356	0.910435	0.884441	7.620156	64.13748	6.20E-06	7.04E-05	8
51194_at	51194	ENSG0000(	IPO11	importin 1: transporter	NI	19	0.595162	1.018103	1.010482	0.913041	0.807262	5.8013	71.19866	4.25E-06	5.40E-05	8
55352_at	55352	ENSG0000(	C17orf79	chromosome 17 open	I	10	0.652444	0.864033	1.069236	0.978876	0.892447	9.573274	47.90092	1.77E-05	0.000151	8
55692_at	55692	ENSG0000(	LUC7L	LUC7-like (S. cerevisiae	I	43	0.674681	0.835994	1.01399	0.94612	0.923125	7.015967	51.6877	1.35E-05	0.000123	8
3927_at	3927	ENSG0000(	LASP1	LIM and SH transporter	I	28	0.781409	0.856267	1.130533	0.907202	0.853066	8.810634	71.08033	4.28E-06	5.42E-05	8
51373_at	51373	NA	MRPS17	mitochondrial ribosom	I	10	0.589621	0.93222	1.139769	0.940845	0.685998	9.400925	68.4194	4.91E-06	5.97E-05	8
26020_at	26020	ENSG0000(	LRP10	low density lipoprotein	I	11	0.596461	0.927604	1.062644	1.018401	0.679267	7.00011	42.8438	2.63E-05	0.000203	8
23390_at	23390	ENSG0000(	ZDHHC17	zinc finger, DHHC-type	NI	41	0.751768	0.93844	1.169044	0.954806	0.624702	4.246443	43.40268	2.51E-05	0.000196	8
5478_at	5478	ENSG0000(	PPIA	peptidylprolyl isomera	I	40	0.782048	0.856536	1.285672	0.98764	1.008157	5.180326	79.15946	2.90E-06	4.17E-05	8
643641_at	643641	ENSG0000(	ZNF862	zinc finger protein 862	NI	11	0.779767	0.902836	1.257188	0.951742	0.947259	4.024685	67.41351	5.18E-06	6.20E-05	8
23186_at	23186	ENSG0000(	RCOR1	REST corepressor 1	I	22	0.764333	0.982204	1.266118	1.036246	0.956287	4.548983	77.04024	3.19E-06	4.46E-05	8
57110_at	57110	ENSG0000(	HRASLS	HRAS-like suppressor	NI	9	0.866388	0.778197	1.1731	1.142168	0.791316	4.412472	16.45494	0.000715	0.00273	8
929_at	929	ENSG0000(	CD14	CD14 mole Secreted	NI	22	0.874079	0.778959	1.2842	1.032267	0.76413	3.804328	103.9367	1.07E-06	2.18E-05	8
9645_at	9645	ENSG0000(	MICAL2	microtubule associate	I	31	0.981431	1.309646	1.162477	0.925821	0.788567	5.412804	102.3293	1.14E-06	2.24E-05	8
221710_at	221710	NA	LOC22171(	hypothetical protein L(	I	43	0.928502	1.293553	1.09018	0.982665	0.828969	6.362522	55.96904	1.01E-05	9.97E-05	8
50810_at	50810	ENSG0000(	HDGFRP3	hepatoma-derived gro	I	10	0.866788	1.206478	1.206615	1.040413	0.840351	7.59348	88.74742	1.91E-06	3.12E-05	8
323_at	323	ENSG0000(	APBB2	amyloid beta (A4) prec	I	74	0.904773	1.198648	1.095162	1.009386	0.922351	6.567254	101.2574	1.18E-06	2.29E-05	8
1119_at	1119	ENSG0000(	CHKA	choline kin kinase	I	18	0.952415	1.06394	1.258782	1.018213	0.760426	7.120669	90.01597	1.81E-06	3.03E-05	8
5528_at	5528	ENSG0000(	PPP2R5D	protein phosphatase 2	I	47	0.855617	1.065268	1.176284	0.971763	0.746449	6.102655	56.41658	9.84E-06	9.77E-05	8
10667_at	10667	ENSG0000(	FARS2	phenylalanyl-tRNA syn	I	20	0.975612	1.105705	1.162384	0.948673	0.614269	6.249648	93.08277	1.61E-06	2.80E-05	8
5910_at	5910	ENSG0000(	RAP1GDS1	RAP1, GTP-GDP dissoc	I	31	0.981117	1.220376	0.926352	1.014766	0.64717	7.79192	96.5891	1.40E-06	2.55E-05	8
3628_at	3628	ENSG0000(	INPP1	inositol polyphosphate	I	11	0.892564	1.141247	0.944746	1.083865	0.628282	8.556818	92.47282	1.65E-06	2.84E-05	8
7052_at	7052	ENSG0000(	TGM2	transglutaminase 2 (C	I	44	1.002695	1.226522	1.051384	1.170452	0.701108	4.927631	106.4735	9.84E-07	2.05E-05	8
572_at	572	ENSG0000(	BAD	BCL2-associated agoni	I	21	0.68367	1.265201	1.120705	1.135052	0.731452	8.124562	94.0561	1.55E-06	2.71E-05	8
7263_at	7263	ENSG0000(	TST	thiosulfate sulfurtrans	I	11	0.832031	1.266929	1.119317	1.077434	0.615785	8.100849	112.785	7.97E-07	1.80E-05	8
2026_at	2026	ENSG0000(	ENO2	enolase 2 (gamma, ne	NI	11	0.624867	1.080213	1.123584	1.226878	0.566344	5.254124	74.41142	3.62E-06	4.85E-05	8
84498_at	84498	ENSG0000(	FAM120B	family with Secreted	NI	32	0.88435	1.112245	0.935413	1.149319	0.908773	4.87827	75.93084	3.37E-06	4.63E-05	8
51562_at	51562	ENSG0000(	MBIP	MAP3K12 binding inhil	I	20	0.896986	1.155157	0.86263	1.10334	0.880185	7.928363	118.1133	6.74E-07	1.60E-05	8
136_at	136	ENSG0000(	ADORA2B	adenosine G-protein c	I	11	0.975376	1.146511	0.867232	1.070605	0.956789	10.69354	59.73643	8.01E-06	8.39E-05	8
1161_at	1161	ENSG0000(	ERCC8	excision repair cross-cc	NI	56	1.023636	1.095775	0.737761	1.132186	0.961885	5.73679	71.63568	4.16E-06	5.32E-05	8
374969_at	374969	ENSG0000(	CCDC23	coiled-coil domain con	I	9	0.895181	1.086253	0.904186	0.993491	1.069135	7.722192	64.47174	6.08E-06	6.97E-05	8
5939_at	5939	ENSG0000(	RBMS2	RNA binding motif, sin	NI	74	1.017484	1.03677	0.855671	0.985287	1.009876	4.351387	73.76181	3.74E-06	4.95E-05	8
10455_at	10455	ENSG0000(	ECI2	enoyl-CoA delta isome	I	11	0.999723	1.098133	0.924666	0.977266	0.936152	10.72131	96.08964	1.43E-06	2.58E-05	8
6636_at	6636	ENSG0000(	SNRPF	small nuclear ribonucl	I	22	0.931743	1.094093	0.946744	0.954581	0.91183	10.38703	114.0212	7.66E-07	1.76E-05	8
55304_at	55304	ENSG0000(	SPTLC3	serine palmitoyltransfe	I	20	0.951361	0.909304	1.017067	1.051895	0.861876	4.419349	74.12564	3.67E-06	4.90E-05	8
78990_at	78990	ENSG0000(	OTUB2	OTU domai peptidase	NI	31	0.894715	0.937351	0.932733	1.08354	0.810511	4.478655	64.45369	6.09E-06	6.97E-05	8
63933_at	63933	ENSG0000(	CCDC90A	coiled-coil Secreted	I	8	0.947541	0.919263	0.93667	1.15066	0.991889	9.343092	97.60176	1.35E-06	2.48E-05	8
4832_at	4832	ENSG0000(	NME3	non-metas kinase	I	11	0.784649	1.04485	1.20767	1.142573	0.873059	7.387143	50.94863	1.42E-05	0.000127	8
4267_at	4267	ENSG0000(	CD99	CD99 molecule	I	25	0.818998	1.037296	1.082956	1.131973	0.833755	9.128224	64.25239	6.16E-06	7.02E-05	8
7050_at	7050	ENSG0000(	TGIF1	TGFB-induced factor h	I	17	0.776581	1.116408	1.119425	1.255614	0.961222	7.911269	106.1598	9.95E-07	2.07E-05	8
2941_at	2941	ENSG0000(	GSTA4	glutathione S-transfera	I	44	0.881675	1.109199	0.950809	1.315935	1.023488	5.957156	74.02008	3.69E-06	4.90E-05	8
5641_at	5641	ENSG0000(	LGMN	legumain peptidase	I	39	0.677279	1.078994	0.923556	1.27004	0.797258	8.283662	102.5947	1.13E-06	2.23E-05	8
23113_at	23113	ENSG0000(	CUL9	cullin 9	I	10	1.08293	1.039522	0.867706	1.492013	0.896369	5.889965	78.75894	2.95E-06	4.22E-05	8
55743_at	55743	ENSG0000(	CHFR	checkpoint with forkhe	I	18	1.136865	0.752502	1.029416	1.075016	0.723149	4.148688	52.78656	1.25E-05	0.000116	8
4756_at	4756	ENSG0000(	NEO1	neogenin 1	NI	32	1.075901	0.812098	0.856027	1.046027	0.679023	4.146651	85.253	2.21E-06	3.49E-05	8
7108_at	7108	ENSG0000(	TM7SF2	transmembrane 7 supe	NI	34	0.86482	0.814503	1.028289	1.112265	0.657782	4.982123	30.73237	8.48E-05	0.000496	8
284611_at	284611	ENSG0000(	FAM102B	family with sequence s	NI	32	0.871343	0.907383	0.864261	1.089293	0.528786	4.340075	35.06968	5.34E-05	0.000346	8
439921_at	439921	ENSG0000(	MXRA7	matrix-remodelling ass	I	22	0.93529	1.296233	1.497147	1.346901	0.993754	5.707048	119.1354	6.53E-07	1.58E-05	8
3159_at	3159	ENSG0000(	HMGA1	high mobility group AT	I	7	0.993573	1.350267	1.309647	1.286594	0.933198	7.814612	98.61169	1.30E-06	2.41E-05	8
54206_at	54206	ENSG0000(	ERRFI1	ERBB receptor feedbac	I	11	1.050981	1.224849	1.251851	1.341336	1.061395	12.04653	131.9662	4.49E-07	1.23E-05	8
1471_at	1471	ENSG0000(	CST3	cystatin C Secreted	I	20	0.966274	1.145412	1.322764	1.371454	1.058561	10.21393	174.0538	1.63E-07	6.81E-06	8
84329_at	84329	ENSG0000(	HVCN1	hydrogen v ion channe	NI	20	0.778591	1.415058	1.404479	1.239333	0.991876	4.51006	109.1923	8.98E-07	1.96E-05	8
10903_at	10903	ENSG0000(	MTMR11	myotubularin related p	NI	39	0.699715	1.407373	1.187759	1.256988	0.989413	4.945266	62.91331	6.65E-06	7.40E-05	8
8601_at	8601	ENSG0000(	RGS20	regulator o Secreted	I	11	0.883577	1.073608	1.449721	1.160109	0.825327	6.145658	72.49737	3.98E-06	5.17E-05	8
3918_at	3918	ENSG0000(	LAMC2	laminin, ga Secreted	I	76	0.89804	0.86011	1.42204	1.078756	0.813248	4.583698	76.62062	3.26E-06	4.52E-05	8
55568_at	55568	ENSG0000(	GALNT10	UDP-N-ace Secreted	I	11	1.069175	0.886484	1.361543	1.174363	0.70436	6.732627	16.01975	0.000781	0.002932	8
8841_at	8841	ENSG0000(	HDAC3	histone deacetylase 3	I	11	0.788874	0.792135	1.392239	1.323051	0.8915	6.066011	82.8823	2.45E-06	3.70E-05	8
8991_at	8991	ENSG0000(	SELENBP1	selenium binding prote	NI	9	0.877978	0.910182	1.25298	1.392478	0.604374	3.803676	21.35869	0.000298	0.001332	8
26269_at	26269	ENSG0000(	FBXO8	F-box protein 8	I	11	0.582717	0.86666	1.18952	0.838953	0.835682	7.736022	57.90663	8.96E-06	9.13E-05	8
26511_at	26511	ENSG0000(	CHIC2	cysteine-rich hydrophc	I	7	0.553353	0.76637	1.233724	0.856317	0.84866	8.715238	14.02767	0.001207	0.004208	8
28971_at	28971	ENSG0000(	C11orf67	chromosome 11 open	I	13	0.45782	0.803124	1.139601	0.794427	0.962675	7.486113	52.2354	1.30E-05	0.000119	8
51780_at	51780	ENSG0000(	KDM3B	lysine (K)-specific dem	I	22	0.472168	0.720468	1.252691	0.921374	1.058256	5.741163	73.45289	3.80E-06	5.00E-05	8
6940_at	6940	ENSG0000(	ZNF354A	zinc finger protein 354	NI	11	0.553195	0.936112	1.071748	0.761222	1.081002	6.235702	50.60721	1.45E-05	0.00013	8
4801_at	4801	ENSG0000(	NFYB	nuclear transcription fa	NI	27	0.535264	0.904857	0.973973	0.745171	1.051778	5.674727	59.77164	7.99E-06	8.39E-05	8
84312_at	84312	ENSG0000(	BRMS1L	breast cancer metasta:	I	36	0.388612	0.872954	1.023804	0.852668	1.098404	5.452659	75.00297	3.52E-06	4.76E-05	8
81928_at	81928	ENSG0000(	CABLES2	Cdk5 and Abl enzyme s	I	11	0.254965	1.010774	1.188145	1.046616	1.071232	6.394097	96.98809	1.38E-06	2.52E-05	8
57178_at	57178	ENSG0000(	ZMIZ1	zinc finger, Secreted	I	21	0.163452	0.809076	1.06017	0.91138	1.106647	7.573713	51.99665	1.32E-05	0.000121	8
145567_at	145567	ENSG0000(	TTC7B	tetratricopeptide repe	NI	8	0.379864	0.820597	0.939412	1.050005	0.763953	6.646534	14.75678	0.001023	0.003677	8
129685_at	129685	ENSG0000(	TAF8	TAF8 RNA polymerase	NI	21	0.368843	0.722321	0.751734	1.030842	0.898056	5.409424	32.1656	7.23E-05	0.000439	8
285343_at	285343	ENSG0000(	C3orf23	chromosome 3 open re	NI	44	0.402817	1.049234	0.825112	0.880078	0.844729	5.735845	62.89703	6.65E-06	7.40E-05	8
118487_at	118487	ENSG0000(	CHCHD1	coiled-coil-helix-coiled	I	9	0.506625	0.801281	1.058632	0.782779	0.613092	10.40166	73.43101	3.80E-06	5.00E-05	8

1875_at	1875	ENSG0000(	E2F5	E2F transcription facto	I	11	0.455338	0.844183	1.047857	0.683999	0.641868	7.989118	33.13165	6.52E-05	0.000404	8
7097_at	7097	ENSG0000(	TLR2	toll-like receptor 2	NI	32	0.464338	0.901499	1.085178	0.844944	0.529292	3.767187	70.24784	4.46E-06	5.58E-05	8
27344_at	27344	ENSG0000(	PCSK1N	proprotein Secreted	NI	16	0.550283	0.726584	1.167492	0.916488	0.431825	4.164328	41.83307	2.86E-05	0.000216	8
122970_at	122970	ENSG0000(	ACOT4	acyl-CoA thioesterase	NI	11	0.522404	0.579844	1.127499	0.845406	0.523161	3.464576	12.92236	0.001574	0.005265	8
80741_at	80741	NA	LY6G5C	lymphocyt Secreted	NI	11	0.513558	0.776386	1.326996	0.923074	0.638689	3.783658	20.7054	0.000331	0.001453	8
4041_at	4041	ENSG0000(	LRP5	low density lipoprotein	NI	10	0.424483	0.799966	1.388186	0.996398	0.542186	4.735077	32.08088	7.30E-05	0.000442	8
6414_at	6414	ENSG0000(	SEPP1	selenoprot Secreted	I	22	0.581511	0.629045	1.405355	1.22262	1.286198	3.769952	102.8349	1.12E-06	2.22E-05	8
51170_at	51170	ENSG0000(	HSD17B11	hydroxyste Secreted	I	22	0.472397	0.600326	1.311586	1.186829	1.473944	9.147424	62.5397	6.79E-06	7.51E-05	8
3149_at	3149	ENSG0000(	HMGB3	high mobility group bo	I	10	0.288743	0.864507	1.254175	1.345246	1.245113	6.11588	20.20995	0.000359	0.001552	8
650_at	650	ENSG0000(	BMP2	bone morp Secreted	I	22	0.424507	0.550609	1.539031	1.411315	1.174134	4.411916	47.96108	1.76E-05	0.00015	8
57584_at	57584	ENSG0000(	ARHGAP21	Rho GTPase activating	I	5	0.462746	0.47473	1.315974	1.237048	1.022573	5.741697	38.18842	3.95E-05	0.000276	8
23234_at	23234	ENSG0000(	DNAJC9	DnaJ (Hsp40) homolog	I	4	0.590496	0.548863	0.794126	1.090106	1.041497	8.571886	86.72355	2.08E-06	3.33E-05	8
4008_at	4008	ENSG0000(	LMO7	LIM domain 7	NI	32	0.430629	0.584405	0.954422	1.041598	0.983351	5.824448	48.26771	1.72E-05	0.000148	8
84190_at	84190	ENSG0000(	C12orf26	chromosome 12 open	NI	6	0.484118	0.374764	0.910638	1.223447	0.915229	4.636475	15.10339	0.000948	0.003445	8
79582_at	79582	ENSG0000(	SPAG16	sperm associated antig	NI	21	0.954815	0.579977	1.083532	1.224891	0.835664	4.002083	51.59303	1.36E-05	0.000123	8
6916_at	6916	ENSG0000(	TBXAS1	thromboxa Secreted	NI	23	0.549847	0.700089	1.008506	1.276873	0.710613	4.400403	68.77092	4.82E-06	5.89E-05	8
9748_at	9748	ENSG0000(	SLK	STE20-like kinase	I	33	0.812945	0.742807	1.031641	1.088927	1.121695	6.733046	98.94109	1.29E-06	2.40E-05	8
79682_at	79682	ENSG0000(	MLF1IP	MLF1 interacting prote	I	19	0.821491	0.71899	0.954503	1.054358	1.159746	7.987418	30.80051	8.42E-05	0.000493	8
2739_at	2739	ENSG0000(	GLO1	glyoxalase I	I	41	0.791127	0.786533	1.014246	1.045348	1.201078	9.459718	62.41747	6.84E-06	7.55E-05	8
54764_at	54764	ENSG0000(	ZRANB1	zinc finger, RAN-bindir	I	44	0.766736	0.903397	0.995833	1.006515	1.209499	5.334592	96.18155	1.43E-06	2.57E-05	8
6605_at	6605	ENSG0000(	SMARCE1	SWI/SNF related, matr	I	43	0.710831	0.813748	1.08916	1.023307	1.241753	8.485699	67.0873	5.27E-06	6.27E-05	8
5782_at	5782	ENSG0000(	PTPN12	protein tyr phosphopr	I	30	0.885708	0.922859	0.998287	1.198333	1.24786	9.489418	116.9549	6.98E-07	1.64E-05	8
23358_at	23358	ENSG0000(	USP24	ubiquitin s peptidase	I	22	0.810294	0.957114	1.048782	1.157462	1.223202	7.371991	61.09555	7.39E-06	7.95E-05	8
6760_at	6760	ENSG0000(	SS18	synovial sarcoma trans	I	64	0.901893	0.977795	1.083655	1.02737	1.258596	7.945815	124.552	5.55E-07	1.42E-05	8
8562_at	8562	ENSG0000(	DENR	density-regulated prot	I	16	0.88756	0.944008	0.88539	1.073313	1.159416	9.830083	126.9206	5.18E-07	1.35E-05	8
51167_at	51167	ENSG0000(	CYB5R4	cytochrome b5 reducta	I	10	0.839285	0.795984	0.907458	1.001605	1.21932	7.919025	49.63792	1.56E-05	0.000137	8
23255_at	23255	ENSG0000(	CCDC165	coiled-coil domain con	I	6	0.795435	0.74325	0.771597	1.031119	1.306923	7.910423	44.14529	2.36E-05	0.000186	8
54876_at	54876	ENSG0000(	DCAF16	DDB1 and CUL4 associ	I	44	0.766859	0.816441	0.904302	1.012759	1.398676	6.737875	82.09505	2.54E-06	3.80E-05	8
23001_at	23001	ENSG0000(	WDFY3	WD repeat and FYVE d	I	63	0.940941	0.868303	1.090493	0.863103	1.241774	5.646595	70.93895	4.31E-06	5.43E-05	8
63979_at	63979	ENSG0000(	FIGNL1	fidgetin-like 1	I	11	0.9625	0.89787	1.06131	0.789361	1.135044	7.745009	54.14607	1.14E-05	0.000109	8
1946_at	1946	ENSG0000(	EFNA5	ephrin-A5 Secreted	I	33	1.169418	0.837526	1.099415	0.937565	1.14017	5.565907	107.237	9.59E-07	2.03E-05	8
5412_at	5412	ENSG0000(	UBL3	ubiquitin-like 3	I	30	0.997776	0.636292	1.060521	0.88	1.2598	6.878054	31.8759	7.47E-05	0.000449	8
4628_at	4628	ENSG0000(	MYH10	myosin, heavy chain 1(	I	29	1.047834	0.702342	1.057251	0.784552	1.114139	5.046559	48.33602	1.71E-05	0.000148	8
90355_at	90355	ENSG0000(	C5orf30	chromosome 5 open r	I	11	1.021961	0.745121	1.158136	1.008001	1.485292	6.420973	54.69906	1.10E-05	0.000106	8
55206_at	55206	ENSG0000(	SBNO1	strawberry notch hom	I	39	0.937127	0.70259	1.0907	0.840379	1.453252	7.117531	74.41182	3.62E-06	4.85E-05	8
57381_at	57381	ENSG0000(	RHOJ	ras homolc GTPase	I	29	1.044983	0.705194	0.995826	1.276698	1.412436	5.769563	27.37972	0.000127	0.000678	8
4306_at	4306	ENSG0000(	NR3C2	nuclear rec nuclear rec	I	22	1.029066	0.668202	1.077809	1.337921	1.181885	3.721503	19.67235	0.000393	0.001675	8
116984_at	116984	ENSG0000(	ARAP2	ArfGAP with RhoGAP c	NI	21	1.035586	0.82517	0.838073	1.102478	1.169221	3.515428	83.32885	2.40E-06	3.67E-05	8
100113407	1E+08	ENSG0000(	TMEM170	transmembrane protei	I	11	1.131607	0.670749	0.79631	1.126341	1.142158	4.828794	27.29752	0.000128	0.000683	8
25945_at	25945	ENSG0000(	PVRL3	poliovirus receptor-rel	I	11	1.106158	0.99106	0.892585	1.122071	1.481486	8.744256	117.5929	6.85E-07	1.62E-05	8
23052_at	23052	ENSG0000(	ENDOD1	endonuclease domain	I	22	1.170181	0.875087	0.959282	1.115693	1.337837	6.755804	49.56126	1.57E-05	0.000138	8
153642_at	153642	ENSG0000(	ARSK	arylsulfatase family, m	I	30	1.089233	0.88918	0.732393	1.255692	1.455686	5.200865	90.06352	1.81E-06	3.03E-05	8
154007_at	154007	ENSG0000(	SNRNP48	small nuclear ribonucl	NI	33	0.66378	0.504569	0.873532	0.737594	1.193572	5.742704	49.31624	1.59E-05	0.00014	8
6198_at	6198	ENSG0000(	RPS6KB1	ribosomal kinase	NI	23	0.630254	0.562579	0.939323	0.697893	1.108706	6.216373	58.2537	8.77E-06	8.97E-05	8
148534_at	148534	ENSG0000(	TMEM56	transmembrane protei	NI	33	0.595329	0.640346	0.918487	0.715574	1.235079	4.610901	19.41662	0.000411	0.001735	8
7443_at	7443	ENSG0000(	VRK1	vaccinia rel kinase	I	9	0.61978	0.502514	1.021656	0.806398	1.199403	8.537943	19.7208	0.00039	0.001664	8
29957_at	29957	ENSG0000(	SLC25A24	solute carri Secreted	I	22	0.580127	0.501276	0.944358	0.856988	1.175996	6.653699	58.98327	8.38E-06	8.69E-05	8
11103_at	11103	ENSG0000(	KRR1	KRR1, small subunit (S	I	41	0.741583	0.71148	0.887324	0.893228	1.139782	7.77699	82.05337	2.54E-06	3.81E-05	8
58478_at	58478	ENSG0000(	ENOPH1	enolase-phosphatase	I	19	0.660226	0.766178	0.883593	0.898257	1.123832	8.303288	98.63158	1.30E-06	2.41E-05	8
27246_at	27246	ENSG0000(	RNF115	ring finger protein 115	I	11	0.731944	0.647766	0.866549	0.858049	1.013488	7.301512	77.31543	3.15E-06	4.42E-05	8
373_at	373	ENSG0000(	TRIM23	tripartite m GTPase	NI	49	0.69555	0.630204	0.912381	0.832714	1.026792	5.006481	80.5509	2.72E-06	3.99E-05	8
5501_at	5501	ENSG0000(	PPP1CC	protein ph phosphopr	I	33	0.553458	0.553819	0.820376	0.872407	1.022169	11.06969	46.61347	1.95E-05	0.000161	8
9787_at	9787	ENSG0000(	DLGAP5	discs, large phosphopr	I	20	0.477465	0.608169	0.819039	0.836264	1.05327	9.64841	62.81094	6.68E-06	7.43E-05	8
5494_at	5494	ENSG0000(	PPM1A	protein ph phosphopr	NI	54	0.562851	0.585984	0.859796	0.810506	1.103869	5.790615	67.24447	5.23E-06	6.24E-05	8
5912_at	5912	ENSG0000(	RAP2B	RAP2B, member of RA	NI	31	0.580045	0.489702	0.80245	0.761663	1.083764	4.938989	43.91983	2.41E-05	0.000189	8
80829_at	80829	NA	ZFP91	zinc finger protein 91 h	I	21	0.596008	0.587031	0.707345	0.743856	1.028382	8.346303	59.9643	7.90E-06	8.32E-05	8
154043_at	154043	ENSG0000(	CNKSR3	CNKSR family member	I	11	0.698523	0.432197	0.67319	0.84469	1.093424	6.967408	19.48739	0.000406	0.001717	8
10520_at	10520	ENSG0000(	ZNF211	zinc finger protein 211	NI	22	0.672766	0.680481	1.044537	0.840692	1.290096	4.209515	55.28243	1.06E-05	0.000103	8
3895_at	3895	ENSG0000(	KTN1	kinectin 1 (kinesin rece	I	89	0.651255	0.581326	1.045876	0.801469	1.429112	9.27302	81.714	2.58E-06	3.85E-05	8
80311_at	80311	ENSG0000(	KLHL15	kelch-like 15 (Drosoph	I	11	0.775058	0.587823	0.959097	0.687803	1.4454	6.425049	46.61245	1.95E-05	0.000161	8
84162_at	84162	ENSG0000(	KIAA1109	KIAA1109	I	32	0.57159	0.371704	1.093918	0.730902	1.319739	6.10369	55.48988	1.04E-05	0.000102	8
64332_at	64332	ENSG0000(	NFKBIZ	nuclear factor of kappa	I	11	0.6805	0.303223	1.021629	0.748213	1.303454	8.578746	10.19622	0.003327	0.009876	8
595_at	595	ENSG0000(	CCND1	cyclin D1	I	43	0.583554	0.764856	0.795327	0.839889	1.466919	8.433646	63.33099	6.49E-06	7.27E-05	8
8905_at	8905	ENSG0000(	AP1S2	adaptor-related protei	I	18	0.53988	0.640019	0.681876	0.994218	1.297384	9.610337	42.09266	2.80E-05	0.000212	8
79666_at	79666	ENSG0000(	PLEKHF2	pleckstrin homology d	I	22	0.432135	0.582665	0.83958	1.242766	1.379773	5.214511	53.49747	1.19E-05	0.000112	8
2627_at	2627	ENSG0000(	GATA6	GATA binding protein	I	11	0.93828	0.498049	1.253688	0.9466	0.976209	7.772491	41.42241	2.97E-05	0.000222	8
10929_at	10929	NA	SRSF8	serine/arginine-rich sp	I	11	0.815654	0.553741	1.179751	0.994131	0.971645	6.824803	22.92819	0.000234	0.001099	8
5715_at	5715	NA	PSMD9	proteasome (prosome	I	21	0.748918	0.719205	1.22187	0.927677	0.937093	6.637945	75.98477	3.36E-06	4.63E-05	8
57531_at	57531	ENSG0000(	HACE1	HECT domain and anky	NI	32	0.732311	0.635265	1.330941	0.884147	0.849848	5.007385	41.6726	2.90E-05	0.000218	8
201895_at	201895	ENSG0000(	C4orf34	chromosome 4 open r	I	20	0.618838	0.442411	1.460213	0.989239	1.123334	7.576505	118.6395	6.63E-07	1.59E-05	8
2706_at	2706	ENSG0000(	GJB2	gap junction protein, b	NI	33	0.519685	0.357151	1.350472	0.884041	0.900899	3.810338	47.78874	1.78E-05	0.000152	8
23012_at	23012	ENSG0000(	STK38L	serine/thre kinase	NI	44	0.770483	0.730405	1.550599	1.008067	0.776312	4.887788	106.5931	9.80E-07	2.04E-05	8
2517_at	2517	ENSG0000(	FUCA1	fucosidase, Secreted	I	26	0.699968	0.792666	1.514587	0.985425	0.782446	7.562869	110.5242	8.59E-07	1.89E-05	8
79899_at	79899	ENSG0000(	PRR5L	proline rich 5 like	NI	22	0.705657	0.533097	1.381902	1.025322	0.696844	4.201874	31.80893	7.52E-05	0.000451	8
84460_at	84460	ENSG0000(	ZMAT1	zinc finger, matrin-typ	NI	21	0.882803	0.568756	1.416059	0.765386	0.786785	3.641517	40.99508	3.08E-05	0.000228	8
4065_at	4065	NA	LY75	lymphocyt Secreted	NI	11	0.474546	0.13854	1.737304	0.990039	1.053363	3.774089	16.55974	0.0007	0.002685	8
80007_at	80007	ENSG0000(	C10orf88	chromosome 10 open	NI	11	0.56633	0.059654	1.461908	0.797985	0.866364	6.097548	42.3527	2.74E-05	0.000209	8
79400_at	79400	NA	NOX5	NADPH oxi ion channe	I	28	0.504145	0.544306	1.778235	0.754134	1.183977	4.506099	11.30259	0.002411	0.007511	8
9023_at	9023	ENSG0000(	CH25H	cholesterol 25-hydroxy	NI	11	0.643492	0.513549	1.672348	0.574441	1.232291	4.191668	41.9938	2.82E-05	0.000214	8
56913_at	56913	ENSG0000(	C1GALT1	core 1 synt Secreted	I	8	0.932495	0.677544	1.74107	0.896665	1.108861	7.227459	29.04622	0.000103	0.00058	8
57728_at	57728	ENSG0000(	WDR19	WD repeat domain 19	NI	9	0.721822	1.01492	1.971783	0.638658	1.168169	5.752861	71.05967	4.28E-06	5.42E-05	8
51186_at	51186	ENSG0000(	WBP5	WW domain binding p	I	33	0.21031	0.388451	0.896391	0.768331	1.243232	9.998948	36.99425	4.42E-05	0.0003	8

4673_at	4673	ENSG0000(	NAP1L1	nucleosome assembly		I	14	0.250401	0.312352	0.98787	0.768802	1.18575	9.237735	39.96881	3.37E-05	0.000244	8
55681_at	55681	ENSG0000(	SCYL2	SCY1-like 2	kinase	I	25	0.307419	0.375136	0.88439	0.980822	1.144875	6.995242	48.05417	1.75E-05	0.00015	8
140862_at	140862	ENSG0000(	ISM1	isthmin 1 homolog (ze		NI	11	0.304685	0.414002	0.806159	0.761327	1.328285	3.596823	21.57284	0.000288	0.001296	8
2531_at	2531	ENSG0000(	KDSR	3-ketodihy	Secreted	I	32	0.395682	0.303071	0.826292	0.778059	1.002327	7.064127	20.70044	0.000331	0.001454	8
25831_at	25831	ENSG0000(	HECTD1	HECT domain containi		I	10	0.415883	0.501026	1.047241	0.802249	0.960711	8.165829	48.85312	1.65E-05	0.000143	8
6133_at	6133	ENSG0000(	RPL9	ribosomal protein L9		I	5	0.334392	0.435709	1.068405	0.848134	1.014706	12.08913	45.56196	2.11E-05	0.000171	8
1176_at	1176	ENSG0000(	AP3S1	adaptor-re	transporte	I	5	0.378368	0.438982	1.109269	0.907608	1.176205	10.87937	35.26918	5.24E-05	0.000342	8
55900_at	55900	ENSG0000(	ZNF302	zinc finger protein 302		I	16	0.302395	0.632388	0.856307	0.902554	1.113018	7.055509	78.24581	3.02E-06	4.30E-05	8
130502_at	130502	ENSG0000(	TTC32	tetratricopeptide repe		I	10	0.051692	0.515924	0.843211	0.835333	1.176965	8.004991	23.85509	0.000204	0.000984	8
901_at	901	ENSG0000(	CCNG2	cyclin G2		NI	30	0.124534	0.364739	0.87986	0.857221	1.109736	4.617028	49.69126	1.55E-05	0.000137	8
414149_at	414149	ENSG0000(	ACBD7	acyl-CoA binding doma		NI	11	0.082063	0.506177	1.01137	1.056619	1.175791	5.102758	13.88936	0.001246	0.004319	8
10253_at	10253	ENSG0000(	SPRY2	sprouty homolog 2 (Dr		I	11	0.221506	0.464066	1.149889	0.962218	0.974378	9.748525	13.28824	0.001438	0.004867	8
644_at	644	ENSG0000(	BLVRA	biliverdin reductase A		I	10	0.279382	0.339166	1.206916	0.894019	0.897617	8.404819	76.44845	3.29E-06	4.55E-05	8
9306_at	9306	ENSG0000(	SOCS6	suppressor of cytokine		NI	16	0.214069	0.329915	1.36265	1.071501	1.10707	5.322998	17.71935	0.000559	0.002232	8
100127888	1E+08	NA	LOC100127	hypothetical LOC10012		I	11	-0.04439	0.300505	1.060747	1.171167	1.47075	9.076528	15.96761	0.000789	0.002958	8
8325_at	8325	ENSG0000(	FZD8	frizzled fan	G-protein c	I	22	0.088829	0.244493	0.884923	1.228472	1.15529	7.612844	40.8472	3.12E-05	0.00023	8
60592_at	60592	ENSG0000(	SCOC	short coiled-coil protei		I	35	0.481093	0.585745	1.41986	0.937416	1.293655	8.347423	83.08817	2.43E-06	3.69E-05	8
6102_at	6102	ENSG0000(	RP2	retinitis pig	kinase	I	11	0.400729	0.370344	1.409261	0.82628	1.330839	8.789207	38.16119	3.96E-05	0.000276	8
594839_at	594839	NA	SNORA33	small nucleolar RNA, H		I	4	0.598987	0.465249	1.372346	0.651486	1.443192	6.622689	10.6111	0.002939	0.008871	8
9975_at	9975	ENSG0000(	NR1D2	nuclear rec	nuclear rec	I	43	0.646679	0.753896	1.33612	0.907419	1.638252	5.51883	43.09574	2.58E-05	0.0002	8
10198_at	10198	ENSG0000(	MPHOSPH	M-phase phosphoprot		NI	11	0.420514	0.599412	1.367347	0.824893	1.555016	6.04871	60.1583	7.81E-06	8.24E-05	8
57393_at	57393	ENSG0000(	TMEM27	transmemt	peptidase	I	11	0.504682	0.23385	1.412928	1.320066	1.610058	3.636973	15.10878	0.000947	0.003443	8
394_at	394	ENSG0000(	ARHGAP5	Rho GTPas	GTPase	I	59	0.675907	0.698853	1.906924	1.403951	1.88549	7.318054	112.5966	8.02E-07	1.81E-05	8
10276_at	10276	ENSG0000(	NET1	neuroepithelial cell tra		I	25	0.681218	0.601169	1.58693	1.292022	1.851119	8.043476	72.20113	4.04E-06	5.21E-05	8
6659_at	6659	ENSG0000(	SOX4	SRY (sex determining r		I	77	0.702383	0.650461	1.50591	1.732297	2.088661	6.309963	47.53493	1.82E-05	0.000154	8
55432_at	55432	ENSG0000(	YOD1	YOD1 OTU	peptidase	I	7	0.531049	0.538064	1.223032	1.147289	2.261932	6.44271	44.5165	2.30E-05	0.000182	8
84108_at	84108	ENSG0000(	PCGF6	polycomb group ring fi		NI	3	0.857426	0.348202	2.15735	1.241395	2.223744	5.259863	13.33055	0.001424	0.004824	8
222223_at	222223	ENSG0000(	KIAA1324L	KIAA1324-like		I	11	0.605378	1.266465	2.04095	1.573273	1.143637	4.330306	235.4311	5.34E-08	3.72E-06	8
6947_at	6947	ENSG0000(	TCN1	transcobal	Secreted	I	9	0.923892	1.025547	2.09663	1.356376	0.865291	4.576862	52.94981	1.24E-05	0.000115	8
22882_at	22882	ENSG0000(	ZHX2	zinc fingers and homec		I	11	0.709261	0.984547	1.685303	1.453417	0.885253	5.728928	16.63667	0.000689	0.002649	8
25925_at	25925	ENSG0000(	ZNF521	zinc finger protein 521		I	9	0.325853	1.024507	1.821371	1.385826	0.883417	4.602708	28.4802	0.000111	0.00061	8
2353_at	2353	ENSG0000(	FOS	FBJ murine osteosarco		I	44	-0.15602	0.810223	2.403398	1.835104	1.297062	4.709096	39.50516	3.51E-05	0.000252	8
152002_at	152002	ENSG0000(	C3orf21	chromosor	Secreted	NI	22	1.089527	1.166288	0.911712	0.785768	0.72036	4.743049	58.83036	8.46E-06	8.74E-05	8
3084_at	3084	ENSG0000(	NRG1	neuregulin	Secreted	NI	84	1.122053	1.174768	0.821739	0.684345	0.708492	4.434182	82.98951	2.44E-06	3.69E-05	8
79814_at	79814	ENSG0000(	AGMAT	agmatine ureohydrola		NI	21	1.187557	1.076298	0.95562	0.728271	0.712839	3.983287	75.0457	3.51E-06	4.75E-05	8
51019_at	51019	ENSG0000(	CCDC53	coiled-coil domain con		I	5	1.149891	0.986281	0.918175	0.762439	0.65887	9.485507	68.54527	4.88E-06	5.93E-05	8
2769_at	2769	ENSG0000(	GNA15	guanine nu	GTPase	I	30	1.049338	1.065759	1.00095	0.881127	0.666828	4.251222	84.61908	2.27E-06	3.56E-05	8
23070_at	23070	ENSG0000(	FTSJD2	FtsJ methyltransferase		I	21	1.001324	1.069433	0.968826	0.846121	0.646359	6.147875	41.55975	2.93E-05	0.000219	8
114548_at	114548	ENSG0000(	NLRP3	NLR family, pyrin doma		NI	21	1.088793	0.949979	0.886792	0.811815	0.525149	4.17545	37.5303	4.21E-05	0.000289	8
79875_at	79875	ENSG0000(	THSD4	thrombosp	peptidase	NI	11	1.105327	1.222744	0.794952	0.862767	0.838444	5.519308	102.9351	1.11E-06	2.22E-05	8
58533_at	58533	ENSG0000(	SNX6	sorting nexin 6		I	16	1.106577	1.069609	0.80615	0.948142	0.859405	9.155514	99.0454	1.28E-06	2.40E-05	8
90480_at	90480	ENSG0000(	GADD45GI	growth arr	Secreted	I	11	1.052892	1.137292	0.718794	1.020856	0.724947	10.27159	95.35717	1.47E-06	2.62E-05	8
144406_at	144406	ENSG0000(	WDR66	WD repeat domain 66		I	21	1.150657	1.062474	0.721227	0.887859	0.683199	3.934045	51.62625	1.35E-05	0.000123	8
57630_at	57630	ENSG0000(	SH3RF1	SH3 domain containing		NI	32	1.121487	0.967329	0.716863	0.817891	0.721771	6.400687	60.59982	7.61E-06	8.08E-05	8
11108_at	11108	ENSG0000(	PRDM4	PR domain containing		I	21	1.2617	0.948858	0.819494	0.862888	0.78774	7.791941	70.98592	4.30E-06	5.42E-05	8
129293_at	129293	ENSG0000(	C2orf89	chromosome 2 open re		NI	10	1.257126	0.858984	0.728651	0.970888	0.452933	4.211566	73.88621	3.72E-06	4.93E-05	8
148641_at	148641	ENSG0000(	SLC35F3	solute carrier family 35		NI	11	1.312272	0.826535	0.56611	1.006589	0.537839	3.69858	83.88209	2.35E-06	3.63E-05	8
55778_at	55778	ENSG0000(	ZNF839	zinc finger protein 839		NI	29	1.223211	0.75708	0.640429	0.832813	0.418834	4.391408	60.82681	7.50E-06	8.01E-05	8
145407_at	145407	ENSG0000(	C14orf37	chromosome 14 open		NI	20	1.117214	0.745527	0.70969	1.017313	0.602331	4.154308	29.82181	9.42E-05	0.000539	8
51063_at	51063	ENSG0000(	CALHM2	calcium homeostasis n		NI	11	1.114565	0.701355	0.666638	0.848584	0.559738	4.629613	24.39251	0.000189	0.000925	8
9860_at	9860	ENSG0000(	LRIG2	leucine-rich repeats an		NI	9	1.273817	0.744483	0.790556	0.606468	0.690483	3.925371	27.69345	0.000122	0.000658	8
5563_at	5563	ENSG0000(	PRKAA2	protein kin	kinase	NI	44	1.154113	0.873074	0.755585	0.53234	0.789364	3.766927	64.90243	5.94E-06	6.85E-05	8
23304_at	23304	ENSG0000(	UBR2	ubiquitin protein ligase		I	19	1.14483	0.679369	0.957037	0.692104	0.656556	5.671112	75.85889	3.38E-06	4.64E-05	8
24149_at	24149	ENSG0000(	ZNF318	zinc finger protein 318		I	22	1.096979	0.625509	0.836711	0.528672	0.683424	6.589187	56.08261	1.00E-05	9.92E-05	8
11227_at	11227	ENSG0000(	GALNT5	UDP-N-ace	Secreted	NI	10	1.001754	0.857988	0.69024	0.791773	0.685961	6.221671	24.4758	0.000187	0.000916	8
412_at	412	ENSG0000(	STS	steroid sulfatase (micr		NI	42	1.019709	0.744703	0.670839	0.690556	0.81057	4.200273	52.62027	1.26E-05	0.000117	8
55823_at	55823	NA	VPS11	vacuolar protein sortin		NI	11	1.025009	0.877514	1.108597	0.673572	0.542505	6.865168	48.9209	1.64E-05	0.000143	8
64757_at	64757	ENSG0000(	MOSC1	MOCO sulf	Secreted	NI	11	1.060886	0.871816	1.010605	0.526733	0.617404	3.852234	28.00862	0.000117	0.000638	8
50855_at	50855	ENSG0000(	PARD6A	par-6 partitioning defe		NI	11	1.018956	0.941963	0.955013	0.624073	0.299191	4.670986	15.93507	0.000795	0.002976	8
207063_at	207063	ENSG0000(	DHRSX	dehydrogenase/reduct		NI	21	1.10038	0.885394	0.624805	0.748747	0.568683	4.517609	65.15571	5.86E-06	6.79E-05	8
10160_at	10160	ENSG0000(	FARP1	FERM, RhoGEF (ARHGI		NI	43	1.06528	0.9703	0.545208	0.68053	0.545552	4.016717	54.25053	1.13E-05	0.000108	8
54958_at	54958	ENSG0000(	TMEM160	transmembrane protei		I	8	1.080166	1.114293	0.622867	0.770226	0.507628	8.516888	79.10499	2.90E-06	4.17E-05	8
57194_at	57194	ENSG0000(	ATP10A	ATPase, cla	transporte	NI	13	1.030165	0.913418	0.701248	0.634527	0.437687	3.946048	37.64751	4.16E-05	0.000286	8
9703_at	9703	ENSG0000(	KIAA0100	KIAA0100		I	33	0.952386	1.009207	0.648079	0.562182	0.509745	8.163151	48.88185	1.64E-05	0.000143	8
65095_at	65095	ENSG0000(	KRI1	KRI1 homolog (S. cerev		I	32	1.011327	0.884179	0.493076	0.63075	0.373214	7.846908	63.69709	6.35E-06	7.17E-05	8
10435_at	10435	ENSG0000(	CDC42EP2	CDC42 effector proteir		NI	11	1.131521	1.119109	0.546527	0.474028	0.425309	5.609294	45.65464	2.10E-05	0.00017	8
283229_at	283229	ENSG0000(	EFCAB4A	EF-hand calcium bindir		NI	11	1.016826	1.130632	0.506528	0.397276	0.537262	3.621823	24.50858	0.000186	0.000913	8
64787_at	64787	ENSG0000(	EPS8L2	EPS8-like 2		NI	33	1.294012	0.904393	0.770278	0.518432	0.51819	4.711581	37.36074	4.27E-05	0.000292	8
9627_at	9627	ENSG0000(	SNCAIP	synuclein, alpha intera		NI	53	1.524988	0.862966	0.548279	0.591089	0.469497	3.901036	74.92839	3.53E-06	4.77E-05	8
125476_at	125476	ENSG0000(	INO80C	INO80 complex subuni		I	9	1.005565	0.928297	0.634284	0.571542	0.132495	9.11906	24.478	0.000187	0.000916	8
10691_at	10691	ENSG0000(	GMEB1	glucocorticoid modula		NI	11	1.035933	0.896141	0.569731	0.544914	0.046001	5.470714	30.2375	8.98E-05	0.000518	8
22907_at	22907	ENSG0000(	DHX30	DEAH (Asp	Secreted	I	17	1.088598	0.809436	0.633042	0.47555	0.153754	8.500696	47.07381	1.88E-05	0.000157	8
9052_at	9052	ENSG0000(	GPRC5A	G protein-c	G-protein c	I	22	1.064039	0.866279	0.812674	0.447402	0.030602	5.316901	22.89112	0.000235	0.001103	8
56603_at	56603	ENSG0000(	CYP26B1	cytochrome P450, fam		NI	21	1.131055	0.758803	0.746414	0.639455	0.302688	3.945697	29.06433	0.000103	0.000579	8
54538_at	54538	ENSG0000(	ROBO4	roundabout homolog 4		I	32	1.129666	0.678848	0.635115	0.55061	0.257687	8.254105	47.75234	1.79E-05	0.000152	8
145781_at	145781	NA	GCOM1	GRINL1A c	Secreted	NI	20	1.034527	0.619139	0.635433	0.763596	0.144982	3.905714	39.14029	3.62E-05	0.000258	8
3592_at	3592	ENSG0000(	IL12A	interleukin	Secreted	NI	11	1.090975	0.886088	0.853227	0.838523	0.046618	4.246757	31.59593	7.70E-05	0.00046	8
101_at	101	NA	ADAM8	ADAM met	peptidase	NI	39	1.059228	0.645732	0.87877	0.797942	0.297561	4.385634	41.00653	3.07E-05	0.000228	8
3624_at	3624	ENSG0000(	INHBA	inhibin, be	Secreted	NI	22	1.181379	0.950333	0.543675	0.903607	0.40558	4.589489	28.44149	0.000111	0.000612	8
65982_at	65982	ENSG0000(	ZSCAN18	zinc finger and SCAN d		NI	38	1.193064	0.897053	0.603288	0.843225	0.328981	4.692743	82.42675	2.50E-06	3.77E-05	8
64374_at	64374	ENSG0000(	SIL1	SIL1 homol	Secreted	I	11	1.22115	1.108709	0.591984	0.850161	0.355101	6.775515	57.39741	9.25E-06	9.32E-05	8
55129_at	55129	ENSG0000(	ANO10	anoctamin 10		NI	11	1.160446	1.166224	0.791273	0.773613	0.156569	5.738124	30.31112	8.90E-05	0.000515	8

9896_at	9896	ENSG0000(	FIG4	FIG4 homolog, SAC1 li		I	10	1.339713	1.125626	0.559476	0.852038	0.162485	7.976578	47.01404	1.89E-05	0.000158	8
387893_at	387893	ENSG0000(	SETD8	SET domain containing		I	9	0.882339	1.171771	0.641837	0.950874	0.688793	6.339448	59.11427	8.32E-06	8.63E-05	8
493856_at	493856	ENSG0000(	CISD2	CDGSH iron sulfur dom		I	11	0.858824	1.109061	0.659923	0.910309	0.629045	9.078166	65.31648	5.80E-06	6.74E-05	8
2687_at	2687	ENSG0000(	GGT5	gamma-glu	Secreted	NI	19	0.802869	1.202653	0.568861	1.010215	0.604391	4.070337	38.84606	3.72E-05	0.000264	8
4794_at	4794	ENSG0000(	NFKBIE	nuclear factor of kappa		I	11	0.916956	1.267661	0.78271	1.04393	0.677815	6.960431	69.36913	4.67E-06	5.75E-05	8
894_at	894	ENSG0000(	CCND2	cyclin D2		NI	31	0.851768	1.251794	0.729963	0.946649	0.554366	3.920719	41.18328	3.03E-05	0.000226	8
79683_at	79683	ENSG0000(	ZDHHC14	zinc finger, DHHC-type		NI	22	1.119564	1.20725	0.81342	0.939704	0.536372	4.728542	29.92505	9.31E-05	0.000534	8
162461_at	162461	ENSG0000(	TMEM92	transmembrane protei		NI	18	0.99861	1.174222	0.699091	1.065298	0.376158	4.435424	18.89294	0.000451	0.001874	8
6510_at	6510	ENSG0000(	SLC1A5	solute carri	Secreted	I	30	0.808396	1.450538	0.879987	0.94167	0.490715	6.696109	76.79065	3.23E-06	4.50E-05	8
137075_at	137075	ENSG0000(	CLDN23	claudin 23		NI	11	0.777586	1.359991	0.81166	0.893545	0.530177	3.610802	18.89672	0.00045	0.001873	8
126308_at	126308	ENSG0000(	MOBKL2A	MOB1, Mps One Binde		I	11	0.837097	1.470054	0.669117	0.965382	0.609905	6.319541	27.01327	0.000133	0.000703	8
11174_at	11174	ENSG0000(	ADAMTS6	ADAM met peptidase		NI	20	1.237157	1.576489	0.682013	0.879855	0.67777	4.576065	52.72063	1.25E-05	0.000116	8
2526_at	2526	ENSG0000(	FUT4	fucosyltransferase 4 (a		NI	22	1.027972	1.537911	0.608991	0.78854	0.641347	4.187135	42.86131	2.63E-05	0.000203	8
56977_at	56977	ENSG0000(	STOX2	storkhead box 2		NI	11	1.12041	1.460615	0.405935	0.89029	0.412202	4.068824	42.12607	2.79E-05	0.000212	8
81490_at	81490	ENSG0000(	PTDSS2	phosphatidylserine syr		NI	22	0.90254	1.238056	0.344588	0.879451	0.647063	5.765476	31.99643	7.37E-05	0.000445	8
84769_at	84769	ENSG0000(	MPV17L2	MPV17 mit	Secreted	I	11	0.848675	1.227108	0.389805	0.907636	0.54083	7.343949	53.0575	1.23E-05	0.000115	8
10538_at	10538	ENSG0000(	BATF	basic leucine zipper tra		NI	11	0.695473	1.465398	0.431356	1.049551	0.637834	3.970245	46.89012	1.91E-05	0.000159	8
8550_at	8550	ENSG0000(	MAPKAPK5	mitogen-ac	kinase	I	9	0.859703	1.036234	0.899396	0.891666	0.860415	7.070714	70.09021	4.50E-06	5.61E-05	8
84934_at	84934	ENSG0000(	C12orf52	chromosome 12 open		I	11	0.877892	1.018276	0.838386	0.922208	0.796156	7.460889	75.81256	3.39E-06	4.65E-05	8
9265_at	9265	ENSG0000(	CYTH3	cytohesin 3		I	21	0.925562	1.098692	0.906234	0.855977	0.770773	4.359916	75.39863	3.45E-06	4.70E-05	8
1026_at	1026	ENSG0000(	CDKN1A	cyclin-depe	kinase	I	22	0.804059	1.165643	0.935593	0.900903	0.660055	8.478794	94.59361	1.52E-06	2.68E-05	8
8798_at	8798	ENSG0000(	DYRK4	dual-specif	kinase	I	11	0.867273	1.136307	0.794891	0.892313	0.718039	7.733048	60.90282	7.47E-06	7.99E-05	8
84866_at	84866	ENSG0000(	TMEM25	transmemb	Secreted	NI	41	0.787426	1.108444	0.828731	0.669251	0.840438	4.235106	32.0082	7.36E-05	0.000444	8
6397_at	6397	ENSG0000(	SEC14L1	SEC14-like	transporte	I	48	0.805256	1.24157	0.706905	0.822311	0.617196	6.962724	68.31754	4.94E-06	5.99E-05	8
5425_at	5425	ENSG0000(	POLD2	polymerase (DNA direc		I	17	0.794796	1.146131	0.664536	0.756804	0.549929	9.12209	53.04819	1.23E-05	0.000115	8
347252_at	347252	ENSG0000(	IGFBPL1	insulin-like growth fact		NI	10	0.766507	1.289024	0.766396	0.709404	0.664837	3.929798	10.49115	0.003045	0.009151	8
221545_at	221545	NA	C6orf136	chromosome 6 open re		I	11	0.931206	1.111807	0.76562	0.681598	0.693958	8.406181	61.02795	7.42E-06	7.97E-05	8
152519_at	152519	ENSG0000(	NIPAL1	NIPA-like domain cont		NI	11	0.97285	1.080125	0.695792	0.72719	0.629402	3.881799	15.12314	0.000944	0.003434	8
5271_at	5271	ENSG0000(	SERPINB8	serpin peptidase inhibi		I	40	0.784105	1.136714	1.083332	0.854078	0.480749	5.780243	45.04213	2.20E-05	0.000177	8
54985_at	54985	ENSG0000(	HCFC1R1	host cell factor C1 regu		I	31	0.76866	1.096602	0.990524	0.841889	0.535871	8.341149	22.98114	0.000232	0.001091	8
388722_at	388722	ENSG0000(	C1orf53	chromosome 1 open re		NI	9	0.913371	1.19403	1.019921	0.639277	0.5192	6.233412	46.75431	1.93E-05	0.00016	8
23479_at	23479	ENSG0000(	ISCU	iron-sulfur cluster scaf		I	21	0.803347	1.009064	0.782122	0.91731	0.853392	10.24332	83.6595	2.37E-06	3.65E-05	8
26524_at	26524	ENSG0000(	LATS2	LATS, large	kinase	I	33	0.800032	1.075787	0.74644	0.90838	0.830706	5.793355	54.0237	1.15E-05	0.000109	8
5037_at	5037	ENSG0000(	PEBP1	phosphatidylethanolar		I	7	0.827142	1.077976	0.749599	0.98627	0.831361	9.702214	91.82986	1.69E-06	2.89E-05	8
55751_at	55751	ENSG0000(	TMEM184(	transmembrane protei		I	29	0.741248	1.010992	0.799802	0.845163	0.786126	7.190738	80.33656	2.74E-06	4.01E-05	8
131965_at	131965	ENSG0000(	METTL6	methyltransferase like		NI	43	0.771377	1.002172	0.708035	0.829673	0.833448	6.04006	61.5466	7.19E-06	7.79E-05	8
57118_at	57118	ENSG0000(	CAMK1D	calcium/ca	kinase	NI	33	0.824126	1.022822	0.736303	0.850838	0.734801	4.046288	75.19536	3.49E-06	4.73E-05	8
55086_at	55086	ENSG0000(	CXorf57	chromosome X open re		NI	32	0.665169	1.085023	0.801935	1.014141	0.684869	3.795611	77.3467	3.15E-06	4.41E-05	8
50854_at	50854	NA	C6orf48	chromosor	Secreted	I	21	0.669352	1.033472	0.714874	0.928671	0.717166	11.50443	50.68874	1.44E-05	0.000129	8
6166_at	6166	ENSG0000(	RPL36AL	ribosomal protein L36a		I	8	0.749661	1.047879	0.574082	0.855903	0.706875	10.68159	71.8289	4.12E-06	5.28E-05	8
51678_at	51678	ENSG0000(	MPP6	membrane protein, pa		NI	9	0.671852	1.030378	0.525154	0.792887	0.659398	7.104652	37.79285	4.10E-05	0.000284	8
59269_at	59269	ENSG0000(	HIVEP3	human immunodeficie		NI	44	0.648395	1.052738	0.597814	0.709521	0.778989	6.357693	30.79899	8.42E-05	0.000493	8
4218_at	4218	ENSG0000(	RAB8A	RAB8A, member RAS o		I	11	0.76906	1.028357	0.714852	0.843515	0.638348	9.299495	67.57324	5.13E-06	6.15E-05	8
79036_at	79036	ENSG0000(	C19orf50	chromosome 19 open		I	22	0.739219	1.083835	0.681574	0.816241	0.573705	8.992064	74.42375	3.62E-06	4.85E-05	8
10312_at	10312	ENSG0000(	TCIRG1	T-cell, imm	transporte	I	21	0.895441	1.077326	0.621833	0.795121	0.395926	7.230874	68.13836	4.98E-06	6.03E-05	8
51161_at	51161	ENSG0000(	C3orf18	chromosome 3 open re		NI	11	0.845796	1.019706	0.602424	0.731732	0.433435	3.912669	15.00404	0.000969	0.003514	8
6520_at	6520	ENSG0000(	SLC3A2	solute carri	transporte	I	73	0.886855	1.009218	0.599907	0.837979	0.471246	7.877641	30.92656	8.30E-05	0.000488	8
1627_at	1627	ENSG0000(	DBN1	drebrin 1		I	31	0.81208	1.045212	0.498277	0.839282	0.517113	6.729588	50.23576	1.49E-05	0.000133	8
51293_at	51293	ENSG0000(	CD320	CD320 mol	Secreted	I	10	0.781661	1.129551	0.649381	0.958379	0.444325	7.771208	35.4715	5.13E-05	0.000336	8
10211_at	10211	NA	FLOT1	flotillin 1		I	54	0.806539	1.174336	0.64658	0.789118	0.353878	8.673456	47.19949	1.86E-05	0.000156	8
6301_at	6301	ENSG0000(	SARS	seryl-tRNA synthetase		I	10	0.653615	1.172042	0.582774	0.88384	0.282657	10.3188	47.20942	1.86E-05	0.000156	8
255488_at	255488	ENSG0000(	RNF144B	ring finger protein 144		NI	33	0.592134	1.140197	0.501622	0.818654	0.338942	4.736552	27.85555	0.000119	0.000647	8
55374_at	55374	ENSG0000(	TMCO6	transmembrane and co		NI	19	0.865141	1.040728	0.823078	0.769233	0.298691	5.184948	33.43992	6.31E-05	0.000394	8
85450_at	85450	ENSG0000(	ITPRIP	inositol 1,4,5-trisphosp		NI	11	0.778786	1.03911	0.878937	0.768825	0.282229	5.497489	45.48799	2.13E-05	0.000172	8
211_at	211	ENSG0000(	ALAS1	aminolevulinate, delta		I	43	0.753822	1.033969	0.752846	0.757468	0.401515	7.768736	28.92739	0.000105	0.000586	8
53635_at	53635	ENSG0000(	PTOV1	prostate tumor overex		I	25	0.701854	1.091425	0.849754	0.991478	0.413025	7.816362	51.36717	1.38E-05	0.000125	8
10809_at	10809	ENSG0000(	STARD10	StAR-related lipid trans		NI	12	0.465204	0.964142	0.820659	1.176954	0.300408	5.346796	48.05446	1.75E-05	0.00015	8
79611_at	79611	ENSG0000(	ACSS3	acyl-CoA synthetase sh		NI	21	0.582529	0.832097	0.829369	1.150834	0.320817	3.981202	44.57811	2.28E-05	0.000181	8
79776_at	79776	ENSG0000(	ZFHX4	zinc finger homeobox 4		NI	33	0.660025	0.998335	0.809801	0.997648	1.068596	3.893377	38.9295	3.69E-05	0.000262	8
220930_at	220930	NA	ZEB1-AS1	ZEB1 antisense RNA 1		NI	21	0.664157	0.804099	0.748439	0.937016	1.127511	3.76773	33.29012	6.41E-05	0.000399	8
10983_at	10983	ENSG0000(	CCNI	cyclin I		I	42	0.510917	0.879311	0.670915	0.875793	1.025592	10.29364	66.60478	5.41E-06	6.42E-05	8
84992_at	84992	NA	PIGY	phosphatidylinositol gl		I	16	0.784425	0.875185	0.745136	0.780814	1.017068	9.13354	86.41544	2.11E-06	3.36E-05	8
1062_at	1062	ENSG0000(	CENPE	centromere protein E,		I	11	0.675792	0.778911	0.79309	0.820174	1.028431	8.276433	44.9708	2.21E-05	0.000178	8
9351_at	9351	ENSG0000(	SLC9A3R2	solute carrier family 9		I	11	0.802297	0.704962	0.592427	1.147584	0.80215	4.194708	70.49756	4.41E-06	5.52E-05	8
7746_at	7746	ENSG0000(	ZNF193	zinc finger protein 193		I	11	0.817386	0.712261	0.679861	1.017113	0.696339	7.145693	82.90224	2.45E-06	3.70E-05	8
5570_at	5570	ENSG0000(	PKIB	protein kin	kinase	NI	55	0.592418	0.706647	0.491866	1.00584	0.799629	3.553592	55.44702	1.05E-05	0.000102	8
3998_at	3998	ENSG0000(	LMAN1	lectin, mannose-bindir		NI	50	0.666217	0.812056	0.610955	0.774917	1.063745	6.989233	33.94516	5.99E-05	0.000379	8
55188_at	55188	ENSG0000(	RIC8B	resistance to inhibitors		NI	22	0.582264	0.729798	0.497406	0.682959	1.078574	6.901126	32.84293	6.73E-05	0.000414	8
2983_at	2983	ENSG0000(	GUCY1B3	guanylate cyclase 1, so		NI	20	0.723163	1.009644	0.408983	0.585655	1.012456	3.971915	28.44913	0.000111	0.000612	8
6624_at	6624	ENSG0000(	FSCN1	fascin homolog 1, actir		NI	24	0.57279	1.050443	0.421634	0.805825	0.887955	4.233089	36.15122	4.80E-05	0.000319	8
1470_at	1470	ENSG0000(	CST2	cystatin SA	Secreted	I	10	1.17378	1.104775	0.383375	1.2461	0.751143	6.777729	62.55039	6.79E-06	7.51E-05	8
142891_at	142891	ENSG0000(	SAMD8	sterile alpha motif don		NI	11	0.979061	1.131455	0.100369	1.27483	1.038594	4.896688	114.7733	7.48E-07	1.72E-05	8
4147_at	4147	ENSG0000(	MATN2	matrilin 2	Secreted	I	11	1.475407	0.653734	0.284933	0.907245	1.097784	4.668683	85.41204	2.20E-06	3.48E-05	8
8835_at	8835	ENSG0000(	SOCS2	suppressor of cytokine		I	19	0.87722	0.586832	0.409873	0.925861	1.42936	5.341019	89.67374	1.84E-06	3.05E-05	8
23057_at	23057	ENSG0000(	NMNAT2	nicotinamide nucleotic		NI	43	1.228955	0.586148	0.667027	0.552501	0.547059	5.247213	63.39248	6.47E-06	7.25E-05	8
5021_at	5021	ENSG0000(	OXTR	oxytocin re	G-protein c	NI	22	1.257247	0.406725	0.504622	0.652473	0.684932	4.033109	57.88084	8.97E-06	9.13E-05	8
9480_at	9480	ENSG0000(	ONECUT2	one cut homeobox 2		NI	22	1.152858	0.824815	0.246178	0.665483	0.802722	3.93635	55.92138	1.02E-05	9.99E-05	8
9699_at	9699	ENSG0000(	RIMS2	regulating synaptic me		NI	33	1.150814	0.551335	0.259778	0.686899	0.590266	3.639573	63.30439	6.50E-06	7.27E-05	8
10013224(	1E+08	NA	LOC100132	hypothetical LOC1001		NI	11	1.044357	0.623866	0.425626	0.420667	0.454385	3.704673	34.51346	5.65E-05	0.000362	8
10844_at	10844	ENSG0000(	TUBGCP2	tubulin, gamma compl		NI	26	1.050323	0.572518	0.577263	0.448161	0.204875	5.17596	10.67266	0.002886	0.008735	8
89782_at	89782	ENSG0000(	LMLN	leishmanol peptidase		NI	11	1.159314	0.326456	0.318383	0.462525	0.356326	6.649094	15.35065	0.000899	0.0033	8
25858_at	25858	ENSG0000(	C11orf20	chromosome 11 open		NI	10	1.204358	0.697109	0.17806	0.673846	0.06407	4.267289	30.69959	8.52E-05	0.000497	8

578_at	578	ENSG0000(	BAK1	BCL2-antagonist/killer	NI	8	1.047315	0.493929	0.099642	0.683072	0.064622	5.753731	13.0169	0.001537	0.005156	8
652995_at	652995	NA	UCA1	urothelial cancer assoc	NI	5	1.721722	0.412742	0.370136	0.155522	0.603274	3.830317	50.86994	1.43E-05	0.000128	8
100288778	1E+08	NA	LOC10028	WAS protein family ho	NI	5	1.25942	0.031583	0.620037	1.137564	0.417301	4.076454	19.6675	0.000394	0.001676	8
51069_at	51069	ENSG0000(	MRPL2	mitochond Secreted	I	18	0.625755	1.029738	0.985825	0.62584	0.39064	6.122038	66.51588	5.44E-06	6.44E-05	8
55558_at	55558	ENSG0000(	PLXNA3	plexin A3 Secreted	NI	11	0.510374	1.160654	0.976211	0.692068	0.117322	5.114982	71.87201	4.11E-06	5.27E-05	8
11007_at	11007	ENSG0000(	CCDC85B	coiled-coil domain con	I	22	0.623247	0.952951	1.050784	0.943015	0.228617	6.935931	58.78558	8.48E-06	8.75E-05	8
6236_at	6236	ENSG0000(	RRAD	Ras-relatec GTPase	NI	11	0.4627	1.196743	1.428328	0.759125	0.311771	4.420549	31.68114	7.63E-05	0.000457	8
728392_at	728392	NA	LOC728392	hypothetical protein L(	NI	11	0.824211	1.405955	0.911551	0.978806	0.145722	4.993993	44.05657	2.38E-05	0.000187	8
113878_at	113878	ENSG0000(	DTX2	deltex homolog 2 (Dro	NI	18	0.922717	1.217467	1.068736	0.520545	-0.04303	4.837049	31.50335	7.78E-05	0.000464	8
79703_at	79703	ENSG0000(	C11orf80	chromosome 11 open	NI	9	0.691627	0.690212	1.729459	1.107114	0.341581	4.630579	35.89041	4.92E-05	0.000325	8
139201_at	139201	NA	LOC139201	mitogen-activated pro	NI	4	0.603336	0.48982	1.70625	1.08725	0.341874	5.223548	49.54948	1.57E-05	0.000138	8
151313_at	151313	ENSG0000(	FAHD2B	fumarylacetoacetate h	NI	7	0.790334	0.914099	1.335231	1.390162	0.129796	3.873476	40.35704	3.25E-05	0.000238	8
2043_at	2043	ENSG0000(	EPHA4	EPH recept kinase	NI	42	1.219671	0.421868	1.188787	0.503261	0.247243	4.790277	28.5688	0.000109	0.000605	8
4864_at	4864	ENSG0000(	NPC1	Niemann-P transporte	I	20	1.094772	0.702065	1.117925	0.358162	0.218514	6.350675	27.38314	0.000127	0.000678	8
81619_at	81619	ENSG0000(	TSPAN14	tetraspanin 14	I	43	1.028081	0.655644	0.850855	0.370803	0.402334	6.637224	38.81762	3.73E-05	0.000264	8
7079_at	7079	ENSG0000(	TIMP4	TIMP meta Secreted	NI	11	1.054915	0.487164	0.911295	0.181996	0.359303	3.980535	19.49228	0.000406	0.001716	8
3855_at	3855	ENSG0000(	KRT7	keratin 7	I	10	1.062307	0.558351	1.145677	0.072887	0.08545	8.886964	13.34913	0.001417	0.004804	8
1308_at	1308	ENSG0000(	COL17A1	collagen, ty Secreted	NI	22	1.114736	0.634909	0.915199	0.155169	0.037096	5.171849	17.13715	0.000625	0.00244	8
27290_at	27290	ENSG0000(	SPINK4	serine pept Secreted	NI	8	1.130313	0.845893	1.508076	0.684098	0.261572	4.212991	51.1647	1.40E-05	0.000126	8
677842_at	677842	NA	SNORA76	small nucleolar RNA, H	NI	3	0.67683	0.846052	1.594793	0.416647	0.139617	4.666349	18.75359	0.000462	0.001913	8
260294_at	260294	NA	NSUN5P2	NOP2/Sun domain fam	NI	7	0.836519	0.851532	1.572074	0.322113	0.812938	4.29188	40.97169	3.08E-05	0.000228	8
55616_at	55616	ENSG0000(	ASAP3	ArfGAP with SH3 doma	NI	19	0.469716	1.060092	0.719004	0.715265	0.625814	4.461014	47.39874	1.84E-05	0.000155	8
124491_at	124491	ENSG0000(	TMEM170A	transmembrane protei	NI	11	0.536396	1.114202	0.596828	0.647309	0.495017	5.358825	15.21913	0.000925	0.003376	8
57161_at	57161	ENSG0000(	PELI2	pellino homolog 2 (Dro	NI	22	0.529496	1.076091	0.753297	0.79411	0.390396	3.983338	31.13351	8.11E-05	0.000479	8
1675_at	1675	ENSG0000(	CFD	complemer peptidase	NI	9	0.350186	1.007546	0.739406	0.564094	0.383655	3.677455	10.24679	0.003276	0.009752	8
26993_at	26993	ENSG0000(	AKAP8L	A kinase (PRKA) ancho	I	17	0.389386	1.324037	0.6519	0.87	0.370438	6.914347	52.72042	1.25E-05	0.000116	8
26284_at	26284	ENSG0000(	ERAL1	Era G-protein-like 1 (E.	NI	11	0.384448	1.310868	0.782073	0.612061	0.263165	5.360421	25.38514	0.000165	0.000829	8
4357_at	4357	ENSG0000(	MPST	mercaptopyruvate sulf	NI	11	0.708188	1.102817	0.61659	0.482013	0.392334	6.865203	51.2787	1.39E-05	0.000125	8
112812_at	112812	ENSG0000(	FDX1L	ferredoxin 1-like	I	11	0.511393	1.064665	0.530676	0.538305	0.297161	7.661885	33.67847	6.16E-05	0.000387	8
51296_at	51296	ENSG0000(	SLC15A3	solute carri transporte	NI	9	0.589356	1.25973	0.50661	0.36513	0.391399	4.323738	38.9203	3.70E-05	0.000262	8
79192_at	79192	ENSG0000(	IRX1	iroquois homeobox 1	NI	10	0.430995	1.157425	0.791646	0.365321	0.145229	3.77373	10.95957	0.002656	0.008155	8
89944_at	89944	ENSG0000(	GLB1L2	galactosidase, beta 1-li	NI	17	0.961683	1.122936	0.384933	0.628287	0.520878	5.091989	20.46371	0.000344	0.0015	8
10645_at	10645	ENSG0000(	CAMKK2	calcium/ca kinase	NI	40	0.782296	1.06381	0.53999	0.641818	0.582738	6.301531	63.99115	6.25E-06	7.09E-05	8
84292_at	84292	ENSG0000(	WDR83	WD repeat domain 83	NI	9	0.788017	1.247897	0.446159	0.601883	0.744797	6.184037	28.55709	0.00011	0.000606	8
376267_at	376267	ENSG0000(	RAB15	RAB15, member RAS o	NI	11	0.989857	1.476181	0.720462	0.644433	0.480404	4.917655	30.67609	8.54E-05	0.000498	8
3887_at	3887	ENSG0000(	KRT81	keratin 81	I	11	0.945772	1.340621	0.778398	0.59985	0.439749	7.198801	10.33866	0.003187	0.009517	8
4803_at	4803	ENSG0000(	NGF	nerve grow Secreted	NI	11	0.979289	1.025908	0.685354	0.282022	0.36219	3.754947	11.95587	0.002018	0.006471	8
56548_at	56548	ENSG0000(	CHST7	carbohydrate (N-acety	I	11	0.815326	1.071131	0.738859	0.339744	0.158072	6.856355	13.4222	0.001393	0.004735	8
205_at	205	ENSG0000(	AK4	adenylate I kinase	I	3	0.761423	1.096621	0.914773	0.158967	0.418982	9.271945	20.96329	0.000317	0.001403	8
89849_at	89849	ENSG0000(	ATG16L2	ATG16 autophagy rela	NI	17	1.00113	1.039484	0.541566	0.765097	0.195941	4.544201	57.56762	9.15E-06	9.27E-05	8
11120_at	11120	ENSG0000(	BTN2A1	butyrophili Secreted	I	22	0.794688	1.014492	0.429734	0.773273	0.235366	6.727908	31.7651	7.56E-05	0.000453	8
349565_at	349565	ENSG0000(	NMNAT3	nicotinamide nucleotic	NI	11	0.823049	1.217726	0.467996	0.494086	0.002978	3.855988	44.48913	2.30E-05	0.000182	8
4598_at	4598	ENSG0000(	MVK	mevalonat kinase	I	20	0.798774	1.028085	0.324316	0.534949	0.280506	4.946494	13.55456	0.001349	0.004613	8
605_at	605	ENSG0000(	BCL7A	B-cell CLL/lymphoma 7	NI	27	0.708838	1.011847	-0.16145	0.626392	0.303428	3.97487	25.57305	0.000161	0.000813	8
29984_at	29984	ENSG0000(	RHOD	ras homolc GTPase	NI	11	0.493915	1.470876	0.439609	0.599565	-0.10826	5.00037	44.72379	2.26E-05	0.00018	8
441168_at	441168	ENSG0000(	FAM26F	family with sequence s	NI	21	0.407503	1.27687	0.310682	0.393437	0.004506	4.437394	43.23872	2.55E-05	0.000198	8
118738_at	118738	ENSG0000(	ZNF488	zinc finger protein 488	NI	11	0.602135	1.614694	0.319767	0.354487	0.4503	4.266254	25.2258	0.000168	0.000844	8
728215_at	728215	ENSG0000(	FAM155A	family with sequence s	NI	33	0.884213	1.0284	0.627554	0.116179	-0.20092	4.599849	12.04995	0.001968	0.006337	8
54920_at	54920	ENSG0000(	DUS2L	dihydrouridine synthas	NI	10	0.606993	1.058921	0.426135	0.388758	-0.31138	6.52833	38.07331	4.00E-05	0.000278	8
10848_at	10848	ENSG0000(	PPP1R13L	protein phosphatase 1	NI	11	0.067888	1.184003	0.49282	0.50753	0.380898	5.505199	24.59041	0.000184	0.000906	8
440_at	440	ENSG0000(	ASNS	asparagine synthetase	I	33	-0.17623	1.061524	0.378084	0.701268	0.307291	9.161038	11.48129	0.002295	0.007199	8
9828_at	9828	ENSG0000(	ARHGEF17	Rho guanine nucleotid	NI	11	0.159846	1.029667	0.273252	0.451903	0.127478	4.893242	21.27141	0.000302	0.001347	8
84892_at	84892	ENSG0000(	C3orf39	chromosome 3 open r	NI	11	0.354506	1.034363	0.411817	0.629681	0.503114	5.07273	28.2055	0.000114	0.000626	8
122953_at	122953	ENSG0000(	JDP2	Jun dimerization prote	NI	33	0.464946	1.175781	0.156217	0.675039	0.297771	3.864392	47.70798	1.79E-05	0.000152	8
51545_at	51545	ENSG0000(	ZNF581	zinc finger protein 581	NI	11	0.166615	1.188246	0.663064	0.636405	0.67098	7.075995	28.61703	0.000109	0.000603	8
7005_at	7005	ENSG0000(	TEAD3	TEA domain family me	NI	11	0.253548	1.068169	0.720676	0.49986	0.642348	5.289302	34.72096	5.53E-05	0.000356	8
133418_at	133418	ENSG0000(	EMB	embigin	NI	5	0.095465	1.064392	0.500249	0.203711	0.867118	3.426586	24.99859	0.000174	0.000866	8
1060_at	1060	ENSG0000(	CENPC1	centromere protein C	NI	15	0.190029	0.611789	1.010939	0.430864	0.351695	4.126372	12.29666	0.001845	0.006014	8
3872_at	3872	ENSG0000(	KRT17	keratin 17	NI	7	-0.10608	0.528137	1.051521	0.454047	0.25248	3.808753	16.00305	0.000784	0.00294	8
4693_at	4693	ENSG0000(	NDP	Norrie dise Secreted	I	11	0.260438	0.789446	1.208192	0.113513	-0.08666	6.381264	14.66362	0.001045	0.003735	8
26872_at	26872	ENSG0000(	STEAP1	six transme transporte	I	10	1.471949	1.238029	1.190312	1.117941	1.227107	8.163468	100.9624	1.19E-06	2.30E-05	8
10486_at	10486	ENSG0000(	CAP2	CAP, adenylate cyclase	I	60	1.380679	1.170026	1.299095	1.113336	1.14839	7.872251	81.66363	2.59E-06	3.85E-05	8
51099_at	51099	ENSG0000(	ABHD5	abhydrolas peptidase	I	66	1.323998	1.206739	1.174869	1.085489	1.294664	5.618461	110.3672	8.63E-07	1.90E-05	8
93973_at	93973	ENSG0000(	ACTR8	ARP8 actin-related pro	I	20	1.310049	1.251862	1.146296	1.187044	1.176971	5.975576	107.2294	9.59E-07	2.03E-05	8
51232_at	51232	ENSG0000(	CRIM1	cysteine ric kinase	I	30	1.304508	1.492506	1.282351	1.232797	1.310076	5.43321	83.29126	2.41E-06	3.67E-05	8
91574_at	91574	ENSG0000(	C12orf65	chromosome 12 open	I	22	1.108817	1.431161	1.128708	1.183968	1.341303	6.772852	89.87545	1.83E-06	3.04E-05	8
81849_at	81849	ENSG0000(	ST6GALNA(	ST6 (alpha- Secreted	I	43	1.535767	1.477115	1.084402	1.414145	1.324861	4.190687	149.395	2.85E-07	9.19E-06	8
51499_at	51499	ENSG0000(	TRIAP1	TP53 regulated inhibit	I	10	1.266478	1.416156	1.065307	1.416477	1.265861	9.475322	144.4827	3.22E-07	9.91E-06	8
6542_at	6542	ENSG0000(	SLC7A2	solute carri transporte	I	41	1.444867	1.127241	1.063805	1.435232	1.311241	4.306802	34.46814	5.68E-05	0.000363	8
55785_at	55785	ENSG0000(	FGD6	FYVE, RhoGEF and PH	I	22	1.739435	1.397194	1.40991	1.312531	1.485543	5.2291	97.51915	1.36E-06	2.49E-05	8
51209_at	51209	ENSG0000(	RAB9B	RAB9B, member RAS o	I	29	1.761961	1.331228	1.288948	1.208185	1.494591	4.698335	76.97521	3.20E-06	4.47E-05	8
79674_at	79674	ENSG0000(	VEPH1	ventricular zone expre	I	29	1.756921	1.651269	1.211711	1.165242	1.27874	4.154683	146.3249	3.07E-07	9.69E-06	8
7915_at	7915	ENSG0000(	ALDH5A1	aldehyde dehydrogena	I	21	1.44771	1.462708	1.384315	1.162648	0.986729	4.282953	83.095	2.43E-06	3.69E-05	8
6001_at	6001	ENSG0000(	RGS10	regulator of G-protein	I	10	1.482658	1.460132	1.247093	1.233424	0.990835	10.13866	192.9645	1.11E-07	5.45E-06	8
3914_at	3914	ENSG0000(	LAMB3	laminin, be Secreted	I	19	1.373275	1.297062	1.279267	1.318933	1.18488	8.333601	85.14021	2.22E-06	3.50E-05	8
91754_at	91754	ENSG0000(	NEK9	NIMA (nev kinase	I	31	1.438027	1.284649	1.224487	1.343034	1.070054	5.311044	92.96815	1.61E-06	2.81E-05	8
83987_at	83987	ENSG0000(	CCDC8	coiled-coil domain con	I	22	1.584257	1.590631	1.349392	1.359991	1.262646	4.522157	66.99565	5.30E-06	6.30E-05	8
9367_at	9367	ENSG0000(	RAB9A	RAB9A, me GTPase	I	9	1.53536	1.393378	1.503383	1.342394	1.107438	9.331838	75.49025	3.44E-06	4.69E-05	8
64420_at	64420	ENSG0000(	SUSD1	sushi domain containir	I	22	1.501815	1.595785	1.526349	1.513223	1.144053	4.719841	162.6323	2.09E-07	7.73E-06	8
2887_at	2887	ENSG0000(	GRB10	growth factor receptor	I	33	1.38021	1.541655	1.721638	1.405216	1.148132	4.174605	255.6389	3.94E-08	3.05E-06	8
5329_at	5329	ENSG0000(	PLAUR	plasminoge Secreted	I	50	1.365955	1.699608	1.427567	1.328179	1.046502	8.549161	189.1441	1.20E-07	5.63E-06	8
220213_at	220213	ENSG0000(	OTUD1	OTU domain containin	I	11	1.260481	1.539639	1.397815	1.318079	1.018671	8.466585	113.2348	7.86E-07	1.78E-05	8

137994_at	137994	ENSG0000(	LETM2	leucine zipper-EF-hanc	I	16	1.339824	1.853129	1.446408	1.438218	1.484193	4.828126	83.87956	2.35E-06	3.63E-05	8
23657_at	23657	ENSG0000(	SLC7A11	solute carri transporte	I	33	1.26741	1.790526	1.420312	1.516003	1.358898	7.333676	31.38376	7.89E-05	0.000469	8
122509_at	122509	ENSG0000(	IFI27L1	interferon, alpha-induc	I	11	1.129246	1.712741	1.309519	1.486065	1.48232	7.306162	80.61734	2.71E-06	3.99E-05	8
2535_at	2535	ENSG0000(	FZD2	frizzled fan G-protein c	I	11	1.161143	1.722375	1.317075	1.487347	1.402971	8.613382	170.4732	1.75E-07	7.02E-06	8
119016_at	119016	ENSG0000(	AGAP4	ArfGAP with GTPase dc	I	4	1.3761	1.801485	1.363375	1.293804	1.60554	6.910607	173.7343	1.64E-07	6.81E-06	8
119710_at	119710	ENSG0000(	C11orf74	chromosome 11 open	I	30	1.433683	1.720849	1.438466	1.402289	1.297404	6.461989	98.78952	1.29E-06	2.41E-05	8
9921_at	9921	ENSG0000(	RNF10	ring finger protein 10	I	28	1.397485	1.739179	1.432339	1.359182	1.233002	8.716979	180.5143	1.42E-07	6.21E-06	8
3219_at	3219	ENSG0000(	HOXB9	homeobox B9	I	11	1.216591	1.822582	1.49	1.379357	1.315468	7.239463	94.14043	1.54E-06	2.71E-05	8
50515_at	50515	ENSG0000(	CHST11	carbohydrate (chondrc	I	29	1.284497	1.677164	1.516493	1.421246	1.310883	8.34928	218.3914	7.05E-08	4.30E-06	8
10365_at	10365	ENSG0000(	KLF2	Kruppel-like factor 2 (l	I	21	1.015312	1.711929	1.156975	1.28536	1.642798	6.90162	164.9068	1.98E-07	7.48E-06	8
23554_at	23554	ENSG0000(	TSPAN12	tetraspanin 12	I	20	1.063341	1.48024	1.195336	1.336885	1.718057	4.801814	53.56559	1.18E-05	0.000112	8
116238_at	116238	ENSG0000(	TLCD1	TLC domain containing	NI	9	1.026315	1.842798	0.958193	1.238129	1.477832	5.402859	91.14965	1.73E-06	2.94E-05	8
2273_at	2273	ENSG0000(	FHL1	four and a half LIM dor	I	107	1.552607	1.771113	1.343043	1.57824	0.981004	4.51273	213.1666	7.71E-08	4.53E-06	8
55653_at	55653	ENSG0000(	BCAS4	breast carcinoma ampl	I	33	1.615856	1.780982	1.113334	1.476334	1.019774	5.843625	33.93406	6.00E-05	0.000379	8
3265_at	3265	ENSG0000(	HRAS	v-Ha-ras Harvey rat sar	I	22	1.38165	1.723418	1.059684	1.461952	0.827104	8.162227	136.9418	3.92E-07	1.12E-05	8
4128_at	4128	ENSG0000(	MAOA	monoamine oxidase A	I	44	1.536674	1.757959	1.217116	1.227439	1.111283	5.965449	186.1073	1.27E-07	5.87E-06	8
117854_at	117854	ENSG0000(	TRIM6	tripartite motif contair	I	22	1.466886	1.75542	1.132577	1.277505	1.066421	5.483167	95.57474	1.46E-06	2.61E-05	8
55030_at	55030	ENSG0000(	FBXO34	F-box protein 34	I	22	1.297218	1.822826	1.342123	1.246484	1.145094	6.950718	182.324	1.37E-07	6.17E-06	8
3382_at	3382	ENSG0000(	ICA1	islet cell autoantigen 1	I	43	1.214312	1.499919	1.237424	1.599499	0.954303	4.462143	196.7017	1.04E-07	5.27E-06	8
255738_at	255738	ENSG0000(	PCSK9	proprotein peptidase	I	19	1.009429	1.477431	1.228163	1.576628	0.960961	5.38741	139.9672	3.62E-07	1.06E-05	8
80212_at	80212	ENSG0000(	CCDC92	coiled-coil domain con	I	11	1.18788	1.553702	1.000255	1.697221	1.19594	6.811151	149.5948	2.83E-07	9.18E-06	8
11153_at	11153	ENSG0000(	FICD	FIC domain Secreted	I	11	1.049641	1.973589	1.146754	1.524794	1.27228	6.311941	52.48682	1.27E-05	0.000118	8
55201_at	55201	ENSG0000(	MAP1S	microtubule-associate	I	10	1.153662	1.94773	1.248795	1.488138	0.929082	6.971326	102.3077	1.14E-06	2.24E-05	8
1112_at	1112	ENSG0000(	FOXN3	forkhead b Secreted	I	18	1.627877	1.525477	1.166843	1.56533	1.832533	6.329269	122.4564	5.90E-07	1.48E-05	8
91392_at	91392	ENSG0000(	ZNF502	zinc finger protein 502	I	22	1.610283	1.445691	1.27853	1.656291	1.726188	4.340403	169.7368	1.78E-07	7.03E-06	8
7049_at	7049	ENSG0000(	TGFBR3	transformi Secreted	I	32	2.066871	1.348491	1.075129	1.516913	1.816365	5.607	194.2719	1.08E-07	5.36E-06	8
50617_at	50617	ENSG0000(	ATP6V0A4	ATPase, H+ transporte	I	18	1.796739	1.382344	0.963679	1.41407	1.301559	4.384593	81.43038	2.61E-06	3.88E-05	8
956_at	956	ENSG0000(	ENTPD3	ectonucleoside triphos	I	19	1.830226	1.173905	0.750869	1.336598	1.128248	4.453059	42.21108	2.77E-05	0.000211	8
375033_at	375033	ENSG0000(	PEAR1	platelet endothelial ag	I	11	1.592942	1.671362	0.766174	1.511234	1.119355	4.308543	77.55825	3.12E-06	4.39E-05	8
10103_at	10103	ENSG0000(	TSPAN1	tetraspanin 1	I	6	1.910515	1.905419	1.773081	1.162431	1.159002	7.32447	133.2295	4.33E-07	1.20E-05	8
2825_at	2825	ENSG0000(	GPR1	G protein-c G-protein c	I	11	1.684899	1.67684	1.619142	1.154393	1.077568	6.018129	69.80948	4.56E-06	5.66E-05	8
10687_at	10687	ENSG0000(	PNMA2	paraneoplastic antiger	I	43	1.886814	1.502311	1.714067	1.302232	1.346905	4.27189	164.9156	1.98E-07	7.48E-06	8
5999_at	5999	ENSG0000(	RGS4	regulator of G-protein	I	43	2.133563	1.828416	2.031435	1.196644	1.382181	5.024267	271.1859	3.17E-08	2.63E-06	8
2124_at	2124	ENSG0000(	EVI2B	ecotropic viral integrat	I	11	2.153125	1.511757	1.496575	1.265781	0.709823	6.107835	59.48834	8.13E-06	8.48E-05	8
56169_at	56169	ENSG0000(	GSDMC	gasdermin C	I	10	1.870265	1.614834	1.470548	1.183202	0.725243	4.192111	94.34203	1.53E-06	2.70E-05	8
347735_at	347735	ENSG0000(	SERINC2	serine incorporator 2	I	20	2.190633	2.050567	1.257114	0.906339	0.621684	4.786556	110.2544	8.66E-07	1.90E-05	8
2131_at	2131	ENSG0000(	EXT1	exostosin 1	I	10	1.543209	1.47486	1.154857	1.06255	0.75489	9.805739	179.6931	1.45E-07	6.27E-06	8
115098_at	115098	ENSG0000(	CCDC124	coiled-coil domain con	I	10	1.354143	1.420942	1.071727	1.138234	0.614676	7.084896	45.34934	2.15E-05	0.000174	8
126374_at	126374	ENSG0000(	WTIP	Wilms tumor 1 interac	NI	11	1.468754	1.508315	0.984346	0.870427	0.603287	5.362265	65.51318	5.74E-06	6.69E-05	8
9535_at	9535	ENSG0000(	GMFG	glia maturation factor,	NI	9	1.57318	1.628888	1.423851	1.150054	0.481612	4.501175	114.5283	7.54E-07	1.73E-05	8
55357_at	55357	ENSG0000(	TBC1D2	TBC1 domain family, m	I	11	1.451114	1.611751	1.537197	1.032729	0.610617	9.007911	61.7122	7.12E-06	7.75E-05	8
3170_at	3170	ENSG0000(	FOXA2	forkhead box A2	NI	11	0.984046	1.912022	0.960537	1.13959	0.65986	4.23334	122.9845	5.81E-07	1.47E-05	8
8291_at	8291	ENSG0000(	DYSF	dysferlin, limb girdle m	I	27	1.159368	1.756405	0.822769	1.314798	0.599488	5.194046	24.73787	0.00018	0.000891	8
83547_at	83547	ENSG0000(	RILP	Rab interacting lysosor	I	10	1.386102	2.192864	1.008921	1.26074	0.540758	5.798298	145.0375	3.18E-07	9.86E-06	8
3885_at	3885	ENSG0000(	KRT34	keratin 34 Secreted	I	11	1.115679	1.901303	0.968002	0.279503	0.35093	4.693146	92.27504	1.66E-06	2.85E-05	8
115265_at	115265	ENSG0000(	DDIT4L	DNA-damage-inducible	I	11	2.020758	1.640683	0.479735	1.386489	0.515454	4.525439	87.88022	1.98E-06	3.21E-05	8
57574_at	57574	ENSG0000(	4-Mar	membrane-associated	I	11	1.981645	1.801042	0.63597	0.648131	0.227888	5.810942	32.75695	6.79E-05	0.000416	8
10632_at	10632	ENSG0000(	ATP5L	ATP syntha transporte	I	26	0.73038	0.740643	1.071459	0.86545	0.975815	9.226964	72.62473	3.96E-06	5.14E-05	7
54521_at	54521	ENSG0000(	WDR44	WD repeat domain 44	I	53	0.763324	0.726474	1.028691	0.837372	0.920789	6.421242	52.34392	1.29E-05	0.000119	7
5868_at	5868	ENSG0000(	RAB5A	RAB5A, me GTPase	I	30	0.75223	0.643474	1.137662	0.822433	0.851692	8.275334	77.97562	3.06E-06	4.33E-05	7
10175_at	10175	ENSG0000(	CNIH	cornichon homolog (D	I	11	0.617348	0.625504	1.036916	0.998408	0.867271	10.34976	66.77491	5.36E-06	6.36E-05	7
65998_at	65998	NA	C11orf95	chromosome 11 open	I	10	0.668408	0.532404	0.989397	1.000908	0.892964	6.555081	27.41723	0.000126	0.000677	7
5128_at	5128	ENSG0000(	CDK17	cyclin-depe kinase	I	20	0.537171	0.61285	1.20135	0.895124	0.806029	4.505372	50.05313	1.51E-05	0.000134	7
169834_at	169834	NA	ZNF883	zinc finger protein 883	NI	15	0.540612	0.71753	1.025186	0.911628	0.803067	3.677092	33.60095	6.21E-05	0.000389	7
56898_at	56898	ENSG0000(	BDH2	3-hydroxybutyrate deh	NI	13	0.474912	0.61154	1.063228	0.772497	0.702072	6.006234	47.8429	1.78E-05	0.000151	7
9262_at	9262	ENSG0000(	STK17B	serine/thre kinase	NI	33	0.803625	0.664912	1.02921	0.738571	0.690075	5.116235	31.49944	7.78E-05	0.000464	7
23092_at	23092	ENSG0000(	ARHGAP26	Rho GTPase activating	NI	65	0.908948	0.698176	1.024301	0.60108	0.664612	4.603697	31.50304	7.78E-05	0.000464	7
2230_at	2230	ENSG0000(	FDX1	ferredoxin 1	NI	20	0.945368	0.503674	1.004879	0.64595	0.79672	5.561122	59.64977	8.05E-06	8.42E-05	7
8536_at	8536	ENSG0000(	CAMK1	calcium/ca kinase	NI	10	0.799441	0.820815	1.137337	0.565143	0.75786	4.770524	41.81508	2.87E-05	0.000216	7
51201_at	51201	ENSG0000(	ZDHHC2	zinc finger, DHHC-type	I	32	0.622248	0.724392	1.141605	0.582324	0.638084	6.98904	45.93534	2.05E-05	0.000167	7
7321_at	7321	ENSG0000(	UBE2D1	ubiquitin-conjugating (	I	22	0.505556	0.49003	1.140598	0.71506	0.854368	8.195096	47.16751	1.87E-05	0.000156	7
255520_at	255520	ENSG0000(	ELMOD2	ELMO/CED-12 domain	NI	19	0.490814	0.420383	1.130034	0.601655	0.953597	6.986278	43.81702	2.43E-05	0.00019	7
7320_at	7320	ENSG0000(	UBE2B	ubiquitin-conjugating (	I	30	0.638231	0.550395	1.006365	0.702485	0.8266	8.066513	23.15603	0.000226	0.00107	7
57692_at	57692	ENSG0000(	MAGEE1	melanoma antigen fan	NI	11	0.47771	0.58467	1.01059	0.517281	1.040912	5.434042	13.69419	0.001305	0.004493	7
23401_at	23401	ENSG0000(	FRAT2	frequently rearranged	NI	11	0.364378	0.564624	1.067908	0.610926	0.908961	4.985116	35.5235	5.10E-05	0.000335	7
221037_at	221037	ENSG0000(	JMJD1C	jumonji domain contai	I	43	0.743302	0.520134	0.91079	0.548901	1.005309	8.009173	56.00268	1.01E-05	9.96E-05	7
1385_at	1385	ENSG0000(	CREB1	cAMP responsive elem	NI	76	0.710661	0.380694	1.000253	0.506125	0.967076	5.240473	49.11433	1.62E-05	0.000141	7
114932_at	114932	ENSG0000(	MRFAP1L1	Morf4 family associate	I	11	0.609047	0.528631	1.211247	0.858103	1.044962	8.521673	80.40613	2.74E-06	4.01E-05	7
55769_at	55769	ENSG0000(	ZNF83	zinc finger protein 83	I	22	0.555875	0.457068	1.056986	0.83827	1.02683	7.771423	28.17874	0.000115	0.000628	7
54443_at	54443	ENSG0000(	ANLN	anillin, actin binding pı	I	42	0.57965	0.608857	1.082064	0.795591	1.093596	9.323699	70.49629	4.41E-06	5.52E-05	7
64343_at	64343	ENSG0000(	AZI2	5-azacytidi Secreted	I	32	0.555268	0.658863	1.037943	0.773006	0.998316	7.673398	57.45529	9.21E-06	9.30E-05	7
2677_at	2677	ENSG0000(	GGCX	gamma-glutamyl carbc	I	33	0.807552	0.589457	1.164506	0.720598	1.057117	5.993701	17.76255	0.000554	0.002216	7
120103_at	120103	ENSG0000(	SLC36A4	solute carri transporte	I	22	0.741259	0.443361	1.142016	0.690806	1.154518	8.154418	36.58236	4.60E-05	0.000309	7
56929_at	56929	ENSG0000(	FEM1C	fem-1 homolog c (C. el	NI	22	0.673282	0.472516	1.305238	0.660656	0.771518	6.04092	72.2073	4.04E-06	5.21E-05	7
51175_at	51175	ENSG0000(	TUBE1	tubulin, ep GTPase	I	10	0.640487	0.424897	1.264188	0.603372	0.820427	6.018612	47.71057	1.79E-05	0.000152	7
684959_at	684959	NA	SNORA25	small nucleolar RNA, H	NI	8	0.564311	0.281322	1.308998	0.59867	0.902223	6.388771	10.71002	0.002855	0.00865	7
79915_at	79915	ENSG0000(	ATAD5	ATPase family, AAA do	NI	10	0.558882	0.568193	1.360097	0.341975	0.999025	5.230247	38.18825	3.95E-05	0.000276	7
3833_at	3833	NA	KIFC1	kinesin family member	I	9	0.513986	0.642861	1.028744	0.568167	0.80789	7.532573	38.97791	3.68E-05	0.000261	7
2189_at	2189	ENSG0000(	FANCG	Fanconi an Secreted	NI	8	0.399725	0.689053	1.054889	0.524258	0.659669	6.205365	14.91193	0.000989	0.003573	7
163227_at	163227	NA	ZNF100	zinc finger protein 100	NI	10	0.47047	0.504591	1.153648	0.29143	0.710116	5.366538	16.96359	0.000646	0.00251	7
4144_at	4144	ENSG0000(	MAT2A	methionine adenosyltr	NI	29	0.511039	0.469105	1.02089	0.411002	0.589522	6.424342	30.79627	8.42E-05	0.000493	7
25842_at	25842	ENSG0000(	ASF1A	ASF1 anti-silencing fun	I	33	0.582683	0.451674	1.160681	0.711923	0.61048	7.59148	39.53942	3.50E-05	0.000251	7

254170_at	254170	ENSG00000	FBXO33	F-box protein 33		NI	22	0.541595	0.501642	1.280388	0.635589	0.502996	7.123388	14.7359	0.001028	0.003691	7
51533_at	51533	ENSG00000	PHF7	PHD finger protein 7		NI	11	0.754709	0.51071	1.228955	0.612751	0.606597	4.036122	40.34173	3.26E-05	0.000238	7
58526_at	58526	ENSG00000	MID1IP1	MID1 interacting prote		I	11	0.694967	0.710492	1.0256	0.75427	0.435425	8.469494	42.3653	2.74E-05	0.000209	7
64129_at	64129	ENSG00000	TINAGL1	tubulointei	peptidase	NI	20	0.659521	0.610017	1.09852	0.602469	0.337913	4.244264	30.07112	9.15E-05	0.000526	7
27345_at	27345	ENSG00000	KCNMB4	potassium	ion channe	NI	11	0.879858	0.462814	1.044784	0.59344	0.584543	3.858102	17.24275	0.000612	0.002402	7
79772_at	79772	ENSG00000	MCTP1	multiple C2 domains, t		NI	29	0.871729	0.359113	1.065457	0.546073	0.500023	5.392358	11.65105	0.00219	0.006925	7
4815_at	4815	ENSG00000	NINJ2	ninjurin 2		NI	11	0.855525	0.240636	1.187952	0.604301	0.385262	3.994607	28.7156	0.000107	0.000597	7
11169_at	11169	ENSG00000	WDHD1	WD repeat and HMG-b		NI	21	0.680487	-0.02176	1.071681	0.702071	1.190818	4.520032	63.16003	6.55E-06	7.32E-05	7
339005_at	339005	NA	WHAMMP:	WAS protein homolog		NI	5	0.875622	-0.11276	1.160215	0.441154	0.968269	3.960711	27.60659	0.000123	0.000664	7
1794_at	1794	ENSG00000	DOCK2	dedicator of cytokinesi		NI	11	1.005301	0.122859	0.838075	0.573972	0.762225	4.164154	54.45203	1.12E-05	0.000107	7
57721_at	57721	ENSG00000	METTL14	methyltransferase like		I	11	0.942855	0.628285	0.827537	0.653904	1.130391	6.774197	23.39102	0.000218	0.001039	7
4820_at	4820	ENSG00000	NKTR	natural killer-tumor re		I	31	0.823095	0.552221	0.850269	0.684234	1.07444	7.354308	57.39359	9.25E-06	9.32E-05	7
23362_at	23362	ENSG00000	PSD3	pleckstrin and Sec7 do		NI	54	0.862791	0.724553	0.758696	0.683224	1.268699	5.403331	48.91938	1.64E-05	0.000143	7
11056_at	11056	ENSG00000	DDX52	DEAD (Asp-Glu-Ala-As		NI	33	1.05048	0.696634	0.935201	0.659124	1.029435	4.955998	80.38012	2.74E-06	4.01E-05	7
57696_at	57696	ENSG00000	DDX55	DEAD (Asp-Glu-Ala-As		I	37	1.030209	0.792066	0.691656	0.757809	1.021405	6.822335	60.96715	7.44E-06	7.99E-05	7
3070_at	3070	ENSG00000	HELLS	helicase, lymphoid-spe		I	31	1.02309	0.399038	0.872029	0.535053	0.953979	5.823176	18.5567	0.000479	0.001969	7
338657_at	338657	ENSG00000	CCDC84	coiled-coil domain con		I	8	0.991553	0.421746	0.63256	0.695463	1.002908	7.595359	48.03752	1.75E-05	0.00015	7
57551_at	57551	ENSG00000	TAOK1	TAO kinase	kinase	NI	22	1.050454	0.63353	0.640943	0.409822	0.98287	4.868156	27.51797	0.000125	0.00067	7
9126_at	9126	ENSG00000	SMC3	structural maintenance		I	18	0.953402	0.400235	0.824244	0.280424	1.264624	8.433193	50.36786	1.48E-05	0.000131	7
10778_at	10778	NA	ZNF271	zinc finger protein 271		NI	32	0.787135	0.402992	0.663563	0.50582	1.036806	5.779952	56.85615	9.57E-06	9.59E-05	7
5529_at	5529	ENSG00000	PPP2R5E	protein phosphatase 2		I	21	0.792114	0.390132	0.705437	0.34227	1.13337	7.968433	17.09186	0.00063	0.002458	7
84296_at	84296	ENSG00000	GINS4	GINS complex subunit		I	24	0.686311	0.226771	0.647445	0.432627	1.124973	7.570422	51.7401	1.34E-05	0.000122	7
9497_at	9497	ENSG00000	SLC4A7	solute carri	transporte	I	20	0.607723	0.281921	0.676131	0.400794	1.124983	7.924021	28.08989	0.000116	0.000634	7
57563_at	57563	ENSG00000	KLHL8	kelch-like 8 (Drosophil		NI	33	0.703488	0.4979	0.678134	0.58197	1.267422	5.888826	42.65107	2.67E-05	0.000206	7
1829_at	1829	ENSG00000	DSG2	desmoglein 2		I	33	0.561359	0.358472	0.426174	0.54761	1.058516	7.099657	22.24775	0.000259	0.001191	7
92259_at	92259	NA	MRPS36	mitochondrial ribosom		NI	21	0.402985	0.479339	0.788404	0.655537	1.104437	5.725559	21.56924	0.000288	0.001297	7
10949_at	10949	ENSG00000	HNRNPA0	heterogeneous nuclea		I	21	0.368212	0.523999	0.850246	0.567201	1.019058	9.171184	47.70703	1.79E-05	0.000152	7
55789_at	55789	ENSG00000	DEPDC1B	DEP domain containing		I	33	0.373765	0.435877	0.678608	0.523792	1.111038	7.193402	27.29451	0.000128	0.000683	7
3842_at	3842	ENSG00000	TNPO1	transportin	transporte	I	29	0.515801	0.643535	0.747406	0.545821	1.07216	7.983878	51.17281	1.40E-05	0.000126	7
5585_at	5585	ENSG00000	PKN1	protein kin	kinase	NI	10	0.528137	0.344302	1.222023	0.671426	0.336373	4.797486	24.37465	0.000189	0.000927	7
55330_at	55330	ENSG00000	CNO	cappuccino homolog (r		I	11	0.356421	0.355478	1.324911	0.656807	0.350263	7.321739	28.48982	0.00011	0.00061	7
285362_at	285362	ENSG00000	SUMF1	sulfatase modifying fa		NI	30	0.428517	0.430088	1.184431	0.468728	0.413133	5.362383	33.9146	6.01E-05	0.000379	7
6228_at	6228	ENSG00000	RPS23	ribosomal protein S23		I	20	0.335807	0.235737	1.074714	0.603288	0.358814	11.56846	24.20719	0.000194	0.000944	7
10009363C	1E+08	NA	SNHG8	small nucleolar RNA ho		I	17	0.358655	0.424416	1.013768	0.683211	0.568904	10.3607	36.63008	4.58E-05	0.000308	7
153241_at	153241	ENSG00000	CEP120	centrosomal protein 1		NI	22	0.20837	0.575506	1.043381	0.657552	0.521149	4.640387	30.82767	8.39E-05	0.000492	7
5176_at	5176	ENSG00000	SERPINF1	serpin pept	Secreted	NI	52	0.143968	0.156657	1.233528	0.543518	0.481561	3.919389	23.3566	0.000219	0.001043	7
6044_at	6044	NA	SNORA62	small nucleolar RNA, H		NI	9	0.297397	0.154754	1.201758	0.436371	0.364651	4.521621	17.01167	0.00064	0.002492	7
135114_at	135114	ENSG00000	HINT3	histidine triad nucleoti		NI	21	0.046529	0.293718	1.113365	0.418726	0.541354	5.675948	15.23326	0.000922	0.003367	7
55812_at	55812	ENSG00000	SPATA7	spermatogenesis asso		NI	11	0.19945	0.087245	1.152745	0.766011	0.511507	4.901981	29.46034	9.83E-05	0.000558	7
5265_at	5265	ENSG00000	SERPINA1	serpin pept	peptidase	NI	35	0.188299	0.073651	1.215021	0.709339	0.407348	4.032826	37.58362	4.18E-05	0.000288	7
8334_at	8334	ENSG00000	HIST1H2AC	histone cluster 1, H2ac		I	11	0.535442	-0.0537	1.258379	0.31602	0.591318	6.529913	41.05895	3.06E-05	0.000227	7
6674_at	6674	ENSG00000	SPAG1	sperm associated antig		NI	22	0.369572	0.069826	1.254614	0.280272	0.500775	6.94959	32.96154	6.64E-05	0.000409	7
119392_at	119392	ENSG00000	SFR1	SWI5-dependent recor		NI	15	0.30345	0.201854	1.161898	0.202333	0.260263	6.777077	19.22175	0.000425	0.001786	7
2012_at	2012	ENSG00000	EMP1	epithelial membrane p		I	32	0.303893	0.353864	1.042034	0.776121	0.910682	8.376124	48.38728	1.71E-05	0.000147	7
163081_at	163081	ENSG00000	ZNF567	zinc finger protein 567		NI	22	0.186144	0.387456	1.071096	0.698865	0.96145	4.352927	46.26081	2.00E-05	0.000164	7
23256_at	23256	ENSG00000	SCFD1	sec1 family domain co		I	17	0.192271	0.372483	1.013841	0.729834	0.720294	8.352766	44.57893	2.28E-05	0.000181	7
345757_at	345757	ENSG00000	FAM174A	family with sequence s		NI	16	0.339102	0.349565	1.329576	0.51153	0.855066	6.45492	41.94471	2.84E-05	0.000215	7
51567_at	51567	ENSG00000	TDP2	tyrosyl-DNA phosphod		I	22	0.240696	0.302538	1.169387	0.44015	0.750188	7.509074	40.35255	3.25E-05	0.000238	7
1027_at	1027	ENSG00000	CDKN1B	cyclin-dependent kinas		I	22	0.095615	0.21446	1.108005	0.734596	1.157706	6.544665	44.81944	2.24E-05	0.000179	7
4082_at	4082	ENSG00000	MARCKS	myristoylated alanine-		I	13	0.275276	0.028455	1.063624	0.70813	1.175893	8.252433	16.01845	0.000781	0.002932	7
11162_at	11162	ENSG00000	NUDT6	nudix (nucleoside diph		NI	21	0.305374	0.194628	1.201881	0.864892	1.169537	5.681838	20.61791	0.000336	0.001471	7
2287_at	2287	ENSG00000	FKBP3	FK506 binding protein		I	8	0.000131	-0.02854	1.347007	0.78	0.860704	8.772116	28.05029	0.000117	0.000635	7
3655_at	3655	ENSG00000	ITGA6	integrin, al	Secreted	I	31	0.138412	-0.11181	1.148444	0.647912	0.896991	9.393179	22.49819	0.000249	0.001155	7
23512_at	23512	ENSG00000	SUZ12	suppressor of zeste 12		I	30	0.25923	0.273403	0.680279	0.590037	1.013407	8.152675	41.65741	2.91E-05	0.000218	7
81615_at	81615	ENSG00000	TMEM163	transmembrane protei		NI	22	0.244176	0.119402	0.60817	0.576385	1.008799	3.504884	25.86477	0.000154	0.000788	7
79982_at	79982	ENSG00000	DNAJB14	DnaJ (Hsp4	Secreted	NI	8	0.459896	0.210135	0.69025	0.534923	1.022348	7.720318	36.79973	4.51E-05	0.000304	7
114884_at	114884	ENSG00000	OSBPL10	oxysterol binding prote		NI	30	0.21523	0.347415	0.670838	0.869593	1.206183	5.148596	40.84763	3.12E-05	0.00023	7
414328_at	414328	ENSG00000	C9orf103	chromosor	kinase	NI	11	0.074398	0.292448	0.552147	0.74798	1.220066	4.188094	32.80337	6.75E-05	0.000415	7
157769_at	157769	ENSG00000	FAM91A1	family with sequence s		NI	12	-0.03613	0.112194	0.670466	0.400707	1.217888	7.577914	28.56509	0.000109	0.000605	7
64848_at	64848	ENSG00000	YTHDC2	YTH domain containing		NI	22	0.085102	-0.06067	0.646709	0.586412	1.040361	4.576103	44.64874	2.27E-05	0.000181	7
6373_at	6373	ENSG00000	CXCL11	chemokine	Secreted	NI	33	0.056359	0.004374	0.44932	0.678123	1.417464	3.607985	56.41924	9.84E-06	9.77E-05	7
10299_at	10299	ENSG00000	6-Mar	membrane-associated		NI	31	0.002604	0.029403	0.377583	0.184586	1.352958	8.242293	30.39545	8.82E-05	0.000511	7
55319_at	55319	ENSG00000	C4orf43	chromosome 4 open re		NI	10	-0.23049	-0.24432	0.962471	0.356533	1.241208	5.549791	41.17141	3.03E-05	0.000226	7
55015_at	55015	ENSG00000	PRPF39	PRP39 pre-mRNA proc		NI	11	-0.34509	-0.17028	0.830699	0.435676	1.032828	5.482027	17.56679	0.000575	0.002287	7
79725_at	79725	ENSG00000	THAP9	THAP domain containi		NI	11	0.073052	-0.26449	0.919121	0.541563	1.076415	4.48865	11.33907	0.002387	0.007445	7
7846_at	7846	ENSG00000	TUBA1A	tubulin, alp	GTPase	I	9	-0.31523	0.234549	0.915199	0.71967	1.175157	10.14634	17.2735	0.000608	0.002392	7
494143_at	494143	ENSG00000	CHAC2	ChaC, cation transport		I	11	-0.12033	0.251398	1.077243	0.378739	1.018355	7.456372	37.96782	4.04E-05	0.00028	7
23002_at	23002	ENSG00000	DAAM1	dishevelled associated		I	11	0.380055	-0.56065	1.354143	0.798543	1.433979	7.170896	24.54191	0.000185	0.000911	7
26035_at	26035	ENSG00000	GLCE	glucuronic acid epimer		NI	11	0.470173	-0.3752	0.78042	0.419207	1.177409	5.685811	29.35369	9.96E-05	0.000563	7
79366_at	79366	ENSG00000	HMGN5	high mobility group nu		NI	11	0.254584	-0.24693	1.56005	0.279177	0.87528	6.092037	21.66381	0.000284	0.001282	7
5033_at	5033	ENSG00000	P4HA1	prolyl 4-hy	Secreted	I	4	-0.54575	-0.4507	1.428051	0.671537	1.743303	6.676483	27.13025	0.000131	0.000695	7
657_at	657	ENSG00000	BMPR1A	bone morp	kinase	I	7	-0.8052	-0.54013	1.08315	0.345371	2.062894	4.517629	11.74943	0.002133	0.006778	7
100505815	1.01E+08	NA	LOC100505	hypothetical LOC1005C		NI	3	0.754869	1.735628	-2.07256	0.420239	-0.37268	4.917999	44.50965	2.30E-05	0.000182	6
441263_at	441263	NA	DTX2P1-UF	DTX2P1-UF	kinase	NI	3	-0.58476	1.377058	-0.85119	0.268479	-0.36269	4.671201	22.54365	0.000248	0.001149	6
574040_at	574040	NA	SNORA6	small nucleolar RNA, H		NI	11	0.084331	-0.63229	0.385937	-0.20516	1.003486	4.127447	10.59691	0.002951	0.008903	5
2653_at	2653	ENSG00000	GCSH	glycine clea	Secreted	NI	6	-0.19853	-1.13387	0.269681	-0.35682	0.297388	5.640099	21.29172	0.000301	0.001344	5
168451_at	168451	ENSG00000	THAP5	THAP domain containi		I	15	-0.72631	-1.05234	0.090139	0.034069	0.332365	7.229432	17.27592	0.000608	0.002392	5
3280_at	3280	ENSG00000	HES1	hairy and enhancer of		I	24	-1.31452	-0.60311	0.6832	-0.19717	-0.13876	6.763233	47.39682	1.84E-05	0.000155	5
692073_at	692073	NA	SNORA16A	small nucleolar RNA, H		NI	11	-1.1885	-0.38013	0.262328	-0.43451	-0.42016	9.908064	11.20576	0.002477	0.007686	5
664618_at	664618	NA	HSP90AB4I	heat shock protein 90k		NI	6	0.292295	0.431037	0.335659	-0.09621	-1.02344	4.52973	18.32728	0.000499	0.00203	5
3203_at	3203	ENSG00000	HOXA6	homeobox A6		NI	11	0.082778	0.306839	-0.17854	-0.16469	-1.00416	3.465639	10.64446	0.00291	0.008796	5
196968_at	196968	NA	DNM1P46	DNM1 pseudogene 46		NI	5	-0.20653	-0.26902	0.183373	-0.28721	-1.00614	3.187755	17.02208	0.000639	0.002488	5

54896_at	54896	ENSG0000(	PQLC2	PQ loop repeat contair	NI	10	0.006129	-0.31239	-0.08108	-0.1986	-1.00556	5.203909	23.13412	0.000227	0.001072	5
90850_at	90850	ENSG0000(	ZNF598	zinc finger protein 598	NI	3	-0.06651	-0.03306	-0.5193	-0.05116	-1.05911	7.411215	11.16504	0.002506	0.007754	5
7087_at	7087	ENSG0000(	ICAM5	intercellular adhesion	NI	11	-0.38767	-0.13406	-0.22692	0.027981	-1.18524	4.523359	25.63684	0.000159	0.000806	5
646513_at	646513	NA	LOC64651:	VLGN1945	NI	3	-0.28234	0.484417	-0.46811	-0.47361	-1.37957	5.455913	17.24178	0.000612	0.002402	5
8372_at	8372	ENSG0000(	HYAL3	hyaluronog Secreted	NI	11	-0.49849	0.062128	-0.49259	-0.47709	-1.29006	3.712246	18.09694	0.000521	0.002102	5
7867_at	7867	ENSG0000(	MAPKAPK3	mitogen-ac kinase	I	21	-0.01457	-0.41804	-0.35562	-0.78927	-1.10302	8.515331	26.30007	0.000146	0.000755	5
5874_at	5874	ENSG0000(	RAB27B	RAB27B, m GTPase	I	11	-0.16002	-0.16807	-0.39921	-0.98838	-1.14268	5.088346	21.56403	0.000288	0.001297	5
84164_at	84164	ENSG0000(	ASCC2	activating signal cointe	NI	9	0.159286	-0.08461	-0.51219	-0.56682	-1.16035	6.250209	26.00203	0.000152	0.000778	5
11336_at	11336	ENSG0000(	EXOC3	exocyst complex comp	NI	11	-0.22247	-0.36345	-0.35536	-0.45635	-1.0215	6.977648	10.36478	0.003162	0.009449	5
10636_at	10636	ENSG0000(	RGS14	regulator of G-protein	NI	22	-0.09641	-0.36786	-0.50496	-0.57809	-1.0559	4.064875	42.57077	2.69E-05	0.000206	5
27076_at	27076	ENSG0000(	LYPD3	LY6/PLAUR Secreted	NI	11	0.221468	0.051102	-1.01549	-0.39866	0.023419	4.03891	13.15765	0.001485	0.005	5
339521_at	339521	NA	FAM58BP	family with sequence s	NI	17	0.472808	0.17182	-1.03519	-0.1589	-0.59928	4.538772	32.39306	7.06E-05	0.00043	5
7480_at	7480	ENSG0000(	WNT10B	wingless-ty Secreted	NI	11	-0.14731	-0.10726	-1.35949	-0.32245	-1.20758	4.958026	62.17401	6.93E-06	7.63E-05	5
9605_at	9605	ENSG0000(	C16orf7	chromosor transporte	NI	11	-0.13468	-0.09208	-1.02466	-0.24989	-0.34923	5.200559	13.59713	0.001335	0.004576	5
2874_at	2874	ENSG0000(	GPS2	G protein pathway sup	I	5	-0.29425	-0.06285	-1.0308	-0.48004	-0.71086	8.785601	32.71342	6.82E-05	0.000417	5
84283_at	84283	ENSG0000(	TMEM79	transmembrane protei	NI	11	-0.54627	-0.13635	-1.04143	-0.2157	-0.66225	4.27344	30.01195	9.22E-05	0.000529	5
10148_at	10148	ENSG0000(	EBI3	Epstein-Ba Secreted	NI	11	-0.13617	-0.71066	-1.39046	-0.45554	-0.74504	4.200206	55.4813	1.04E-05	0.000102	5
2784_at	2784	ENSG0000(	GNB3	guanine nu GTPase	NI	11	-0.62467	-0.61421	-1.54601	-0.55834	-0.78915	4.421498	75.96175	3.36E-06	4.63E-05	5
1844_at	1844	ENSG0000(	DUSP2	dual specif phosphopr	NI	11	-0.47586	-0.29261	-1.21394	-0.87412	-0.51939	4.190969	18.29315	0.000502	0.002041	5
8078_at	8078	ENSG0000(	USP5	ubiquitin s peptidase	NI	9	-0.47988	-0.2027	-1.0771	-0.54336	-0.86518	6.334775	15.78263	0.00082	0.003061	5
60673_at	60673	ENSG0000(	C12orf44	chromosome 12 open	I	11	-0.61323	-0.35482	-1.22213	-0.62997	-0.92825	8.31819	35.31386	5.21E-05	0.000341	5
150356_at	150356	ENSG0000(	CHADL	chondroadherin-like	NI	11	-0.76124	-0.21121	-1.29872	-0.38458	-0.54414	4.462429	12.87605	0.001592	0.005314	5
6663_at	6663	ENSG0000(	SOX10	SRY (sex determining r	NI	22	-0.49022	-0.31026	-1.26451	-0.53395	-0.57532	3.803173	31.42887	7.85E-05	0.000467	5
83861_at	83861	ENSG0000(	RSPH3	radial spoke 3 homolo	NI	10	-0.60599	-0.50049	-1.08809	-0.47404	-0.62495	4.253471	51.74245	1.34E-05	0.000122	5
255783_at	255783	NA	PRR24	proline rich 24	NI	11	-0.53427	-0.41463	-1.19441	-0.45289	-0.71389	5.282701	42.90929	2.62E-05	0.000202	5
91445_at	91445	ENSG0000(	RNF185	ring finger protein 185	NI	6	-0.1908	-1.1536	-0.90471	-0.38629	-0.44735	4.476712	10.76789	0.002807	0.008535	5
133690_at	133690	ENSG0000(	CAPSL	calcyphosine-like	NI	8	-0.45054	-1.04482	-0.56636	-0.40981	-0.76553	3.622735	35.52455	5.10E-05	0.000335	5
125061_at	125061	ENSG0000(	AFMID	arylformamidase	NI	11	-0.20328	-0.99169	-1.18056	-0.68709	-0.44988	5.348895	15.13686	0.000941	0.003427	5
27314_at	27314	ENSG0000(	RAB30	RAB30, me GTPase	NI	22	-0.11651	-0.63545	-1.05045	-0.56412	-0.46267	4.791132	21.9435	0.000271	0.001241	5
84824_at	84824	ENSG0000(	FCRLA	Fc receptor Secreted	NI	40	-0.44569	-0.75936	-1.00634	-0.62673	-0.5151	4.250884	45.30131	2.16E-05	0.000174	5
400506_at	400506	ENSG0000(	C16orf88	chromosome 16 open	I	20	-0.19597	-0.72345	-0.84898	-1.04862	-0.63862	6.494144	63.02695	6.60E-06	7.36E-05	5
1513_at	1513	ENSG0000(	CTSK	cathepsin k peptidase	NI	11	-0.05693	-0.96464	-1.06283	-0.86163	-0.93908	5.037142	21.59106	0.000287	0.001293	5
1016_at	1016	ENSG0000(	CDH18	cadherin 18, type 2	NI	20	-0.28396	-1.06515	-0.80103	-0.7157	-0.93121	3.812019	64.5254	6.07E-06	6.96E-05	5
51400_at	51400	ENSG0000(	PPME1	protein phosphatase m	NI	11	-0.32742	-0.91174	-0.87552	-0.7018	-1.00161	6.766773	15.68098	0.000838	0.003115	5
5118_at	5118	ENSG0000(	PCOLCE	procollage Secreted	NI	11	-0.27159	-1.19438	-1.45544	-0.9189	-0.56911	4.977811	14.1687	0.001168	0.0041	5
727936_at	727936	ENSG0000(	GXYLT2	glucoside xylosyltransf	I	11	-0.40677	-1.309	-1.33811	-1.0623	-0.63929	6.491909	27.547	0.000124	0.000668	5
26052_at	26052	ENSG0000(	DNM3	dynamin 3 GTPase	NI	9	-0.62997	-1.0711	-1.38114	-0.53592	0.121148	4.124396	47.46852	1.83E-05	0.000154	5
29066_at	29066	ENSG0000(	ZC3H7A	zinc finger CCCH-type	I	32	-0.77866	-1.10217	-0.87738	-0.59941	-0.05559	7.479014	36.75477	4.53E-05	0.000305	5
10208_at	10208	ENSG0000(	USPL1	ubiquitin s peptidase	NI	11	-0.73384	-1.17916	-0.9355	-0.64219	-0.00382	6.195702	36.80455	4.51E-05	0.000304	5
80071_at	80071	ENSG0000(	CCDC15	coiled-coil domain con	NI	11	-0.4904	-1.38116	-1.0635	-0.89981	-0.3264	5.587422	72.46602	3.99E-06	5.17E-05	5
148398_at	148398	ENSG0000(	SAMD11	sterile alpha motif dor	NI	10	-0.75574	-1.14238	-0.95247	-0.86337	-0.37501	5.258168	71.11549	4.27E-06	5.41E-05	5
2009_at	2009	ENSG0000(	EML1	echinoderm microtubu	NI	54	-0.77015	-1.2269	-1.01812	-0.84985	-0.35442	4.16904	75.21069	3.49E-06	4.73E-05	5
261726_at	261726	ENSG0000(	TIPRL	TIP41, TOR signaling p	I	31	-0.81513	-1.22629	-0.81595	-0.81023	-0.42745	7.820892	63.72277	6.35E-06	7.17E-05	5
2033_at	2033	ENSG0000(	EP300	E1A binding protein p3	I	10	-0.76876	-1.15194	-0.82974	-0.86574	-0.23268	6.725337	69.33191	4.68E-06	5.76E-05	5
54704_at	54704	ENSG0000(	PDP1	pyruvate d phosphopr	I	33	-0.83765	-1.19955	-0.88973	-0.79634	-0.23177	8.727657	18.01167	0.000529	0.002129	5
719_at	719	ENSG0000(	C3AR1	compleme G-protein c	NI	11	-0.10045	-1.59122	-0.33231	-0.85158	-0.66946	4.097252	92.10417	1.67E-06	2.87E-05	5
128486_at	128486	ENSG0000(	FITM2	fat storage-inducing tr	NI	11	-0.32927	-1.58158	-0.90074	-0.76189	-0.68258	5.216517	88.64163	1.92E-06	3.13E-05	5
1431_at	1431	ENSG0000(	CS	citrate synthase	NI	4	-0.28848	-1.12844	-0.19949	-0.71504	-0.65571	5.295706	42.10015	2.80E-05	0.000212	5
339761_at	339761	ENSG0000(	CYP27C1	cytochrome P450, fam	NI	11	-0.63264	-1.07719	-0.01632	-0.93606	-0.57408	3.541039	20.74547	0.000329	0.001445	5
26191_at	26191	ENSG0000(	PTPN22	protein tyr phosphopr	I	28	-0.1879	-0.95562	-0.16556	-1.16543	-0.91365	4.411748	74.89663	3.54E-06	4.77E-05	5
255928_at	255928	ENSG0000(	SYT14	synaptotag Secreted	NI	22	-0.59429	-0.64459	-0.3319	-1.23749	-0.98273	3.681088	64.15491	6.19E-06	7.04E-05	5
23515_at	23515	ENSG0000(	MORC3	MORC fam transporte	I	22	-0.69555	-1.40694	-0.28855	-0.46275	0.004389	6.88303	27.34876	0.000127	0.00068	5
5290_at	5290	ENSG0000(	PIK3CA	phosphoin kinase	I	11	-0.51454	-1.09658	-0.27237	-0.4435	-0.04236	7.835548	16.43528	0.000718	0.002736	5
79572_at	79572	ENSG0000(	ATP13A3	ATPase typ transporte	I	11	-0.5578	-1.04304	-0.46852	-0.38503	-0.04698	9.119903	24.05454	0.000198	0.000961	5
51136_at	51136	ENSG0000(	RNFT1	ring finger protein, tra	NI	22	-0.61595	-1.02024	-0.13819	-0.40996	-0.15034	5.427646	27.04956	0.000132	0.0007	5
4686_at	4686	ENSG0000(	NCBP1	nuclear cap binding pr	NI	20	-0.6551	-1.04339	-0.23053	-0.52221	-0.23453	7.827109	28.35028	0.000112	0.000617	5
440567_at	440567	ENSG0000(	UQCRHL	ubiquinol-c transporte	I	11	-0.93535	-1.11143	-0.11169	-0.57942	-0.23071	7.321013	23.59278	0.000212	0.001015	5
26973_at	26973	ENSG0000(	CHORDC1	cysteine and histidine-	I	25	-0.9812	-1.00136	-0.22651	-0.6941	-0.19813	6.798611	33.02266	6.60E-05	0.000408	5
4751_at	4751	ENSG0000(	NEK2	NIMA (nev kinase	NI	61	-1.067	-1.01591	-0.16335	-0.48307	-0.05379	6.034782	34.81651	5.48E-05	0.000354	5
23251_at	23251	ENSG0000(	KIAA1024	KIAA1024	NI	11	-1.0487	-0.92537	-0.0335	-0.55098	-0.0796	3.935697	16.5577	0.0007	0.002686	5
23527_at	23527	ENSG0000(	ACAP2	ArfGAP with coiled-coi	NI	21	-1.04717	-0.8792	-0.08404	-0.58319	-0.10008	5.145479	47.7818	1.78E-05	0.000152	5
9541_at	9541	ENSG0000(	CIR1	corepressor interacting	NI	10	-1.03389	-1.1397	-0.41128	-0.37377	-0.27471	7.205143	36.01331	4.86E-05	0.000322	5
51258_at	51258	ENSG0000(	MRPL51	mitochondrial ribosom	I	10	-1.00399	-1.15957	-0.31173	-0.48073	-0.36781	11.27777	48.21418	1.73E-05	0.000149	5
56133_at	56133	ENSG0000(	PCDHB2	protocadherin beta 2	I	17	-1.00693	-0.95007	-0.17773	-0.38212	-0.6264	6.9886	50.8785	1.42E-05	0.000128	5
7770_at	7770	ENSG0000(	ZNF227	zinc finger protein 227	NI	11	-0.91501	-1.1479	-0.04301	-0.40416	-0.31411	6.76	31.90416	7.44E-05	0.000448	5
6302_at	6302	ENSG0000(	TSPAN31	tetraspanin 31	NI	22	-0.97658	-1.17984	-0.00537	-0.52217	-0.50758	5.271493	45.09075	2.19E-05	0.000177	5
3685_at	3685	ENSG0000(	ITGAV	integrin, alpha V (vitro	NI	52	-0.88166	-1.13818	-0.47085	-0.84909	-0.01022	4.56031	60.87579	7.48E-06	8.00E-05	5
199221_at	199221	ENSG0000(	DZIP1L	DAZ interacting protei	NI	11	-0.94452	-0.93023	-0.76466	-1.02755	-0.27536	4.357702	83.03875	2.43E-06	3.69E-05	5
80205_at	80205	ENSG0000(	CHD9	chromodomain helicas	I	44	-1.02321	-1.10517	-0.61411	-0.90799	-0.3732	5.34784	81.93666	2.55E-06	3.83E-05	5
23376_at	23376	ENSG0000(	UFL1	UFM1-specific ligase 1	I	30	-0.8615	-1.1782	-0.66242	-1.00234	-0.32686	6.673939	63.79056	6.32E-06	7.15E-05	5
23468_at	23468	ENSG0000(	CBX5	chromobox homolog 5	NI	49	-0.93763	-1.33596	-0.73439	-0.72368	-0.36089	4.630828	56.94312	9.51E-06	9.54E-05	5
96764_at	96764	ENSG0000(	TGS1	trimethylguanosine sy	I	21	-1.02476	-1.21347	-0.72015	-0.76049	-0.42237	6.345077	44.0296	2.39E-05	0.000188	5
29899_at	29899	ENSG0000(	GPSM2	G-protein signaling mc	I	11	-0.85996	-1.25159	-0.5782	-0.56098	-0.31531	9.465204	47.2727	1.85E-05	0.000156	5
258010_at	258010	ENSG0000(	SVIP	small VCP/p97-interac	I	20	-0.92311	-1.17466	-0.41435	-0.68678	-0.28905	6.798276	45.72579	2.09E-05	0.00017	5
7272_at	7272	ENSG0000(	TTK	TTK protein kinase	I	10	-0.96622	-1.08422	-0.52295	-0.74003	-0.29361	9.091912	62.11943	6.96E-06	7.64E-05	5
28513_at	28513	ENSG0000(	CDH19	cadherin 19, type 2	NI	33	-0.6989	-1.00566	-0.41713	-0.5345	-0.38017	3.381172	47.27958	1.85E-05	0.000156	5
54629_at	54629	ENSG0000(	FAM63B	family with transporte	NI	25	-0.69659	-1.08606	-0.52131	-0.54728	-0.22738	3.928633	30.21201	9.01E-05	0.000519	5
55676_at	55676	ENSG0000(	SLC30A6	solute carri transporte	NI	42	-0.70948	-1.01224	-0.52579	-0.81099	-0.2797	5.22092	63.80197	6.32E-06	7.15E-05	5
5965_at	5965	ENSG0000(	RECQL	RecQ protein-like (DN/	I	23	-0.78526	-1.01215	-0.39212	-0.73498	-0.43342	8.602093	56.46535	9.81E-06	9.75E-05	5
9101_at	9101	ENSG0000(	USP8	ubiquitin s peptidase	I	32	-0.76735	-1.02268	-0.50819	-0.63693	-0.36445	7.193217	30.71284	8.50E-05	0.000496	5
148867_at	148867	ENSG0000(	SLC30A7	solute carri transporte	I	32	-0.79749	-1.08635	-0.49085	-0.61609	-0.32805	7.521463	56.52736	9.77E-06	9.73E-05	5
84154_at	84154	ENSG0000(	RPF2	ribosome production f	I	7	-0.72966	-1.04028	-0.6013	-0.65715	-0.38332	9.141343	46.55135	1.96E-05	0.000161	5

55843_at	55843	ENSG0000(	ARHGAP15	Rho GTPase activating		NI	19	-0.78332	-1.04785	-0.52732	-0.75003	-0.40466	3.765954	10.78351	0.002795	0.008502	5
401466_at	401466	ENSG0000(	C8orf59	chromosome 8 open r		I	25	-0.8308	-1.07821	-0.61617	-0.84112	-0.73412	8.848543	43.78466	2.43E-05	0.000191	5
6894_at	6894	ENSG0000(	TARBP1	TAR (HIV-1) RNA bindi		I	11	-0.96	-1.01597	-0.5005	-0.83987	-0.76208	7.043726	60.96286	7.44E-06	7.99E-05	5
191_at	191	ENSG0000(	AHCY	adenosylhomocysteina		I	18	-0.93659	-1.04778	-0.58765	-0.70952	-0.76592	9.376145	49.70112	1.55E-05	0.000137	5
85415_at	85415	ENSG0000(	RHPN2	rhophilin, Rho GTPase		NI	17	-0.93134	-1.07409	-0.58731	-0.79131	-0.68725	5.824884	39.0824	3.64E-05	0.000259	5
375444_at	375444	ENSG0000(	C5orf34	chromosome 5 open r		I	21	-0.96545	-1.00948	-0.60682	-0.77083	-0.72485	5.358659	66.24057	5.52E-06	6.49E-05	5
51506_at	51506	ENSG0000(	UFC1	ubiquitin-fold modifier		I	9	-1.07319	-1.10914	-0.426	-0.71488	-0.44884	9.537002	68.6917	4.84E-06	5.90E-05	5
780_at	780	NA	DDR1	discoidin d	kinase	I	40	-1.13486	-1.0726	-0.40971	-0.70971	-0.56819	6.120203	48.54396	1.69E-05	0.000146	5
54842_at	54842	ENSG0000(	MFSD6	major facilitator super		I	33	-1.13808	-1.1261	-0.438	-0.89668	-0.66948	6.17463	37.25792	4.31E-05	0.000294	5
146754_at	146754	ENSG0000(	DNAH2	dynein, axonemal, hea		I	20	-1.18084	-1.17392	-0.57531	-0.79095	-0.5439	6.770452	65.76641	5.66E-06	6.62E-05	5
6120_at	6120	ENSG0000(	RPE	ribulose-5-phosphate-		I	34	-1.10171	-1.10324	-0.58072	-0.81809	-0.47017	7.182677	64.92013	5.93E-06	6.85E-05	5
8675_at	8675	NA	STX16	syntaxin 1(	Secreted	I	59	-1.05776	-1.12073	-0.62973	-0.83476	-0.56638	8.29198	70.98922	4.30E-06	5.42E-05	5
2059_at	2059	ENSG0000(	EPS8	epidermal growth fact		I	31	-0.59714	-1.01523	-0.42419	-0.76975	-0.4472	6.665322	19.69041	0.000392	0.001671	5
4999_at	4999	ENSG0000(	ORC2	origin recognition com		NI	32	-0.53677	-1.07819	-0.4314	-0.7209	-0.54548	5.866735	42.2209	2.77E-05	0.000211	5
54103_at	54103	ENSG0000(	PION	pigeon homolog (Dros		I	30	-0.65172	-1.11693	-0.53574	-0.70452	-0.74455	5.768511	56.42826	9.83E-06	9.77E-05	5
427_at	427	ENSG0000(	ASAH1	N-acylsphir	Secreted	I	52	-0.65205	-1.01262	-0.6582	-0.75645	-0.77145	7.712229	25.26866	0.000167	0.00084	5
63926_at	63926	ENSG0000(	ANKRD5	ankyrin repeat domain		NI	19	-0.60582	-1.07723	-0.41346	-0.99957	-0.73562	4.184096	59.5571	8.10E-06	8.45E-05	5
483_at	483	ENSG0000(	ATP1B3	ATPase, Na	transporte	I	14	-0.56401	-1.03949	-0.63471	-0.91755	-0.5728	9.009761	64.48287	6.08E-06	6.97E-05	5
83941_at	83941	ENSG0000(	TM2D1	TM2 domain containin		I	8	-0.57891	-1.13732	-0.61132	-0.89702	-0.67945	8.862993	40.35392	3.25E-05	0.000238	5
56986_at	56986	ENSG0000(	DTWD1	DTW domain containir		NI	32	-0.75242	-1.34735	-0.47004	-0.79995	-0.50904	5.765248	65.29757	5.81E-06	6.75E-05	5
874_at	874	ENSG0000(	CBR3	carbonyl reductase 3		I	11	-0.84404	-1.12077	-0.3993	-0.6407	-0.57604	8.380523	35.893	4.92E-05	0.000325	5
64421_at	64421	ENSG0000(	DCLRE1C	DNA cross-link repair 1		NI	22	-0.90562	-1.19417	-0.48262	-0.70228	-0.65754	6.296876	35.28979	5.22E-05	0.000341	5
79149_at	79149	ENSG0000(	ZSCAN5A	zinc finger and SCAN d		NI	8	-0.86536	-1.22617	-0.66624	-0.92777	-0.50018	4.598119	13.49536	0.001368	0.004665	5
960_at	960	ENSG0000(	CD44	CD44 mole	Secreted	I	109	-0.93102	-1.19227	-0.55968	-0.98417	-0.6009	10.45143	61.01651	7.42E-06	7.97E-05	5
116843_at	116843	ENSG0000(	C6orf192	chromosor	transporte	I	20	-0.72803	-1.05644	-0.59976	-0.95605	-0.39282	7.039619	61.94134	7.03E-06	7.67E-05	5
51110_at	51110	ENSG0000(	LACTB2	lactamase, beta 2		I	14	-0.79996	-1.04944	-0.68124	-0.95571	-0.54826	9.283248	61.67982	7.14E-06	7.75E-05	5
54931_at	54931	ENSG0000(	RG9MTD1	RNA (guanine-9-) metl		I	22	-0.73841	-1.08072	-0.63676	-0.78886	-0.46966	9.337731	71.25586	4.24E-06	5.39E-05	5
7707_at	7707	ENSG0000(	ZNF148	zinc finger protein 148		NI	97	-0.76833	-1.12752	-0.74568	-0.85285	-0.49394	6.423862	54.44783	1.12E-05	0.000107	5
9317_at	9317	ENSG0000(	PTER	phosphotriesterase rel		I	44	-0.77376	-1.15086	-0.6659	-0.90627	-0.51422	7.289247	77.65724	3.10E-06	4.37E-05	5
138255_at	138255	ENSG0000(	C9orf135	chromosome 9 open r		NI	10	-2.17324	-0.86432	-1.2943	-0.08828	-0.20847	3.699778	73.8124	3.73E-06	4.94E-05	5
4340_at	4340	NA	MOG	myelin oligodendrocyt		I	42	-1.75461	-0.85244	-1.43751	-0.4579	-0.38666	3.57179	102.4956	1.13E-06	2.23E-05	5
25758_at	25758	ENSG0000(	C11orf41	chromosome 11 open		NI	22	-1.67241	-1.0639	-1.51392	-0.54709	-0.77794	4.139516	29.63655	9.63E-05	0.000548	5
10100_at	10100	ENSG0000(	TSPAN2	tetraspanin 2		NI	28	-0.7171	-0.27721	-1.25512	-0.14572	-0.1807	3.481929	28.49825	0.00011	0.00061	5
347733_at	347733	ENSG0000(	TUBB2B	tubulin, be	GTPase	NI	11	-1.27969	-0.50905	-1.16922	-0.44325	-0.57438	4.176076	64.57863	6.05E-06	6.94E-05	5
3897_at	3897	ENSG0000(	L1CAM	L1 cell adhesion molec		NI	21	-1.20658	-0.42042	-0.83887	-0.40309	-0.37196	5.578397	57.53568	9.17E-06	9.27E-05	5
23596_at	23596	ENSG0000(	OPN3	opsin 3	G-protein c	NI	11	-1.16241	-0.37815	-0.92996	-0.25713	-0.56277	6.365435	29.52	9.76E-05	0.000554	5
3709_at	3709	ENSG0000(	ITPR2	inositol 1,4	ion channe	NI	31	-1.44918	-0.91714	-0.71045	-0.43369	-0.10774	4.519689	72.01379	4.08E-06	5.24E-05	5
387763_at	387763	ENSG0000(	C11orf96	chromosome 11 open		NI	11	-1.52161	-0.47963	-0.57618	-0.49778	-0.36365	4.131078	17.49045	0.000583	0.002314	5
25960_at	25960	ENSG0000(	GPR124	G protein-c	G-protein c	NI	22	-1.65712	-0.56065	-0.96733	-0.34506	-0.41974	3.897471	86.16901	2.13E-06	3.39E-05	5
84626_at	84626	ENSG0000(	KRBA1	KRAB-A domain contai		NI	11	-1.65866	-0.75435	-0.98531	-0.55338	-0.33883	4.720033	33.9294	6.00E-05	0.000379	5
7486_at	7486	ENSG0000(	WRN	Werner syndrome, Re(		NI	11	-1.31095	-0.50232	-0.22562	-0.71444	-0.0938	5.676711	18.42871	0.00049	0.002004	5
94039_at	94039	ENSG0000(	ZNF101	zinc finger protein 101		NI	19	-1.18821	-0.32449	-0.49424	-0.93759	-0.38826	4.375765	32.73708	6.80E-05	0.000417	5
90381_at	90381	ENSG0000(	C15orf42	chromosome 15 open		NI	11	-1.05358	-0.25143	-0.41635	-0.53647	-0.55883	4.814789	23.31125	0.000221	0.00105	5
57210_at	57210	ENSG0000(	SLC45A4	solute carrier family 4!		NI	10	-1.07483	-0.47666	-0.37315	-0.83317	-0.64245	5.968785	30.95945	8.27E-05	0.000487	5
51390_at	51390	ENSG0000(	AIG1	androgen-induced 1		I	10	-1.04071	-0.3885	-0.62852	-0.71155	-0.67941	7.106682	38.1151	3.98E-05	0.000277	5
84984_at	84984	ENSG0000(	CEP19	centrosomal protein 1!		NI	22	-1.00388	-0.62177	-0.43618	-0.48992	-0.19297	5.916723	34.35492	5.74E-05	0.000366	5
175_at	175	ENSG0000(	AGA	aspartylglu	Secreted	NI	32	-1.00722	-0.92409	-0.49846	-0.33838	-0.2565	6.989305	21.74208	0.00028	0.00127	5
30011_at	30011	ENSG0000(	SH3KBP1	SH3-domai	kinase	I	38	-1.03267	-0.88069	-0.48065	-0.42302	-0.38971	8.430871	23.03437	0.00023	0.001085	5
1952_at	1952	ENSG0000(	CELSR2	cadherin, E	G-protein c	NI	22	-1.22429	-0.89223	-0.71696	-0.60318	-0.07372	4.738029	15.59601	0.000853	0.003161	5
2744_at	2744	ENSG0000(	GLS	glutaminase		NI	31	-1.10917	-0.91319	-0.82209	-0.67125	-0.21859	4.622414	14.62985	0.001052	0.003759	5
2633_at	2633	ENSG0000(	GBP1	guanylate I	GTPase	I	36	-0.92904	-1.04625	-0.59936	-0.53409	-0.21905	6.864778	12.16821	0.001908	0.006176	5
51380_at	51380	ENSG0000(	CSAD	cysteine su	Secreted	NI	20	-1.00747	-0.87046	-0.58714	-0.6382	-0.12887	3.953972	39.85818	3.40E-05	0.000246	5
54965_at	54965	ENSG0000(	PIGX	phosphatidylinositol gl		I	33	-1.03019	-0.99115	-0.50754	-0.64695	-0.10461	7.264537	57.81372	9.01E-06	9.17E-05	5
6642_at	6642	ENSG0000(	SNX1	sorting nex	transporte	I	21	-1.04327	-0.78631	-0.87145	-0.4392	-0.47901	7.295629	61.29059	7.30E-06	7.88E-05	5
79090_at	79090	ENSG0000(	TRAPPC6A	trafficking protein part		NI	9	-1.09616	-0.78605	-0.80915	-0.61052	-0.40684	4.697641	29.61596	9.65E-05	0.000549	5
1827_at	1827	ENSG0000(	RCAN1	regulator of calcineuri		I	49	-1.162	-0.59411	-0.63216	-0.32137	-0.52593	8.724929	14.50739	0.001082	0.003846	5
5337_at	5337	ENSG0000(	PLD1	phospholipase D1, phc		NI	32	-1.21629	-0.72595	-0.48951	-0.38672	-0.50877	5.326073	54.70952	1.10E-05	0.000106	5
6470_at	6470	ENSG0000(	SHMT1	serine hydroxymethylt		NI	36	-1.18726	-0.84315	-0.6361	-0.42965	-0.534	5.586384	40.92531	3.10E-05	0.000229	5
6715_at	6715	ENSG0000(	SRD5A1	steroid-5-alpha-reduct		NI	32	-1.22794	-0.59959	-0.30617	-0.40109	-0.44745	5.082165	29.02276	0.000104	0.000581	5
201299_at	201299	ENSG0000(	RDM1	RAD52 motif 1		NI	56	-1.13592	-0.56713	-0.33622	-0.59432	-0.66493	3.79365	38.42893	3.87E-05	0.000271	5
10123_at	10123	ENSG0000(	ARL4C	ADP-ribosy	GTPase	I	31	-1.0131	-0.61834	-0.35904	-0.62984	-0.50301	10.00082	23.83009	0.000205	0.000986	5
84223_at	84223	ENSG0000(	IQCG	IQ motif containing G		NI	11	-1.1459	-0.69056	-0.31641	-0.5897	-0.53963	4.848296	17.81466	0.000549	0.002201	5
10855_at	10855	ENSG0000(	HPSE	heparanas	Secreted	NI	19	-1.29161	-0.76154	-0.42325	-0.75856	-0.72967	5.834288	61.01162	7.42E-06	7.97E-05	5
26355_at	26355	ENSG0000(	FAM162A	family with sequence s		I	8	-1.09869	-0.87618	-0.32624	-0.59516	-0.67333	9.764908	32.23036	7.18E-05	0.000436	5
144363_at	144363	ENSG0000(	LYRM5	LYR motif containing 5		NI	8	-1.12125	-1.00327	-0.45444	-0.64511	-0.73786	4.753893	30.71571	8.50E-05	0.000496	5
6596_at	6596	ENSG0000(	HLTF	helicase-like transcript		I	22	-1.02637	-0.77264	-0.4337	-0.64436	-0.39077	7.091588	35.23556	5.25E-05	0.000342	5
9111_at	9111	ENSG0000(	NMI	N-myc (and STAT) inte		I	52	-1.05254	-0.84613	-0.47889	-0.71173	-0.58409	8.152659	38.59269	3.81E-05	0.000268	5
55179_at	55179	ENSG0000(	FAIM	Fas apoptotic inhibitor		NI	22	-1.28496	-0.87738	-0.28402	-0.5983	-0.33958	5.917611	49.67044	1.55E-05	0.000137	5
81671_at	81671	ENSG0000(	VMP1	vacuole membrane pr(		I	19	-1.3347	-0.78154	-0.40365	-0.59475	-0.53837	9.329436	47.3787	1.84E-05	0.000155	5
1466_at	1466	ENSG0000(	CSRP2	cysteine and glycine-ri		I	7	-1.81858	-1.65263	-0.63655	-0.40338	0.038519	7.416802	18.4924	0.000484	0.001987	5
55437_at	55437	ENSG0000(	STRADB	STE20-related kinase a		NI	3	-1.29592	-1.34488	-0.36572	-0.53596	0.420594	4.826644	31.69499	7.62E-05	0.000456	5
79589_at	79589	ENSG0000(	RNF128	ring finger protein 128		I	11	-1.54903	-1.30044	0.000443	-0.61728	0.111902	7.078395	38.16805	3.96E-05	0.000276	5
23530_at	23530	ENSG0000(	NNT	nicotinami	transporte	I	54	-1.01975	-0.91851	-0.27687	-0.37702	0.086633	7.684143	25.17901	0.000169	0.000849	5
10096_at	10096	ENSG0000(	ACTR3	ARP3 actin-related pro		I	15	-1.34156	-0.9207	-0.17293	-0.43761	0.225002	10.04626	54.40561	1.12E-05	0.000107	5
27075_at	27075	ENSG0000(	TSPAN13	tetraspanin 13		I	11	-1.14328	-1.17336	-0.19161	-0.19737	-0.11884	7.301056	22.5475	0.000247	0.001148	5
27242_at	27242	ENSG0000(	TNFRSF21	tumor necrosis factor r		I	29	-1.18991	-1.04189	-0.5284	-0.23601	-0.06124	9.261254	51.72655	1.34E-05	0.000122	5
1945_at	1945	ENSG0000(	EFNA4	ephrin-A4	kinase	NI	11	-1.31348	-1.57218	-0.13618	-1.0849	-0.43498	5.006294	75.45199	3.45E-06	4.70E-05	5
90806_at	90806	ENSG0000(	ANGEL2	angel homolog 2 (Dros		I	22	-1.16631	-1.4088	-0.30307	-0.88716	-0.37704	5.528531	72.64809	3.95E-06	5.14E-05	5
55027_at	55027	ENSG0000(	HEATR3	HEAT repeat containin		NI	9	-0.94394	-1.34823	-0.16644	-0.80755	-0.21297	4.538499	22.52216	0.000248	0.001152	5
5423_at	5423	ENSG0000(	POLB	polymerase (DNA direc		I	9	-0.99416	-1.25303	-0.11461	-0.74756	-0.37647	8.263055	58.90998	8.42E-06	8.71E-05	5
51635_at	51635	ENSG0000(	DHRS7	dehydroge	Secreted	I	19	-0.98333	-1.67816	-0.43582	-0.54097	-0.22062	5.724397	71.32754	4.22E-06	5.38E-05	5
55783_at	55783	ENSG0000(	FTSJD1	FtsJ methyltransferase		I	22	-1.16711	-1.68895	-0.43431	-0.61026	-0.34469	5.794883	49.80577	1.54E-05	0.000136	5

7071_at	7071	ENSG0000(	KLF10	Kruppel-like factor 10		I	8	-1.44324	-1.52827	-0.11196	-0.67914	-0.12003	8.610853	105.689	1.01E-06	2.09E-05	5
10438_at	10438	ENSG0000(	C1D	C1D nuclear receptor c		I	9	-1.51452	-1.70627	-0.29131	-0.66544	-0.16345	6.702076	36.67933	4.56E-05	0.000307	5
57405_at	57405	NA	SPC25	SPC25, NDC80 kinetoc		I	10	-1.42928	-1.30045	-0.31868	-0.75968	-0.0712	8.113318	80.99009	2.66E-06	3.94E-05	5
10081_at	10081	ENSG0000(	PDCD7	programmed cell deatl		I	8	-1.36197	-1.31948	-0.34702	-0.90757	-0.19848	6.830434	70.13393	4.49E-06	5.60E-05	5
25907_at	25907	ENSG0000(	TMEM158	transmembrane protei		I	11	-1.3763	-1.42928	-0.38036	-0.93564	-0.10249	9.482652	59.8948	7.93E-06	8.35E-05	5
5872_at	5872	ENSG0000(	RAB13	RAB13, me	GTPase	I	3	-1.41187	-1.09569	-0.53873	-0.80451	-0.2408	10.01407	43.47683	2.50E-05	0.000195	5
27022_at	27022	ENSG0000(	FOXD3	forkhead box D3		NI	11	-1.45046	-1.10304	-0.64696	-1.00154	-0.4274	3.872809	20.61016	0.000336	0.001471	5
7057_at	7057	ENSG0000(	THBS1	thrombospondin 1		I	40	-1.45332	-0.85197	-0.64177	-0.8067	-0.4058	9.132264	28.07689	0.000116	0.000634	5
114299_at	114299	NA	PALM2	paralemmin 2		NI	22	-1.26315	-0.74113	-0.68453	-0.85491	-0.53818	4.364861	56.86094	9.56E-06	9.59E-05	5
4086_at	4086	ENSG0000(	SMAD1	SMAD fami	Secreted	I	22	-1.30837	-0.8342	-0.70692	-0.81155	-0.5307	7.199322	58.79281	8.48E-06	8.75E-05	5
79618_at	79618	ENSG0000(	HMBOX1	homeobox containing		NI	22	-1.22879	-1.09289	-0.67048	-0.65279	-0.50397	4.088792	39.73238	3.44E-05	0.000248	5
81627_at	81627	ENSG0000(	TRMT1L	TRM1 tRN/	Secreted	I	43	-1.28062	-1.02762	-0.62185	-0.70855	-0.59775	5.772845	52.36021	1.29E-05	0.000119	5
9816_at	9816	ENSG0000(	URB2	URB2 ribosome bioger		NI	11	-1.32418	-0.96394	-0.67569	-0.70473	-0.57435	4.488757	75.88968	3.37E-06	4.64E-05	5
23446_at	23446	ENSG0000(	SLC44A1	solute carri	transporte	I	22	-1.13421	-1.04485	-0.69041	-0.80913	-0.60237	9.788191	65.82708	5.64E-06	6.61E-05	5
9910_at	9910	ENSG0000(	RABGAP1L	RAB GTPase activating		NI	11	-1.21568	-1.03351	-0.72493	-0.78344	-0.52692	6.276344	64.2007	6.18E-06	7.03E-05	5
57560_at	57560	NA	IFT80	intraflagellar transport		I	20	-1.23077	-0.93175	-0.80712	-0.76349	-0.60427	6.414845	42.74141	2.65E-05	0.000204	5
60491_at	60491	ENSG0000(	NIF3L1	NIF3 NGG1 interacting		I	18	-1.22419	-0.99029	-0.80781	-0.79955	-0.62663	8.238196	90.28331	1.80E-06	3.01E-05	5
29980_at	29980	ENSG0000(	DONSON	downstream neighbor		I	10	-1.21443	-1.33384	-0.62841	-0.65626	-0.39431	8.715982	57.14612	9.39E-06	9.44E-05	5
10314_at	10314	ENSG0000(	LANCL1	LanC lantib	G-protein c	NI	54	-1.23153	-1.3248	-0.73607	-0.80391	-0.6234	5.541773	51.38087	1.38E-05	0.000125	5
28951_at	28951	ENSG0000(	TRIB2	tribbles homolog 2 (Dr		I	22	-1.37389	-1.31625	-0.68979	-0.80275	-0.57751	7.399413	80.68312	2.70E-06	3.98E-05	5
339487_at	339487	ENSG0000(	ZBTB8OS	zinc finger and BTB doı		I	7	-1.56876	-1.3632	-0.48167	-0.79599	-0.41165	9.445346	89.48563	1.85E-06	3.07E-05	5
8813_at	8813	ENSG0000(	DPM1	dolichyl-phosphate ma		I	9	-1.4369	-1.53758	-0.54656	-0.84308	-0.51497	11.65586	89.14153	1.88E-06	3.09E-05	5
91147_at	91147	ENSG0000(	TMEM67	transmembrane protei		NI	57	-1.24909	-1.44247	-0.53348	-0.74149	-0.50036	3.964754	83.53546	2.38E-06	3.66E-05	5
57717_at	57717	ENSG0000(	PCDHB16	protocadherin beta 16		I	11	-1.36596	-1.32074	-0.53996	-0.77906	-0.5222	6.400333	83.33349	2.40E-06	3.67E-05	5
55764_at	55764	ENSG0000(	IFT122	intraflagellar transport		NI	9	-1.71375	-1.18134	-1.02742	-0.77633	-0.13438	5.145996	108.1332	9.30E-07	1.99E-05	5
91947_at	91947	ENSG0000(	ARRDC4	arrestin domain contai		NI	32	-1.35337	-1.14811	-0.89399	-0.56451	-0.46275	3.841533	60.50877	7.65E-06	8.12E-05	5
10020_at	10020	ENSG0000(	GNE	glucosamir	kinase	I	33	-1.30322	-1.28912	-1.15438	-0.57108	-0.41099	4.577352	73.04575	3.87E-06	5.07E-05	5
114876_at	114876	ENSG0000(	OSBPL1A	oxysterol binding prot		I	22	-1.49515	-1.24622	-0.87603	-0.75962	-0.56071	7.581869	110.9957	8.45E-07	1.88E-05	5
7083_at	7083	ENSG0000(	TK1	thymidine	kinase	I	22	-1.48397	-1.13103	-1.07863	-0.87223	-0.52027	9.582734	70.85184	4.33E-06	5.44E-05	5
3987_at	3987	ENSG0000(	LIMS1	LIM and senescent cell		I	60	-1.16837	-1.19642	-1.0739	-1.0512	-0.3082	8.73503	118.1995	6.72E-07	1.60E-05	5
57018_at	57018	ENSG0000(	CCNL1	cyclin L1	Secreted	I	32	-1.14583	-1.35859	-0.96182	-0.8061	-0.39882	8.181877	94.36811	1.53E-06	2.70E-05	5
6259_at	6259	ENSG0000(	RYK	RYK recept	kinase	I	6	-1.21526	-1.33261	-1.00276	-0.80048	-0.53693	9.038303	111.4352	8.33E-07	1.86E-05	5
389136_at	389136	ENSG0000(	VGLL3	vestigial like 3 (Drosop		I	22	-1.5171	-1.38655	-1.0108	-0.95486	-0.29423	4.300129	30.95058	8.28E-05	0.000487	5
79733_at	79733	ENSG0000(	E2F8	E2F transcr	Secreted	I	32	-1.40446	-1.28323	-0.727	-0.94516	-0.22996	5.340572	58.88679	8.43E-06	8.72E-05	5
22856_at	22856	ENSG0000(	CHSY1	chondroitir	Secreted	I	31	-1.26956	-1.26469	-0.86502	-0.94654	-0.3816	4.32825	93.2523	1.60E-06	2.78E-05	5
1410_at	1410	ENSG0000(	CRYAB	crystallin, alpha B		NI	11	-0.9942	0.231639	-1.69872	-0.40012	-0.81049	4.115825	46.22149	2.01E-05	0.000165	5
256281_at	256281	ENSG0000(	NUDT14	nudix (nucl	Secreted	NI	8	-1.42807	0.016307	-1.86446	-0.78333	-1.33377	5.077928	53.82922	1.16E-05	0.00011	5
100132288	1E+08	NA	TEKT4P2	tektin 4 pseudogene 2		NI	6	-1.03825	-0.051	-1.34721	-0.72019	-1.31594	4.262812	71.36414	4.21E-06	5.37E-05	5
80153_at	80153	ENSG0000(	EDC3	enhancer of mRNA dec		NI	22	-0.8633	-0.26536	-1.11464	-0.59211	-0.95707	6.255699	37.25182	4.32E-05	0.000294	5
6522_at	6522	ENSG0000(	SLC4A2	solute carri	transporte	I	13	-0.80649	-0.47672	-1.34151	-0.74532	-1.0816	6.370313	23.69811	0.000209	0.001003	5
260429_at	260429	ENSG0000(	PRSS33	protease, s	peptidase	I	11	-2.02198	-0.70257	-1.48486	-0.69695	-1.19323	3.791058	145.9234	3.10E-07	9.71E-06	5
333_at	333	ENSG0000(	APLP1	amyloid beta (A4) prec		NI	31	-1.39175	-0.7544	-1.04503	-0.70442	-1.54587	4.471921	51.23762	1.39E-05	0.000125	5
2788_at	2788	ENSG0000(	GNG7	guanine nu	Secreted	NI	32	-1.55932	-0.76969	-0.96585	-0.56919	-0.95079	4.363052	84.67945	2.27E-06	3.55E-05	5
92922_at	92922	ENSG0000(	CCDC102A	coiled-coil domain con		NI	11	-1.47051	-0.57025	-1.14311	-0.68206	-1.1895	5.397142	62.98739	6.62E-06	7.37E-05	5
150368_at	150368	ENSG0000(	FAM109B	family with sequence s		NI	22	-1.47128	-0.75264	-1.48258	-0.78824	-0.98199	4.541599	42.33363	2.74E-05	0.000209	5
441161_at	441161	ENSG0000(	OOEP	oocyte expressed prot		NI	7	-1.48515	-0.71911	-1.3999	-0.90846	-0.96757	4.009748	35.79394	4.97E-05	0.000328	5
29926_at	29926	ENSG0000(	GMPPA	GDP-mannose pyropho		I	18	-1.44766	-0.81105	-1.24794	-0.67715	-1.21956	6.209969	94.60582	1.51E-06	2.68E-05	5
2134_at	2134	ENSG0000(	EXTL1	exostoses (	Secreted	NI	10	-1.6036	-0.85168	-1.36867	-0.70574	-1.21095	4.399873	46.01008	2.04E-05	0.000166	5
255324_at	255324	ENSG0000(	EPGN	epithelial n	kinase	I	57	-0.29391	-0.49683	-0.33541	-0.97679	-1.73345	3.743416	44.46287	2.31E-05	0.000182	5
1437_at	1437	ENSG0000(	CSF2	colony stir	Secreted	I	11	-0.61875	-0.42058	-0.29688	-1.26278	-1.48739	8.428589	24.90258	0.000176	0.000875	5
10522_at	10522	ENSG0000(	DEAF1	deformed (	Secreted	I	11	-0.32804	-0.21748	-0.29279	-0.6005	-1.04996	7.687608	26.82428	0.000136	0.000716	5
23643_at	23643	ENSG0000(	LY96	lymphocyt	Secreted	I	9	-0.43931	-0.35876	-0.26381	-0.60613	-1.12507	6.942696	20.98083	0.000316	0.001399	5
388389_at	388389	ENSG0000(	CCDC103	coiled-coil domain con		NI	11	-0.61986	-0.1943	-0.38249	-0.67137	-1.08442	3.748872	14.42898	0.001101	0.003903	5
5600_at	5600	ENSG0000(	MAPK11	mitogen-ac	kinase	NI	18	-0.47109	-0.38758	-0.50057	-0.66453	-1.21148	3.89604	25.15842	0.00017	0.000851	5
51061_at	51061	ENSG0000(	TXNDC11	thioredoxin domain co		NI	11	-0.5736	-0.29498	-0.50933	-0.72887	-1.14039	6.13805	21.63846	0.000285	0.001286	5
8535_at	8535	ENSG0000(	CBX4	chromobox homolog 4		NI	22	-0.46369	-0.58253	-0.4121	-0.54927	-1.00201	3.920556	30.85841	8.36E-05	0.00049	5
726_at	726	ENSG0000(	CAPN5	calpain 5	peptidase	NI	21	-0.63935	-0.45475	-0.4498	-0.47574	-1.0373	5.040113	14.74423	0.001026	0.003685	5
4982_at	4982	ENSG0000(	TNFRSF11E	tumor necr	Secreted	NI	11	-0.68393	-0.48588	-0.22607	-0.40196	-1.05671	6.521319	27.54245	0.000124	0.000668	5
5580_at	5580	ENSG0000(	PRKCD	protein kin	kinase	NI	21	-0.66645	-0.71855	-0.30796	-0.41838	-1.15235	6.886818	35.98576	4.88E-05	0.000323	5
91300_at	91300	ENSG0000(	C19orf22	chromosome 19 open		NI	31	-0.38859	-0.65775	-0.4006	-0.35677	-1.16812	6.270547	21.46075	0.000293	0.001314	5
3489_at	3489	ENSG0000(	IGFBP6	insulin-like	Secreted	I	10	-0.40375	-0.73487	-0.29299	-0.65518	-1.11449	8.382042	27.96354	0.000118	0.000641	5
5973_at	5973	ENSG0000(	RENBP	renin binding protein		NI	11	-0.48159	-0.59471	-0.40683	-0.64005	-1.35152	3.673136	29.30469	0.0001	0.000566	5
6242_at	6242	ENSG0000(	RTKN	rhotekin		NI	33	-0.43472	-0.70074	-0.59057	-0.67491	-1.34024	4.93218	41.90553	2.85E-05	0.000215	5
113655_at	113655	ENSG0000(	MFSD3	major facilitator super		NI	9	-0.35242	-0.86493	-0.9835	-0.53482	-1.33677	5.885299	37.9334	4.05E-05	0.00028	5
285613_at	285613	ENSG0000(	RELL2	RELT-like 2		NI	14	-0.34538	-0.6841	-0.6965	-0.49066	-1.1624	5.190724	14.73165	0.001029	0.003693	5
29952_at	29952	ENSG0000(	DPP7	dipeptidyl-	peptidase	NI	31	-0.44171	-0.82769	-0.69391	-0.40692	-1.02372	6.6869	50.8769	1.43E-05	0.000128	5
55687_at	55687	ENSG0000(	TRMU	tRNA 5-methylaminon		NI	35	-0.22756	-0.17104	-0.78732	-0.55063	-1.01229	6.664776	34.10471	5.89E-05	0.000374	5
57140_at	57140	ENSG0000(	RNPEPL1	arginyl ami	kinase	NI	11	-0.45446	-0.34311	-0.73202	-0.43841	-1.0073	4.732362	24.70808	0.000181	0.000894	5
54480_at	54480	ENSG0000(	CHPF2	chondroitin polymerizi		I	20	-0.3628	-0.34873	-0.68406	-0.51692	-1.12599	6.662063	49.24634	1.60E-05	0.00014	5
64118_at	64118	ENSG0000(	DUS1L	dihydrouridine synthas		NI	16	-0.28642	-0.38893	-0.94577	-0.59405	-1.05959	7.093181	49.00014	1.63E-05	0.000142	5
27132_at	27132	ENSG0000(	CPNE7	copine VII	transporte	NI	22	-0.38067	-0.29829	-0.90261	-0.61007	-1.09327	5.411679	21.10417	0.00031	0.001379	5
54498_at	54498	ENSG0000(	SMOX	spermine oxidase		NI	39	-0.46425	-0.34735	-0.8921	-0.53443	-1.07945	4.244362	32.20562	7.20E-05	0.000437	5
26469_at	26469	ENSG0000(	PTPN18	protein tyr	phosphopr	NI	33	-0.49854	-0.40217	-0.86303	-0.62577	-1.03559	4.843756	45.21924	2.17E-05	0.000175	5
5213_at	5213	ENSG0000(	PFKM	phosphofr	kinase	I	10	-0.43076	-0.62033	-0.79212	-0.85557	-1.00105	9.000231	28.63504	0.000109	0.000602	5
7004_at	7004	ENSG0000(	TEAD4	TEA domain family me		I	10	-0.41682	-0.53845	-0.82905	-0.70042	-1.05284	8.053098	36.48175	4.65E-05	0.000311	5
403313_at	403313	ENSG0000(	PPAPDC2	phosphatidic acid phos		NI	22	-0.37816	-0.60116	-0.86385	-0.66327	-1.073	4.533537	65.43893	5.77E-06	6.71E-05	5
5026_at	5026	NA	P2RX5	purinergic	ion channe	NI	20	-0.26512	-0.37712	-0.59087	-0.78174	-1.05352	5.927758	53.47265	1.19E-05	0.000112	5
220323_at	220323	ENSG0000(	OAF	OAF homol	Secreted	I	22	-0.31147	-0.29654	-0.72863	-0.71639	-1.17291	8.413465	46.99823	1.89E-05	0.000158	5
9409_at	9409	ENSG0000(	PEX16	peroxisomal biogenesi		NI	20	-0.31426	-0.38046	-0.80777	-0.83132	-1.11526	5.975125	27.33181	0.000128	0.000681	5
29985_at	29985	ENSG0000(	SLC39A3	solute carri	transporte	NI	27	-0.41063	-0.3482	-0.87089	-0.78308	-1.07854	4.752267	43.86743	2.42E-05	0.000189	5
80256_at	80256	ENSG0000(	KIAA1539	KIAA1539		I	9	-0.28508	-0.83231	-0.59454	-0.79215	-1.25931	6.957859	70.36797	4.43E-06	5.54E-05	5
3554_at	3554	ENSG0000(	IL1R1	interleukin	Secreted	I	22	-0.17988	-1.03539	-0.68636	-0.883	-1.26728	4.057844	39.79134	3.42E-05	0.000247	5

23464_at	23464	ENSG0000(	GCAT	glycine C-acetyltransfe		NI	7	-0.17121	-0.94872	-0.85307	-0.9084	-1.30124	6.614077	39.97134	3.36E-05	0.000244	5
190_at	190	ENSG0000(	NR0B1	nuclear rec	nuclear rec	NI	21	-0.324	-0.48779	-0.72394	-1.16909	-1.28396	3.738201	27.82463	0.00012	0.00065	5
1608_at	1608	ENSG0000(	DGKG	diacylglyce	kinase	I	41	-0.53865	-0.61807	-0.56858	-1.17346	-1.39966	3.860314	55.73921	1.03E-05	0.000101	5
401647_at	401647	ENSG0000(	GOLGA7B	golgin A7 family, mem		NI	11	-0.57339	-0.70605	-0.86472	-1.17925	-1.4233	4.02221	130.2169	4.71E-07	1.28E-05	5
284114_at	284114	ENSG0000(	TMEM102	transmembrane protei		I	11	-0.45022	-1.12757	-1.31327	-0.97724	-1.5145	5.497043	54.43836	1.12E-05	0.000107	5
92399_at	92399	ENSG0000(	MRRF	mitochondrial ribosom		I	11	-0.41054	-1.06162	-0.94595	-0.94126	-1.17744	6.984151	30.78956	8.43E-05	0.000493	5
9610_at	9610	ENSG0000(	RIN1	Ras and Rab interactor		NI	56	-0.58423	-1.14325	-0.89278	-0.95034	-1.10125	5.067623	102.9078	1.11E-06	2.22E-05	5
115399_at	115399	ENSG0000(	LRRC56	leucine rich repeat con		NI	11	-0.70578	-1.08669	-0.70131	-1.07252	-1.40343	5.374858	30.67269	8.54E-05	0.000498	5
2184_at	2184	ENSG0000(	FAH	fumarylacetoacetate h		I	9	-0.69925	-0.96034	-0.81584	-0.95941	-1.23971	9.03795	44.79988	2.24E-05	0.000179	5
5799_at	5799	ENSG0000(	PTPRN2	protein tyr	phosphopr	I	30	-0.73174	-1.0324	-0.83861	-1.08766	-1.24119	4.133896	128.4718	4.95E-07	1.31E-05	5
23500_at	23500	ENSG0000(	DAAM2	dishevelled associated		NI	20	-0.74597	-0.88983	-1.11285	-0.85616	-1.32762	4.924407	121.6989	6.04E-07	1.50E-05	5
401093_at	401093	NA	LOC40109	hypothetical LOC40109		NI	11	-0.74986	-0.90846	-0.95862	-0.87845	-1.23048	4.390651	53.74288	1.17E-05	0.000111	5
409_at	409	ENSG0000(	ARRB2	arrestin, beta 2		NI	27	-0.85228	-0.87325	-1.07231	-0.81963	-1.21177	4.965617	24.00977	0.0002	0.000967	5
10073_at	10073	ENSG0000(	SNUPN	snurportin	transporter	I	11	-0.92328	-0.86988	-0.9985	-0.86769	-1.23862	8.479508	78.29382	3.01E-06	4.29E-05	5
79701_at	79701	ENSG0000(	C17orf101	chromosome 17 open		I	19	-0.89429	-0.89761	-0.9743	-0.93848	-1.41276	6.833308	71.50713	4.18E-06	5.35E-05	5
117144_at	117144	ENSG0000(	CATSPER1	cation char	ion channe	I	10	-0.92114	-0.91589	-1.00416	-1.09821	-1.30971	6.185132	33.22312	6.46E-05	0.000401	5
5045_at	5045	ENSG0000(	FURIN	furin (paire	peptidase	NI	11	-0.78634	-0.96989	-1.1075	-1.01515	-1.19775	6.084828	20.24061	0.000357	0.001545	5
23704_at	23704	ENSG0000(	KCNE4	potassium	ion channe	NI	22	-0.85633	-1.00179	-0.95579	-1.01786	-1.16444	4.093693	102.891	1.12E-06	2.22E-05	5
54751_at	54751	ENSG0000(	FBLIM1	filamin binding LIM pro		I	33	-0.81519	-1.06771	-1.06265	-0.98867	-1.24586	6.940545	104.5226	1.05E-06	2.14E-05	5
706_at	706	ENSG0000(	TSPO	translocatc	Secreted	I	32	-0.89802	-1.07348	-1.059	-0.99774	-1.20398	11.66759	97.55332	1.35E-06	2.48E-05	5
51524_at	51524	ENSG0000(	TMEM138	transmembrane protei		I	11	-0.53715	-0.75499	-1.17947	-0.92898	-0.98156	8.664592	73.42854	3.80E-06	5.00E-05	5
83986_at	83986	ENSG0000(	ITFG3	integrin alpha FG-GAP		I	11	-0.57929	-0.58773	-1.15843	-0.80412	-0.98374	7.108226	41.20502	3.02E-05	0.000225	5
148304_at	148304	ENSG0000(	C1orf74	chromosome 1 open re		NI	11	-0.65691	-0.69299	-1.16111	-0.77659	-0.93244	4.928188	49.76098	1.54E-05	0.000136	5
6721_at	6721	ENSG0000(	SREBF2	sterol regulatory elem		I	20	-0.53982	-0.52779	-1.06156	-0.89771	-0.91964	5.531633	27.40015	0.000127	0.000677	5
60386_at	60386	ENSG0000(	SLC25A19	solute carri	transporter	I	21	-0.55783	-0.64736	-1.0536	-0.88502	-0.87969	7.104227	48.0853	1.74E-05	0.00015	5
85474_at	85474	ENSG0000(	LBX2	ladybird homeobox 2		NI	22	-0.43504	-0.61173	-1.22978	-0.88716	-0.7691	4.534592	65.49436	5.75E-06	6.69E-05	5
10300_at	10300	ENSG0000(	KATNB1	katanin p80 (WD repea		NI	16	-0.45822	-0.54705	-1.1911	-0.98311	-0.92902	5.399089	67.69265	5.10E-06	6.13E-05	5
64236_at	64236	ENSG0000(	PDLIM2	PDZ and LIM domain 2		I	21	-0.70262	-0.5485	-1.2592	-0.9225	-0.71127	3.734062	67.37779	5.19E-06	6.20E-05	5
51172_at	51172	ENSG0000(	NAGPA	N-acetylglu	Secreted	NI	11	-0.74264	-0.40194	-1.27982	-0.90541	-0.85272	5.843929	19.23601	0.000424	0.001783	5
283687_at	283687	NA	C15orf37	chromosome 15 open		NI	20	-1.05537	-0.49788	-1.26958	-0.80523	-0.74647	3.727433	33.37069	6.36E-05	0.000397	5
25966_at	25966	ENSG0000(	C2CD2	C2 calcium-dependent		I	22	-1.05745	-0.52223	-1.25611	-0.7912	-0.83514	6.193469	44.50996	2.30E-05	0.000182	5
64425_at	64425	ENSG0000(	POLR1E	polymerase (RNA) I po		I	32	-0.92373	-0.8404	-1.21991	-0.98266	-0.72946	8.69093	77.73355	3.09E-06	4.36E-05	5
9289_at	9289	ENSG0000(	GPR56	G protein-c	G-protein c	I	33	-0.98272	-0.82417	-1.33339	-0.87853	-0.78939	5.287239	76.67736	3.25E-06	4.51E-05	5
649_at	649	ENSG0000(	BMP1	bone morp	peptidase	NI	42	-0.74775	-0.93355	-1.35246	-0.89725	-0.68632	4.465146	54.73279	1.10E-05	0.000106	5
9631_at	9631	ENSG0000(	NUP155	nucleopori	Secreted	I	9	-0.78519	-0.70584	-1.22723	-0.90309	-0.63466	7.841668	47.12211	1.87E-05	0.000157	5
29886_at	29886	ENSG0000(	SNX8	sorting nexin 8		I	8	-0.85156	-0.82786	-1.21481	-0.88767	-0.58743	6.708552	28.48274	0.000111	0.00061	5
23350_at	23350	ENSG0000(	U2SURP	U2 snRNP-associated S		I	29	-0.53963	-0.94966	-0.88229	-1.01157	-0.5017	7.821313	46.46149	1.97E-05	0.000162	5
9648_at	9648	ENSG0000(	GCC2	GRIP and coiled-coil dc		I	43	-0.72778	-0.8782	-1.10451	-1.02944	-0.50538	7.581101	89.95714	1.82E-06	3.03E-05	5
9392_at	9392	ENSG0000(	TGFBRAP1	transforming growth fa		NI	11	-0.65555	-0.83791	-1.12931	-1.23065	-0.76371	4.742621	45.61132	2.11E-05	0.000171	5
10082_at	10082	ENSG0000(	GPC6	glypican 6	Secreted	NI	32	-0.65609	-0.71541	-0.897	-1.08891	-0.66305	4.829645	19.37493	0.000414	0.001744	5
286128_at	286128	ENSG0000(	ZFP41	zinc finger protein 41 h		NI	8	-0.5156	-0.62897	-1.05159	-1.24476	-0.65783	5.382577	15.305	0.000908	0.003326	5
56257_at	56257	ENSG0000(	MEPCE	methylphosphate capp		I	11	-0.6338	-0.94085	-1.0343	-0.54325	-0.42396	7.845527	50.82089	1.43E-05	0.000128	5
11342_at	11342	ENSG0000(	RNF13	ring finger protein 13		I	10	-0.76032	-0.7979	-1.07699	-0.53699	-0.62891	8.52809	45.08935	2.19E-05	0.000177	5
25871_at	25871	ENSG0000(	C3orf17	chromosome 3 open re		I	72	-0.85924	-0.82834	-1.01349	-0.55783	-0.56097	6.608603	45.63557	2.10E-05	0.000171	5
79640_at	79640	ENSG0000(	C22orf46	chromosome 22 open		NI	11	-0.77179	-0.60323	-1.21726	-0.67976	-0.61448	5.219308	61.26525	7.31E-06	7.89E-05	5
64753_at	64753	ENSG0000(	CCDC136	coiled-coil domain con		NI	11	-0.81171	-0.54606	-1.02118	-0.67664	-0.44408	4.006131	30.4478	8.77E-05	0.000509	5
91523_at	91523	ENSG0000(	FAM113B	family with sequence s		NI	11	-0.7987	-0.53329	-1.17161	-0.79792	-0.47866	4.677467	61.22523	7.33E-06	7.90E-05	5
64601_at	64601	ENSG0000(	VPS16	vacuolar protein sortin		I	6	-0.7739	-0.64876	-1.02234	-0.64548	-0.8348	7.843348	29.68685	9.57E-05	0.000546	5
51335_at	51335	ENSG0000(	NGRN	neugrin, neurite outgro		I	10	-0.74543	-0.74605	-1.05192	-0.72977	-0.79756	10.77109	58.21576	8.79E-06	8.99E-05	5
79621_at	79621	ENSG0000(	RNASEH2B	ribonuclease H2, subu		I	19	-0.85945	-0.6928	-1.07565	-0.69459	-0.64334	7.642383	58.55769	8.60E-06	8.84E-05	5
55174_at	55174	ENSG0000(	INTS10	integrator complex sub		I	20	-0.91279	-0.77908	-1.00287	-0.72597	-0.73879	8.442997	61.722	7.12E-06	7.75E-05	5
80228_at	80228	ENSG0000(	ORAI2	ORAI calcium release-a		NI	44	-0.52639	-0.72151	-1.05177	-0.7794	-0.56322	4.233468	25.66976	0.000159	0.000804	5
221458_at	221458	ENSG0000(	KIF6	kinesin family member		NI	32	-0.58899	-0.85104	-1.04544	-0.89962	-0.56403	3.948849	27.42508	0.000126	0.000677	5
7791_at	7791	ENSG0000(	ZYX	zyxin		NI	22	-0.62245	-0.75751	-1.03611	-0.87457	-0.85363	6.009897	42.57838	2.69E-05	0.000206	5
5433_at	5433	ENSG0000(	POLR2D	polymerase (RNA) II (D		I	19	-0.49016	-0.82286	-1.09599	-0.92954	-0.81305	6.682521	87.94564	1.98E-06	3.21E-05	5
130612_at	130612	ENSG0000(	TMEM198	transmembrane protei		NI	11	-0.47144	-1.09177	-1.07086	-0.79245	-0.59374	4.707951	53.75028	1.17E-05	0.000111	5
79690_at	79690	ENSG0000(	GAL3ST4	galactose-3	Secreted	NI	22	-0.65127	-0.8947	-1.04288	-0.85439	-0.74055	4.167955	45.83438	2.07E-05	0.000169	5
10806_at	10806	ENSG0000(	SDCCAG8	serologically defined c		I	20	-0.71617	-0.98689	-1.01125	-0.89928	-0.74888	6.089269	21.14865	0.000308	0.001371	5
1879_at	1879	ENSG0000(	EBF1	early B-cell factor 1		NI	31	-0.59846	-1.01534	-0.79817	-0.8866	-0.71636	3.833217	27.54373	0.000124	0.000668	5
64921_at	64921	ENSG0000(	CASD1	CAS1 domain containir		NI	19	-0.63345	-1.01586	-0.83983	-0.80079	-0.58845	5.443747	43.11622	2.57E-05	0.000199	5
9276_at	9276	ENSG0000(	COPB2	coatomer protein com		I	21	-0.69146	-1.05507	-0.82296	-0.77163	-0.68777	7.048115	48.65528	1.67E-05	0.000145	5
27122_at	27122	ENSG0000(	DKK3	dickkopf hc	Secreted	NI	44	-0.53322	-0.56341	-1.26607	-0.75447	-1.27874	4.452826	31.2372	8.02E-05	0.000475	5
3215_at	3215	ENSG0000(	HOXB5	homeobox B5		NI	25	-0.59922	-0.56348	-1.12537	-0.70372	-1.0941	4.119898	83.37077	2.40E-06	3.67E-05	5
393_at	393	ENSG0000(	ARHGAP4	Rho GTPase activating		NI	42	-0.58949	-0.66686	-1.16283	-0.61658	-1.19188	4.550444	44.53399	2.29E-05	0.000182	5
91373_at	91373	ENSG0000(	UAP1L1	UDP-N-acteylglucosam		NI	11	-0.84204	-0.81271	-1.31677	-0.56823	-1.37681	5.449448	71.01864	4.29E-06	5.42E-05	5
9159_at	9159	ENSG0000(	PCSK7	proprotein	peptidase	NI	11	-0.71005	-0.84292	-1.22121	-0.58769	-1.03132	6.593547	33.09795	6.55E-05	0.000405	5
6988_at	6988	ENSG0000(	TCTA	T-cell leukemia translo		I	21	-0.66867	-0.98033	-1.30021	-0.79513	-1.09382	6.740424	94.54284	1.52E-06	2.68E-05	5
29927_at	29927	ENSG0000(	SEC61A1	Sec61 alph	transporter	I	32	-0.84536	-0.85494	-1.24593	-0.74659	-1.16439	7.811203	72.69105	3.94E-06	5.13E-05	5
6296_at	6296	ENSG0000(	ACSM3	acyl-CoA synthetase m		NI	16	-0.8811	-0.64439	-0.90571	-1.16427	-1.08247	4.056203	39.95481	3.37E-05	0.000244	5
64708_at	64708	ENSG0000(	COPS7B	COP9 constitutive pho		I	11	-0.61936	-0.48264	-1.08631	-1.01318	-1.15149	6.407837	58.82239	8.47E-06	8.74E-05	5
2548_at	2548	ENSG0000(	GAA	glucosidase	Secreted	NI	11	-0.81724	-0.55236	-1.15552	-1.05265	-1.10844	5.669602	64.20815	6.17E-06	7.03E-05	5
84331_at	84331	ENSG0000(	FAM195A	family with sequence s		I	7	-0.62303	-0.5921	-0.97571	-0.8305	-1.45009	6.89652	67.14507	5.25E-06	6.26E-05	5
27037_at	27037	ENSG0000(	TRMT2A	TRM2 tRNA methyltran		I	43	-0.58695	-0.73763	-1.02247	-0.84787	-1.40833	5.491246	79.21025	2.89E-06	4.16E-05	5
83985_at	83985	ENSG0000(	SPNS1	spinster hc	transporter	I	23	-0.88422	-0.64641	-0.99055	-0.8775	-1.29295	5.563893	81.21134	2.64E-06	3.91E-05	5
83642_at	83642	ENSG0000(	SELO	selenoprotein O		I	11	-0.90704	-0.61061	-1.01499	-0.78361	-1.44228	6.570161	78.66132	2.96E-06	4.23E-05	5
23583_at	23583	ENSG0000(	SMUG1	single-strar	Secreted	I	21	-0.86504	-0.67236	-1.0305	-1.11669	-1.36178	6.127167	131.5465	4.54E-07	1.24E-05	5
5524_at	5524	ENSG0000(	PPP2R4	protein phosphatase 2		NI	22	-0.88409	-0.75508	-0.84256	-0.98917	-1.28278	5.406038	41.77362	2.88E-05	0.000217	5
27351_at	27351	ENSG0000(	PPPDE2	PPPDE peptidase doma		I	18	-0.84879	-0.7954	-0.94595	-0.9264	-1.3158	7.668778	98.74864	1.30E-06	2.41E-05	5
8349_at	8349	ENSG0000(	HIST2H2BE	histone cluster 2, H2be		NI	11	-0.5477	-0.89819	-1.01419	-0.78954	-1.02778	4.471064	39.52612	3.50E-05	0.000251	5
1454_at	1454	ENSG0000(	CSNK1E	casein kina	kinase	I	35	-0.65226	-0.81459	-1.02356	-0.81794	-1.01568	8.636921	91.24352	1.73E-06	2.94E-05	5
65057_at	65057	ENSG0000(	ACD	adrenocortical dysplas		I	19	-0.70329	-0.87145	-1.05532	-0.75808	-0.97559	5.938546	91.14788	1.73E-06	2.94E-05	5
64852_at	64852	NA	TUT1	terminal uridylyl trans		NI	11	-0.61605	-0.7521	-1.02639	-0.66059	-1.25922	5.558744	36.76039	4.52E-05	0.000305	5

9900_at	9900	ENSG0000(	SV2A	synaptic ve	transporter	NI	27	-0.68749	-0.99009	-1.00897	-0.66439	-1.15977	3.927324	86.20475	2.12E-06	3.39E-05	5
8733_at	8733	NA	GPAA1	glycosylphosphatidylin		I	14	-0.59009	-0.98188	-0.92075	-0.79281	-1.26948	6.833783	59.32561	8.21E-06	8.54E-05	5
8818_at	8818	ENSG0000(	DPM2	dolichyl-phosphate ma		I	11	-0.75955	-0.59517	-0.91944	-0.87857	-1.03547	8.929333	60.92643	7.46E-06	7.99E-05	5
138428_at	138428	ENSG0000(	PTRH1	peptidyl-tRNA hydrola		I	11	-0.63751	-0.72792	-0.8927	-0.8806	-1.10005	7.961932	20.17742	0.000361	0.001559	5
2114_at	2114	ENSG0000(	ETS2	v-ets erythroblastosis v		I	21	-0.65291	-0.6304	-0.93416	-0.97773	-1.09279	7.665797	20.71651	0.00033	0.001451	5
90196_at	90196	NA	SYS1	SYS1 Golgi-localized in		I	22	-0.63243	-0.7297	-0.6816	-0.74854	-1.01848	6.561832	57.37178	9.26E-06	9.33E-05	5
59271_at	59271	ENSG0000(	C21orf63	chromosome 21 open		NI	19	-0.61251	-0.6609	-0.77527	-0.74439	-1.0767	6.043845	49.25032	1.60E-05	0.00014	5
85014_at	85014	ENSG0000(	TMEM141	transmembrane protei		I	6	-0.63489	-0.82251	-0.74129	-0.84125	-1.01804	8.296127	58.62525	8.57E-06	8.82E-05	5
23170_at	23170	ENSG0000(	TTLL12	tubulin tyrosine ligase-		I	26	-0.69696	-0.81547	-0.79745	-0.86706	-1.07592	6.117139	91.54037	1.71E-06	2.91E-05	5
64320_at	64320	ENSG0000(	RNF25	ring finger protein 25		I	11	-0.68096	-0.82916	-0.87064	-0.8426	-1.10423	6.934563	63.72138	6.35E-06	7.17E-05	5
51650_at	51650	ENSG0000(	MRPS33	mitochondrial ribosom		I	11	-0.7411	-0.81632	-0.90663	-0.6961	-1.01395	9.558601	55.65283	1.03E-05	0.000101	5
51078_at	51078	ENSG0000(	THAP4	THAP domain containi		I	10	-0.71546	-0.68813	-0.9815	-0.74238	-1.09831	9.291738	59.371	8.19E-06	8.53E-05	5
23192_at	23192	ENSG0000(	ATG4B	ATG4 autop	peptidase	NI	55	-0.78239	-0.62687	-0.75488	-0.69105	-1.04961	6.490013	39.2895	3.58E-05	0.000255	5
84836_at	84836	ENSG0000(	ABHD14B	abhydrolase domain co		NI	21	-0.84161	-0.66122	-0.80774	-0.77633	-1.02212	5.117567	44.82765	2.24E-05	0.000179	5
2987_at	2987	ENSG0000(	GUK1	guanylate k	kinase	I	48	-0.7405	-0.68107	-0.82703	-0.80827	-1.05571	10.2303	65.60281	5.71E-06	6.67E-05	5
513_at	513	ENSG0000(	ATP5D	ATP syntha	transporter	I	32	-0.74821	-0.6414	-0.78803	-0.75137	-1.08522	9.701341	70.72626	4.35E-06	5.47E-05	5
977_at	977	ENSG0000(	CD151	CD151 molecule (Raph		I	54	-0.77227	-0.65672	-0.82067	-0.73852	-1.03266	8.990844	57.55737	9.15E-06	9.27E-05	5
11068_at	11068	ENSG0000(	CYB561D2	cytochrome b-561 don		I	11	-0.65908	-0.87132	-1.01367	-1.08241	-1.10536	8.327768	81.88148	2.56E-06	3.83E-05	5
10195_at	10195	ENSG0000(	ALG3	asparagine-linked glyco		I	16	-0.5858	-0.82935	-1.21629	-1.08815	-1.14426	8.205745	91.80442	1.69E-06	2.89E-05	5
55657_at	55657	ENSG0000(	ZNF692	zinc finger protein 692		I	30	-0.81374	-0.87761	-1.37032	-0.93591	-1.03163	6.657397	63.70861	6.35E-06	7.17E-05	5
471_at	471	ENSG0000(	ATIC	5-aminoimidazole-4-ca		I	9	-0.75368	-0.93625	-1.20262	-0.93744	-0.9268	9.842524	87.07409	2.05E-06	3.30E-05	5
57168_at	57168	ENSG0000(	ASPHD2	aspartate beta-hydroxy		NI	22	-0.87001	-0.90428	-1.25513	-0.89525	-0.9009	5.436134	44.49414	2.30E-05	0.000182	5
26001_at	26001	ENSG0000(	RNF167	ring finger protein 167		I	31	-0.86806	-0.94554	-1.27314	-1.05656	-1.04124	6.763863	101.3495	1.18E-06	2.29E-05	5
157697_at	157697	ENSG0000(	ERICH1	glutamate-rich 1		I	39	-0.84817	-0.93922	-1.25901	-1.13148	-1.07515	7.595773	91.49916	1.71E-06	2.91E-05	5
23378_at	23378	ENSG0000(	RRP8	ribosomal RNA proces		I	19	-0.75014	-0.84118	-1.23263	-1.05546	-1.16957	6.222907	50.4863	1.46E-05	0.00013	5
171568_at	171568	ENSG0000(	POLR3H	polymerase (RNA) III (D		I	38	-0.76218	-0.92779	-1.13501	-1.05431	-1.10258	6.814024	88.21825	1.95E-06	3.18E-05	5
79587_at	79587	ENSG0000(	CARS2	cysteinyl-tRNA synthet		I	15	-0.76799	-0.88542	-1.15592	-1.1198	-1.07276	6.839421	19.85023	0.000381	0.001634	5
113_at	113	ENSG0000(	ADCY7	adenylate cyclase 7		NI	31	-0.72264	-1.0907	-0.91924	-1.07786	-1.00198	5.416587	33.33109	6.39E-05	0.000397	5
55798_at	55798	ENSG0000(	METTL2B	methyltransferase like		I	18	-0.79742	-1.03432	-1.00972	-1.14796	-1.04891	6.778383	131.6201	4.53E-07	1.24E-05	5
9467_at	9467	ENSG0000(	SH3BP5	SH3-domain binding pr		I	22	-0.64719	-1.15062	-1.1556	-1.15758	-0.95682	7.685579	118.8877	6.58E-07	1.58E-05	5
2055_at	2055	ENSG0000(	CLN8	ceroid-lipofuscinosis, n		NI	43	-0.63566	-1.07042	-1.07266	-1.04309	-1.10031	5.233085	92.54362	1.64E-06	2.84E-05	5
10642_at	10642	ENSG0000(	IGF2BP1	insulin-like growth fact		NI	25	-0.7229	-1.05948	-1.17086	-1.03688	-1.06605	4.277338	38.32346	3.91E-05	0.000273	5
55756_at	55756	ENSG0000(	INTS9	integrator complex sub		I	32	-0.79023	-1.08009	-1.03265	-0.83413	-1.12514	5.722232	68.68244	4.84E-06	5.90E-05	5
219_at	219	ENSG0000(	ALDH1B1	aldehyde dehydrogena		I	22	-0.79261	-1.15904	-1.16948	-0.81775	-1.01019	6.29375	89.0396	1.89E-06	3.10E-05	5
64792_at	64792	ENSG0000(	RABL5	RAB, member RAS onc		I	55	-0.84794	-0.99108	-1.11286	-0.96816	-1.00611	7.042801	74.09672	3.68E-06	4.90E-05	5
256355_at	256355	NA	RPS2P32	ribosomal protein S2 p		I	22	-0.8256	-0.93929	-1.1252	-0.91747	-1.08835	5.202048	84.00307	2.33E-06	3.63E-05	5
25894_at	25894	ENSG0000(	PLEKHG4	pleckstrin homology do		I	11	-0.73217	-0.97201	-1.08552	-0.89225	-1.07436	6.940025	83.3815	2.40E-06	3.67E-05	5
374875_at	374875	ENSG0000(	HSD11B1L	hydroxyste	Secreted	NI	20	-0.76018	-1.0149	-1.15409	-0.90979	-1.05054	4.808079	56.23072	9.95E-06	9.85E-05	5
84942_at	84942	ENSG0000(	WDR73	WD repeat domain 73		I	21	-0.62801	-1.03942	-0.9164	-0.92347	-0.88819	6.057179	99.4969	1.26E-06	2.38E-05	5
2274_at	2274	ENSG0000(	FHL2	four and a half LIM dor		I	33	-0.74452	-1.00792	-0.87385	-0.92067	-0.88408	9.594278	100.6142	1.21E-06	2.33E-05	5
53944_at	53944	ENSG0000(	CSNK1G1	casein kina	kinase	NI	42	-0.79311	-1.05171	-0.89243	-0.88661	-0.82157	5.097475	68.87734	4.79E-06	5.86E-05	5
8607_at	8607	ENSG0000(	RUVBL1	RuvB-like 1 (E. coli)		I	30	-0.82693	-0.9995	-1.07111	-0.84312	-0.83204	9.261752	68.40998	4.91E-06	5.97E-05	5
51603_at	51603	ENSG0000(	METTL13	methyltransferase like		I	33	-0.85134	-1.02264	-1.06104	-0.86654	-0.91002	7.573754	95.02723	1.49E-06	2.65E-05	5
84954_at	84954	ENSG0000(	MPND	MPN doma	peptidase	NI	17	-0.71705	-1.1569	-0.97166	-0.87078	-0.89309	5.17799	65.27552	5.82E-06	6.75E-05	5
8888_at	8888	ENSG0000(	MCM3AP	minichromosome mair		I	30	-0.79189	-1.05429	-1.03317	-0.88144	-0.96976	7.516466	62.62634	6.76E-06	7.48E-05	5
255104_at	255104	ENSG0000(	TMCO4	transmembrane and co		I	22	-0.83151	-1.09217	-0.99973	-0.95444	-0.92944	5.990586	74.07206	3.68E-06	4.90E-05	5
7096_at	7096	ENSG0000(	TLR1	toll-like receptor 1		I	11	-1.09314	-0.89709	-1.11882	-0.7717	-1.22306	3.763654	83.77071	2.36E-06	3.64E-05	5
949_at	949	ENSG0000(	SCARB1	scavenger r	transporter	I	20	-0.99174	-1.07221	-1.08777	-0.83784	-1.27065	4.747445	50.64403	1.45E-05	0.000129	5
57085_at	57085	ENSG0000(	AGTRAP	angiotensin II receptor		I	34	-0.95475	-0.84727	-1.18031	-0.92178	-1.3007	6.900812	70.80361	4.34E-06	5.45E-05	5
27237_at	27237	ENSG0000(	ARHGEF16	Rho guanine nucleotid		NI	32	-1.02146	-0.88787	-1.28966	-0.95377	-1.25531	4.46923	51.61426	1.35E-05	0.000123	5
80758_at	80758	ENSG0000(	PRR7	proline rich	Secreted	I	10	-1.07188	-0.98285	-1.24339	-1.01671	-1.1745	6.537165	96.03916	1.43E-06	2.58E-05	5
26470_at	26470	ENSG0000(	SEZ6L2	seizure rela	Secreted	I	41	-1.07468	-0.95357	-1.22105	-1.09015	-1.21099	5.972363	110.8659	8.49E-07	1.88E-05	5
6881_at	6881	ENSG0000(	TAF10	TAF10 RNA polymerase		I	16	-1.27816	-0.88513	-1.18094	-0.96814	-1.20743	10.56148	93.98303	1.55E-06	2.72E-05	5
92856_at	92856	ENSG0000(	IMP4	IMP4, U3 small nucleo		I	10	-1.1762	-0.90113	-1.29148	-1.00489	-1.1661	8.825885	63.23304	6.52E-06	7.30E-05	5
10785_at	10785	ENSG0000(	WDR4	WD repeat	Secreted	NI	41	-1.11113	-0.77354	-1.00679	-0.97919	-1.03089	4.488154	43.76073	2.44E-05	0.000191	5
84303_at	84303	ENSG0000(	CHCHD6	coiled-coil-helix-coiled		I	20	-1.15593	-0.74948	-1.09975	-0.99566	-1.19198	7.760635	47.39043	1.84E-05	0.000155	5
148327_at	148327	ENSG0000(	CREB3L4	cAMP resp	Secreted	I	10	-1.15715	-0.82568	-1.16673	-0.97711	-1.14124	6.46496	101.0629	1.19E-06	2.30E-05	5
10172_at	10172	ENSG0000(	ZNF256	zinc finger protein 256		NI	11	-1.22917	-0.95471	-0.99218	-0.918	-1.16447	4.635997	19.98361	0.000373	0.001603	5
57406_at	57406	ENSG0000(	ABHD6	abhydrolase domain co		I	22	-1.10576	-0.91757	-1.0608	-0.97902	-1.11548	5.110191	118.6234	6.63E-07	1.59E-05	5
252839_at	252839	ENSG0000(	TMEM9	transmembrane protei		I	43	-1.15524	-1.03991	-1.12415	-1.00222	-1.14753	7.864685	95.54404	1.46E-06	2.61E-05	5
4126_at	4126	ENSG0000(	MANBA	mannosida	Secreted	I	22	-1.01703	-0.86343	-0.89963	-0.73353	-0.90909	5.367192	47.37179	1.84E-05	0.000155	5
3704_at	3704	ENSG0000(	ITPA	inosine triphosphatase		I	17	-1.02108	-0.85191	-0.97321	-0.81778	-0.91337	9.1542	93.63037	1.57E-06	2.74E-05	5
134265_at	134265	ENSG0000(	AFAP1L1	actin filament associat		NI	43	-0.90304	-0.98367	-0.92368	-0.71311	-1.04749	4.212072	57.95968	8.93E-06	9.11E-05	5
91582_at	91582	ENSG0000(	RPS19BP1	ribosomal protein S19		I	10	-0.94721	-0.94704	-0.83608	-0.85156	-1.03164	10.41883	96.33628	1.42E-06	2.57E-05	5
7264_at	7264	ENSG0000(	TSTA3	tissue specific transpla		I	34	-0.98277	-0.72134	-0.95146	-0.84263	-1.0809	8.868384	73.05058	3.87E-06	5.07E-05	5
26151_at	26151	ENSG0000(	NAT9	N-acetyltransferase 9 (		I	11	-1.01995	-0.8223	-0.97019	-0.80593	-1.11743	7.297977	35.50557	5.11E-05	0.000336	5
22820_at	22820	ENSG0000(	COPG	coatomer protein com		I	10	-1.07492	-0.72634	-1.06201	-0.72799	-1.13475	7.956632	48.24619	1.72E-05	0.000148	5
114897_at	114897	ENSG0000(	C1QTNF1	C1q and tu	Secreted	NI	22	-0.95141	-0.83112	-1.08222	-0.77173	-1.10347	4.18348	91.39023	1.72E-06	2.92E-05	5
2629_at	2629	ENSG0000(	GBA	glucosidase	Secreted	I	33	-0.91597	-0.94066	-0.99761	-0.85553	-1.09355	6.839477	47.33662	1.84E-05	0.000155	5
10067_at	10067	NA	SCAMP3	secretory carrier meml		I	9	-0.96409	-0.83153	-1.02108	-0.92877	-1.05545	9.310973	55.63654	1.03E-05	0.000101	5
54344_at	54344	ENSG0000(	DPM3	dolichyl-ph	Secreted	I	11	-0.95052	-0.90132	-1.02667	-0.94796	-1.1242	9.510398	86.54831	2.09E-06	3.35E-05	5
57026_at	57026	ENSG0000(	PDXP	pyridoxal (pyridoxine, v		I	11	-1.0223	-0.88992	-1.00491	-0.90881	-1.15965	8.430331	67.69562	5.10E-06	6.13E-05	5
26519_at	26519	ENSG0000(	TIMM10	translocase of inner m		I	10	-0.797	-0.70926	-1.13817	-0.87806	-0.85169	10.34837	92.36817	1.65E-06	2.85E-05	5
79019_at	79019	ENSG0000(	CENPM	centromere protein M		I	11	-0.81942	-0.82615	-1.12927	-1.02711	-0.84473	8.098785	34.22406	5.82E-05	0.00037	5
1030_at	1030	ENSG0000(	CDKN2B	cyclin-depe	kinase	I	33	-0.81935	-0.77864	-1.01031	-0.87064	-1.00803	3.63353	38.05092	4.01E-05	0.000278	5
51647_at	51647	ENSG0000(	FAM96B	family with sequence s		I	18	-0.94561	-0.74396	-1.0365	-0.82546	-0.98602	10.41299	75.63659	3.41E-06	4.67E-05	5
54512_at	54512	NA	EXOSC4	exosome component 4		I	10	-0.74552	-0.73175	-1.1487	-0.8533	-1.05716	9.594469	72.61226	3.96E-06	5.14E-05	5
55848_at	55848	ENSG0000(	C9orf46	chromosome 9 open re		I	10	-0.92493	-0.75432	-1.20681	-0.86985	-0.96909	8.567338	65.1327	5.86E-06	6.79E-05	5
3614_at	3614	ENSG0000(	IMPDH1	IMP (inosine 5'-monop		I	21	-0.8996	-1.0316	-1.16091	-0.90774	-0.76282	7.035666	83.56247	2.38E-06	3.66E-05	5
7168_at	7168	ENSG0000(	TPM1	tropomyosin 1 (alpha)		I	70	-0.98629	-1.01631	-1.27844	-0.89231	-0.81852	5.126152	118.5721	6.64E-07	1.59E-05	5
6573_at	6573	ENSG0000(	SLC19A1	solute carri	transporter	I	33	-1.00882	-1.07806	-1.21408	-0.94535	-0.88267	4.013157	101.0909	1.19E-06	2.30E-05	5
7041_at	7041	ENSG0000(	TGFB1I1	transforming growth fa		I	11	-1.00062	-0.92683	-1.07592	-0.7207	-0.96996	8.752772	66.12076	5.55E-06	6.53E-05	5

79174_at	79174	ENSG0000(	CRELD2	cysteine-ric	Secreted	I	11	-1.09557	-1.04071	-1.14571	-0.77925	-0.99035	9.165974	94.45526	1.52E-06	2.69E-05	5
78999_at	78999	ENSG0000(	LRFN4	leucine rich repeat anc		I	22	-0.94684	-0.90865	-1.09509	-0.89824	-1.00157	5.402175	68.67512	4.84E-06	5.90E-05	5
79006_at	79006	ENSG0000(	METRN	meteorin, g	Secreted	I	20	-0.97661	-0.96131	-1.17844	-0.83489	-0.99733	7.551569	97.2248	1.37E-06	2.51E-05	5
5287_at	5287	ENSG0000(	PIK3C2B	phosphoin	kinase	NI	11	-0.99591	-0.37909	-0.4151	-0.68123	-1.33832	4.693236	53.02608	1.23E-05	0.000115	5
90990_at	90990	ENSG0000(	KIFC2	kinesin family member		NI	11	-1.27542	-0.51443	-0.3976	-0.89016	-1.20007	4.747615	76.78023	3.23E-06	4.50E-05	5
112703_at	112703	ENSG0000(	FAM71E1	family with sequence s		NI	11	-1.15944	-0.40084	-0.90303	-0.92664	-1.00825	4.516715	45.27918	2.16E-05	0.000175	5
63941_at	63941	ENSG0000(	NECAB3	N-terminal	Secreted	NI	11	-0.93522	-0.45123	-1.00222	-0.79465	-0.94545	5.626883	29.8252	9.42E-05	0.000539	5
121268_at	121268	ENSG0000(	RHEBL1	Ras homolog enriched		NI	10	-0.95967	-0.48158	-0.85567	-0.81313	-1.05909	5.098122	15.14477	0.00094	0.003424	5
23231_at	23231	ENSG0000(	SEL1L3	sel-1 suppressor of lin-		NI	31	-1.03559	-0.70361	-0.88453	-0.61196	-0.71789	4.450313	18.24433	0.000507	0.002055	5
5165_at	5165	ENSG0000(	PDK3	pyruvate d	kinase	NI	9	-1.0131	-0.71014	-0.99981	-0.52451	-0.80943	5.734006	21.46385	0.000293	0.001314	5
27044_at	27044	ENSG0000(	SND1	staphylococcal nucleas		I	11	-1.12911	-0.53392	-0.91132	-0.59243	-0.9413	9.072191	51.80036	1.34E-05	0.000122	5
51181_at	51181	ENSG0000(	DCXR	dicarbonyl/L-xylulose r		I	4	-1.15556	-0.65715	-0.94083	-0.59783	-0.99371	8.738439	66.81619	5.35E-06	6.35E-05	5
29844_at	29844	ENSG0000(	TFPT	TCF3 (E2A) fusion part		I	8	-0.95841	-0.7635	-1.10073	-0.6735	-0.91385	8.281058	66.04137	5.58E-06	6.54E-05	5
58477_at	58477	ENSG0000(	SRPRB	signal reco	Secreted	I	30	-1.03498	-0.64354	-1.05787	-0.70157	-0.85676	8.466111	44.5217	2.29E-05	0.000182	5
9709_at	9709	ENSG0000(	HERPUD1	homocysteine-inducibl		I	32	-1.10482	-0.69292	-1.06304	-0.6694	-0.94418	9.801341	42.39419	2.73E-05	0.000208	5
1978_at	1978	ENSG0000(	EIF4EBP1	eukaryotic translation		I	25	-0.83889	-0.52191	-1.10084	-0.64005	-1.02015	10.19043	62.30946	6.88E-06	7.59E-05	5
6746_at	6746	ENSG0000(	SSR2	signal sequence recept		I	22	-0.93536	-0.60909	-1.02777	-0.68565	-0.96478	10.98505	69.61791	4.61E-06	5.70E-05	5
6598_at	6598	ENSG0000(	SMARCB1	SWI/SNF related, matr		NI	23	-0.99631	-0.5758	-1.11814	-0.71103	-0.99147	6.205744	24.2997	0.000191	0.000935	5
1746_at	1746	ENSG0000(	DLX2	distal-less homeobox 2		NI	22	-0.97	-0.55763	-1.09547	-0.77422	-1.06309	3.975853	46.27446	2.00E-05	0.000164	5
25809_at	25809	ENSG0000(	TTLL1	tubulin tyrosine ligase-		NI	10	-0.56733	-0.4817	-0.97292	-0.45146	-1.02102	4.186749	46.51492	1.96E-05	0.000162	5
5531_at	5531	ENSG0000(	PPP4C	protein pho	kinase	I	10	-0.76269	-0.49742	-0.75686	-0.59609	-1.0322	7.935872	31.17889	8.07E-05	0.000478	5
1478_at	1478	ENSG0000(	CSTF2	cleavage stimulation fa		I	11	-0.68114	-0.62919	-0.87335	-0.57664	-1.0761	7.368458	60.29923	7.74E-06	8.20E-05	5
26233_at	26233	ENSG0000(	FBXL6	F-box and leucine-rich		NI	10	-0.64862	-0.3617	-0.59892	-0.61369	-1.01339	5.327346	16.95518	0.000647	0.002513	5
80764_at	80764	ENSG0000(	THAP7	THAP domain containi		NI	22	-0.77234	-0.36439	-0.62401	-0.52611	-1.01351	7.115491	52.8783	1.24E-05	0.000116	5
55611_at	55611	ENSG0000(	OTUB1	OTU domai	peptidase	NI	22	-0.54808	-0.45416	-0.59501	-0.5558	-1.05244	7.3413	47.45348	1.83E-05	0.000154	5
6182_at	6182	ENSG0000(	MRPL12	mitochondrial ribosom		I	13	-0.57353	-0.5363	-0.65988	-0.59308	-1.0601	10.09053	29.41374	9.89E-05	0.00056	5
7301_at	7301	ENSG0000(	TYRO3	TYRO3 prot	kinase	NI	19	-0.59609	-0.38336	-0.86081	-0.7856	-1.03885	4.042893	46.66998	1.94E-05	0.000161	5
6300_at	6300	ENSG0000(	MAPK12	mitogen-ac	kinase	NI	21	-0.69347	-0.42191	-0.86867	-0.82643	-1.28493	5.67834	30.13231	9.09E-05	0.000523	5
65094_at	65094	ENSG0000(	JMJD4	jumonji domain contai		NI	21	-0.54043	-0.66923	-0.66847	-0.92445	-1.21279	5.636311	42.62141	2.68E-05	0.000206	5
1889_at	1889	ENSG0000(	ECE1	endothelin	peptidase	I	53	-0.61445	-0.53469	-0.68258	-0.80431	-1.06999	6.395391	14.20159	0.00116	0.004074	5
10005_at	10005	ENSG0000(	ACOT8	acyl-CoA thioesterase {		I	26	-0.74738	-0.57229	-0.77119	-0.80563	-1.19138	7.905925	46.38163	1.98E-05	0.000163	5
399909_at	399909	ENSG0000(	PCNXL3	pecanex-like 3 (Drosop		NI	11	-0.73665	-0.40722	-0.7171	-0.58133	-1.36749	6.243437	28.85637	0.000106	0.00059	5
55527_at	55527	ENSG0000(	FEM1A	fem-1 homolog a (C. el		I	10	-0.91052	-0.52702	-0.68517	-0.67022	-1.27009	6.553377	50.49439	1.46E-05	0.00013	5
55367_at	55367	ENSG0000(	PIDD	p53-induced death dor		I	11	-0.83774	-0.51536	-0.52994	-0.7695	-1.0937	5.985713	60.40577	7.69E-06	8.16E-05	5
80199_at	80199	ENSG0000(	FUZ	fuzzy homo	Secreted	NI	30	-0.91566	-0.55927	-0.68297	-0.83647	-1.02817	4.790315	36.10876	4.82E-05	0.00032	5
23647_at	23647	ENSG0000(	ARFIP2	ADP-ribosylation facto		I	74	-1.00915	-0.48924	-0.6829	-0.7515	-1.0986	7.082963	36.50353	4.64E-05	0.00031	5
55139_at	55139	ENSG0000(	ANKZF1	ankyrin rep	Secreted	NI	10	-0.95266	-0.59902	-0.72572	-0.33691	-1.10492	6.436938	51.82125	1.33E-05	0.000122	5
1509_at	1509	ENSG0000(	CTSD	cathepsin D	peptidase	I	33	-1.07048	-0.59623	-0.51584	-0.39568	-0.78815	7.623138	22.75312	0.00024	0.00112	5
93129_at	93129	ENSG0000(	ORAI3	ORAI calcium release-a		NI	20	-1.09426	-0.64633	-0.64328	-0.54201	-0.94111	6.355328	18.10196	0.00052	0.002101	5
220_at	220	ENSG0000(	ALDH1A3	aldehyde dehydrogena		I	39	-1.24439	-0.84601	-0.69455	-0.72684	-1.02131	3.918438	31.94986	7.41E-05	0.000446	5
200316_at	200316	ENSG0000(	APOBEC3F	apolipoprotein B mRN.		I	32	-1.20489	-0.8648	-0.53233	-0.91295	-1.13803	4.090392	39.93431	3.38E-05	0.000245	5
26260_at	26260	ENSG0000(	FBXO25	F-box protein 25		I	38	-0.98154	-0.73834	-0.75637	-0.75197	-1.08332	6.534716	47.98935	1.76E-05	0.00015	5
84532_at	84532	ENSG0000(	ACSS1	acyl-CoA synthetase sh		NI	33	-0.92612	-0.6716	-0.80276	-0.91445	-1.14766	4.736846	59.3432	8.20E-06	8.54E-05	5
27286_at	27286	ENSG0000(	SRPX2	sushi-repea	Secreted	I	9	-1.18234	-0.71772	-0.83352	-0.83634	-1.17421	7.097362	44.50269	2.30E-05	0.000182	5
8508_at	8508	ENSG0000(	NIPSNAP1	nipsnap homolog 1 (C.		NI	19	-1.1825	-0.73763	-0.76095	-0.93038	-0.96821	4.919743	25.12148	0.000171	0.000854	5
80700_at	80700	ENSG0000(	UBXN6	UBX domain protein 6		I	20	-1.09223	-0.70576	-0.81133	-0.75637	-0.99694	7.892708	88.96264	1.89E-06	3.11E-05	5
93100_at	93100	ENSG0000(	NAPRT1	nicotinate phosphoribo		I	10	-1.08147	-0.73065	-0.83005	-0.87766	-0.97153	7.266995	97.63701	1.35E-06	2.48E-05	5
84305_at	84305	ENSG0000(	WIBG	within bgcn homolog (		I	10	-0.98309	-1.10378	-0.72699	-0.47951	-1.07904	6.512518	25.85075	0.000155	0.000789	5
85026_at	85026	ENSG0000(	C9orf37	chromosome 9 open re		NI	11	-0.79575	-0.81946	-0.67636	-0.66157	-1.09671	5.996516	24.17995	0.000195	0.000946	5
788_at	788	ENSG0000(	SLC25A20	solute carri	transporter	NI	30	-0.77986	-1.01166	-0.70798	-0.75174	-1.02183	5.183676	44.34672	2.33E-05	0.000184	5
124995_at	124995	ENSG0000(	MRPL10	mitochondrial ribosom		I	10	-1.00055	-1.03342	-0.42998	-0.59089	-0.95325	8.611843	74.23174	3.65E-06	4.88E-05	5
116841_at	116841	ENSG0000(	SNAP47	synaptosomal-associat		I	11	-0.96958	-0.82453	-0.53016	-0.65088	-1.02405	7.597754	42.89322	2.62E-05	0.000202	5
10654_at	10654	ENSG0000(	PMVK	phosphom	kinase	I	33	-1.00511	-0.87356	-0.59696	-0.63205	-0.99908	7.210405	46.19154	2.01E-05	0.000165	5
112849_at	112849	ENSG0000(	C14orf149	chromosome 14 open		NI	20	-0.83799	-1.08235	-0.51545	-0.62854	-1.04233	6.463247	76.44721	3.29E-06	4.55E-05	5
2101_at	2101	ENSG0000(	ESRRA	estrogen-re	nuclear rec	NI	6	-0.81546	-1.02478	-0.53799	-0.7817	-0.98509	4.819384	11.49512	0.002286	0.00718	5
51409_at	51409	ENSG0000(	HEMK1	HemK metl	Secreted	NI	21	-0.89923	-1.10965	-0.47258	-0.74069	-0.92373	5.085149	79.48225	2.85E-06	4.13E-05	5
53838_at	53838	ENSG0000(	C11orf24	chromosor	Secreted	NI	11	-1.40096	-0.51971	-0.57842	-0.58043	-0.88145	5.982236	31.16553	8.08E-05	0.000478	5
10362_at	10362	ENSG0000(	HMG20B	high mobility group 20		I	28	-1.51434	-1.00262	-0.68327	-0.72132	-0.97099	8.196801	91.6304	1.70E-06	2.91E-05	5
10423_at	10423	ENSG0000(	CDIPT	CDP-diacyl	transporter	NI	30	-1.05525	-0.85591	-0.68587	-0.59402	-0.73363	5.42307	69.62159	4.61E-06	5.70E-05	5
6184_at	6184	ENSG0000(	RPN1	ribophorin I		I	40	-1.0095	-0.93211	-0.79052	-0.54894	-0.85209	9.267485	81.53577	2.60E-06	3.87E-05	5
37_at	37	ENSG0000(	ACADVL	acyl-CoA dehydrogena		I	28	-1.00976	-0.95843	-0.74149	-0.71213	-0.88234	8.164201	33.21456	6.46E-05	0.000402	5
2495_at	2495	ENSG0000(	FTH1	ferritin, heavy polypep		I	12	-1.1465	-0.89593	-0.87934	-0.71143	-0.58681	8.997988	17.10687	0.000628	0.002452	5
123355_at	123355	ENSG0000(	LRRC28	leucine rich repeat con		NI	41	-1.12617	-0.86713	-0.96274	-0.69131	-0.60212	5.510435	70.39004	4.43E-06	5.54E-05	5
126282_at	126282	ENSG0000(	TNFAIP8L1	tumor necrosis factor,		I	19	-1.12202	-0.82433	-0.83819	-0.91741	-0.76065	5.596486	55.61739	1.04E-05	0.000101	5
11118_at	11118	ENSG0000(	BTN3A2	butyrophili	Secreted	I	29	-1.20875	-0.85963	-0.85028	-0.75304	-0.833	7.717717	40.7715	3.14E-05	0.000231	5
58492_at	58492	ENSG0000(	ZNF77	zinc finger protein 77		NI	11	-1.28045	-0.75935	-1.05081	-0.52458	-1.0805	4.809858	80.54149	2.72E-06	3.99E-05	5
9294_at	9294	ENSG0000(	S1PR2	sphingosin	G-protein c	NI	22	-1.18014	-0.92295	-0.93219	-0.54263	-0.92859	5.357977	37.37068	4.27E-05	0.000292	5
51614_at	51614	ENSG0000(	ERGIC3	ERGIC and golgi 3		I	28	-1.12299	-0.82553	-0.91921	-0.74533	-1.05276	9.161148	82.31441	2.51E-06	3.78E-05	5
1984_at	1984	ENSG0000(	EIF5A	eukaryotic translation		I	3	-1.07262	-0.89722	-0.99651	-0.69876	-1.07194	9.670613	39.95277	3.37E-05	0.000244	5
84885_at	84885	ENSG0000(	ZDHHC12	zinc finger, DHHC-type		I	11	-1.11757	-0.96276	-0.9552	-0.6666	-1.09965	6.121789	63.05028	6.59E-06	7.36E-05	5
7086_at	7086	ENSG0000(	TKT	transketolase		I	28	-1.34045	-0.7877	-0.967	-0.72326	-0.89249	9.239451	85.20522	2.22E-06	3.50E-05	5
116349_at	116349	ENSG0000(	C5orf55	chromosome 5 open re		NI	11	-1.3554	-0.83424	-0.93854	-0.85485	-0.94005	5.90268	13.38036	0.001407	0.004777	5
63899_at	63899	ENSG0000(	NSUN3	NOP2/Sun domain fan		I	11	-1.16352	-0.78215	-1.02662	-0.86754	-0.83886	7.18217	79.5711	2.84E-06	4.11E-05	5
114804_at	114804	ENSG0000(	RNF157	ring finger protein 157		NI	22	-1.17356	-0.74509	-1.01454	-0.81986	-1.00635	3.888465	77.11366	3.18E-06	4.45E-05	5
51147_at	51147	ENSG0000(	ING4	inhibitor of growth fan		I	22	-1.20784	-0.85296	-1.09606	-0.77166	-0.95682	7.043617	59.7466	8.00E-06	8.39E-05	5
26275_at	26275	ENSG0000(	HIBCH	3-hydroxyisobutyryl-Co		I	3	-0.7812	-1.01384	-0.25668	-0.9124	-0.96426	7.615806	33.75741	6.11E-05	0.000384	5
26015_at	26015	ENSG0000(	RPAP1	RNA polymerase II ass		I	11	-1.01949	-0.79213	-0.39196	-0.90181	-0.98235	6.465193	75.65284	3.41E-06	4.67E-05	5
3574_at	3574	ENSG0000(	IL7	interleukin	Secreted	NI	64	-1.02735	-0.95534	-0.67291	-1.01213	-1.02202	4.104868	51.03649	1.41E-05	0.000127	5
26229_at	26229	ENSG0000(	B3GAT3	beta-1,3-glucuronyltra		I	14	-0.79478	-0.88825	-0.63242	-1.00534	-1.12708	6.040918	69.71415	4.59E-06	5.68E-05	5
26995_at	26995	ENSG0000(	TRUB2	TruB pseudouridine (p		I	11	-0.78158	-0.95474	-0.76407	-0.91573	-1.02729	8.328313	90.37803	1.79E-06	3.01E-05	5
23042_at	23042	ENSG0000(	PDXDC1	pyridoxal-dependent d		I	33	-0.70629	-1.03131	-0.75558	-0.96788	-1.00268	6.248039	49.96068	1.52E-05	0.000135	5
127428_at	127428	ENSG0000(	TCEANC2	transcription elongatic		NI	33	-0.74207	-1.23148	-0.49085	-1.04596	-0.9771	5.082372	69.7353	4.58E-06	5.68E-05	5

4515_at	4515	ENSG0000(	MTCP1	mature T-cell prolifera		NI	11	-0.82599	-1.41023	-0.50157	-0.86171	-0.95773	4.327605	32.85847	6.71E-05	0.000413	5
127943_at	127943	ENSG0000(	FCRLB	Fc receptor-like B		NI	22	-0.39304	-1.33582	-0.67649	-0.97239	-0.8917	4.277445	74.64265	3.58E-06	4.82E-05	5
7107_at	7107	ENSG0000(	GPR137B	G protein-coupled rec(		I	11	-0.69758	-1.51272	-0.73482	-0.98176	-0.98043	6.240788	67.17166	5.25E-06	6.26E-05	5
84524_at	84524	ENSG0000(	ZC3H8	zinc finger CCCH-type (		I	11	-0.64264	-1.35381	-0.8818	-1.01283	-1.05904	6.996109	62.03472	6.99E-06	7.67E-05	5
54503_at	54503	ENSG0000(	ZDHHC13	zinc finger, DHHC-type		I	11	-1.00528	-1.5729	-0.66264	-0.76874	-1.00078	7.061139	46.49189	1.97E-05	0.000162	5
85458_at	85458	ENSG0000(	DIXDC1	DIX domain containing		NI	44	-0.89378	-1.34212	-0.77191	-0.98993	-0.89928	4.926947	100.2009	1.23E-06	2.35E-05	5
55664_at	55664	ENSG0000(	CDC37L1	cell division cycle 37 h(		I	22	-1.0727	-1.44743	-0.726	-0.94506	-0.88032	5.966452	100.9537	1.20E-06	2.30E-05	5
3111_at	3111	NA	HLA-DOA	major histc	Secreted	NI	45	-1.05616	-1.24506	-0.67302	-0.64829	-0.72634	4.169643	15.34038	0.000901	0.003306	5
1138_at	1138	ENSG0000(	CHRNA5	cholinergic ion channe		NI	22	-0.91675	-1.43644	-0.7738	-0.73501	-0.63998	4.956622	71.56786	4.17E-06	5.34E-05	5
5372_at	5372	ENSG0000(	PMM1	phosphomannomutas(		I	10	-0.84978	-1.27334	-0.90386	-0.70205	-0.69185	7.662316	83.9036	2.34E-06	3.63E-05	5
55669_at	55669	ENSG0000(	MFN1	mitofusin 1	GTPase	I	29	-0.98465	-1.24331	-0.77956	-0.80913	-0.63821	8.223583	89.69569	1.84E-06	3.05E-05	5
285203_at	285203	ENSG0000(	C3orf64	chromosome 3 open r(		NI	21	-1.0062	-1.26589	-0.86717	-0.74592	-0.68459	5.854545	88.2429	1.95E-06	3.18E-05	5
50848_at	50848	ENSG0000(	F11R	F11 recept(	Secreted	NI	65	-0.94334	-1.23894	-0.86901	-0.79491	-0.73453	4.654755	67.7132	5.10E-06	6.13E-05	5
8801_at	8801	ENSG0000(	SUCLG2	succinate-CoA ligase, (		I	14	-0.8991	-1.24804	-0.87249	-0.85716	-0.7044	9.128763	99.21577	1.27E-06	2.39E-05	5
79668_at	79668	ENSG0000(	PARP8	poly (ADP-ribose) poly		NI	10	-1.02861	-0.7059	-0.56135	-0.99058	-0.87009	5.739414	32.39027	7.06E-05	0.00043	5
201266_at	201266	ENSG0000(	SLC39A11	solute carri	transporter	NI	30	-1.08411	-0.59	-0.70875	-0.96762	-0.84722	4.650192	32.19986	7.21E-05	0.000437	5
26230_at	26230	ENSG0000(	TIAM2	T-cell lymphoma invas		I	11	-1.21576	-0.91989	-0.58873	-1.05407	-0.6846	5.899623	35.1715	5.29E-05	0.000344	5
51616_at	51616	ENSG0000(	TAF9B	TAF9B RNA polymeras		I	29	-1.01208	-0.95152	-0.62291	-0.92416	-0.634	6.471471	26.02835	0.000151	0.000776	5
5993_at	5993	ENSG0000(	RFX5	regulatory factor X, 5 (		I	33	-1.03298	-0.97528	-0.8089	-0.9039	-0.68768	7.474825	62.50629	6.80E-06	7.52E-05	5
11212_at	11212	ENSG0000(	PROSC	proline synthetase co-(		I	31	-1.09337	-0.99024	-0.68169	-0.90064	-0.7538	7.243398	39.78105	3.42E-05	0.000247	5
151963_at	151963	ENSG0000(	MB21D2	Mab-21 domain contai		NI	22	-0.87678	-0.6993	-0.83585	-1.01086	-0.7556	5.179663	44.50448	2.30E-05	0.000182	5
51560_at	51560	ENSG0000(	RAB6B	RAB6B, me	GTPase	NI	33	-0.89912	-0.59312	-0.94937	-1.05084	-0.70141	4.00024	83.97577	2.34E-06	3.63E-05	5
23236_at	23236	ENSG0000(	PLCB1	phospholipase C, beta		NI	32	-0.97189	-0.79794	-0.94758	-1.02256	-0.85492	4.045722	75.787	3.39E-06	4.65E-05	5
5763_at	5763	ENSG0000(	PTMS	parathymosin		I	11	-1.10029	-0.86112	-1.02946	-0.95574	-0.91039	6.198479	25.41442	0.000164	0.000827	5
26088_at	26088	ENSG0000(	GGA1	golgi-associated, gamn		NI	30	-0.97921	-0.84595	-1.06243	-0.99384	-0.9234	4.92148	90.00046	1.82E-06	3.03E-05	5
9918_at	9918	ENSG0000(	NCAPD2	non-SMC condensin I (		I	18	-0.98685	-0.90677	-1.02478	-0.99321	-0.92039	7.64413	33.41618	6.33E-05	0.000395	5
23394_at	23394	ENSG0000(	ADNP	activity-dependent ne(		I	31	-1.03639	-0.91002	-1.08798	-0.91178	-0.75962	9.891646	96.0066	1.44E-06	2.58E-05	5
594_at	594	ENSG0000(	BCKDHB	branched c	Secreted	I	43	-0.98492	-0.90232	-1.11833	-0.97786	-0.75555	7.493863	81.08008	2.65E-06	3.93E-05	5
9779_at	9779	ENSG0000(	TBC1D5	TBC1 domain family, m		I	22	-0.90896	-0.84932	-1.04993	-0.84299	-0.73822	8.076838	58.80078	8.48E-06	8.75E-05	5
617_at	617	ENSG0000(	BCS1L	BCS1-like (S. cerevisiae		I	17	-1.0097	-0.80876	-1.03731	-0.81378	-0.77449	7.787578	72.25613	4.03E-06	5.21E-05	5
116832_at	116832	ENSG0000(	RPL39L	ribosomal protein L39-		I	11	-1.01333	-0.87336	-1.00248	-0.81674	-0.79354	9.5702	69.51963	4.63E-06	5.72E-05	5
22949_at	22949	ENSG0000(	PTGR1	prostaglandin reducta:		I	11	-1.09899	-0.6761	-0.66616	-0.78676	-0.64132	8.045691	39.55113	3.49E-05	0.000251	5
49854_at	49854	ENSG0000(	ZNF295	zinc finger protein 295		I	33	-1.08258	-0.67168	-0.86201	-0.81815	-0.39878	6.18126	66.31473	5.50E-06	6.47E-05	5
3455_at	3455	ENSG0000(	IFNAR2	interferon (alpha, beta		NI	31	-1.10376	-0.73782	-0.73068	-0.80931	-0.45236	5.415816	53.62663	1.18E-05	0.000111	5
102_at	102	ENSG0000(	ADAM10	ADAM met peptidase		NI	44	-1.1046	-1.00019	-0.7872	-0.65534	-0.44321	6.50253	45.281	2.16E-05	0.000175	5
1968_at	1968	ENSG0000(	EIF2S3	eukaryotic	GTPase	I	20	-1.05935	-1.00557	-0.81997	-0.79891	-0.51427	10.39398	87.00241	2.05E-06	3.31E-05	5
8479_at	8479	ENSG0000(	HIRIP3	HIRA interacting prote		I	10	-1.10941	-0.97088	-0.87416	-0.71991	-0.52613	7.50447	47.50028	1.82E-05	0.000154	5
9949_at	9949	ENSG0000(	AMMECR1	Alport syndrome, men		I	22	-0.95784	-1.14539	-0.81024	-0.77782	-0.51344	7.954603	56.07568	1.01E-05	9.92E-05	5
79075_at	79075	ENSG0000(	DSCC1	defective in sister chro		I	31	-0.9666	-1.12461	-0.81558	-0.8219	-0.44526	7.648527	64.60025	6.04E-06	6.94E-05	5
3857_at	3857	ENSG0000(	KRT9	keratin 9		NI	19	-1.03688	-1.1064	-0.83009	-0.83265	-0.48359	4.09142	45.16769	2.18E-05	0.000176	5
130872_at	130872	ENSG0000(	AHSA2	AHA1, activator of hea		NI	11	-0.86957	-1.05452	-0.68348	-0.51674	-0.45299	4.197863	10.97635	0.002644	0.008121	5
134266_at	134266	ENSG0000(	GRPEL2	GrpE-like 2, mitochonc		I	15	-1.01463	-1.00037	-0.61232	-0.55046	-0.58345	7.22517	60.27045	7.76E-06	8.20E-05	5
80321_at	80321	ENSG0000(	CEP70	centrosomal protein 7(		NI	83	-1.11593	-1.11499	-0.62392	-0.6884	-0.35686	6.33792	60.67377	7.57E-06	8.05E-05	5
112479_at	112479	ENSG0000(	ERI2	ERI1 exoribonuclease f		NI	43	-1.09749	-1.02557	-0.66264	-0.75118	-0.36982	4.856891	73.44986	3.80E-06	5.00E-05	5
79230_at	79230	ENSG0000(	ZNF557	zinc finger protein 557		NI	33	-0.89234	-1.08782	-0.74529	-0.75891	-0.41961	5.040399	66.06332	5.57E-06	6.54E-05	5
7257_at	7257	ENSG0000(	TSNAX	translin-as:	transporter	I	22	-0.9762	-1.0662	-0.66791	-0.75976	-0.51438	8.933789	61.61468	7.16E-06	7.76E-05	5
3329_at	3329	ENSG0000(	HSPD1	heat shock 60kDa prot		I	12	-0.8541	-1.05604	-0.6275	-0.72807	-0.50242	11.26833	72.05015	4.07E-06	5.24E-05	5
9868_at	9868	ENSG0000(	TOMM70A	translocase	Secreted	I	28	-0.89582	-1.1138	-0.61343	-0.72844	-0.51502	8.420014	76.92889	3.21E-06	4.48E-05	5
23178_at	23178	ENSG0000(	PASK	PAS domai	kinase	NI	10	-0.91005	-0.88902	-1.05449	-0.73421	-0.3321	4.941569	79.95997	2.79E-06	4.06E-05	5
9156_at	9156	ENSG0000(	EXO1	exonuclease 1		I	10	-1.12465	-0.98674	-1.07962	-0.7485	-0.31952	7.546365	78.05576	3.05E-06	4.32E-05	5
529_at	529	ENSG0000(	ATP6V1E1	ATPase, H+	transporter	I	10	-1.09396	-0.98334	-1.08533	-0.75944	-0.70928	10.44508	74.70548	3.57E-06	4.81E-05	5
26046_at	26046	ENSG0000(	LTN1	listerin E3 ubiquitin pr(		I	22	-0.94921	-1.07248	-1.0171	-0.91858	-0.69238	7.680019	79.70818	2.82E-06	4.09E-05	5
56945_at	56945	ENSG0000(	MRPS22	mitochond	Secreted	I	17	-1.0345	-0.99927	-0.94677	-0.87375	-0.73058	10.19009	60.25156	7.76E-06	8.20E-05	5
1314_at	1314	ENSG0000(	COPA	coatomer p	Secreted	I	22	-1.07402	-1.0153	-1.01739	-0.89965	-0.69035	8.460738	82.35834	2.51E-06	3.77E-05	5
201562_at	201562	ENSG0000(	PTPLB	protein tyrosine phosp		NI	11	-1.07223	-1.04062	-1.17174	-0.85861	-0.535	4.042008	14.41662	0.001104	0.003912	5
8886_at	8886	ENSG0000(	DDX18	DEAD (Asp-Glu-Ala-As)		I	14	-1.0922	-1.07933	-1.14347	-0.9345	-0.49734	8.96011	96.76677	1.39E-06	2.54E-05	5
79728_at	79728	ENSG0000(	PALB2	partner and localizer o		I	11	-0.97979	-1.09296	-1.13326	-0.96132	-0.56434	6.960501	78.66675	2.96E-06	4.23E-05	5
84172_at	84172	ENSG0000(	POLR1B	polymerase (RNA) I po		I	66	-1.02057	-1.13733	-1.15709	-0.97647	-0.57852	7.169334	108.6724	9.13E-07	1.98E-05	5
54953_at	54953	ENSG0000(	C1orf27	chromosome 1 open r(		I	31	-0.81681	-1.00472	-0.90274	-0.77581	-0.40074	5.765293	71.04032	4.28E-06	5.42E-05	5
5775_at	5775	ENSG0000(	PTPN4	protein tyr(	phosphopr	NI	9	-0.67176	-1.11396	-0.94555	-0.71566	-0.5142	5.939873	25.91145	0.000153	0.000785	5
5019_at	5019	ENSG0000(	OXCT1	3-oxoacid CoA transfer		I	32	-0.797	-1.02097	-0.89428	-0.86376	-0.69067	5.807569	45.65893	2.10E-05	0.00017	5
55759_at	55759	ENSG0000(	WDR12	WD repeat domain 12		I	13	-0.8456	-1.0715	-0.84981	-0.90866	-0.54467	10.49087	90.43422	1.78E-06	3.01E-05	5
83752_at	83752	ENSG0000(	LONP2	lon peptida	peptidase	I	33	-0.76517	-1.11451	-0.85433	-0.95547	-0.63459	6.753273	37.19153	4.34E-05	0.000295	5
57477_at	57477	ENSG0000(	SHROOM4	shroom family membe		NI	31	-0.75456	-1.10181	-0.75003	-0.60263	-0.65755	3.783525	33.08697	6.55E-05	0.000406	5
55353_at	55353	ENSG0000(	LAPTM4B	lysosomal protein tran		I	22	-0.82149	-1.00053	-0.70258	-0.70165	-0.6055	10.12883	64.40785	6.11E-06	6.98E-05	5
22916_at	22916	ENSG0000(	NCBP2	nuclear cap binding pr(		I	55	-0.77761	-1.05912	-0.73636	-0.75428	-0.62537	8.954532	76.77485	3.23E-06	4.50E-05	5
79672_at	79672	ENSG0000(	FN3KRP	fructosami	kinase	I	31	-0.91934	-1.01856	-0.6829	-0.66908	-0.61155	6.870419	48.81276	1.65E-05	0.000144	5
3428_at	3428	ENSG0000(	IFI16	interferon, gamma-ind		I	30	-0.93912	-1.04371	-0.79757	-0.69933	-0.58259	10.16693	49.29302	1.60E-05	0.00014	5
165055_at	165055	ENSG0000(	CCDC138	coiled-coil domain con		NI	9	-0.88081	-1.07086	-0.8402	-0.66778	-0.72052	5.700968	32.28168	7.14E-05	0.000434	5
81556_at	81556	ENSG0000(	C15orf44	chromosome 15 open		I	33	-0.87684	-1.02648	-0.78484	-0.76244	-0.73037	7.354458	66.46082	5.45E-06	6.46E-05	5
7553_at	7553	ENSG0000(	ZNF7	zinc finger protein 7		I	11	-1.32508	-0.93804	-1.17668	-0.78167	-0.53762	8.052322	62.00243	7.00E-06	7.67E-05	5
321_at	321	ENSG0000(	APBA2	amyloid beta (A4) prec		NI	33	-1.22899	-0.90283	-1.27745	-0.78145	-0.87493	4.786074	56.81786	9.59E-06	9.60E-05	5
5899_at	5899	ENSG0000(	RALB	v-ral simian leukemia v		I	22	-1.23299	-1.02786	-1.27472	-0.9306	-0.66969	6.562499	55.03868	1.08E-05	0.000104	5
3797_at	3797	ENSG0000(	KIF3C	kinesin family member		I	32	-1.23422	-0.94947	-1.3465	-0.97947	-0.68212	6.741136	121.18	6.13E-07	1.51E-05	5
26061_at	26061	ENSG0000(	HACL1	2-hydroxyacyl-CoA lya:		I	7	-1.42459	-1.03238	-1.16672	-0.89064	-0.84284	8.393028	108.1469	9.30E-07	1.99E-05	5
5095_at	5095	ENSG0000(	PCCA	propionyl CoA carboxy		I	20	-1.26548	-1.12914	-1.13413	-0.82666	-0.75782	6.44326	70.70704	4.36E-06	5.47E-05	5
10233_at	10233	ENSG0000(	LRRC23	leucine rich repeat con		NI	28	-1.29512	-1.16757	-1.26243	-0.98081	-0.75904	4.68497	67.23875	5.23E-06	6.24E-05	5
56255_at	56255	ENSG0000(	TMX4	thioredoxin-related tra		I	20	-1.01235	-1.248	-1.1335	-0.6217	-0.64726	6.088112	53.83178	1.16E-05	0.00011	5
1515_at	1515	ENSG0000(	CTSL2	cathepsin L	peptidase	NI	29	-1.27944	-1.31195	-1.0471	-0.6094	-0.73228	4.834534	101.9194	1.15E-06	2.26E-05	5
55827_at	55827	ENSG0000(	DCAF6	DDB1 and CUL4 associ		I	45	-1.25889	-1.09427	-0.86568	-0.85618	-0.55533	7.615832	102.1114	1.15E-06	2.25E-05	5
339559_at	339559	ENSG0000(	ZNF642	zinc finger protein 642		I	11	-1.20246	-1.1751	-0.97296	-0.83553	-0.67083	4.553863	99.90787	1.24E-06	2.36E-05	5
4711_at	4711	ENSG0000(	NDUFB5	NADH dehydrogenase		I	22	-1.13916	-1.13997	-0.87666	-0.74091	-0.60399	10.38432	81.4987	2.60E-06	3.87E-05	5

29_at	29	ENSG0000(	ABR	active BCR-related gen		I	39	-1.13267	-1.12717	-0.86769	-0.82982	-0.68004	8.544945	84.79575	2.26E-06	3.54E-05	5
114926_at	114926	ENSG0000(	C8orf40	chromosome 8 open re		I	10	-1.22765	-1.01994	-0.94412	-0.65443	-0.75503	8.30014	67.73457	5.09E-06	6.13E-05	5
79001_at	79001	ENSG0000(	VKORC1	vitamin K e	peptidase	I	6	-1.3377	-0.98924	-1.02164	-0.77879	-0.64714	10.30512	52.74325	1.25E-05	0.000116	5
57102_at	57102	ENSG0000(	C12orf4	chromosome 12 open		I	22	-1.30343	-1.00421	-1.06629	-0.81396	-0.72685	6.416721	67.22296	5.23E-06	6.24E-05	5
25824_at	25824	ENSG0000(	PRDX5	peroxiredoxin 5		I	12	-1.27157	-1.09087	-0.97245	-0.88206	-0.88592	10.71234	106.6796	9.77E-07	2.04E-05	5
1622_at	1622	ENSG0000(	DBI	diazepam binding inhil		I	19	-1.21696	-0.98462	-0.89229	-0.82069	-0.79972	10.69038	103.6895	1.08E-06	2.19E-05	5
9516_at	9516	ENSG0000(	LITAF	lipopolysac	Secreted	I	22	-1.27511	-1.05881	-0.86546	-0.79652	-0.79096	9.330682	85.46065	2.19E-06	3.47E-05	5
7421_at	7421	ENSG0000(	VDR	vitamin D (	nuclear rec	I	44	-0.7501	-1.10672	-1.10774	-1.15601	-0.66234	5.485105	47.19512	1.86E-05	0.000156	5
7918_at	7918	NA	GPANK1	G patch domain and ar		I	22	-0.78564	-1.14697	-1.00054	-1.09459	-0.77168	7.788912	120.7624	6.21E-07	1.52E-05	5
9738_at	9738	ENSG0000(	CCP110	centriolar coiled coil pı		I	33	-0.94161	-1.12701	-0.93653	-1.00025	-0.47715	6.186688	96.2747	1.42E-06	2.57E-05	5
150864_at	150864	ENSG0000(	FAM117B	family with sequence s		NI	21	-0.95517	-1.06642	-0.90219	-0.91067	-0.54665	4.597878	53.31203	1.21E-05	0.000113	5
11051_at	11051	ENSG0000(	NUDT21	nudix (nucleoside diph		I	28	-1.02696	-1.04616	-0.92576	-0.93814	-0.56562	9.176307	92.42126	1.65E-06	2.85E-05	5
116064_at	116064	ENSG0000(	LRRC58	leucine rich repeat con		I	30	-0.98723	-1.19967	-0.88029	-1.09491	-0.6843	9.156715	79.38417	2.87E-06	4.14E-05	5
51602_at	51602	ENSG0000(	NOP58	NOP58 ribonucleoprot		I	19	-0.86472	-1.21484	-0.9036	-1.05377	-0.61709	10.26527	63.91974	6.28E-06	7.12E-05	5
170506_at	170506	ENSG0000(	DHX36	DEAH (Asp-Glu-Ala-His		I	48	-0.84013	-1.1526	-0.99727	-1.10226	-0.59133	8.213958	104.2408	1.06E-06	2.16E-05	5
8930_at	8930	ENSG0000(	MBD4	methyl-CpG binding dc		I	30	-0.91453	-1.38378	-1.12332	-0.99468	-0.81172	7.958725	84.70295	2.26E-06	3.55E-05	5
51377_at	51377	ENSG0000(	UCHL5	ubiquitin c	peptidase	I	86	-0.9585	-1.34967	-0.93718	-1.07957	-0.76977	5.694934	127.6929	5.06E-07	1.33E-05	5
5889_at	5889	ENSG0000(	RAD51C	RAD51 homolog C (S. c		I	33	-1.01128	-1.3641	-1.03232	-1.10279	-0.79326	8.208987	132.9203	4.37E-07	1.21E-05	5
84318_at	84318	ENSG0000(	CCDC77	coiled-coil domain con		I	20	-0.93035	-1.16717	-1.06923	-0.84575	-0.72574	6.832005	87.95101	1.97E-06	3.21E-05	5
64208_at	64208	ENSG0000(	POPDC3	popeye domain contai		I	11	-0.87236	-1.25797	-1.13377	-0.81503	-0.75603	7.868547	108.1197	9.31E-07	1.99E-05	5
80198_at	80198	ENSG0000(	MUS81	MUS81 endonuclease		I	11	-0.85848	-1.18922	-0.9855	-0.87356	-0.93647	8.035333	66.32998	5.49E-06	6.47E-05	5
246243_at	246243	ENSG0000(	RNASEH1	ribonuclease H1		I	9	-0.88647	-1.20462	-1.12496	-0.91902	-0.93901	8.245004	70.0703	4.50E-06	5.61E-05	5
79876_at	79876	ENSG0000(	UBA5	ubiquitin-like modifier		I	40	-0.84331	-1.21232	-0.91368	-0.91942	-0.70158	6.645225	102.5306	1.13E-06	2.23E-05	5
2960_at	2960	ENSG0000(	GTF2E1	general transcription fa		I	21	-0.91759	-1.14899	-0.88048	-0.9654	-0.84531	6.823825	67.15646	5.25E-06	6.26E-05	5
4000_at	4000	ENSG0000(	LMNA	lamin A/C		I	61	-0.9139	-1.13204	-0.94856	-0.95569	-0.83791	9.556478	96.25794	1.42E-06	2.57E-05	5
80254_at	80254	ENSG0000(	CEP63	centrosomal protein 6:		NI	50	-0.95525	-1.09904	-0.85302	-0.78654	-0.65626	4.965882	84.00004	2.33E-06	3.63E-05	5
51068_at	51068	ENSG0000(	NMD3	NMD3 homolog (S. cer		I	25	-1.03499	-1.10004	-0.92238	-0.75356	-0.67021	7.895132	75.93533	3.37E-06	4.63E-05	5
307_at	307	ENSG0000(	ANXA4	annexin A4		I	70	-1.05563	-1.09894	-0.81419	-0.78499	-0.82219	9.620004	84.75268	2.26E-06	3.55E-05	5
5608_at	5608	ENSG0000(	MAP2K6	mitogen-ac	kinase	NI	8	-1.055	-1.13191	-0.84463	-0.92818	-0.7244	5.130574	15.15733	0.000937	0.003416	5
9324_at	9324	ENSG0000(	HMGN3	high mobility group nu		I	11	-0.84165	-1.14442	-0.76479	-0.94161	-0.95772	8.512147	72.86664	3.91E-06	5.10E-05	5
56947_at	56947	ENSG0000(	MFF	mitochondrial fission f		I	45	-0.92718	-1.18074	-0.77798	-0.83353	-0.97917	8.604534	71.32426	4.22E-06	5.38E-05	5
8407_at	8407	ENSG0000(	TAGLN2	transgelin 2		I	17	-1.03865	-1.13457	-0.82991	-0.98271	-0.95903	9.772159	39.06353	3.65E-05	0.00026	5
23484_at	23484	ENSG0000(	LEPROTL1	leptin receptor overlap		I	33	-0.97893	-1.14816	-0.90931	-0.91202	-0.96428	7.712665	93.92751	1.55E-06	2.72E-05	5
9470_at	9470	ENSG0000(	EIF4E2	eukaryotic translation		I	13	-0.97238	-1.20023	-0.88534	-0.95607	-0.90699	9.467637	94.30938	1.53E-06	2.70E-05	5
10519_at	10519	ENSG0000(	CIB1	calcium and integrin bi		I	9	-1.02639	-1.03855	-0.88651	-0.91466	-0.87802	10.43325	61.01882	7.42E-06	7.97E-05	5
130814_at	130814	ENSG0000(	PQLC3	PQ loop repeat contair		I	21	-0.96036	-1.00467	-0.94786	-0.84491	-0.80744	6.968014	81.85216	2.56E-06	3.83E-05	5
84186_at	84186	ENSG0000(	ZCCHC7	zinc finger, CCHC doma		I	21	-1.01806	-0.96928	-0.96124	-0.91761	-0.83144	7.98041	86.05893	2.14E-06	3.40E-05	5
124565_at	124565	ENSG0000(	SLC38A10	solute carrier family 38		I	22	-0.94569	-1.1196	-0.96733	-0.93845	-0.94492	5.786026	48.99431	1.63E-05	0.000142	5
22928_at	22928	ENSG0000(	SEPHS2	selenophos	kinase	I	11	-0.98444	-1.15145	-1.00573	-0.94918	-0.92496	8.752542	64.42899	6.10E-06	6.98E-05	5
23568_at	23568	ENSG0000(	ARL2BP	ADP-ribosylation facto		I	25	-0.97557	-1.07234	-0.98162	-0.81932	-0.94012	9.712153	95.79379	1.45E-06	2.59E-05	5
55661_at	55661	ENSG0000(	DDX27	DEAD (Asp-Glu-Ala-As		I	9	-0.92983	-1.05843	-0.93232	-0.92713	-0.95403	9.636762	100.3143	1.22E-06	2.34E-05	5
23423_at	23423	ENSG0000(	TMED3	transmemt	Secreted	I	44	-0.93677	-1.05625	-0.94087	-0.91943	-1.00399	9.655495	107.0403	9.65E-07	2.03E-05	5
203569_at	203569	ENSG0000(	PAGE2	P antigen family, mem		I	14	-5.36258	-4.82582	-4.58679	-3.28906	-2.50333	7.03622	840.7522	4.81E-10	9.95E-07	4
116441_at	116441	ENSG0000(	TM4SF18	transmembrane 4 L six		I	19	-5.31755	-4.84821	-4.60713	-3.24702	-2.5953	7.226994	544.0442	2.41E-09	1.03E-06	4
6285_at	6285	ENSG0000(	S100B	S100 calcium binding p		I	30	-5.24176	-4.69892	-4.1418	-3.45934	-3.14956	7.122012	63.61722	6.38E-06	7.19E-05	4
3117_at	3117	NA	HLA-DQA1	major histc	Secreted	I	20	-4.87158	-4.87171	-4.54274	-3.36434	-3.18153	7.046732	115.8294	7.24E-07	1.68E-05	4
84466_at	84466	ENSG0000(	MEGF10	multiple EC	Secreted	I	11	-3.99526	-4.35665	-4.007	-3.43008	-2.8035	5.719659	358.2958	1.13E-08	1.68E-06	4
158511_at	158511	ENSG0000(	CSAG1	chondrosarcoma assoc		I	10	-4.51401	-4.54995	-3.76546	-2.97313	-2.58177	7.562638	603.287	1.65E-09	9.95E-07	4
5354_at	5354	ENSG0000(	PLP1	proteolipid protein 1		I	54	-4.43947	-4.22923	-3.9722	-3.18204	-2.62556	6.962186	160.3306	2.20E-07	7.98E-06	4
6347_at	6347	ENSG0000(	CCL2	chemokine	kinase	I	10	-4.82897	-4.45775	-3.9223	-3.02043	-2.80595	9.384196	101.6312	1.17E-06	2.27E-05	4
3119_at	3119	NA	HLA-DQB1	major histocompatibili		I	14	-4.98824	-4.72096	-3.93627	-3.13695	-2.8109	8.45407	165.9255	1.94E-07	7.40E-06	4
4100_at	4100	ENSG0000(	MAGEA1	melanoma antigen fan		I	11	-3.46021	-4.66558	-2.79311	-2.97764	-2.74696	6.295345	356.9118	1.15E-08	1.68E-06	4
4111_at	4111	ENSG0000(	MAGEA12	melanoma antigen fan		I	11	-4.56583	-4.92978	-3.77678	-2.84462	-1.81477	7.685204	1089.867	1.84E-10	9.95E-07	4
3212_at	3212	ENSG0000(	HOXB2	homeobox B2		I	11	-4.89682	-5.08407	-3.32098	-2.74303	-2.02772	7.653748	744.6449	7.54E-10	9.95E-07	4
12_at	12	ENSG0000(	SERPINA3	serpin pept	Secreted	I	21	-4.50926	-4.11924	-4.3702	-2.77419	-1.8558	6.753475	699.4271	9.52E-10	9.95E-07	4
83604_at	83604	ENSG0000(	TMEM47	transmembrane protei		I	22	-4.34235	-3.98795	-3.98822	-3.01802	-2.51348	5.96149	84.42446	2.29E-06	3.58E-05	4
10584_at	10584	ENSG0000(	COLEC10	collectin su	Secreted	I	11	-4.5527	-3.90844	-4.05131	-3.13717	-2.31969	6.735764	272.9058	3.10E-08	2.60E-06	4
23475_at	23475	ENSG0000(	QPRT	quinolinate phosphoril		I	22	-4.28167	-4.31095	-3.94751	-2.81114	-2.27678	9.032904	424.0856	6.07E-09	1.23E-06	4
5445_at	5445	ENSG0000(	PON2	paraoxona:	Secreted	I	31	-3.93863	-4.47105	-3.66417	-2.87486	-2.20584	7.558073	771.7769	6.61E-10	9.95E-07	4
9122_at	9122	ENSG0000(	SLC16A4	solute carri	transporter	I	41	-3.97311	-4.31053	-3.8431	-2.81556	-2.33818	6.53604	531.0189	2.64E-09	1.03E-06	4
6999_at	6999	NA	TDO2	tryptophan 2,3-dioxyg		I	11	-4.4734	-3.97539	-3.64794	-2.68227	-2.3925	8.441573	78.09946	3.04E-06	4.32E-05	4
30848_at	30848	ENSG0000(	CTAG2	cancer/testis antigen 2		I	29	-4.07908	-4.07492	-3.67162	-2.79312	-2.51034	7.265498	526.082	2.73E-09	1.03E-06	4
6678_at	6678	ENSG0000(	SPARC	secreted pr	Secreted	I	83	-4.19764	-4.10968	-3.5322	-2.66013	-2.41039	8.886384	435.7218	5.49E-09	1.23E-06	4
7857_at	7857	ENSG0000(	SCG2	secretograi	Secreted	I	11	-4.07419	-4.29436	-3.55348	-2.63385	-2.39153	6.424474	673.3417	1.10E-09	9.95E-07	4
23532_at	23532	ENSG0000(	PRAME	preferentially expresse		I	11	-4.03309	-4.0192	-3.83762	-2.95326	-2.72739	7.037454	602.868	1.65E-09	9.95E-07	4
4494_at	4494	ENSG0000(	MT1F	metallothionein 1F		I	21	-3.92949	-3.61608	-3.97064	-2.7894	-2.38958	8.642677	327.9783	1.57E-08	1.80E-06	4
10631_at	10631	ENSG0000(	POSTN	periostin, c	Secreted	I	43	-3.96967	-4.01158	-3.8677	-2.86588	-2.27817	7.067247	53.50007	1.19E-05	0.000112	4
51299_at	51299	ENSG0000(	NRN1	neuritin 1		I	17	-4.10398	-3.53514	-3.5585	-2.89239	-2.35589	6.941634	481.3176	3.80E-09	1.07E-06	4
25927_at	25927	ENSG0000(	CNRIP1	cannabinoid receptor i		I	20	-3.93178	-3.80747	-3.45463	-2.68426	-2.28203	6.943155	330.3575	1.53E-08	1.80E-06	4
5738_at	5738	ENSG0000(	PTGFRN	prostaglandin F2 recep		I	22	-3.9505	-3.72056	-3.58244	-2.73081	-2.25981	6.292647	430.0896	5.77E-09	1.23E-06	4
8614_at	8614	ENSG0000(	STC2	stanniocalc	Secreted	I	33	-3.77502	-3.83431	-3.4269	-2.91146	-2.26183	7.366065	259.9612	3.71E-08	2.93E-06	4
1000_at	1000	ENSG0000(	CDH2	cadherin 2, type 1, N-c		I	22	-3.94689	-3.84483	-3.48017	-2.85637	-2.42354	6.31938	658.332	1.19E-09	9.95E-07	4
3123_at	3123	NA	HLA-DRB1	major histocompatibili		I	3	-4.23	-4.01318	-3.04113	-2.19997	-1.87919	7.3529	417.4007	6.44E-09	1.25E-06	4
3217_at	3217	ENSG0000(	HOXB7	homeobox B7		I	11	-4.17661	-4.27285	-3.06201	-2.78418	-1.91806	7.441469	564.0904	2.11E-09	1.03E-06	4
54843_at	54843	ENSG0000(	SYTL2	synaptotag	transporter	I	22	-3.87636	-3.88894	-2.88783	-2.49572	-1.75908	8.84004	979.7389	2.73E-10	9.95E-07	4
54947_at	54947	ENSG0000(	LPCAT2	lysophosphatidylcholir		I	32	-3.88013	-3.79435	-3.08871	-2.7117	-2.05172	7.275921	667.698	1.13E-09	9.95E-07	4
10457_at	10457	ENSG0000(	GPNMB	glycoprotein (transmei		I	43	-3.56087	-4.07705	-3.118	-2.77657	-2.19149	6.589313	94.17152	1.54E-06	2.71E-05	4
970_at	970	ENSG0000(	CD70	CD70 molecule		I	11	-3.96441	-4.03508	-3.13914	-2.45124	-2.42141	7.040814	728.3595	8.19E-10	9.95E-07	4
4256_at	4256	ENSG0000(	MGP	matrix Gla	Secreted	I	18	-3.84585	-3.65004	-3.26361	-2.37469	-2.6834	6.881171	171.7082	1.71E-07	6.94E-06	4
10791_at	10791	ENSG0000(	VAMP5	vesicle-associated mer		I	9	-3.87781	-3.70276	-3.35818	-2.71251	-2.74439	8.045199	344.2198	1.31E-08	1.75E-06	4
135228_at	135228	ENSG0000(	CD109	CD109 mol	Secreted	I	33	-3.75613	-3.7805	-3.36535	-2.44049	-2.20711	6.638294	208.5028	8.36E-08	4.75E-06	4
3936_at	3936	ENSG0000(	LCP1	lymphocyte cytosolic p		I	22	-3.88044	-3.61579	-3.45611	-2.4569	-2.29961	8.763037	423.9413	6.08E-09	1.23E-06	4

7164_at	7164	ENSG00000(	TPD52L1	tumor protein D52-like		I	11	-4.00348	-3.60966	-3.44987	-2.48196	-2.20807	9.501373	510.1638	3.06E-09	1.03E-06	4
716_at	716	ENSG00000(	C1S	complemei	G-protein c	I	11	-3.38814	-3.90118	-3.35498	-2.33531	-2.02365	7.312891	739.2384	7.75E-10	9.95E-07	4
51438_at	51438	ENSG00000(	MAGEC2	melanoma antigen fan		I	11	-3.67833	-3.90853	-3.2188	-2.34091	-1.92013	10.00488	588.8813	1.80E-09	9.95E-07	4
85462_at	85462	ENSG00000(	FHDC1	FH2 domain containing		I	11	-3.20509	-3.41941	-3.28925	-2.16683	-1.85629	5.15736	64.93374	5.93E-06	6.85E-05	4
143662_at	143662	ENSG00000(	MUC15	mucin 15, (	Secreted	I	22	-3.47883	-3.40816	-2.92362	-2.46859	-1.94232	6.692867	244.7475	4.63E-08	3.37E-06	4
6274_at	6274	ENSG00000(	S100A3	S100 calcium binding p		I	11	-3.48895	-3.61788	-3.11519	-2.35893	-2.1677	9.503112	426.4095	5.95E-09	1.23E-06	4
286077_at	286077	NA	FAM83H	family with sequence s		I	22	-3.18686	-3.43333	-3.18732	-2.83323	-2.09317	6.079016	578.3818	1.92E-09	9.95E-07	4
2191_at	2191	ENSG00000(	FAP	fibroblast a	peptidase	I	9	-3.01506	-3.60764	-3.62459	-2.79202	-1.98562	6.413991	482.337	3.77E-09	1.07E-06	4
282969_at	282969	ENSG00000(	C10orf125	chromosome 10 open		I	22	-3.28518	-3.29723	-3.1115	-2.59163	-2.53649	6.404934	603.2981	1.65E-09	9.95E-07	4
54_at	54	ENSG00000(	ACP5	acid phosp	Secreted	I	11	-3.58778	-3.21326	-3.61996	-2.76319	-2.46371	7.989197	422.3849	6.16E-09	1.23E-06	4
5168_at	5168	ENSG00000(	ENPP2	ectonucleo	Secreted	I	42	-3.36284	-3.74998	-3.32054	-2.7346	-2.36202	6.763176	234.7458	5.40E-08	3.74E-06	4
6566_at	6566	ENSG00000(	SLC16A1	solute carri	transporte	I	21	-3.45036	-3.4765	-3.4386	-2.51018	-2.18793	5.564863	573.6902	1.98E-09	9.95E-07	4
2995_at	2995	ENSG00000(	GYPC	glycophorin C (Gerbich		I	11	-3.66683	-3.61749	-3.38947	-2.53244	-2.18352	9.122462	503.1325	3.23E-09	1.03E-06	4
60681_at	60681	NA	FKBP10	FK506 bind	Secreted	I	54	-3.67604	-3.53306	-3.47477	-2.61212	-2.21741	6.343399	481.3616	3.80E-09	1.07E-06	4
79750_at	79750	ENSG00000(	ZNF385D	zinc finger protein 385		I	10	-1.45986	-1.75071	-2.6268	-2.34867	-2.39309	4.591413	46.60519	1.95E-05	0.000161	3
54914_at	54914	ENSG00000(	KIAA1797	KIAA1797	Secreted	I	9	-1.93723	-1.72794	-2.96931	-2.21058	-1.97372	5.423539	108.1436	9.30E-07	1.99E-05	3
718_at	718	ENSG00000(	C3	complemei	Secreted	I	8	-2.14613	-3.07379	-3.24293	-2.54043	-2.80377	5.5246	63.33937	6.49E-06	7.27E-05	3
7226_at	7226	ENSG00000(	TRPM2	transient re	ion channe	I	53	-1.99442	-2.68335	-2.76411	-2.514	-2.50453	5.857943	577.2381	1.94E-09	9.95E-07	3
5003_at	5003	ENSG00000(	SLC22A18A	solute carrier family 22		I	11	-1.9517	-2.81837	-2.56653	-2.52762	-2.90078	6.221629	163.1834	2.06E-07	7.66E-06	3
79187_at	79187	ENSG00000(	FSD1	fibronectin type III and		I	9	-2.08488	-2.5411	-2.31896	-1.937	-2.42801	5.62336	167.6472	1.87E-07	7.25E-06	3
2705_at	2705	ENSG00000(	GJB1	gap junctio	transporte	I	11	-1.86931	-3.05461	-2.03997	-1.96898	-2.51394	4.483405	100.0391	1.24E-06	2.36E-05	3
4507_at	4507	ENSG00000(	MTAP	methylthioadenosine p		I	52	-2.24074	-2.82822	-2.60549	-2.47956	-2.13093	5.515466	226.8724	6.12E-08	3.99E-06	3
6696_at	6696	ENSG00000(	SPP1	secreted pl	Secreted	I	65	-1.82293	-3.24987	-2.34669	-2.69406	-2.2802	6.117446	275.0523	3.01E-08	2.57E-06	3
3772_at	3772	ENSG00000(	KCNJ15	potassium	ion channe	I	22	-1.79762	-2.99985	-2.68201	-2.78383	-2.38385	6.097686	113.6512	7.75E-07	1.77E-05	3
3575_at	3575	ENSG00000(	IL7R	interleukin	Secreted	I	24	-2.73795	-3.0478	-3.45815	-2.63821	-2.09495	6.3444	219.3535	6.94E-08	4.29E-06	3
256227_at	256227	ENSG00000(	STEAP1B	STEAP family member		I	9	-2.74826	-2.98763	-3.33565	-2.90063	-2.67093	6.361822	512.5691	3.01E-09	1.03E-06	3
7412_at	7412	ENSG00000(	VCAM1	vascular cell adhesion		I	22	-2.92485	-3.18049	-4.15325	-2.90998	-2.09163	5.947766	29.53848	9.74E-05	0.000554	3
3221_at	3221	ENSG00000(	HOXC4	homeobox C4		I	11	-2.87728	-3.82227	-3.80792	-2.58731	-2.61719	5.900558	342.0932	1.34E-08	1.75E-06	3
1917_at	1917	ENSG00000(	EEF1A2	eukaryotic	GTPase	I	21	-2.98811	-2.50435	-2.47835	-2.51884	-2.53788	6.078974	288.1536	2.53E-08	2.34E-06	3
10215_at	10215	ENSG00000(	OLIG2	oligodendrocyte lineag		I	11	-3.21031	-2.87614	-2.57531	-2.66102	-2.79605	5.432749	227.6233	6.05E-08	3.97E-06	3
125058_at	125058	ENSG00000(	TBC1D16	TBC1 domain family, m		I	11	-2.84691	-3.1895	-2.30468	-2.94007	-2.54033	5.434133	168.124	1.85E-07	7.18E-06	3
644538_at	644538	ENSG00000(	CXorf69	chromosome X open re		I	22	-3.20556	-3.36634	-2.49012	-2.93342	-2.32822	6.195802	106.0072	1.00E-06	2.08E-05	3
26227_at	26227	ENSG00000(	PHGDH	phosphogly	transporte	I	11	-3.25436	-2.13522	-2.56307	-1.71233	-1.86011	8.59982	280.0541	2.82E-08	2.48E-06	3
26471_at	26471	ENSG00000(	NUPR1	nuclear protein, transc		I	10	-3.38527	-2.29056	-2.74979	-1.81099	-2.25544	9.511766	129.6816	4.79E-07	1.29E-05	3
3823_at	3823	ENSG00000(	KLRC3	killer cell lectin-like rec		I	44	-3.24964	-2.78975	-2.43379	-2.19306	-2.08437	5.214111	397.2669	7.73E-09	1.37E-06	3
1993_at	1993	ENSG00000(	ELAVL2	ELAV (embryonic letha		I	11	-3.2743	-2.45139	-2.55602	-2.58275	-1.74687	4.797854	113.9235	7.69E-07	1.76E-05	3
3216_at	3216	ENSG00000(	HOXB6	homeobox B6		I	11	-3.00807	-2.29815	-2.40827	-2.25408	-1.75527	5.137776	103.7149	1.08E-06	2.19E-05	3
590_at	590	ENSG00000(	BCHE	butyrylcho	Secreted	I	22	-3.13603	-2.70548	-2.34568	-2.18584	-1.59781	4.744528	152.28	2.66E-07	8.79E-06	3
4935_at	4935	ENSG00000(	GPR143	G protein-c	G-protein c	I	10	-3.5741	-2.48754	-3.22022	-2.56701	-2.24125	6.782382	918.4749	3.46E-10	9.95E-07	3
715_at	715	ENSG00000(	C1R	complemei	peptidase	I	33	-3.22219	-3.14032	-2.81588	-2.23512	-2.04708	8.291961	500.923	3.28E-09	1.03E-06	3
26577_at	26577	ENSG00000(	PCOLCE2	procollage	Secreted	I	11	-3.22287	-2.8251	-3.05705	-2.38296	-1.95114	6.626675	346.4603	1.28E-08	1.73E-06	3
1029_at	1029	ENSG00000(	CDKN2A	cyclin-dependent kinas		I	30	-3.22665	-2.89244	-2.78875	-2.21835	-2.12061	6.272492	453.3074	4.75E-09	1.23E-06	3
7984_at	7984	ENSG00000(	ARHGEF5	Rho guanine nucleotid		I	11	-3.21171	-2.82716	-3.01095	-2.24755	-2.21604	5.520131	320.4691	1.71E-08	1.89E-06	3
1278_at	1278	ENSG00000(	COL1A2	collagen, ty	Secreted	I	58	-2.62394	-2.52951	-3.2338	-2.41823	-2.40902	5.421013	50.11832	1.50E-05	0.000134	3
646962_at	646962	ENSG00000(	HRCT1	histidine rich carboxyl		I	11	-2.853	-2.55882	-3.29647	-2.13127	-1.9725	5.827507	138.8872	3.72E-07	1.07E-05	3
2297_at	2297	NA	FOXD1	forkhead box D1		I	11	-2.96004	-2.84753	-3.19721	-2.08733	-2.11592	5.856032	71.06202	4.28E-06	5.42E-05	3
100128108	1E+08	NA	LOC10012	hypothetical LOC10012		I	4	-2.88193	-2.7016	-2.89941	-2.6523	-1.6506	4.871811	172.3992	1.68E-07	6.87E-06	3
115557_at	115557	ENSG00000(	ARHGEF25	Rho guanine nucleotid		I	10	-2.84698	-2.46863	-2.6941	-2.72677	-2.12586	5.002455	181.7066	1.39E-07	6.18E-06	3
91614_at	91614	ENSG00000(	DEPDC7	DEP domain containing		I	20	-3.08528	-2.67443	-2.9875	-2.55498	-2.31308	5.143888	525.5749	2.74E-09	1.03E-06	3
51279_at	51279	ENSG00000(	C1RL	complemei	peptidase	I	11	-3.98199	-3.42141	-2.73212	-2.34194	-1.81799	6.531281	265.9188	3.41E-08	2.79E-06	3
3371_at	3371	ENSG00000(	TNC	tenascin C	Secreted	I	39	-3.23334	-3.35404	-2.57187	-2.11442	-1.73922	9.878307	608.404	1.60E-09	9.95E-07	3
5270_at	5270	ENSG00000(	SERPINE2	serpin pept	Secreted	I	66	-3.52292	-3.12937	-2.71898	-2.09457	-1.78262	10.24514	102.7429	1.12E-06	2.22E-05	3
4314_at	4314	ENSG00000(	MMP3	matrix met	peptidase	I	11	-2.74282	-3.53656	-3.17319	-2.15234	-1.41829	9.254825	108.745	9.11E-07	1.98E-05	3
7364_at	7364	ENSG00000(	UGT2B7	UDP glucuronosyltrans		I	8	-3.02757	-3.74997	-2.3595	-2.67088	-1.77586	5.457345	416.3975	6.50E-09	1.25E-06	3
29887_at	29887	ENSG00000(	SNX10	sorting nexin 10		I	53	-2.61193	-3.14998	-2.34705	-2.45818	-1.80371	5.685712	99.11241	1.28E-06	2.39E-05	3
54898_at	54898	ENSG00000(	ELOVL2	ELOVL fatty acid elonga		I	11	-2.77423	-3.26304	-2.19711	-2.53116	-2.28594	4.553047	180.3659	1.43E-07	6.21E-06	3
55711_at	55711	ENSG00000(	FAR2	fatty acyl CoA reducta		I	10	-2.69857	-3.11619	-2.85762	-2.61021	-2.15333	5.212723	244.3431	4.66E-08	3.38E-06	3
79962_at	79962	ENSG00000(	DNAJC22	DnaJ (Hsp40) homolog		I	11	-2.69238	-3.44209	-2.82502	-2.565	-2.27334	5.45213	505.1577	3.18E-09	1.03E-06	3
683_at	683	ENSG00000(	BST1	bone marr	Secreted	I	11	-3.12306	-3.24467	-2.07986	-1.7648	-1.70232	5.667788	474.3853	4.01E-09	1.09E-06	3
3122_at	3122	NA	HLA-DRA	major histc	Secreted	I	31	-3.01572	-2.78257	-1.99968	-1.73166	-1.19825	11.17401	294.8248	2.33E-08	2.20E-06	3
10265_at	10265	ENSG00000(	IRX5	iroquois homeobox 5		I	44	-2.79197	-2.64504	-1.96226	-2.03197	-1.54939	5.104658	148.1495	2.94E-07	9.35E-06	3
7022_at	7022	ENSG00000(	TFAP2C	transcription factor AP		I	21	-2.96733	-2.85311	-2.00775	-2.06064	-1.5334	6.906434	103.2781	1.10E-06	2.20E-05	3
55076_at	55076	ENSG00000(	TMEM45A	transmembrane protei		I	11	-3.21253	-2.71972	-2.45979	-1.61781	-1.56224	8.399799	368.353	1.02E-08	1.60E-06	3
3161_at	3161	ENSG00000(	HMMR	hyaluronan-mediated		I	43	-2.74089	-2.98597	-2.50552	-1.88952	-1.37905	7.501086	249.4276	4.32E-08	3.23E-06	3
1290_at	1290	ENSG00000(	COL5A2	collagen, ty	Secreted	I	59	-2.69807	-2.6903	-2.6619	-1.90787	-1.55579	5.184875	220.0761	6.85E-08	4.27E-06	3
3134_at	3134	NA	HLA-F	major histocompatibili		I	52	-2.89232	-2.72669	-2.51477	-2.04221	-1.55193	7.177888	320.9446	1.70E-08	1.89E-06	3
79411_at	79411	ENSG00000(	GLB1L	galactosidase, beta 1-li		I	11	-2.77398	-2.73042	-2.82337	-1.84317	-1.91947	5.94515	140.4012	3.58E-07	1.05E-05	3
91107_at	91107	ENSG00000(	TRIM47	tripartite motif contair		I	11	-2.655	-2.594	-2.51735	-1.87204	-1.79964	7.145169	316.8305	1.79E-08	1.93E-06	3
2040_at	2040	ENSG00000(	STOM	stomatin		I	54	-2.57119	-2.47325	-2.58384	-1.97699	-1.83998	6.563393	286.9158	2.58E-08	2.34E-06	3
972_at	972	ENSG00000(	CD74	CD74 molecule, major		I	54	-2.83805	-2.89796	-2.28481	-2.00185	-1.83317	8.546987	158.8377	2.27E-07	8.07E-06	3
116372_at	116372	ENSG00000(	LYPD1	LY6/PLAUR	Secreted	I	21	-2.53491	-2.87587	-2.62627	-2.1262	-1.85926	6.217265	59.24241	8.25E-06	8.58E-05	3
10052_at	10052	ENSG00000(	GJC1	gap junction protein, g		I	33	-2.62039	-2.61282	-2.46698	-2.24647	-1.71576	5.21555	242.4207	4.80E-08	3.44E-06	3
79686_at	79686	NA	LINC00341	long intergenic non-pr		I	11	-2.74662	-2.86844	-2.61455	-2.33713	-1.69839	8.214438	266.2167	3.40E-08	2.79E-06	3
8707_at	8707	ENSG00000(	B3GALT2	UDP-Gal:be	Secreted	I	11	-3.96704	-2.84383	-2.65055	-0.82739	-0.39581	3.859567	208.857	8.31E-08	4.75E-06	2
1299_at	1299	ENSG00000(	COL9A3	collagen, ty	Secreted	I	11	-2.96175	-2.85089	-2.02336	-0.83557	-0.78373	4.269356	153.4481	2.58E-07	8.65E-06	2
25890_at	25890	ENSG00000(	ABI3BP	ABI family,	Secreted	I	20	-2.9995	-2.82126	-2.35995	-1.14416	-0.33805	5.022957	21.26281	0.000302	0.001349	2
2335_at	2335	ENSG00000(	FN1	fibronectin	Secreted	I	67	-2.20526	-1.78612	-1.52962	-0.77591	-0.48133	8.899621	92.47192	1.65E-06	2.84E-05	2
5157_at	5157	ENSG00000(	PDGFRL	platelet-de	G-protein c	I	11	-2.14136	-1.72367	-1.15049	-0.85456	-0.77135	6.490726	51.94388	1.32E-05	0.000121	2
631_at	631	ENSG00000(	BFSP1	beaded filament struct		I	11	-1.92054	-1.77834	-1.36557	-0.92193	-0.74782	5.37219	102.8194	1.12E-06	2.22E-05	2
4600_at	4600	ENSG00000(	MX2	myxovirus	GTPase	I	19	-2.3245	-1.60928	-1.81911	-0.91156	-1.04936	5.41064	45.00116	2.21E-05	0.000177	2
10587_at	10587	ENSG00000(	TXNRD2	thioredoxin reductase		I	39	-2.26689	-1.8524	-1.70907	-1.0576	-1.15129	5.425108	74.5878	3.59E-06	4.83E-05	2
3127_at	3127	ENSG00000(	HLA-DRB5	major histocompatibili		I	8	-2.24847	-1.869	-1.78596	-1.28398	-0.9605	5.049096	34.57232	5.62E-05	0.00036	2

26137_at	26137	ENSG0000(	ZBTB20	zinc finger and BTB do	I	20	-1.90164	-1.93386	-1.95191	-1.6294	-0.53087	6.715315	199.3232	9.87E-08	5.18E-06	2
55790_at	55790	ENSG0000(	CSGALNAC	chondroitin sulfate N-a	I	33	-1.82526	-2.00152	-1.85312	-1.28603	-0.92902	3.955767	190.7975	1.16E-07	5.53E-06	2
10161_at	10161	ENSG0000(	LPAR6	lysophospł G-protein c	I	11	-2.00352	-1.83871	-1.76595	-1.6055	-0.98852	4.231529	90.81154	1.76E-06	2.97E-05	2
4435_at	4435	ENSG0000(	CITED1	Cbp/p300-interacting t	I	11	-2.04532	-1.82801	-2.10184	-1.03398	-0.45176	5.500107	49.39388	1.58E-05	0.000139	2
25805_at	25805	ENSG0000(	BAMBI	BMP and activin memb	I	11	-2.23927	-2.04717	-1.94194	-1.18937	-0.83865	7.968488	61.65388	7.15E-06	7.76E-05	2
1836_at	1836	ENSG0000(	SLC26A2	solute carri transporte	I	44	-2.22537	-2.02457	-1.96185	-1.43462	-0.80602	8.054622	88.65391	1.92E-06	3.13E-05	2
54795_at	54795	ENSG0000(	TRPM4	transient re ion channe	I	9	-2.0723	-0.92389	-2.03759	-1.27486	-1.64268	5.838572	57.46302	9.21E-06	9.30E-05	2
6094_at	6094	ENSG0000(	ROM1	retinal outer segment	I	10	-1.87182	-1.28668	-2.08406	-0.86438	-1.0331	4.624642	39.50227	3.51E-05	0.000252	2
51339_at	51339	ENSG0000(	DACT1	dapper, antagonist of I	I	22	-1.6873	-1.28048	-2.02622	-1.15464	-1.23939	4.783325	62.67483	6.74E-06	7.47E-05	2
126969_at	126969	ENSG0000(	SLC44A3	solute carrier family 44	I	21	-1.8668	-1.24751	-1.93046	-1.43978	-1.43693	4.362345	212.2564	7.83E-08	4.56E-06	2
139628_at	139628	ENSG0000(	FOXR2	forkhead box R2	I	11	-1.96567	-1.4208	-1.80626	-1.57137	-1.30935	4.921968	76.46942	3.28E-06	4.55E-05	2
642852_at	642852	NA	LOC642852	hypothetical LOC64285	I	9	-1.95459	-1.51833	-1.83879	-1.34817	-1.11114	7.417953	49.93977	1.52E-05	0.000135	2
340252_at	340252	ENSG0000(	ZNF680	zinc finger protein 680	I	18	-1.82621	-1.27561	-1.59671	-1.56043	-1.10252	4.284829	136.4397	3.97E-07	1.13E-05	2
23765_at	23765	ENSG0000(	IL17RA	interleukin 17 receptor	I	11	-1.72507	-1.39634	-1.83924	-1.71399	-1.14301	5.460154	139.54	3.66E-07	1.07E-05	2
339230_at	339230	ENSG0000(	CCDC137	coiled-coil domain con	I	11	-1.764	-1.18378	-1.53751	-1.26802	-1.18716	8.035578	108.704	9.12E-07	1.98E-05	2
79798_at	79798	ENSG0000(	ARMC5	armadillo r Secreted	I	33	-1.73302	-1.22296	-1.40093	-1.29778	-1.3129	6.26748	198.437	1.00E-07	5.18E-06	2
25847_at	25847	ENSG0000(	ANAPC13	anaphase promoting c	I	20	-1.48513	-1.49788	-1.51899	-1.23195	-1.18146	10.40936	181.1399	1.40E-07	6.20E-06	2
10079_at	10079	ENSG0000(	ATP9A	ATPase, cla transporte	I	21	-1.3618	-1.52203	-1.47684	-1.29474	-1.28955	4.586538	200.0811	9.73E-08	5.17E-06	2
126321_at	126321	ENSG0000(	C19orf28	chromosome 19 open	I	21	-1.58552	-1.42514	-1.48647	-1.23917	-1.36623	7.812208	60.80257	7.51E-06	8.01E-05	2
4668_at	4668	ENSG0000(	NAGA	N-acetylgalactosamini	I	22	-1.60401	-1.56224	-1.34332	-1.23119	-1.40882	7.110373	171.0593	1.73E-07	6.98E-06	2
57017_at	57017	ENSG0000(	COQ9	coenzyme Q9 homolog	I	11	-1.48566	-1.38589	-1.70728	-1.38205	-1.56454	8.763579	155.0686	2.48E-07	8.42E-06	2
56521_at	56521	ENSG0000(	DNAJC12	DnaJ (Hsp40) homolog	I	21	-1.43013	-1.41609	-1.56254	-1.31071	-1.42348	4.175493	222.3523	6.60E-08	4.12E-06	2
84080_at	84080	ENSG0000(	C16orf48	chromosome 16 open	I	11	-1.41606	-1.49505	-1.57797	-1.17839	-1.4912	6.589886	71.73427	4.14E-06	5.30E-05	2
55734_at	55734	ENSG0000(	ZFP64	zinc finger protein 64 I	I	29	-1.45899	-1.52102	-1.50418	-1.50776	-1.27395	5.883596	199.6288	9.81E-08	5.18E-06	2
359845_at	359845	NA	FAM101B	family with sequence s	I	20	-1.64597	-1.40439	-1.40758	-1.45267	-1.36181	5.072473	164.6206	1.99E-07	7.50E-06	2
8800_at	8800	ENSG0000(	PEX11A	peroxisomal biogenesi	I	11	-1.55711	-1.44049	-1.47644	-1.55592	-1.50183	4.629651	168.6969	1.82E-07	7.13E-06	2
23321_at	23321	ENSG0000(	TRIM2	tripartite motif contair	I	33	-2.63022	-2.5671	-1.57994	-1.49103	-1.18371	7.74569	107.6163	9.47E-07	2.02E-05	2
2634_at	2634	ENSG0000(	GBP2	guanylate I GTPase	I	10	-2.65392	-2.85977	-1.51335	-1.6354	-1.2023	7.006194	217.3147	7.18E-08	4.33E-06	2
133383_at	133383	ENSG0000(	C5orf35	chromosome 5 open re	I	10	-2.53533	-2.25426	-1.24267	-1.38707	-0.69576	5.547428	60.28698	7.75E-06	8.20E-05	2
168374_at	168374	ENSG0000(	ZNF92	zinc finger protein 92	I	11	-2.89375	-2.07989	-1.60463	-1.55579	-0.53764	5.12574	223.7779	6.44E-08	4.10E-06	2
26167_at	26167	ENSG0000(	PCDHB5	protocadherin beta 5	I	11	-2.7333	-2.17033	-1.74363	-1.02537	-1.093	5.514084	91.54646	1.71E-06	2.91E-05	2
2934_at	2934	ENSG0000(	GSN	gelsolin Secreted	I	11	-2.82283	-2.21098	-1.31548	-1.33553	-1.35613	8.894913	249.0164	4.34E-08	3.24E-06	2
60489_at	60489	ENSG0000(	APOBEC3G	apolipoprotein B mRN,	I	10	-2.47344	-1.65209	-0.67725	-1.22325	-1.24456	5.329254	15.89277	0.000802	0.002999	2
25797_at	25797	ENSG0000(	QPCT	glutaminyl- peptidase	I	22	-2.4307	-2.23915	-1.1159	-1.46307	-1.45499	6.025642	33.208	6.47E-05	0.000402	2
79912_at	79912	ENSG0000(	PYROXD1	pyridine nucleotide-dis	I	7	-2.32461	-2.16779	-1.00926	-1.64835	-1.09629	4.977667	347.0403	1.28E-08	1.73E-06	2
8544_at	8544	ENSG0000(	PIR	pirin (iron-binding nuc	I	6	-2.21313	-2.16427	-0.84347	-1.67858	-1.3962	6.206497	198.4009	1.00E-07	5.18E-06	2
56130_at	56130	ENSG0000(	PCDHB6	protocadherin beta 6	I	33	-1.96639	-1.6644	-1.30084	-1.3718	-1.00059	4.262128	158.0341	2.32E-07	8.09E-06	2
83940_at	83940	ENSG0000(	TATDN1	TatD DNase domain co	I	4	-1.99025	-1.86956	-1.42172	-1.47453	-0.87384	8.011442	96.00246	1.44E-06	2.58E-05	2
643911_at	643911	NA	CRNDE	colorectal neoplasia di	I	11	-2.31747	-1.876	-1.11295	-1.29942	-0.77493	9.700557	144.893	3.19E-07	9.88E-06	2
6273_at	6273	ENSG0000(	S100A2	S100 calcium binding p	I	10	-2.29675	-1.82168	-1.1553	-1.37665	-1.22223	8.230224	20.86737	0.000322	0.001423	2
55329_at	55329	ENSG0000(	MNS1	meiosis-specific nuclea	I	11	-1.8985	-1.90557	-1.07092	-1.54809	-0.62979	6.215221	143.6644	3.29E-07	9.97E-06	2
64168_at	64168	ENSG0000(	NECAB1	N-terminal EF-hand cal	I	22	-1.68978	-2.14787	-0.89036	-1.81714	-0.80856	3.990551	38.4942	3.84E-05	0.00027	2
145389_at	145389	ENSG0000(	SLC38A6	solute carrier family 38	I	9	-1.97174	-2.49934	-1.37334	-1.45906	-0.79734	6.622388	194.8725	1.07E-07	5.35E-06	2
9582_at	9582	ENSG0000(	APOBEC3B	apolipoprotein B mRN,	I	19	-2.16799	-2.26306	-1.49642	-1.68638	-0.94711	10.04466	344.0133	1.32E-08	1.75E-06	2
729967_at	729967	ENSG0000(	MORN2	MORN repeat containi	I	10	-1.84131	-1.64223	-1.0544	-1.06487	-1.19791	6.970463	127.0673	5.16E-07	1.35E-05	2
2109_at	2109	ENSG0000(	ETFB	electron-transfer-flavo	I	7	-1.76967	-1.71322	-1.06294	-1.00039	-1.00195	9.003328	175.4083	1.58E-07	6.66E-06	2
10553_at	10553	ENSG0000(	HTATIP2	HIV-1 Tat interactive p	I	33	-1.72674	-1.70683	-1.24484	-1.07641	-1.07875	8.981088	195.6955	1.06E-07	5.33E-06	2
151194_at	151194	ENSG0000(	METTL21A	methyltransferase like	I	9	-1.57631	-1.66035	-1.00891	-1.31501	-1.31421	7.265175	51.91055	1.33E-05	0.000122	2
64081_at	64081	ENSG0000(	PBLD	phenazine biosynthesi	I	20	-1.55911	-1.70379	-1.06543	-1.46831	-1.16409	4.811476	78.547	2.98E-06	4.25E-05	2
51313_at	51313	ENSG0000(	FAM198B	family with Secreted	I	55	-1.60462	-2.0348	-1.14537	-1.29582	-1.05376	3.813073	64.94263	5.93E-06	6.85E-05	2
55502_at	55502	ENSG0000(	HES6	hairy and enhancer of	I	11	-1.50625	-1.71113	-1.23673	-1.35714	-1.02044	7.332206	99.54326	1.26E-06	2.38E-05	2
123920_at	123920	ENSG0000(	CMTM3	CKLF-like MARVEL tran	I	22	-1.38542	-1.68465	-1.21494	-1.15561	-1.09472	7.562734	88.84855	1.90E-06	3.11E-05	2
114793_at	114793	ENSG0000(	FMNL2	formin-like 2	I	22	-1.51143	-1.8537	-1.29373	-1.14659	-1.08433	7.106827	104.6657	1.05E-06	2.13E-05	2
1728_at	1728	ENSG0000(	NQO1	NAD(P)H dehydrogena	I	22	-1.71513	-1.49142	-1.23242	-1.14768	-1.00928	9.981539	199.0588	9.92E-08	5.18E-06	2
64172_at	64172	ENSG0000(	OSGEPL1	O-sialoglyc peptidase	I	11	-1.64495	-1.58195	-1.32964	-1.16045	-0.8937	7.875318	91.77351	1.69E-06	2.89E-05	2
9488_at	9488	ENSG0000(	PIGB	phosphatidylinositol gl	I	9	-1.58913	-1.55369	-1.41485	-1.25334	-0.97665	7.338245	171.1859	1.73E-07	6.98E-06	2
115209_at	115209	ENSG0000(	OMA1	OMA1 hom peptidase	I	11	-1.94092	-1.44182	-1.47568	-1.11995	-0.92338	8.215374	90.9001	1.75E-06	2.96E-05	2
54101_at	54101	ENSG0000(	RIPK4	receptor-in kinase	I	33	-1.8322	-1.45784	-1.41086	-1.34477	-1.15151	5.186712	141.1982	3.50E-07	1.04E-05	2
148022_at	148022	ENSG0000(	TICAM1	toll-like receptor adapt	I	11	-1.79911	-1.56219	-1.52084	-1.24537	-1.09179	6.211608	40.81215	3.13E-05	0.000231	2
23189_at	23189	ENSG0000(	KANK1	KN motif and ankyrin r	I	18	-1.77171	-1.85829	-1.51826	-1.11947	-1.10958	6.94597	86.80244	2.07E-06	3.33E-05	2
2585_at	2585	ENSG0000(	GALK2	galactokina kinase	I	31	-1.78064	-1.79593	-1.34392	-1.23523	-1.17545	7.35387	170.457	1.75E-07	7.02E-06	2
55909_at	55909	ENSG0000(	BIN3	bridging integrator 3	I	22	-1.68268	-1.81592	-1.47719	-1.20656	-1.27403	6.263959	162.5267	2.09E-07	7.73E-06	2
85476_at	85476	ENSG0000(	GFM1	G elongatic GTPase	I	19	-1.76736	-1.69903	-1.34124	-1.26711	-0.88607	8.321067	183.2253	1.35E-07	6.10E-06	2
11341_at	11341	ENSG0000(	SCRG1	stimulator Secreted	I	21	-1.71587	-1.80803	-1.41252	-1.10168	-1.00706	3.898907	83.06981	2.43E-06	3.69E-05	2
79070_at	79070	ENSG0000(	KDELC1	KDEL (Lys-A Secreted	I	7	-1.66569	-1.82985	-1.41259	-1.21847	-0.93543	5.917501	33.7599	6.11E-05	0.000384	2
8460_at	8460	ENSG0000(	TPST1	tyrosylprotein sulfotra	I	11	-1.71044	-1.83556	-1.4564	-1.18617	-0.91253	7.130963	255.7313	3.94E-08	3.05E-06	2
11158_at	11158	ENSG0000(	RABL2B	RAB, meml GTPase	I	3	-2.12948	-1.89624	-1.53209	-1.04002	-1.62932	7.710227	131.2688	4.58E-07	1.25E-05	2
1573_at	1573	ENSG0000(	CYP2J2	cytochrome P450, fam	I	11	-1.77704	-1.65695	-1.56932	-1.0181	-1.44945	5.021313	42.59215	2.69E-05	0.000206	2
55741_at	55741	ENSG0000(	EDEM2	ER degrada Secreted	I	11	-1.84182	-1.42023	-1.54221	-1.17937	-1.66871	8.062831	70.88146	4.32E-06	5.43E-05	2
11078_at	11078	ENSG0000(	TRIOBP	TRIO and F-actin bindi	I	54	-1.89106	-1.52065	-1.43112	-1.40479	-1.2714	7.434844	224.9963	6.32E-08	4.03E-06	2
28232_at	28232	ENSG0000(	SLCO3A1	solute carri transporte	I	44	-1.98713	-1.51191	-1.45158	-1.43725	-1.33551	5.13264	207.7254	8.48E-08	4.78E-06	2
55092_at	55092	ENSG0000(	TMEM51	transmembrane protei	I	11	-1.92445	-1.67394	-1.5847	-1.44329	-1.58251	8.221127	287.8172	2.55E-08	2.34E-06	2
4358_at	4358	ENSG0000(	MPV17	MpV17 mitochondrial	I	11	-1.98139	-1.53496	-1.74588	-1.48017	-1.53233	10.02558	231.0492	5.73E-08	3.86E-06	2
64216_at	64216	ENSG0000(	TFB2M	transcription factor B2	I	9	-1.55315	-1.65144	-1.6154	-1.35086	-1.11453	9.815861	207.9611	8.44E-08	4.78E-06	2
8563_at	8563	ENSG0000(	THOC5	THO complex 5	I	10	-1.60186	-1.6397	-1.73091	-1.30953	-1.2632	7.972177	218.5967	7.02E-08	4.30E-06	2
80349_at	80349	ENSG0000(	WDR61	WD repeat domain 61	I	10	-1.59737	-1.91002	-1.51837	-1.44891	-1.16682	9.906518	188.233	1.22E-07	5.70E-06	2
25777_at	25777	ENSG0000(	SUN2	Sad1 and UNC84 doma	I	54	-1.63164	-1.84461	-1.59141	-1.55233	-1.30868	6.328707	178.1746	1.49E-07	6.42E-06	2
90102_at	90102	ENSG0000(	PHLDB2	pleckstrin homology-li	I	65	-1.58895	-1.71344	-1.39167	-1.55089	-1.19742	6.977719	214.0179	7.59E-08	4.50E-06	2
54478_at	54478	ENSG0000(	FAM64A	family with sequence s	I	17	-1.6376	-1.71382	-1.34443	-1.38351	-1.32647	9.562539	194.7931	1.07E-07	5.35E-06	2
23517_at	23517	ENSG0000(	SKIV2L2	superkiller viralicidic a	I	9	-1.71717	-1.84885	-1.43605	-1.39915	-1.29723	7.623485	102.9921	1.11E-06	2.22E-05	2
79157_at	79157	ENSG0000(	MFSD11	major facilitator super	I	21	-2.01701	-1.96127	-1.683	-1.31182	-1.28902	7.785822	195.3158	1.06E-07	5.35E-06	2
26958_at	26958	NA	COPG2	coatomer protein com	I	10	-2.05516	-1.8576	-1.58593	-1.60691	-1.21378	8.060444	336.6181	1.43E-08	1.76E-06	2

128061_at	128061	ENSG0000(	C1orf131	chromosome 1 open re	I	29	-1.95365	-1.70017	-1.69842	-1.51492	-1.33935	6.809351	111.5113	8.31E-07	1.85E-05	2
129138_at	129138	ENSG0000(	ANKRD54	ankyrin repeat domain	I	11	-1.95892	-1.84418	-1.59181	-1.48841	-1.44842	7.04095	128.3578	4.97E-07	1.32E-05	2
54460_at	54460	ENSG0000(	MRPS21	mitochond Secreted	I	33	-1.76987	-1.74202	-1.62904	-1.43945	-1.30081	10.17811	260.5233	3.68E-08	2.93E-06	2
95681_at	95681	ENSG0000(	TSGA14	testis specific, 14	I	54	-1.85681	-1.68839	-1.71781	-1.39804	-1.11159	5.880431	287.388	2.56E-08	2.34E-06	2
10721_at	10721	ENSG0000(	POLQ	polymerase (DNA direc	I	11	-1.94882	-1.71336	-1.61582	-1.45335	-1.18694	7.375283	121.4147	6.09E-07	1.51E-05	2
25825_at	25825	ENSG0000(	BACE2	beta-site A peptidase	I	33	-1.74622	-1.97552	-1.69926	-1.51916	-1.33242	8.196928	186.2143	1.27E-07	5.87E-06	2
4715_at	4715	ENSG0000(	NDUFB9	NADH dehydrogenase	I	16	-1.83973	-2.02776	-1.79255	-1.57493	-1.19537	11.3004	278.7101	2.87E-08	2.51E-06	2
23336_at	23336	ENSG0000(	SYNM	synemin, intermediate	I	22	-1.8836	-1.97018	-1.81742	-1.62449	-1.1927	7.672045	50.40652	1.47E-05	0.000131	2
10763_at	10763	ENSG0000(	NES	nestin	I	22	-2.67066	-2.39205	-2.27652	-1.13386	-1.50606	5.117419	165.6327	1.95E-07	7.40E-06	2
55287_at	55287	ENSG0000(	TMEM40	transmembrane protei	I	16	-3.16098	-1.62866	-2.29373	-1.40307	-1.64872	4.481565	227.9407	6.02E-08	3.97E-06	2
26157_at	26157	ENSG0000(	GIMAP2	GTPase, IMAP family m	I	11	-2.55828	-1.87257	-2.06711	-1.97889	-1.40301	5.097608	55.14976	1.07E-05	0.000104	2
90768_at	90768	NA	MGC45800	hypothetical LOC90768	I	11	-2.86524	-1.92037	-2.24241	-1.88573	-1.31509	4.446809	149.297	2.86E-07	9.19E-06	2
55039_at	55039	ENSG0000(	TRMT12	tRNA methyltransferas	I	11	-2.64767	-1.92188	-2.562	-1.76903	-1.38422	7.036288	434.0096	5.57E-09	1.23E-06	2
1942_at	1942	ENSG0000(	EFNA1	ephrin-A1 Secreted	I	22	-2.58481	-1.96946	-2.8369	-1.78956	-1.56456	5.152198	174.3929	1.61E-07	6.78E-06	2
57545_at	57545	ENSG0000(	CC2D2A	coiled-coil and C2 dom	I	11	-2.50924	-1.64364	-2.32261	-1.53087	-1.34064	6.5405	294.726	2.33E-08	2.20E-06	2
1593_at	1593	ENSG0000(	CYP27A1	cytochrome P450, fam	I	11	-2.23586	-1.66727	-2.48433	-1.57804	-1.50582	7.67849	166.0112	1.93E-07	7.40E-06	2
1397_at	1397	ENSG0000(	CRIP2	cysteine-rich protein 2	I	28	-2.28027	-1.81651	-2.37505	-1.76167	-1.66995	6.530588	127.7493	5.06E-07	1.33E-05	2
55277_at	55277	ENSG0000(	FGGY	FGGY carbo kinase	I	11	-2.41153	-1.92406	-1.3838	-2.05617	-1.96457	5.664782	98.36611	1.31E-06	2.43E-05	2
6604_at	6604	ENSG0000(	SMARCD3	SWI/SNF related, matr	I	9	-2.62002	-1.64914	-1.94885	-1.92234	-1.87272	6.041631	425.0461	6.02E-09	1.23E-06	2
274_at	274	ENSG0000(	BIN1	bridging integrator 1	I	40	-2.15288	-2.17226	-1.62956	-1.77361	-1.88865	8.582958	263.692	3.52E-08	2.86E-06	2
4054_at	4054	ENSG0000(	LTBP3	latent trans Secreted	I	39	-2.00104	-1.89597	-1.72384	-1.54515	-1.86839	5.404005	194.8035	1.07E-07	5.35E-06	2
6157_at	6157	ENSG0000(	RPL27A	ribosomal protein L27a	I	11	-2.23218	-1.9081	-1.61208	-1.57337	-1.71988	6.380382	61.99209	7.01E-06	7.67E-05	2
3202_at	3202	ENSG0000(	HOXA5	homeobox A5	I	10	-1.89293	-1.69931	-2.13714	-2.13421	-1.65638	4.496136	186.1762	1.27E-07	5.87E-06	2
375061_at	375061	ENSG0000(	FAM89A	family with sequence s	I	9	-1.93107	-2.1046	-2.23144	-1.74326	-1.67082	6.293458	129.252	4.84E-07	1.30E-05	2
10398_at	10398	ENSG0000(	MYL9	myosin, light chain 9, r	I	18	-1.93774	-1.92761	-2.2125	-1.65057	-1.6745	8.52293	357.2673	1.15E-08	1.68E-06	2
3229_at	3229	ENSG0000(	HOXC13	homeobox C13	I	10	-1.96593	-1.93754	-2.10515	-1.80317	-1.74048	4.955446	205.0933	8.88E-08	4.89E-06	2
55194_at	55194	ENSG0000(	FAM176B	family with Secreted	I	11	-1.90576	-1.63008	-2.11264	-1.52943	-1.9568	4.174335	352.7674	1.20E-08	1.71E-06	2
84993_at	84993	ENSG0000(	UBL7	ubiquitin-like 7 (bone r	I	11	-2.07053	-1.869	-2.06332	-1.74523	-1.97887	7.648648	376.5841	9.43E-09	1.56E-06	2
3418_at	3418	ENSG0000(	IDH2	isocitrate dehydrogena	I	25	-1.95886	-1.88079	-2.00651	-1.52691	-1.5966	6.77078	141.7126	3.46E-07	1.03E-05	2
85027_at	85027	ENSG0000(	C5orf62	chromosome 5 open re	I	21	-2.06752	-1.74068	-1.99452	-1.4203	-1.52447	5.653119	216.6142	7.26E-08	4.35E-06	2
9657_at	9657	ENSG0000(	IQCB1	IQ motif containing B1	I	8	-2.17967	-1.88968	-1.98746	-1.34333	-1.56251	5.613144	137.4647	3.87E-07	1.11E-05	2
3226_at	3226	ENSG0000(	HOXC10	homeobox C10	I	10	-2.73755	-1.90884	-2.47371	-2.21602	-2.12271	5.375973	512.5328	3.01E-09	1.03E-06	2
2952_at	2952	ENSG0000(	GSTT1	glutathione S-transfera	I	9	-2.47697	-2.25218	-2.6081	-2.27944	-2.04539	5.607711	151.6276	2.70E-07	8.88E-06	2
140686_at	140686	ENSG0000(	WFDC3	WAP four-c Secreted	I	30	-2.8064	-2.18114	-2.28066	-1.77367	-1.74009	7.661833	412.6931	6.72E-09	1.25E-06	2
54763_at	54763	ENSG0000(	ROPN1	rhophilin associated ta	I	11	-2.4484	-2.03435	-2.45555	-1.79486	-2.05929	6.678377	53.31249	1.21E-05	0.000113	2
254887_at	254887	ENSG0000(	ZDHHC23	zinc finger, DHHC-type	I	11	-2.37066	-1.85095	-2.42395	-2.12269	-1.70584	6.031211	262.9891	3.55E-08	2.88E-06	2
9249_at	9249	ENSG0000(	DHRS3	dehydrogenase/reduct	I	20	-2.43221	-2.1651	-2.40729	-1.78099	-1.68989	4.83289	173.5903	1.64E-07	6.81E-06	2
339803_at	339803	NA	LOC33980:	hypothetical LOC3398(	I	20	-2.12292	-2.124	-2.28198	-1.91524	-1.55647	6.783698	281.9895	2.75E-08	2.43E-06	2
26515_at	26515	ENSG0000(	FXC1	fracture callus 1 homo	I	43	-2.36714	-2.0671	-2.19831	-1.90916	-1.55554	7.50462	327.51	1.58E-08	1.80E-06	2
55351_at	55351	ENSG0000(	STK32B	serine/thre kinase	I	33	-2.51495	-2.20279	-1.92287	-1.87872	-2.20936	4.664887	318.3553	1.75E-08	1.92E-06	2
642946_at	642946	NA	FLVCR1-AS	FLVCR1 antisense RNA	I	20	-2.19518	-2.04034	-1.95299	-1.84651	-1.94438	5.55308	129.9279	4.75E-07	1.28E-05	2
4605_at	4605	ENSG0000(	MYBL2	v-myb myeloblastosis	I	11	-2.3871	-2.03857	-1.91495	-1.95571	-2.06719	7.471442	86.10053	2.13E-06	3.39E-05	2
1123_at	1123	ENSG0000(	CHN1	chimerin (chimaerin) 1	I	27	-2.36936	-2.31074	-1.93112	-1.90123	-1.8202	7.514606	260.8799	3.66E-08	2.93E-06	2
55971_at	55971	ENSG0000(	BAIAP2L1	BAI1-associated protei	I	19	-2.32255	-2.55711	-2.14714	-1.93199	-1.86491	6.244439	207.5159	8.51E-08	4.79E-06	2
254552_at	254552	ENSG0000(	NUDT8	nudix (nucleoside diph	I	9	-2.42588	-2.27977	-2.11541	-1.92302	-1.99002	7.267252	278.3616	2.88E-08	2.51E-06	2
9214_at	9214	ENSG0000(	FAIM3	Fas apoptotic inhibitor	I	50	-2.29152	-2.42468	-2.08213	-2.05966	-2.05269	6.007836	89.70903	1.84E-06	3.05E-05	2
254268_at	254268	ENSG0000(	AKNAD1	AKNA domain containi	I	10	-1.69123	-2.29718	-2.36002	-2.26399	-1.83641	5.718077	442.2406	5.20E-09	1.23E-06	2
7036_at	7036	ENSG0000(	TFR2	transferrin peptidase	I	11	-1.8131	-2.16486	-2.60503	-2.30265	-2.03569	5.33107	158.5766	2.29E-07	8.07E-06	2
1307_at	1307	ENSG0000(	COL16A1	collagen, ty Secreted	I	10	-2.07362	-2.58851	-2.93646	-1.87081	-1.81144	5.758417	321.8132	1.69E-08	1.89E-06	2
10013425S	1E+08	NA	LOC10013	hypothetical LOC1001	I	9	-2.0901	-2.35854	-2.61293	-2.25511	-1.27697	5.105084	310.0565	1.93E-08	1.99E-06	2
6926_at	6926	ENSG0000(	TBX3	T-box 3 Secreted	I	52	-2.11833	-2.12908	-2.76418	-2.32239	-1.69723	5.198834	180.642	1.42E-07	6.21E-06	2
374928_at	374928	ENSG0000(	ZNF773	zinc finger protein 773	I	22	-2.31767	-2.41244	-2.67668	-2.07146	-1.64281	5.376017	152.9882	2.61E-07	8.70E-06	2
5055_at	5055	ENSG0000(	SERPINB2	serpin pept peptidase	I	11	-1.79318	-2.86064	-2.48266	-2.06364	-1.55625	8.005537	31.64554	7.66E-05	0.000458	2
23705_at	23705	ENSG0000(	CADM1	cell adhesion molecule	I	54	-2.25261	-2.73559	-2.18912	-2.09798	-1.63876	5.264045	125.7628	5.35E-07	1.39E-05	2
51027_at	51027	ENSG0000(	BOLA1	bolA homo Secreted	I	11	-2.13847	-2.85201	-2.21368	-2.21088	-1.6575	7.183004	310.4074	1.93E-08	1.99E-06	2
4921_at	4921	ENSG0000(	DDR2	discoidin d kinase	I	9	-2.21492	-2.6695	-2.44201	-2.22123	-1.81457	4.801508	259.8638	3.71E-08	2.93E-06	2
56776_at	56776	ENSG0000(	FMN2	formin 2	I	32	-2.10725	-2.81238	-2.41346	-2.3621	-1.73695	5.183398	300.9012	2.16E-08	2.13E-06	2
6883_at	6883	ENSG0000(	TAF12	TAF12 RNA polymeras	I	10	-2.02922	-2.47176	-2.31208	-1.63113	-1.83031	7.660135	335.8158	1.44E-08	1.76E-06	2
54981_at	54981	ENSG0000(	C9orf95	chromosor kinase	I	9	-2.06272	-2.47212	-2.48357	-1.8211	-1.84709	6.742472	309.7231	1.94E-08	1.99E-06	2
11234_at	11234	ENSG0000(	HPS5	Hermansky-Pudlak syn	I	31	-2.30292	-2.37501	-2.29635	-1.62495	-1.47885	8.423658	154.4622	2.52E-07	8.49E-06	2
58538_at	58538	ENSG0000(	MPP4	membrane protein, pa	I	11	-2.19978	-2.47963	-2.31236	-1.80601	-1.63749	6.061185	206.2356	8.70E-08	4.84E-06	2
59307_at	59307	ENSG0000(	SIGIRR	single immunoglobulir	I	25	-2.23806	-2.50066	-2.06998	-1.61726	-1.78769	6.094023	195.8369	1.05E-07	5.33E-06	2
115908_at	115908	ENSG0000(	CTHRC1	collagen triple helix re	I	33	-2.29972	-2.38472	-2.09971	-1.76839	-1.5465	6.164771	352.8177	1.20E-08	1.71E-06	2
8869_at	8869	ENSG0000(	ST3GAL5	ST3 beta-galactoside a	I	11	-2.18427	-2.47421	-2.01726	-1.89883	-1.59532	8.575893	142.3319	3.40E-07	1.02E-05	2
64167_at	64167	ENSG0000(	ERAP2	endoplasm peptidase	I	30	-2.50887	-2.62805	-2.06277	-2.09855	-1.73234	7.005306	347.064	1.27E-08	1.73E-06	2
5002_at	5002	ENSG0000(	SLC22A18	solute carri transporte	I	10	-2.51186	-2.79354	-2.21626	-2.00657	-1.79223	8.019185	337.4145	1.41E-08	1.76E-06	2
3676_at	3676	ENSG0000(	ITGA4	integrin, alpha 4 (antig	I	38	-2.49667	-2.60999	-2.05835	-1.93452	-1.50032	4.619915	540.5636	2.47E-09	1.03E-06	2
4638_at	4638	ENSG0000(	MYLK	myosin ligh kinase	I	33	-2.52264	-2.57062	-2.38629	-1.81027	-1.47501	8.746849	588.3995	1.81E-09	9.95E-07	2
4828_at	4828	ENSG0000(	NMB	neuromedi Secreted	I	10	-2.3392	-2.73177	-2.37345	-1.88815	-1.61467	7.206308	342.3298	1.34E-08	1.75E-06	2
55619_at	55619	ENSG0000(	DOCK10	dedicator c Secreted	I	33	-2.33449	-2.61738	-2.29558	-2.01627	-1.6531	8.142932	422.7113	6.15E-09	1.23E-06	2
4856_at	4856	ENSG0000(	NOV	nephroblas Secreted	I	22	-2.38779	-2.7461	-2.32084	-2.01587	-1.67052	8.378515	90.71783	1.76E-06	2.98E-05	2
81691_at	81691	ENSG0000(	LOC81691	exonuclease NEF-sp	I	8	-2.25712	-2.58666	-1.3873	-1.93588	-2.06005	5.526946	105.7428	1.01E-06	2.08E-05	2
92737_at	92737	ENSG0000(	DNER	delta/notcl Secreted	I	22	-1.66062	-2.25954	-1.2332	-1.76826	-1.7387	6.82004	68.73443	4.83E-06	5.89E-05	2
84779_at	84779	ENSG0000(	NAA11	N(alpha)-acetyltransfe	I	11	-1.60548	-2.34186	-1.28478	-2.06509	-1.91814	4.775944	110.0315	8.73E-07	1.92E-05	2
6422_at	6422	ENSG0000(	SFRP1	secreted fr Secreted	I	43	-2.19402	-2.08198	-1.51339	-1.3322	-1.07427	4.273884	32.10442	7.28E-05	0.000441	2
3109_at	3109	NA	HLA-DMB	major histocompatibili	I	47	-2.01476	-2.20436	-1.41522	-1.41572	-1.35248	6.319358	83.81667	2.35E-06	3.64E-05	2
2992_at	2992	ENSG0000(	GYG1	glycogenin 1	I	15	-2.24984	-2.19313	-1.61933	-1.55778	-1.2506	8.365285	316.1042	1.80E-08	1.93E-06	2
3321_at	3321	ENSG0000(	IGSF3	immunoglc Secreted	I	33	-2.26235	-2.06846	-1.60424	-1.54627	-1.3395	4.593117	156.1022	2.42E-07	8.30E-06	2
4061_at	4061	ENSG0000(	LY6E	lymphocyte Secreted	I	11	-2.17617	-2.06009	-1.72821	-1.51593	-1.35171	6.709098	154.0267	2.55E-07	8.56E-06	2
64785_at	64785	ENSG0000(	GINS3	GINS complex subunit	I	11	-2.07616	-2.16106	-2.08491	-1.78255	-1.30494	8.83827	429.8466	5.78E-09	1.23E-06	2
3115_at	3115	NA	HLA-DPB1	major histocompatibili	I	60	-2.21176	-2.16587	-2.008	-1.50234	-1.30519	6.254554	64.06528	6.22E-06	7.07E-05	2
11015_at	11015	ENSG0000(	KDELR3	KDEL (Lys-Asp-Glu-Leu	I	22	-2.07228	-2.00005	-1.91953	-1.47617	-1.43981	9.21117	217.5141	7.15E-08	4.33E-06	2

55313_at	55313	ENSG0000(	CPPED1	calcineurin-like phospl		I	53	-2.08268	-2.07413	-1.93952	-1.55222	-1.40606	7.826542	298.5773	2.22E-08	2.14E-06	2
29091_at	29091	ENSG0000(	STXBP6	syntaxin binding prote		I	32	-2.56721	-2.27807	-1.9859	-1.55787	-1.28823	4.128191	60.69887	7.56E-06	8.04E-05	2
26022_at	26022	ENSG0000(	TMEM98	transmembrane protei		I	10	-2.28739	-2.2817	-1.87017	-1.47575	-1.49306	4.696098	55.74144	1.03E-05	0.000101	2
3113_at	3113	NA	HLA-DPA1	major histocompatibili		I	42	-2.44594	-2.27325	-1.80479	-1.38306	-1.23199	10.66033	223.1061	6.51E-08	4.11E-06	2
3108_at	3108	NA	HLA-DMA	major histocompatibili		I	20	-2.50284	-2.26096	-1.71497	-1.43658	-1.40847	8.504502	193.7531	1.10E-07	5.39E-06	2
28990_at	28990	ENSG0000(	ASTE1	asteroid hc	Secreted	I	10	-1.55711	-2.06612	-1.97858	-1.54723	-1.23042	6.003599	199.0304	9.92E-08	5.18E-06	2
6648_at	6648	ENSG0000(	SOD2	superoxide dismutase		I	21	-1.56852	-2.25066	-1.89658	-1.7067	-1.17653	7.956829	12.66645	0.001678	0.005546	2
7456_at	7456	ENSG0000(	WIPF1	WAS/WASL interacting		I	64	-1.57571	-2.16294	-1.51827	-1.68542	-1.39285	5.490596	181.6415	1.39E-07	6.18E-06	2
55686_at	55686	ENSG0000(	MREG	melanoreg	Secreted	I	11	-1.60582	-2.38016	-1.64572	-1.74534	-1.27519	8.053133	299.2642	2.20E-08	2.13E-06	2
10683_at	10683	ENSG0000(	DLL3	delta-like 3	Secreted	I	31	-1.50818	-2.15809	-2.19011	-1.67504	-1.45614	4.734411	169.9142	1.78E-07	7.03E-06	2
200728_at	200728	ENSG0000(	TMEM17	transmembrane protei		I	11	-1.68905	-2.18169	-2.00216	-1.53468	-1.66592	7.310376	166.6739	1.91E-07	7.33E-06	2
6695_at	6695	ENSG0000(	SPOCK1	sparc/oste	Secreted	I	11	-1.8503	-2.07699	-2.04553	-1.57735	-1.48292	9.638488	100.4906	1.22E-06	2.33E-05	2
11057_at	11057	ENSG0000(	ABHD2	abhydrolase domain cc		I	42	-1.80732	-2.05433	-1.97045	-1.70788	-1.50876	6.334354	213.1293	7.71E-08	4.53E-06	2
1306_at	1306	ENSG0000(	COL15A1	collagen, ty	Secreted	I	11	-1.97665	-2.0797	-1.8396	-2.0358	-1.73579	5.166662	32.73943	6.80E-05	0.000417	2
23043_at	23043	ENSG0000(	TNIK	TRAF2 and	kinase	I	30	-1.67946	-2.3263	-1.69544	-2.01977	-1.44906	4.924787	172.9219	1.66E-07	6.84E-06	2
22873_at	22873	ENSG0000(	DZIP1	DAZ interacting protei		I	31	-1.75738	-2.28043	-2.07388	-2.02667	-1.46385	5.482023	151.7388	2.69E-07	8.87E-06	2
26018_at	26018	ENSG0000(	LRIG1	leucine-rich repeats an		I	21	-1.89919	-2.02599	-1.80577	-1.80573	-1.42322	5.932113	116.6873	7.04E-07	1.65E-05	2
55239_at	55239	ENSG0000(	OGFOD1	2-oxoglutarate and iro		I	30	-2.04011	-1.88855	-1.69886	-1.61909	-1.56263	7.920429	214.1795	7.57E-08	4.50E-06	2
51226_at	51226	ENSG0000(	COPZ2	coatomer protein com		I	8	-1.88078	-1.89867	-1.79166	-1.61113	-1.66753	7.222214	254.2376	4.02E-08	3.10E-06	2
56993_at	56993	ENSG0000(	TOMM22	translocase of outer m		I	10	-1.94269	-2.06667	-1.81575	-1.59629	-1.65944	9.818788	334.0109	1.47E-08	1.76E-06	2
51530_at	51530	ENSG0000(	ZC3HC1	zinc finger, C3HC-type		I	10	-1.84969	-1.7858	-1.77424	-1.65869	-1.48308	8.75895	159.511	2.24E-07	8.04E-06	2
286053_at	286053	ENSG0000(	NSMCE2	non-SMC element 2, N		I	9	-1.75545	-1.96829	-1.82819	-1.64024	-1.47302	8.358086	191.5027	1.14E-07	5.51E-06	2
115701_at	115701	ENSG0000(	ALPK2	alpha-kinas	kinase	I	11	-1.69662	-1.76839	-1.84112	-1.59183	-1.49531	4.312206	42.53494	2.70E-05	0.000207	2
51300_at	51300	ENSG0000(	TIMMDC1	translocase	Secreted	I	11	-1.61837	-1.87073	-1.81814	-1.52875	-1.43148	10.85478	229.3885	5.88E-08	3.90E-06	2
81618_at	81618	ENSG0000(	ITM2C	integral membrane prc		I	70	-1.70297	-1.86403	-1.75159	-1.5914	-1.47155	6.527823	218.6268	7.02E-08	4.30E-06	2
84856_at	84856	NA	LOC84856	hypothetical LOC8485(		NI	3	0.423555	-1.818	-1.43861	-1.10435	-0.81999	4.595906	31.94971	7.41E-05	0.000446	1
79630_at	79630	ENSG0000(	C1orf54	chromosome 1 open rc		NI	8	-0.00363	-0.6081	-2.09094	-1.21659	-0.85081	4.741895	74.41808	3.62E-06	4.85E-05	1
55199_at	55199	ENSG0000(	FAM86C1	family with sequence s		NI	3	0.740111	-1.05937	-2.04647	-1.37647	-1.31253	4.21601	18.45059	0.000488	0.001998	1
119467_at	119467	ENSG0000(	CLRN3	clarin 3		I	10	-0.6752	-0.91665	-2.31541	-1.16254	-1.11355	4.027912	13.72258	0.001296	0.004469	1
11272_at	11272	ENSG0000(	PRR4	proline rich	Secreted	I	3	-0.69722	-1.01611	-2.61272	-1.01699	-1.45821	5.539061	16.6466	0.000688	0.002645	1
1281_at	1281	ENSG0000(	COL3A1	collagen, ty	Secreted	I	51	-0.91999	-1.42619	-2.35617	-0.83989	-0.87793	4.046024	33.87964	6.03E-05	0.00038	1
2212_at	2212	ENSG0000(	FCGR2A	Fc fragment of IgG, low		NI	10	-0.94035	-1.08273	-1.67011	-0.7308	-0.73534	4.322299	16.94653	0.000648	0.002516	1
9242_at	9242	ENSG0000(	MSC	musculin		I	11	-0.91427	-0.82683	-2.05251	-0.70085	-1.23744	3.819878	42.43634	2.72E-05	0.000208	1
10800_at	10800	ENSG0000(	CYSLTR1	cysteinyl le	G-protein c	I	11	-0.35175	-1.66173	-1.29356	-2.48088	-2.16415	4.643161	26.65271	0.000139	0.000729	1
623_at	623	ENSG0000(	BDKRB1	bradykinin	G-protein c	I	11	-0.87905	-2.32437	-2.03687	-2.52498	-2.32571	4.991493	104.0423	1.07E-06	2.17E-05	1
8000_at	8000	ENSG0000(	PSCA	prostate stem cell anti		I	11	-0.94848	-1.28039	-1.62122	-1.93728	-2.29156	4.991019	70.5692	4.39E-06	5.50E-05	1
28638_at	28638	NA	TRBC2	T cell receptor beta col		I	33	-1.31976	-1.16846	-1.65992	-2.04951	-2.64218	6.214347	25.73368	0.000157	0.000799	1
8710_at	8710	ENSG0000(	SERPINB7	serpin peptidase inhibi		I	22	-1.25715	-1.76614	-1.39235	-2.01433	-2.06027	5.208338	117.8255	6.80E-07	1.61E-05	1
51702_at	51702	ENSG0000(	PADI3	peptidyl arginine deim		I	11	-1.36492	-1.5749	-1.74482	-2.25003	-2.14091	5.402902	159.7866	2.23E-07	8.02E-06	1
283234_at	283234	ENSG0000(	CCDC88B	coiled-coil domain con		I	11	-1.17349	-1.86638	-1.68381	-2.32234	-2.38906	5.533676	85.04759	2.23E-06	3.51E-05	1
83938_at	83938	ENSG0000(	C10orf11	chromosome 10 open		I	8	-1.28325	-1.53216	-2.08065	-2.04637	-1.70805	5.012867	158.8824	2.27E-07	8.07E-06	1
5396_at	5396	ENSG0000(	PRRX1	paired related homeot		I	21	-1.14088	-1.86639	-1.89044	-1.83194	-1.58707	5.061257	89.41129	1.86E-06	3.07E-05	1
26353_at	26353	ENSG0000(	HSPB8	heat shock	kinase	I	22	-1.14632	-1.95254	-1.94833	-1.97768	-1.54003	5.745053	288.9758	2.51E-08	2.34E-06	1
140885_at	140885	ENSG0000(	SIRPA	signal-regulatory prote		I	20	-1.40164	-1.79234	-1.32955	-1.67199	-2.02259	4.881858	116.4998	7.08E-07	1.66E-05	1
5360_at	5360	ENSG0000(	PLTP	phospholip	Secreted	I	5	-1.54516	-1.99528	-1.46316	-1.49267	-1.96417	5.124959	54.33082	1.13E-05	0.000108	1
6535_at	6535	ENSG0000(	SLC6A8	solute carri	transporter	I	16	-1.13368	-2.25513	-1.66081	-1.84997	-1.78705	7.996693	69.1566	4.72E-06	5.80E-05	1
8612_at	8612	ENSG0000(	PPAP2C	phosphatic	phosphopr	I	21	-1.51168	-2.14811	-1.81824	-1.58657	-1.8118	8.170636	191.8287	1.14E-07	5.51E-06	1
57333_at	57333	ENSG0000(	RCN3	reticulocall	Secreted	I	11	-1.37925	-1.87186	-1.58572	-1.69604	-1.77423	5.467472	72.89544	3.90E-06	5.10E-05	1
6622_at	6622	ENSG0000(	SNCA	synuclein, alpha (non A		I	70	-1.30933	-1.84194	-1.82945	-1.8999	-1.84415	5.305004	120.8541	6.19E-07	1.52E-05	1
11159_at	11159	ENSG0000(	RABL2A	RAB, meml	GTPase	I	3	-1.23693	-1.39798	-2.04261	-1.16171	-2.22445	6.124472	103.2502	1.10E-06	2.20E-05	1
3201_at	3201	ENSG0000(	HOXA4	homeobox A4		I	11	-1.05869	-1.05143	-1.90061	-1.18574	-1.82498	4.575148	131.0054	4.61E-07	1.25E-05	1
84561_at	84561	ENSG0000(	SLC12A8	solute carri	transporter	I	22	-1.04211	-1.27275	-1.76501	-1.45445	-1.8387	5.224151	48.2618	1.72E-05	0.000148	1
100_at	100	ENSG0000(	ADA	adenosine deaminase		I	21	-1.1467	-1.39126	-1.77351	-1.45763	-1.6334	6.329552	39.48732	3.51E-05	0.000252	1
92815_at	92815	ENSG0000(	HIST3H2A	histone cluster 3, H2a		I	11	-1.95893	-1.33393	-1.88682	-1.67273	-2.19466	4.581768	43.75822	2.44E-05	0.000191	1
55715_at	55715	ENSG0000(	DOK4	docking protein 4		I	20	-1.71643	-1.37052	-1.36962	-1.62773	-1.91874	5.903357	82.38486	2.50E-06	3.77E-05	1
8572_at	8572	ENSG0000(	PDLIM4	PDZ and LIM domain 4		I	22	-1.82196	-1.61712	-1.51415	-1.44215	-1.8719	7.451894	252.5104	4.13E-08	3.14E-06	1
3778_at	3778	ENSG0000(	KCNMA1	potassium	ion channe	I	53	-1.59446	-1.34039	-1.76333	-1.55987	-2.0126	4.761733	151.4653	2.71E-07	8.90E-06	1
773_at	773	ENSG0000(	CACNA1A	calcium cha	ion channe	I	33	-1.46593	-1.32732	-1.82029	-1.51562	-1.72882	4.609662	89.37288	1.86E-06	3.07E-05	1
4151_at	4151	ENSG0000(	MB	myoglobin	transporter	I	31	-1.44864	-1.35315	-1.78968	-1.64664	-1.78594	5.986326	19.57901	0.0004	0.001697	1
3383_at	3383	ENSG0000(	ICAM1	intercellular adhesion		I	30	-1.61072	-1.33659	-2.02322	-1.26203	-1.56762	7.70923	111.3574	8.35E-07	1.86E-05	1
64780_at	64780	ENSG0000(	MICAL1	microtubule associatec		I	9	-1.65521	-1.54752	-2.05326	-1.17302	-1.69864	6.394715	72.15979	4.05E-06	5.22E-05	1
661_at	661	ENSG0000(	POLR3D	polymerase (RNA) III (I		I	48	-1.76726	-1.60047	-1.86728	-1.41369	-1.6823	6.118587	190.2983	1.17E-07	5.57E-06	1
6484_at	6484	ENSG0000(	ST3GAL4	ST3 beta-ga	Secreted	I	33	-1.61588	-1.54382	-1.80526	-1.35165	-1.40576	7.181661	212.2058	7.84E-08	4.56E-06	1
221184_at	221184	ENSG0000(	CPNE2	copine II		I	11	-1.6707	-1.60408	-1.84551	-1.26006	-1.45666	6.930006	136.8551	3.93E-07	1.12E-05	1
339229_at	339229	ENSG0000(	C17orf90	chromosome 17 open		I	11	-1.74382	-1.79588	-1.80071	-1.69874	-1.72386	6.192036	67.73149	5.09E-06	6.13E-05	1
80115_at	80115	ENSG0000(	BAIAP2L2	BAI1-associated protei		I	11	-1.67104	-1.78119	-2.01818	-1.68687	-1.73138	5.118356	120.1211	6.33E-07	1.54E-05	1
400916_at	400916	ENSG0000(	CHCHD10	coiled-coil-helix-coiled		I	6	-1.42634	-1.76271	-1.83329	-1.67731	-1.68253	8.823894	274.1204	3.05E-08	2.58E-06	1
118611_at	118611	ENSG0000(	C10orf90	chromosome 10 open		I	19	-1.36936	-1.82853	-1.87844	-1.69131	-1.69331	4.453246	99.66272	1.25E-06	2.37E-05	1
64801_at	64801	ENSG0000(	ARV1	ARV1 homolog (S. cere		I	10	-1.5507	-1.7118	-1.83594	-1.7151	-1.5364	8.020809	197.6779	1.02E-07	5.22E-06	1
27350_at	27350	ENSG0000(	APOBEC3C	apolipoprotein B mRN.		I	10	-1.60404	-1.84143	-1.78752	-1.65987	-1.62138	6.59549	211.5353	7.93E-08	4.60E-06	1
11142_at	11142	ENSG0000(	PKIG	protein kinase (cAMP-(		I	33	-1.59665	-1.82158	-2.05415	-1.48433	-1.56656	8.292985	227.5901	6.05E-08	3.97E-06	1
11030_at	11030	ENSG0000(	RBPMS	RNA binding protein w		I	48	-1.55368	-1.51289	-2.16104	-1.51242	-1.54702	6.20632	216.0709	7.33E-08	4.38E-06	1
253982_at	253982	ENSG0000(	ASPHD1	aspartate beta-hydrox		I	9	-1.64675	-1.66974	-2.24145	-1.61361	-1.55139	7.190625	133.5559	4.30E-07	1.20E-05	1
10758_at	10758	ENSG0000(	TRAF3IP2	TRAF3 interacting prot		I	11	-1.88172	-1.72207	-2.0327	-1.60781	-1.49013	8.179045	218.4068	7.05E-08	4.30E-06	1
7425_at	7425	ENSG0000(	VGF	VGF nerve growth fact		I	22	-1.72767	-1.71244	-2.08958	-1.74514	-1.52309	6.700031	218.1879	7.07E-08	4.30E-06	1
55510_at	55510	ENSG0000(	DDX43	DEAD (Asp-Glu-Ala-Asp		I	18	-1.682	-1.62118	-2.02785	-1.62783	-1.38606	4.104465	201.0222	9.56E-08	5.15E-06	1
27000_at	27000	ENSG0000(	DNAJC2	DnaJ (Hsp40) homolog		I	9	-1.63486	-1.51985	-1.94116	-1.50288	-1.40944	9.581203	241.7306	4.85E-08	3.45E-06	1
11343_at	11343	ENSG0000(	MGLL	monoglyceride lipase		I	60	-1.65136	-1.58238	-1.99327	-1.43257	-1.42767	9.979906	300.7773	2.16E-08	2.13E-06	1
285989_at	285989	ENSG0000(	ZNF789	zinc finger protein 789		I	11	-1.08833	-2.38459	-1.48712	-1.74741	-0.6531	5.881731	105.0527	1.03E-06	2.12E-05	1
55217_at	55217	ENSG0000(	TMLHE	trimethyllysine hydrox		I	9	-1.66916	-1.94423	-1.45788	-1.61861	-1.03401	5.398205	169.9114	1.78E-07	7.03E-06	1
85461_at	85461	ENSG0000(	TANC1	tetratricopeptide repe		I	22	-1.57108	-1.83269	-1.56488	-1.37014	-0.77809	6.10269	171.2309	1.73E-07	6.98E-06	1
23767_at	23767	ENSG0000(	FLRT3	fibronectin leucine rich		I	33	-1.47401	-2.00575	-1.80964	-1.37511	-0.929	4.137194	25.9004	0.000154	0.000786	1

91975_at	91975	ENSG0000(	ZNF300	zinc finger protein 300	I	22	-1.36994	-1.5911	-1.5569	-1.42749	-0.92251	4.862436	152.2473	2.66E-07	8.79E-06	1
3234_at	3234	ENSG0000(	HOXD8	homeobox D8	I	20	-1.36547	-1.73834	-1.53546	-1.6197	-1.10698	4.150139	144.6104	3.21E-07	9.91E-06	1
7328_at	7328	ENSG0000(	UBE2H	ubiquitin-conjugating (	I	36	-1.43095	-1.85572	-1.62761	-1.50496	-1.16176	8.268197	273.8919	3.06E-08	2.58E-06	1
84928_at	84928	ENSG0000(	TMEM209	transmembrane protei	I	21	-1.44998	-1.71941	-1.89603	-1.58006	-0.94205	6.538158	139.5107	3.66E-07	1.07E-05	1
4074_at	4074	ENSG0000(	M6PR	mannose-6 transporter	I	7	-1.34799	-1.74167	-1.79642	-1.78169	-1.00086	8.451653	59.59717	8.08E-06	8.44E-05	1
9897_at	9897	ENSG0000(	KIAA0196	KIAA0196	I	9	-1.4086	-1.83118	-1.71042	-1.76969	-1.0753	8.050716	126.9171	5.18E-07	1.35E-05	1
1130_at	1130	ENSG0000(	LYST	lysosomal trafficking re	I	33	-1.00454	-1.91552	-1.20285	-1.73178	-1.01007	4.61542	87.91978	1.98E-06	3.21E-05	1
3398_at	3398	ENSG0000(	ID2	inhibitor of DNA bindir	I	3	-0.90084	-1.96963	-1.09117	-1.80695	-1.52457	3.837668	48.3432	1.71E-05	0.000148	1
2247_at	2247	ENSG0000(	FGF2	fibroblast growth facto	I	31	-1.64905	-1.98306	-1.00968	-1.66479	-1.48673	4.222476	90.30354	1.79E-06	3.01E-05	1
79837_at	79837	ENSG0000(	PIP4K2C	phosphatic kinase	I	11	-1.82188	-1.73436	-1.21659	-1.15648	-1.49002	7.635709	192.5243	1.12E-07	5.48E-06	1
11092_at	11092	ENSG0000(	C9orf9	chromosome 9 open re	I	12	-1.84633	-1.86095	-0.98141	-1.19799	-1.59301	5.182716	43.43497	2.51E-05	0.000195	1
57580_at	57580	ENSG0000(	PREX1	phosphatidylinositol-3	I	22	-1.3778	-1.56985	-0.75904	-1.63604	-1.6459	4.582032	33.07618	6.56E-05	0.000406	1
27147_at	27147	ENSG0000(	DENND2A	DENN/MADD domain (	I	11	-1.62345	-1.27653	-1.12208	-1.77128	-1.63588	5.990856	67.61397	5.12E-06	6.15E-05	1
254359_at	254359	ENSG0000(	ZDHHC24	zinc finger, DHHC-type	I	11	-1.61497	-1.56462	-1.22126	-1.28537	-1.64123	6.119668	201.1026	9.55E-08	5.15E-06	1
375387_at	375387	ENSG0000(	LRRC33	leucine rich repeat con	I	11	-1.38242	-1.3217	-1.18502	-1.4386	-1.51148	7.007768	80.91053	2.67E-06	3.95E-05	1
6609_at	6609	ENSG0000(	SMPD1	sphingomy Secreted	I	11	-1.52701	-1.44239	-1.18284	-1.37911	-1.47634	8.769687	230.6121	5.77E-08	3.86E-06	1
2258_at	2258	ENSG0000(	FGF13	fibroblast growth facto	I	32	-1.35832	-1.72956	-1.59719	-1.75273	-1.43695	4.39922	182.0212	1.38E-07	6.18E-06	1
55958_at	55958	ENSG0000(	KLHL9	kelch-like 9 (Drosophil	I	44	-1.53037	-1.70173	-1.60865	-1.79552	-1.51378	4.278957	199.4599	9.84E-08	5.18E-06	1
7291_at	7291	ENSG0000(	TWIST1	twist homolog 1 (Dros(	I	11	-1.58859	-1.73578	-1.33513	-1.5412	-1.67633	4.053782	86.68584	2.08E-06	3.33E-05	1
123283_at	123283	ENSG0000(	TARSL2	threonyl-tRNA synthet	I	22	-1.65633	-1.71961	-1.59581	-1.71659	-1.66163	5.649364	185.1957	1.29E-07	5.92E-06	1
4643_at	4643	ENSG0000(	MYO1E	myosin IE	I	19	-1.19603	-1.61124	-1.4651	-1.61006	-1.44949	5.456124	200.6767	9.63E-08	5.16E-06	1
51393_at	51393	ENSG0000(	TRPV2	transient re ion channe	I	20	-1.32064	-1.65793	-1.28881	-1.41241	-1.67917	5.00504	51.16811	1.40E-05	0.000126	1
875_at	875	ENSG0000(	CBS	cystathionine-beta-syr	I	59	-1.1983	-1.7123	-1.28062	-1.55708	-1.62143	4.368135	225.3757	6.28E-08	4.03E-06	1
91607_at	91607	ENSG0000(	SLFN11	schlafen family membe	I	33	-1.44541	-1.63581	-1.25446	-1.79578	-1.38752	4.646935	103.5818	1.09E-06	2.19E-05	1
10544_at	10544	ENSG0000(	PROCR	protein C receptor, enc	I	11	-1.41765	-1.6908	-1.32932	-1.46155	-1.3746	9.207505	176.771	1.54E-07	6.53E-06	1
90557_at	90557	ENSG0000(	CCDC74A	coiled-coil domain con	I	8	-1.51076	-1.61012	-1.33367	-1.44079	-1.39135	6.231692	127.303	5.12E-07	1.34E-05	1
125228_at	125228	ENSG0000(	C18orf19	chromosome 18 open	I	22	-1.22837	-1.67802	-1.16797	-1.30563	-1.29532	8.48304	79.13114	2.90E-06	4.17E-05	1
545_at	545	ENSG0000(	ATR	ataxia telar kinase	I	8	-1.03847	-1.70113	-1.36567	-1.37316	-1.22263	7.076171	189.0912	1.20E-07	5.63E-06	1
8985_at	8985	ENSG0000(	PLOD3	procollage Secreted	I	46	-1.17479	-1.45759	-1.6033	-1.20919	-1.3501	5.552611	157.0594	2.37E-07	8.18E-06	1
79918_at	79918	ENSG0000(	SETD6	SET domain containing	I	46	-1.28743	-1.43151	-1.584	-1.42149	-1.44295	5.921729	105.7914	1.01E-06	2.08E-05	1
1465_at	1465	ENSG0000(	CSRP1	cysteine and glycine-ri	I	63	-1.3154	-1.34869	-1.31753	-1.13926	-1.36306	8.414562	186.8394	1.25E-07	5.83E-06	1
7045_at	7045	ENSG0000(	TGFBI	transformi Secreted	I	44	-1.31954	-1.28866	-1.30093	-1.23095	-1.40517	9.171314	23.18714	0.000225	0.001066	1
7074_at	7074	ENSG0000(	TIAM1	T-cell lymphoma invas	I	22	-1.06911	-1.3379	-1.26328	-1.36479	-1.27005	6.097174	43.05996	2.58E-05	0.0002	1
4882_at	4882	ENSG0000(	NPR2	natriuretic G-protein (	I	10	-1.14951	-1.24346	-1.30684	-1.3438	-1.36969	4.964622	105.9169	1.00E-06	2.08E-05	1
80339_at	80339	ENSG0000(	PNPLA3	patatin-like phospholip	I	11	-1.21724	-1.44767	-1.26065	-1.45053	-1.43556	6.674512	23.21502	0.000224	0.001062	1
5698_at	5698	NA	PSMB9	proteasom peptidase	I	21	-1.18153	-1.41973	-1.35207	-1.33787	-1.32827	8.875526	68.93857	4.78E-06	5.85E-05	1
57019_at	57019	ENSG0000(	CIAPIN1	cytokine induced apop	I	21	-1.27146	-1.47554	-1.35572	-1.32063	-1.4185	8.077858	170.8993	1.74E-07	6.98E-06	1
585_at	585	ENSG0000(	BBS4	Bardet-Biedl syndrome	I	20	-1.03733	-1.4335	-1.12526	-1.5021	-1.34681	5.533689	47.16167	1.87E-05	0.000156	1
64506_at	64506	ENSG0000(	CPEB1	cytoplasmic polyadeny	I	31	-1.13466	-1.32913	-1.14402	-1.46265	-1.43207	4.089017	77.70973	3.10E-06	4.36E-05	1
93183_at	93183	ENSG0000(	PIGM	phosphatidylinositol gl	I	22	-1.34713	-1.3985	-0.84391	-1.43405	-1.28511	5.290202	150.5373	2.77E-07	8.99E-06	1
55604_at	55604	ENSG0000(	LRRC16A	leucine rich repeat con	I	11	-1.18525	-1.48662	-1.01318	-1.35741	-1.13201	5.134238	121.2366	6.12E-07	1.51E-05	1
5627_at	5627	ENSG0000(	PROS1	protein S (a Secreted	I	17	-1.10052	-1.61311	-0.88659	-1.35146	-1.32085	7.050471	80.00606	2.79E-06	4.06E-05	1
80213_at	80213	ENSG0000(	TM2D3	TM2 domain containin	I	29	-1.3448	-1.48967	-1.13789	-1.19355	-1.15841	8.071301	180.525	1.42E-07	6.21E-06	1
6150_at	6150	ENSG0000(	MRPL23	mitochondrial ribosom	I	8	-1.40438	-1.42871	-1.22481	-1.20221	-1.18364	10.67899	171.0536	1.73E-07	6.98E-06	1
78991_at	78991	ENSG0000(	PCYOX1L	prenylcyste Secreted	I	11	-1.46111	-1.5125	-1.25845	-1.29841	-1.24119	7.060724	41.0337	3.07E-05	0.000228	1
27304_at	27304	ENSG0000(	MOCS3	molybdenum cofactor	I	20	-1.368	-1.59794	-1.19693	-1.31317	-1.17086	6.635838	174.9986	1.59E-07	6.71E-06	1
79675_at	79675	ENSG0000(	FASTKD1	FAST kinas kinase	I	6	-1.34378	-1.57643	-1.28911	-1.26232	-1.18363	8.260653	133.7564	4.27E-07	1.19E-05	1
221336_at	221336	ENSG0000(	BEND6	BEN domain containin	I	21	-1.15476	-1.36704	-1.16429	-1.23023	-1.01803	4.122513	44.82847	2.24E-05	0.000179	1
83666_at	83666	ENSG0000(	PARP9	poly (ADP-r Secreted	I	32	-1.2129	-1.47355	-1.28972	-1.22411	-1.06776	5.466641	22.21068	0.00026	0.001196	1
4720_at	4720	ENSG0000(	NDUFS2	NADH dehydrogenase	I	32	-1.23293	-1.33013	-1.30045	-1.21376	-1.04682	9.605544	154.6046	2.51E-07	8.48E-06	1
79047_at	79047	ENSG0000(	KCTD15	potassium ion channe	I	53	-1.2034	-1.37261	-1.2885	-1.29848	-1.0929	4.291645	126.5225	5.24E-07	1.36E-05	1
8459_at	8459	ENSG0000(	TPST2	tyrosylprot Secreted	I	10	-1.39741	-1.35671	-1.10497	-1.24252	-0.96094	8.19424	75.91537	3.37E-06	4.63E-05	1
91433_at	91433	ENSG0000(	RCCD1	RCC1 domain containi	I	22	-1.41939	-1.28371	-1.21132	-1.2755	-0.98979	7.343495	118.8706	6.58E-07	1.58E-05	1
151636_at	151636	ENSG0000(	DTX3L	deltex 3-like (Drosophi	I	33	-1.35637	-1.47274	-1.14101	-1.21269	-0.97476	6.496047	44.29793	2.34E-05	0.000184	1
7277_at	7277	ENSG0000(	TUBA4A	tubulin, alp GTPase	I	20	-1.41636	-1.48633	-1.22085	-1.25879	-1.02355	9.464049	61.43725	7.24E-06	7.83E-05	1
84083_at	84083	ENSG0000(	ZRANB3	zinc finger, RAN-bindir	I	11	-1.94394	-1.89271	-0.48608	-1.16999	-0.84103	4.310652	66.05048	5.58E-06	6.54E-05	1
6814_at	6814	ENSG0000(	STXBP3	syntaxin binding prote	I	11	-1.5565	-1.7773	-0.57844	-1.10316	-0.38069	8.565589	72.99601	3.88E-06	5.08E-05	1
54465_at	54465	ENSG0000(	ETAA1	Ewing tumor-associate	I	11	-1.43758	-1.97114	-0.49783	-1.0183	-0.61798	6.537808	36.01322	4.86E-05	0.000322	1
10610_at	10610	ENSG0000(	ST6GALNA(	ST6 (alpha-N-acetyl-ne	I	11	-1.81656	-2.07843	-1.10678	-0.74861	-0.89633	3.772642	201.2094	9.53E-08	5.15E-06	1
124936_at	124936	ENSG0000(	CYB5D2	cytochrom Secreted	I	11	-1.4627	-1.7137	-0.8857	-0.57677	-0.98779	6.671772	111.7348	8.25E-07	1.84E-05	1
55788_at	55788	ENSG0000(	LMBRD1	LMBR1 domain contair	I	11	-1.26849	-1.64841	-0.84903	-0.8109	-0.91602	8.342601	122.2792	5.93E-07	1.49E-05	1
3912_at	3912	ENSG0000(	LAMB1	laminin, be Secreted	I	19	-1.34876	-1.56095	-0.89129	-0.92075	-0.86779	5.140095	100.4446	1.22E-06	2.34E-05	1
114907_at	114907	ENSG0000(	FBXO32	F-box protein 32	I	8	-2.09104	-1.43816	-0.64669	-1.12962	-0.78417	8.666348	72.7835	3.92E-06	5.12E-05	1
6563_at	6563	ENSG0000(	SLC14A1	solute carri transporter	I	29	-1.75412	-1.12758	-0.71327	-1.06936	-0.94515	4.736298	135.2857	4.10E-07	1.15E-05	1
285193_at	285193	ENSG0000(	DUSP28	dual specif phosphopr	I	11	-1.70888	-1.21926	-0.88841	-1.06611	-0.80376	6.844409	80.57924	2.71E-06	3.99E-05	1
10308_at	10308	ENSG0000(	ZNF267	zinc finger protein 267	I	31	-1.88031	-1.30583	-1.28665	-1.10071	-0.51853	6.147452	95.81271	1.45E-06	2.59E-05	1
84189_at	84189	ENSG0000(	SLITRK6	SLIT and NTRK-like fam	I	22	-2.0616	-1.4903	-1.13134	-1.0579	-0.60819	3.831271	51.99999	1.32E-05	0.000121	1
563_at	563	ENSG0000(	AZGP1	alpha-2-gly Secreted	NI	7	-1.5683	-1.65824	-1.18041	-1.17143	-0.42614	4.983121	13.00759	0.001541	0.005166	1
25937_at	25937	ENSG0000(	WWTR1	WW domain containin	I	29	-1.67808	-1.52655	-1.03975	-0.7398	-0.46803	9.166341	90.2654	1.80E-06	3.01E-05	1
1910_at	1910	ENSG0000(	EDNRB	endothelin G-protein (	I	39	-1.75798	-1.53861	-1.11452	-0.97103	-0.46752	5.47059	167.1099	1.89E-07	7.28E-06	1
29969_at	29969	ENSG0000(	MDFIC	MyoD fami Secreted	I	22	-1.62408	-1.55216	-1.15848	-1.44222	-0.73786	6.659028	104.6005	1.05E-06	2.14E-05	1
583_at	583	ENSG0000(	BBS2	Bardet-Biedl syndrome	I	11	-1.88267	-1.49692	-1.18246	-1.27048	-0.8157	7.474823	197.2601	1.03E-07	5.24E-06	1
23433_at	23433	ENSG0000(	RHOQ	ras homolc GTPase	I	16	-1.60915	-1.49073	-1.37533	-1.05392	-0.65932	6.98005	103.5677	1.09E-06	2.19E-05	1
51454_at	51454	ENSG0000(	GULP1	GULP, engulfment ada	I	44	-1.71502	-1.64781	-1.49576	-1.00508	-0.72952	8.215621	184.1328	1.32E-07	6.03E-06	1
64770_at	64770	ENSG0000(	CCDC14	coiled-coil domain con	I	33	-1.5959	-1.81582	-1.20561	-1.05809	-0.60819	7.251755	136.7469	3.94E-07	1.12E-05	1
55603_at	55603	ENSG0000(	FAM46A	family with sequence s	I	53	-1.58732	-1.86555	-1.2599	-1.10511	-0.8033	6.117916	158.398	2.30E-07	8.08E-06	1
100505813	1.01E+08	NA	LOC10050	hypothetical LOC1005(	NI	9	-1.53585	-0.91111	-1.21817	-1.39597	-0.63908	3.779653	96.73231	1.40E-06	2.54E-05	1
7391_at	7391	ENSG0000(	USF1	upstream transcriptior	NI	11	-0.73298	-1.15384	-1.22185	-1.37718	-0.52362	4.784495	60.78418	7.52E-06	8.02E-05	1
5587_at	5587	ENSG0000(	PRKD1	protein kin kinase	I	11	-1.11255	-1.0598	-1.15568	-1.379	-0.53258	3.820634	25.41432	0.000164	0.000827	1
89970_at	89970	ENSG0000(	RSPRY1	ring finger Secreted	I	9	-0.96591	-1.32093	-1.33125	-1.15149	-0.53024	8.419116	64.79157	5.98E-06	6.87E-05	1
79183_at	79183	ENSG0000(	TTPAL	tocopherol transporter	I	22	-0.86809	-1.26405	-1.42017	-1.2411	-0.73247	8.01185	153.3258	2.59E-07	8.65E-06	1
79159_at	79159	ENSG0000(	NOL12	nucleolar protein 12	I	32	-0.92244	-1.15429	-1.35892	-1.04255	-0.78144	6.150511	75.54241	3.43E-06	4.69E-05	1

85377_at	85377	ENSG0000(	MICALL1	MICAL-like 1	I	11	-0.96979	-1.26379	-1.33828	-1.0887	-0.69103	7.627742	83.9544	2.34E-06	3.63E-05	1
55691_at	55691	ENSG0000(	FRMD4A	FERM domain containi	I	30	-0.79027	-1.36841	-0.91024	-1.31938	-0.90757	6.58901	95.24205	1.48E-06	2.63E-05	1
55634_at	55634	ENSG0000(	ZNF673	zinc finger family mem	I	11	-0.96715	-1.51532	-0.83067	-1.3196	-0.98164	6.457586	52.88493	1.24E-05	0.000116	1
9096_at	9096	ENSG0000(	TBX18	T-box 18	I	22	-0.89087	-1.51991	-0.98465	-1.40874	-0.91357	3.976324	79.42703	2.86E-06	4.13E-05	1
2161_at	2161	ENSG0000(	F12	coagulatior peptidase	I	10	-1.22724	-1.2086	-1.06927	-1.29679	-1.07519	6.07781	86.72331	2.08E-06	3.33E-05	1
55009_at	55009	ENSG0000(	C19orf24	chromosome 19 open	I	11	-1.00282	-1.17796	-1.18977	-1.18212	-1.17297	8.749613	107.4538	9.52E-07	2.02E-05	1
1201_at	1201	ENSG0000(	CLN3	ceroid-lipofuscinosis, r	I	65	-0.97154	-1.21463	-1.10521	-1.30207	-1.26628	5.921713	87.29443	2.03E-06	3.28E-05	1
80185_at	80185	ENSG0000(	C8orf41	chromosome 8 open re	I	38	-0.9198	-1.35755	-1.27617	-1.22454	-1.04648	6.302412	150.8291	2.75E-07	8.96E-06	1
10609_at	10609	NA	LEPREL4	leprecan-lil Secreted	I	27	-1.0123	-1.41437	-1.36448	-1.24186	-0.99037	5.689241	109.3519	8.93E-07	1.95E-05	1
151613_at	151613	ENSG0000(	TTC14	tetratricopeptide repea	I	19	-1.08916	-1.43177	-1.23328	-1.32959	-0.82963	7.264021	125.3	5.43E-07	1.40E-05	1
2180_at	2180	ENSG0000(	ACSL1	acyl-CoA synthetase lo	I	22	-1.00277	-1.42024	-1.08938	-1.21081	-0.81951	6.843417	85.87935	2.15E-06	3.42E-05	1
65258_at	65258	ENSG0000(	MPPE1	metallophosphoestera	I	21	-0.96672	-1.39226	-1.1419	-1.33941	-0.95585	4.772885	127.0505	5.16E-07	1.35E-05	1
23317_at	23317	ENSG0000(	DNAJC13	DnaJ (Hsp40) homolog	I	11	-1.09293	-1.5731	-0.67594	-1.17879	-0.40435	6.269562	123.3367	5.75E-07	1.46E-05	1
55773_at	55773	ENSG0000(	TBC1D23	TBC1 doma Secreted	I	20	-1.22849	-1.77711	-0.89177	-0.97287	-0.64039	7.608825	110.9281	8.47E-07	1.88E-05	1
137392_at	137392	ENSG0000(	FAM92A1	family with sequence s	I	4	-1.29263	-1.68633	-0.96741	-0.95061	-0.34543	8.255903	28.02448	0.000117	0.000637	1
6840_at	6840	ENSG0000(	SVIL	supervillin	I	30	-1.38501	-1.64027	-1.00064	-0.88861	-0.42635	8.384683	103.6887	1.08E-06	2.19E-05	1
28981_at	28981	ENSG0000(	IFT81	intraflagellar transport	I	21	-1.01404	-1.58654	-0.80495	-0.89403	-0.51573	4.866403	95.89002	1.44E-06	2.59E-05	1
10165_at	10165	ENSG0000(	SLC25A13	solute carri transporte	I	29	-0.89734	-1.41282	-0.94083	-1.0201	-0.58743	6.970395	81.67336	2.58E-06	3.85E-05	1
256356_at	256356	ENSG0000(	GK5	glycerol kir kinase	NI	19	-0.90213	-1.3919	-0.98416	-0.77926	-0.39978	4.364063	49.29229	1.60E-05	0.00014	1
51010_at	51010	ENSG0000(	EXOSC3	exosome component 3	NI	13	-0.91445	-1.34948	-0.9209	-0.92643	-0.34841	6.523508	47.21766	1.86E-05	0.000156	1
7993_at	7993	NA	UBXN8	UBX domai Secreted	I	18	-0.98418	-1.32821	-0.89544	-0.92243	-0.46395	7.482439	76.22483	3.32E-06	4.59E-05	1
800_at	800	ENSG0000(	CALD1	caldesmon 1	I	96	-1.03686	-1.45082	-1.17738	-0.9796	-0.32486	6.601505	112.3214	8.10E-07	1.82E-05	1
51163_at	51163	ENSG0000(	DBR1	debranchir Secreted	I	11	-1.11893	-1.54834	-1.14058	-0.90939	-0.4094	5.545371	27.67245	0.000122	0.000659	1
6909_at	6909	ENSG0000(	TBX2	T-box 2	NI	11	-0.86192	-1.547	-1.11574	-0.95677	-0.57905	3.863621	20.02559	0.00037	0.001592	1
641_at	641	ENSG0000(	BLM	Bloom syndrome, RecC	I	11	-0.95461	-1.57543	-1.19863	-1.03681	-0.64543	7.340231	96.57322	1.40E-06	2.55E-05	1
7586_at	7586	ENSG0000(	ZKSCAN1	zinc finger with KRAB a	I	53	-1.08871	-1.47891	-0.92756	-1.22728	-0.57777	4.871544	55.11275	1.07E-05	0.000104	1
8458_at	8458	ENSG0000(	TTF2	transcription terminati	I	39	-1.11063	-1.62613	-1.03414	-1.2107	-0.59685	7.223183	159.7403	2.23E-07	8.02E-06	1
23008_at	23008	ENSG0000(	KLHDC10	kelch domain containir	I	39	-1.04494	-1.506	-1.17289	-1.14723	-0.66397	5.486856	98.48446	1.31E-06	2.42E-05	1
56886_at	56886	ENSG0000(	UGGT1	UDP-glucos Secreted	I	31	-1.15327	-1.49724	-1.1084	-1.21299	-0.69655	5.721418	158.6031	2.29E-07	8.07E-06	1
54956_at	54956	ENSG0000(	PARP16	poly (ADP-ribose) poly	I	11	-0.94008	-1.74343	-1.16966	-1.07312	-1.10526	5.636329	97.83917	1.34E-06	2.47E-05	1
117145_at	117145	ENSG0000(	THEM4	thioesterase superfam	I	10	-1.1684	-1.87242	-1.06985	-1.00431	-0.97959	5.854179	55.43464	1.05E-05	0.000102	1
83943_at	83943	ENSG0000(	IMMP2L	IMP2 inner peptidase	I	11	-1.41014	-1.82402	-0.96804	-1.2397	-0.68626	6.519115	45.04721	2.20E-05	0.000177	1
54875_at	54875	ENSG0000(	CNTLN	centlein, ce kinase	I	30	-1.29972	-1.85254	-1.00179	-1.35247	-0.81733	4.047795	53.35639	1.20E-05	0.000113	1
8548_at	8548	ENSG0000(	BLZF1	basic leucine zipper nu	I	22	-1.17195	-1.65316	-1.09265	-1.40324	-0.92381	4.996364	64.2741	6.15E-06	7.01E-05	1
523_at	523	ENSG0000(	ATP6V1A	ATPase, H+ transporte	I	52	-1.19188	-1.67857	-1.15353	-1.20275	-0.80359	7.309011	177.7801	1.50E-07	6.45E-06	1
64222_at	64222	ENSG0000(	TOR3A	torsin fami Secreted	I	16	-1.31822	-1.63336	-1.11922	-1.27119	-0.79758	6.480223	73.25035	3.84E-06	5.03E-05	1
51319_at	51319	ENSG0000(	RSRC1	arginine/serine-rich co	I	10	-1.23242	-1.16593	-0.89177	-1.12577	-0.70447	7.633389	74.20658	3.66E-06	4.88E-05	1
80119_at	80119	ENSG0000(	PIF1	PIF1 5'-to-3' DNA helic	I	11	-1.09814	-1.24592	-0.93006	-1.36211	-0.75054	7.113818	144.5533	3.21E-07	9.91E-06	1
57568_at	57568	ENSG0000(	SIPA1L2	signal-induced prolifer	I	33	-1.35661	-1.42097	-0.70298	-1.15913	-1.06023	4.387966	25.33841	0.000166	0.000833	1
5567_at	5567	ENSG0000(	PRKACB	protein kin kinase	I	44	-1.09987	-1.52991	-0.87799	-1.07659	-0.99112	4.212548	142.511	3.39E-07	1.02E-05	1
374659_at	374659	ENSG0000(	HDDC3	HD domain containing	I	11	-1.23916	-1.4853	-0.94133	-1.12377	-0.92104	7.557963	138.315	3.78E-07	1.09E-05	1
196394_at	196394	ENSG0000(	AMN1	antagonist of mitotic e	I	21	-1.13743	-1.29308	-0.74367	-1.19174	-0.97438	5.303583	24.53477	0.000185	0.000911	1
7372_at	7372	ENSG0000(	UMPS	uridine monophosphat	I	33	-1.10445	-1.34244	-0.95112	-1.08089	-0.93215	7.010716	116.163	7.16E-07	1.67E-05	1
2791_at	2791	ENSG0000(	GNG11	guanine nu GTPase	I	10	-1.00507	-1.28697	-0.86724	-1.11657	-0.90841	11.31624	72.03518	4.07E-06	5.24E-05	1
79659_at	79659	ENSG0000(	DYNC2H1	dynein, cytoplasmic 2,	I	11	-1.06799	-1.32504	-0.79809	-1.04449	-0.98161	6.846794	21.93937	0.000272	0.001241	1
157570_at	157570	ENSG0000(	ESCO2	establishment of cohe:	I	11	-1.38011	-1.35895	-0.78972	-0.95111	-0.51483	8.507104	72.73523	3.93E-06	5.13E-05	1
64746_at	64746	ENSG0000(	ACBD3	acyl-CoA binding doma	I	21	-1.37062	-1.31437	-0.82987	-1.06275	-0.59927	6.918018	110.7238	8.53E-07	1.89E-05	1
9839_at	9839	ENSG0000(	ZEB2	zinc finger E-box bindi	NI	11	-1.38183	-1.45337	-0.64905	-0.95706	-0.64388	3.841364	18.08011	0.000522	0.002107	1
5934_at	5934	ENSG0000(	RBL2	retinoblastoma-like 2 (	I	36	-1.41349	-1.43787	-0.58071	-1.0453	-0.75887	5.040327	90.76953	1.76E-06	2.97E-05	1
5264_at	5264	ENSG0000(	PHYH	phytanoyl-CoA 2-hydr	I	9	-1.43536	-1.51882	-0.6458	-0.97707	-0.78366	8.467888	99.42521	1.26E-06	2.38E-05	1
116448_at	116448	ENSG0000(	OLIG1	oligodendrocyte transc	NI	11	-1.15412	-1.32393	-0.58292	-0.81445	-0.87036	3.602937	90.28221	1.80E-06	3.01E-05	1
5558_at	5558	NA	PRIM2	primase, DNA, polypep	I	8	-1.1767	-1.19223	-0.70352	-0.88604	-0.93878	6.236575	65.08672	5.88E-06	6.81E-05	1
90_at	90	ENSG0000(	ACVR1	activin A re kinase	I	42	-1.27182	-1.26876	-0.74884	-0.95487	-0.93358	6.161681	99.3572	1.27E-06	2.38E-05	1
11046_at	11046	ENSG0000(	SLC35D2	solute carri transporte	I	11	-1.34812	-1.35601	-0.81792	-0.77316	-0.98701	7.371456	45.08802	2.19E-05	0.000177	1
83479_at	83479	ENSG0000(	DDX59	DEAD (Asp-Glu-Ala-As	I	42	-1.39037	-1.16145	-0.83869	-0.78685	-0.86411	6.455783	121.944	5.99E-07	1.50E-05	1
57103_at	57103	ENSG0000(	C12orf5	chromosome 12 open	I	21	-1.39147	-1.22387	-0.82093	-0.78336	-0.95928	5.764493	122.8201	5.84E-07	1.47E-05	1
3790_at	3790	ENSG0000(	KCNS3	potassium ion channe	I	11	-1.25708	-1.2912	-1.03447	-0.99968	-0.54084	6.704539	60.63302	7.59E-06	8.07E-05	1
1854_at	1854	ENSG0000(	DUT	deoxyuridine triphospl	I	35	-1.38208	-1.35203	-1.05816	-1.10688	-0.54057	8.019125	120.3929	6.28E-07	1.53E-05	1
116496_at	116496	ENSG0000(	FAM129A	family with sequence s	I	33	-1.36476	-1.47047	-0.93686	-1.09228	-0.63934	7.420998	26.7686	0.000137	0.00072	1
11073_at	11073	ENSG0000(	TOPBP1	topoisomerase (DNA) I	I	16	-1.3698	-1.38171	-1.05011	-1.21333	-0.68166	9.016922	101.6324	1.17E-06	2.27E-05	1
4644_at	4644	ENSG0000(	MYO5A	myosin VA (heavy chai	I	22	-1.33805	-1.60531	-1.0291	-1.01274	-0.68432	7.217796	35.2044	5.27E-05	0.000343	1
83879_at	83879	ENSG0000(	CDCA7	cell division cycle asso	I	20	-1.35733	-1.54251	-1.07841	-1.12385	-0.75287	6.78447	106.8925	9.70E-07	2.04E-05	1
84191_at	84191	ENSG0000(	FAM96A	family with sequence s	I	14	-1.46657	-1.56357	-1.03584	-1.02305	-0.53425	9.815261	113.6228	7.76E-07	1.77E-05	1
10550_at	10550	ENSG0000(	ARL6IP5	ADP-ribosylation-like f	I	35	-1.49713	-1.48587	-1.129	-0.97663	-0.6864	10.35804	163.2964	2.05E-07	7.66E-06	1
58499_at	58499	ENSG0000(	ZNF462	zinc finger protein 462	I	21	-1.5027	-1.4159	-1.01582	-1.02089	-0.57747	7.517845	142.8635	3.36E-07	1.01E-05	1
57149_at	57149	ENSG0000(	LYRM1	LYR motif containing 1	I	22	-1.58949	-1.49	-1.0499	-1.08696	-0.61985	7.602437	89.33179	1.87E-06	3.07E-05	1
55215_at	55215	ENSG0000(	FANCI	Fanconi anemia, comp	I	27	-1.32595	-1.41361	-1.25998	-1.12914	-0.85138	7.611887	136.2786	3.99E-07	1.13E-05	1
56683_at	56683	ENSG0000(	C21orf59	chromosome 21 open	I	21	-1.29849	-1.56017	-1.24148	-1.16358	-0.83032	10.13973	181.4594	1.39E-07	6.18E-06	1
55471_at	55471	ENSG0000(	C2orf56	chromosor Secreted	I	33	-1.44655	-1.37543	-1.22598	-0.98257	-0.77257	5.846611	109.1636	8.98E-07	1.96E-05	1
2065_at	2065	ENSG0000(	ERBB3	v-erb-b2 er kinase	I	33	-1.19696	-1.38935	-1.16151	-0.90501	-0.76414	4.286971	55.99712	1.01E-05	9.96E-05	1
3224_at	3224	ENSG0000(	HOXC8	homeobox C8	I	22	-1.21438	-1.48026	-1.26141	-0.88594	-0.86276	4.616015	61.41884	7.25E-06	7.83E-05	1
60561_at	60561	ENSG0000(	RINT1	RAD50 interactor 1	I	22	-1.10224	-1.34978	-0.98448	-0.88744	-0.78178	6.889479	120.9477	6.18E-07	1.52E-05	1
1508_at	1508	ENSG0000(	CTSB	cathepsin E peptidase	I	42	-1.18487	-1.36914	-0.92044	-1.02842	-0.65293	10.03437	141.4916	3.48E-07	1.03E-05	1
55608_at	55608	ENSG0000(	ANKRD10	ankyrin repeat domain	I	11	-1.25275	-1.25595	-0.99584	-1.00637	-0.93706	7.328295	27.96562	0.000118	0.000641	1
100505792	1.01E+08	NA	LOC10050!	hypothetical LOC1005(	I	6	-1.33616	-1.34916	-1.08124	-0.96833	-0.83856	8.33665	112.8972	7.95E-07	1.80E-05	1
7270_at	7270	ENSG0000(	TTF1	transcription terminati	I	31	-1.09948	-1.1344	-1.06651	-0.99317	-0.82756	7.249278	89.91177	1.82E-06	3.04E-05	1
79719_at	79719	ENSG0000(	AAGAB	alpha- and Secreted	I	29	-1.18716	-1.16956	-1.18004	-0.89459	-0.85795	6.728063	90.9661	1.75E-06	2.96E-05	1
84904_at	84904	ENSG0000(	C9orf100	chromosome 9 open re	I	32	-1.14096	-1.29197	-1.21067	-0.94159	-0.88698	4.801405	63.43599	6.45E-06	7.24E-05	1
8530_at	8530	ENSG0000(	CST7	cystatin F (Secreted	I	9	-0.76904	-1.01767	-0.4075	-1.69941	-1.57601	4.85981	15.07673	0.000954	0.003463	1
128646_at	128646	ENSG0000(	SIRPD	signal-regulatory prote	I	10	-0.84824	-1.01193	-0.77961	-1.58588	-1.48961	4.336717	115.9503	7.21E-07	1.68E-05	1
1428_at	1428	ENSG0000(	CRYM	crystallin, mu	I	11	0.012052	-1.25431	-0.69887	-2.04675	-1.59991	4.775482	62.45115	6.82E-06	7.54E-05	1
5651_at	5651	ENSG0000(	TMPRSS15	transmemb peptidase	I	21	-0.30003	-0.85402	-1.08622	-1.63903	-2.06685	4.577269	31.94475	7.41E-05	0.000446	1

3707_at	3707	ENSG0000(	ITPKB	inositol-tris	kinase	I	20	-0.92293	-1.06426	-1.10406	-1.17582	-1.95858	5.369961	101.2374	1.18E-06	2.29E-05		1
4908_at	4908	ENSG0000(	NTF3	neurotropł	Secreted	NI	11	-1.00753	-1.35927	-0.8551	-1.27823	-1.98601	4.581624	103.5728	1.09E-06	2.19E-05		1
3012_at	3012	NA	HIST1H2AE	histone cluster 1, H2ae		NI	22	-0.76407	-1.19008	-0.96427	-0.79259	-1.52936	4.181893	73.44758	3.80E-06	5.00E-05		1
770_at	770	ENSG0000(	CA11	carbonic ar	Secreted	NI	7	-1.0283	-1.10559	-0.95391	-0.96731	-1.68363	5.270783	34.96006	5.40E-05	0.000349		1
26270_at	26270	ENSG0000(	FBXO6	F-box protein 6		I	10	-1.11509	-1.23804	-1.00113	-0.90818	-1.65913	5.050532	73.28647	3.83E-06	5.03E-05		1
126295_at	126295	ENSG0000(	ZNF57	zinc finger protein 57		I	11	-1.09462	-1.21098	-1.0987	-0.96389	-1.67556	5.675882	146.3872	3.07E-07	9.69E-06		1
9638_at	9638	ENSG0000(	FEZ1	fasciculation and elong		NI	32	-1.28058	-1.44326	-0.71796	-0.96997	-1.54297	4.19493	104.7499	1.04E-06	2.13E-05		1
1369_at	1369	ENSG0000(	CPN1	carboxypep	peptidase	NI	10	-0.86371	-1.52883	-0.87632	-1.04599	-1.55903	4.128206	29.9284	9.31E-05	0.000534		1
9170_at	9170	ENSG0000(	LPAR2	lysophosph	G-protein c	I	11	-1.02373	-1.50149	-1.1273	-1.01635	-1.52673	5.195255	68.85024	4.80E-06	5.87E-05		1
27148_at	27148	ENSG0000(	STK36	serine/thre	kinase	I	11	-1.11116	-1.46558	-1.18625	-1.04083	-1.42364	6.245923	43.05172	2.59E-05	0.0002		1
79873_at	79873	ENSG0000(	NUDT18	nudix (nucl	Secreted	I	11	-0.79954	-0.69435	-1.01855	-0.98122	-1.8703	4.68397	72.30485	4.02E-06	5.20E-05		1
400566_at	400566	NA	C17orf97	chromosome 17 open		NI	11	-0.69495	-0.65548	-1.45527	-1.32367	-1.58273	4.747592	115.9189	7.21E-07	1.68E-05		1
55423_at	55423	ENSG0000(	SIRPG	signal-regulatory prote		I	22	-0.88794	-0.7609	-1.42492	-1.18804	-1.38201	4.104054	32.55747	6.93E-05	0.000423		1
55190_at	55190	ENSG0000(	NUDT11	nudix (nucleoside diph		I	11	-0.89493	-0.59186	-1.48613	-1.17882	-1.54121	4.528132	64.27215	6.15E-06	7.01E-05		1
6233_at	6233	ENSG0000(	RPS27A	ribosomal protein S27a		I	8	-1.03489	-0.76487	-1.76179	-1.39235	-1.26228	4.89141	23.67244	0.000209	0.001006		1
57491_at	57491	ENSG0000(	AHRR	aryl-hydrocarbon recep		NI	22	-1.0918	-0.67649	-1.61201	-1.21346	-0.93418	5.101088	101.3203	1.18E-06	2.29E-05		1
24146_at	24146	ENSG0000(	CLDN15	claudin 15		I	9	-1.02271	-0.71332	-1.56112	-1.00155	-0.92046	6.349237	14.20249	0.001159	0.004074		1
6764_at	6764	ENSG0000(	ST5	suppression of tumorig		NI	28	-1.01562	-0.90281	-1.57769	-0.94987	-1.0323	4.691675	80.38126	2.74E-06	4.01E-05		1
57190_at	57190	ENSG0000(	SEPN1	selenoprot	Secreted	NI	11	-1.22115	-0.39952	-1.27718	-1.09451	-1.11686	5.252872	23.67448	0.000209	0.001006		1
11182_at	11182	NA	SLC2A6	solute carri	transporter	I	29	-0.87357	-0.51103	-1.42737	-1.04518	-1.15365	7.021073	27.15635	0.00013	0.000693		1
4582_at	4582	ENSG0000(	MUC1	mucin 1, ce	Secreted	NI	109	-1.00894	-0.6563	-1.43325	-0.98898	-1.10725	4.756621	123.6992	5.69E-07	1.45E-05		1
29780_at	29780	ENSG0000(	PARVB	parvin, beta		I	8	-1.27321	-0.5306	-1.2866	-1.34662	-1.54491	4.224462	37.74046	4.12E-05	0.000285		1
284615_at	284615	ENSG0000(	ANKRD34A	ankyrin repeat domain		NI	11	-1.10609	-0.50007	-1.05849	-1.12699	-1.34498	4.260593	29.78739	9.46E-05	0.000541		1
54913_at	54913	ENSG0000(	RPP25	ribonuclease P/MRP 25		I	11	-1.30578	-0.71295	-1.17545	-1.05337	-1.40631	6.247441	35.50348	5.11E-05	0.000336		1
56477_at	56477	ENSG0000(	CCL28	chemokine	Secreted	I	13	-1.13496	-0.98689	-1.22573	-1.4555	-1.39722	4.510903	43.66819	2.46E-05	0.000192		1
80227_at	80227	ENSG0000(	PAAF1	proteasomal ATPase-a		I	28	-1.41439	-1.02011	-1.29162	-1.27781	-1.28271	7.965347	64.85449	5.96E-06	6.86E-05		1
80210_at	80210	ENSG0000(	ARMC9	armadillo repeat conta		I	9	-1.46755	-1.06574	-1.15149	-1.44253	-1.37518	6.864694	57.88341	8.97E-06	9.13E-05		1
55620_at	55620	ENSG0000(	STAP2	signal transducing ada		I	19	-1.27902	-1.04154	-1.16196	-1.14792	-1.26902	6.750773	135.9842	4.02E-07	1.13E-05		1
10069_at	10069	ENSG0000(	RWDD2B	RWD domain containir		I	11	-1.23974	-1.01795	-1.14422	-1.14615	-1.39797	8.045107	55.03958	1.07E-05	0.000104		1
6713_at	6713	ENSG0000(	SQLE	squalene epoxidase		I	33	-1.20988	-1.04338	-1.30759	-1.25002	-1.29391	7.513261	16.86733	0.000658	0.002549		1
26276_at	26276	ENSG0000(	VPS33B	vacuolar protein sortin		I	18	-1.14315	-1.02151	-1.29053	-1.17968	-1.4357	6.971407	122.4647	5.90E-07	1.48E-05		1
10514_at	10514	ENSG0000(	MYBBP1A	MYB binding protein (F		I	50	-1.14208	-1.09521	-1.2173	-1.19121	-1.43684	6.309084	148.8618	2.89E-07	9.25E-06		1
57447_at	57447	ENSG0000(	NDRG2	NDRG family member		I	22	-1.46572	-0.74623	-1.35878	-1.41446	-1.26154	5.166235	141.0801	3.51E-07	1.04E-05		1
348801_at	348801	ENSG0000(	LNP1	leukemia NUP98 fusior		I	11	-1.52031	-0.67136	-1.44333	-1.39792	-1.34257	5.476776	19.3618	0.000415	0.001747		1
2817_at	2817	ENSG0000(	GPC1	glypican 1	Secreted	I	21	-1.44758	-1.06983	-1.6229	-1.00604	-1.44563	4.640304	63.57863	6.40E-06	7.19E-05		1
23616_at	23616	ENSG0000(	SH3BP1	SH3-domai	Secreted	I	32	-1.50313	-1.11406	-1.49833	-1.08691	-1.61304	5.481538	94.73603	1.51E-06	2.67E-05		1
25911_at	25911	ENSG0000(	DPCD	deleted in primary cilia		I	9	-1.4402	-1.204	-1.46844	-1.30528	-1.54338	8.641375	189.49	1.19E-07	5.63E-06		1
5092_at	5092	ENSG0000(	PCBD1	pterin-4 alpha-carbino		I	10	-1.30892	-1.22801	-1.53969	-1.40303	-1.57883	7.808601	67.61561	5.12E-06	6.15E-05		1
1020_at	1020	ENSG0000(	CDK5	cyclin-depe	kinase	I	13	-1.59685	-1.18486	-1.51213	-1.23664	-1.44223	7.730608	57.49198	9.19E-06	9.30E-05		1
283537_at	283537	ENSG0000(	SLC46A3	solute carrier family 46		I	11	-1.63788	-1.154	-1.56907	-1.42861	-1.62377	6.167916	134.1692	4.22E-07	1.18E-05		1
241_at	241	ENSG0000(	ALOX5AP	arachidonate 5-lipoxyg		NI	10	-1.1082	-1.79664	-1.85578	-0.88702	-0.87911	4.881957	18.37573	0.000495	0.002019		1
375035_at	375035	ENSG0000(	SFT2D2	SFT2 domain containin		I	9	-1.25995	-1.93874	-1.63376	-1.10964	-0.79448	4.744987	97.07049	1.38E-06	2.51E-05		1
30849_at	30849	ENSG0000(	PIK3R4	phosphoin	kinase	I	10	-1.26802	-1.86792	-1.39486	-1.26512	-0.95591	8.032766	222.4044	6.59E-08	4.12E-06		1
10451_at	10451	ENSG0000(	VAV3	vav 3 guanine nucleoti		I	54	-1.41289	-1.3788	-1.44207	-1.10377	-0.83752	4.683607	158.6594	2.28E-07	8.07E-06		1
10521_at	10521	ENSG0000(	DDX17	DEAD (Asp-Glu-Ala-Asp		I	30	-1.24859	-1.25035	-1.42258	-1.15695	-0.72776	5.771296	142.1669	3.42E-07	1.02E-05		1
55740_at	55740	ENSG0000(	ENAH	enabled homolog (Dro		I	28	-1.28352	-1.37101	-1.43336	-1.09184	-0.75901	5.058603	105.975	1.00E-06	2.08E-05		1
84734_at	84734	ENSG0000(	FAM167B	family with sequence s		I	11	-1.16133	-1.26438	-1.58666	-1.14904	-0.85591	4.524176	42.56929	2.69E-05	0.000206		1
115509_at	115509	ENSG0000(	ZNF689	zinc finger protein 689		I	11	-1.156	-1.42715	-1.55832	-1.26132	-0.73256	5.890034	158.5252	2.29E-07	8.07E-06		1
81875_at	81875	ENSG0000(	ISG20L2	interferon stimulated e		I	7	-1.11434	-1.41702	-1.55889	-1.2775	-0.9166	8.628371	134.4698	4.19E-07	1.17E-05		1
10478_at	10478	ENSG0000(	SLC25A17	solute carri	transporter	I	21	-1.11011	-1.33076	-1.39089	-1.04015	-1.05505	6.651799	170.2799	1.76E-07	7.03E-06		1
898_at	898	ENSG0000(	CCNE1	cyclin E1		I	22	-1.17004	-1.3826	-1.40719	-1.02029	-1.01472	6.394048	168.4302	1.83E-07	7.15E-06		1
29990_at	29990	ENSG0000(	PILRB	paired immunoglobin-		I	31	-1.32203	-1.39027	-1.33249	-1.05186	-1.06548	6.902391	68.00742	5.02E-06	6.06E-05		1
55157_at	55157	ENSG0000(	DARS2	aspartyl-tRNA syntheta		I	21	-1.38805	-1.37556	-1.46164	-1.08453	-1.00271	7.356513	144.3071	3.23E-07	9.92E-06		1
51115_at	51115	ENSG0000(	FAM82B	family with sequence s		I	16	-1.08341	-1.54076	-1.36594	-1.24943	-1.06039	8.402963	100.3861	1.22E-06	2.34E-05		1
526_at	526	ENSG0000(	ATP6V1B2	ATPase, H+	transporter	I	28	-1.23286	-1.50219	-1.40909	-1.11631	-1.04109	8.744675	102.2404	1.14E-06	2.24E-05		1
5873_at	5873	ENSG0000(	RAB27A	RAB27A, m	GTPase	I	59	-1.25332	-1.54196	-1.34357	-1.34332	-0.92616	7.817301	106.8835	9.70E-07	2.04E-05		1
54471_at	54471	ENSG0000(	SMCR7L	Smith-Mag	Secreted	I	33	-1.23587	-1.55191	-1.42528	-1.28047	-1.10326	7.671192	200.0584	9.74E-08	5.17E-06		1
9319_at	9319	ENSG0000(	TRIP13	thyroid hormone recep		I	21	-1.3443	-1.49194	-1.43294	-1.28747	-1.09838	8.32659	148.2841	2.93E-07	9.35E-06		1
8877_at	8877	ENSG0000(	SPHK1	sphingosin	kinase	I	11	-0.61578	-1.28825	-1.62806	-1.39853	-1.21175	6.193024	91.2299	1.73E-06	2.94E-05		1
3399_at	3399	ENSG0000(	ID3	inhibitor of	Secreted	I	28	-0.75602	-1.54468	-1.57964	-1.6297	-1.17418	5.877729	23.10302	0.000228	0.001075		1
1244_at	1244	ENSG0000(	ABCC2	ATP-bindin	transporter	I	10	-0.7281	-1.51231	-1.27123	-1.53326	-1.42116	4.859828	74.42897	3.62E-06	4.85E-05		1
8764_at	8764	ENSG0000(	TNFRSF14	tumor necrosis factor r		I	56	-0.85108	-1.43614	-1.51568	-1.4904	-1.42368	5.315222	159.1209	2.26E-07	8.07E-06		1
90853_at	90853	ENSG0000(	SPOCD1	SPOC domain containi		I	11	-0.89685	-1.67747	-1.54079	-1.4346	-1.53252	5.957371	87.70606	1.99E-06	3.23E-05		1
55080_at	55080	ENSG0000(	TAPBPL	TAP binding protein-lik		I	20	-0.85825	-1.74412	-1.61193	-1.48781	-1.49297	5.053431	95.75618	1.45E-06	2.59E-05		1
3214_at	3214	ENSG0000(	HOXB4	homeobox B4		I	19	-1.32797	-1.31478	-1.84369	-1.58001	-1.34747	4.711696	67.52183	5.15E-06	6.16E-05		1
3305_at	3305	NA	HSPA1L	heat shock 70kDa prot		I	11	-1.19725	-1.53138	-1.81441	-1.04664	-1.21728	4.664833	63.44822	6.45E-06	7.24E-05		1
10171_at	10171	ENSG0000(	RCL1	RNA terminal phospha		I	9	-1.11774	-1.33211	-1.83368	-1.3122	-1.37963	6.690697	107.8896	9.38E-07	2.00E-05		1
148523_at	148523	ENSG0000(	C1orf51	chromosome 1 open re		I	11	-1.36451	-1.34188	-1.82976	-1.12419	-1.48846	5.107459	129.5858	4.80E-07	1.29E-05		1
27043_at	27043	ENSG0000(	PELP1	proline, glu	Secreted	I	22	-1.11747	-0.99071	-1.68134	-0.88693	-1.39055	5.540284	81.32493	2.63E-06	3.90E-05		1
10536_at	10536	ENSG0000(	LEPREL2	leprecan-lil	Secreted	I	33	-1.23567	-0.91491	-1.69778	-1.17121	-1.60243	4.457904	103.1132	1.11E-06	2.21E-05		1
26301_at	26301	ENSG0000(	GBGT1	globoside alpha-1,3-N-		I	22	-1.2644	-1.00611	-1.80672	-1.31197	-1.38817	4.616439	196.9588	1.03E-07	5.26E-06		1
4833_at	4833	ENSG0000(	NME4	non-metas	kinase	I	7	-0.86125	-1.21597	-1.29721	-1.24367	-1.3088	8.120418	67.78598	5.08E-06	6.12E-05		1
114880_at	114880	ENSG0000(	OSBPL6	oxysterol binding prot		I	29	-0.88024	-1.35303	-1.37993	-1.24748	-1.23962	5.982967	132.1259	4.47E-07	1.23E-05		1
2022_at	2022	ENSG0000(	ENG	endoglin	Secreted	I	51	-0.87455	-1.23109	-1.44375	-1.34109	-1.25716	5.525264	53.90999	1.16E-05	0.00011		1
51106_at	51106	ENSG0000(	TFB1M	transcription factor B1		I	11	-1.05662	-1.27782	-1.45264	-1.48333	-1.09293	7.442211	56.54417	9.76E-06	9.73E-05		1
9463_at	9463	ENSG0000(	PICK1	protein interacting wit		I	11	-1.00026	-1.07717	-1.42873	-1.49665	-1.42539	6.175924	101.3951	1.18E-06	2.29E-05		1
84166_at	84166	ENSG0000(	NLRC5	NLR family, CARD dom		I	22	-0.97792	-1.17157	-1.574	-1.51496	-1.40915	4.767946	86.67984	2.08E-06	3.33E-05		1
7059_at	7059	ENSG0000(	THBS3	thrombosp	Secreted	I	17	-1.09542	-1.01004	-1.44796	-1.08361	-1.42332	4.416069	54.26757	1.13E-05	0.000108		1
23649_at	23649	ENSG0000(	POLA2	polymerase (DNA direc		I	19	-1.06568	-1.11629	-1.36362	-1.12714	-1.31927	6.524808	50.53538	1.46E-05	0.00013		1
3140_at	3140	ENSG0000(	MR1	major histocompatibili		NI	47	-1.15805	-1.10805	-1.34089	-1.10822	-1.35529	4.533271	40.35633	3.25E-05	0.000238		1
1459_at	1459	ENSG0000(	CSNK2A2	casein kina	kinase	I	8	-1.08567	-1.30157	-1.52395	-1.06376	-1.49513	7.658573	107.3312	9.56E-07	2.03E-05		1
5175_at	5175	ENSG0000(	PECAM1	platelet/endothelial ce		I	12	-1.14642	-1.23643	-1.31946	-1.11503	-1.60829	5.795739	11.19662	0.002484	0.007705		1

83660_at	83660	ENSG0000(	TLN2	talin 2		I	22	-1.09209	-1.3288	-1.37178	-1.19835	-1.50506	4.958235	83.51352	2.38E-06	3.66E-05	1
9129_at	9129	ENSG0000(	PRPF3	PRP3 pre-mRNA proce		I	10	-1.22679	-1.17273	-1.35628	-1.11471	-1.10909	6.858276	43.87853	2.42E-05	0.000189	1
55127_at	55127	ENSG0000(	HEATR1	HEAT repeat containin		I	20	-1.19387	-1.21796	-1.35871	-1.2858	-1.06931	8.370507	108.5486	9.17E-07	1.98E-05	1
84549_at	84549	ENSG0000(	MAK16	MAK16 homolog (S. ce		I	16	-1.11426	-1.22359	-1.46352	-1.17504	-1.1682	8.639309	146.4489	3.06E-07	9.69E-06	1
9688_at	9688	ENSG0000(	NUP93	nucleoporin 93kDa		I	11	-1.09501	-1.18089	-1.51176	-1.20774	-1.21225	10.01144	148.824	2.89E-07	9.25E-06	1
57512_at	57512	ENSG0000(	GPR158	G protein-c	G-protein c	I	22	-1.2862	-1.18388	-1.43485	-1.2796	-1.32881	4.778105	144.0445	3.26E-07	9.92E-06	1
79191_at	79191	ENSG0000(	IRX3	iroquois homeobox 3		I	14	-1.36493	-1.22794	-1.4983	-1.38586	-1.22787	6.45052	43.09579	2.58E-05	0.0002	1
55840_at	55840	ENSG0000(	EAF2	ELL associated factor 2		I	11	-1.45768	-1.23181	-1.35396	-1.14968	-1.26209	5.313234	84.04392	2.33E-06	3.63E-05	1
5432_at	5432	ENSG0000(	POLR2C	polymerase (RNA) II (D		I	23	-1.41391	-1.34091	-1.43047	-1.19308	-1.16513	9.189368	141.5293	3.47E-07	1.03E-05	1
2588_at	2588	ENSG0000(	GALNS	galactosam	Secreted	I	18	-1.32843	-1.31712	-1.37582	-1.21138	-1.21565	6.470605	98.88877	1.29E-06	2.40E-05	1
25813_at	25813	ENSG0000(	SAMM50	sorting and assembly r		I	10	-1.37683	-1.3457	-1.36966	-1.2648	-1.27891	10.40314	180.8253	1.41E-07	6.21E-06	1
10058_at	10058	ENSG0000(	ABCB6	ATP-bindin	transporte	NI	18	-1.44347	-1.14501	-0.87113	-0.95186	-1.43961	5.186004	93.76582	1.56E-06	2.73E-05	1
6767_at	6767	ENSG0000(	ST13	suppressio	Secreted	I	3	-1.51734	-1.22971	-0.87694	-0.86256	-1.09941	8.864951	26.25611	0.000147	0.000759	1
114088_at	114088	ENSG0000(	TRIM9	tripartite motif contair		I	33	-1.5043	-1.30287	-0.8433	-1.03754	-1.12275	3.828478	117.5942	6.85E-07	1.62E-05	1
84284_at	84284	ENSG0000(	NTPCR	nucleoside	kinase	I	20	-1.4464	-1.18847	-0.99704	-1.13386	-1.1994	8.164377	109.9549	8.75E-07	1.92E-05	1
80150_at	80150	ENSG0000(	ASRGL1	asparaginase like 1		I	22	-1.56172	-1.19133	-0.99285	-1.07145	-1.17577	4.808592	99.96258	1.24E-06	2.36E-05	1
64946_at	64946	ENSG0000(	CENPH	centromere protein H		I	9	-1.48259	-1.10399	-0.91378	-1.04753	-0.81844	7.492079	38.06703	4.00E-05	0.000278	1
149345_at	149345	ENSG0000(	SHISA4	shisa homolog 4 (Xeno		I	11	-1.37108	-1.15488	-1.00416	-1.03726	-1.00247	7.403385	37.78662	4.11E-05	0.000284	1
4232_at	4232	ENSG0000(	MEST	mesoderm specific trai		NI	21	-1.51055	-1.1322	-0.93487	-1.03249	-1.03945	5.065899	140.8466	3.54E-07	1.04E-05	1
144501_at	144501	ENSG0000(	KRT80	keratin 80		I	11	-1.39972	-1.00404	-0.76169	-1.16906	-0.93956	7.220336	62.76701	6.70E-06	7.44E-05	1
23729_at	23729	ENSG0000(	SHPK	sedoheptu	kinase	I	20	-1.32644	-1.00893	-0.85195	-1.19439	-1.18869	4.13715	117.2747	6.91E-07	1.63E-05	1
91949_at	91949	ENSG0000(	COG7	component of oligome		NI	10	-1.46909	-1.00062	-0.93025	-1.17446	-1.20115	5.855463	46.05466	2.03E-05	0.000166	1
1265_at	1265	ENSG0000(	CNN2	calponin 2		I	70	-1.42341	-0.94305	-0.96889	-0.9698	-1.1025	5.175032	112.3762	8.08E-07	1.81E-05	1
116447_at	116447	ENSG0000(	TOP1MT	topoisomerase (DNA) I		I	27	-1.39445	-0.94505	-1.1534	-0.9105	-1.0363	7.326209	82.47212	2.49E-06	3.76E-05	1
9121_at	9121	ENSG0000(	SLC16A5	solute carri	transporte	NI	11	-1.35826	-1.05094	-1.10434	-1.13165	-1.04346	4.163612	40.29303	3.27E-05	0.000239	1
682_at	682	ENSG0000(	BSG	basigin (Ok blood grou		I	64	-1.29087	-1.01391	-1.06509	-1.03693	-1.20146	7.996197	75.38323	3.46E-06	4.71E-05	1
55850_at	55850	ENSG0000(	USE1	unconventional SNARE		I	8	-1.21449	-1.03235	-1.09954	-1.09449	-1.12639	8.979463	119.7506	6.41E-07	1.55E-05	1
197370_at	197370	ENSG0000(	NSMCE1	non-SMC e	Secreted	I	19	-1.23992	-1.05593	-1.10026	-1.11263	-1.12628	9.102802	143.749	3.28E-07	9.97E-06	1
115572_at	115572	ENSG0000(	FAM46B	family with sequence s		I	22	-1.5977	-1.22617	-1.47344	-0.96279	-0.87928	5.95246	80.2427	2.76E-06	4.02E-05	1
93664_at	93664	ENSG0000(	CADPS2	Ca++-dependent secre		I	33	-1.75074	-1.42086	-1.38073	-0.94709	-1.10013	5.431935	73.34052	3.82E-06	5.02E-05	1
3223_at	3223	ENSG0000(	HOXC6	homeobox C6		I	33	-1.66865	-1.35287	-1.46038	-1.02571	-1.14831	6.821147	164.2047	2.01E-07	7.55E-06	1
3693_at	3693	ENSG0000(	ITGB5	integrin, be	Secreted	I	11	-1.38413	-1.23831	-1.03786	-0.87538	-1.11429	10.17135	106.8012	9.73E-07	2.04E-05	1
686_at	686	ENSG0000(	BTD	biotinidase	Secreted	I	11	-1.52237	-1.23627	-1.07421	-0.7957	-1.09082	6.13469	132.9658	4.37E-07	1.21E-05	1
8406_at	8406	ENSG0000(	SRPX	sushi-repe	Secreted	I	11	-1.35711	-1.34058	-1.18297	-0.8159	-1.01244	10.86959	108.3799	9.22E-07	1.99E-05	1
2013_at	2013	ENSG0000(	EMP2	epithelial membrane p		I	39	-1.44846	-1.29924	-1.27505	-0.97511	-0.98868	6.467078	95.84041	1.44E-06	2.59E-05	1
4493_at	4493	ENSG0000(	MT1E	metallothionein 1E		I	9	-1.38085	-1.05894	-1.54827	-0.7901	-0.98044	9.945592	83.56105	2.38E-06	3.66E-05	1
10954_at	10954	ENSG0000(	PDIA5	protein dis	Secreted	I	8	-1.50578	-1.07276	-1.47128	-1.05075	-1.20726	8.013885	133.0149	4.36E-07	1.21E-05	1
9197_at	9197	ENSG0000(	SLC33A1	solute carri	transporte	I	31	-1.58075	-1.12819	-1.60988	-1.04247	-1.16917	7.894254	86.61214	2.09E-06	3.34E-05	1
94122_at	94122	ENSG0000(	SYTL5	synaptotagmin-like 5		I	22	-1.41757	-1.40429	-1.30833	-0.82837	-1.38722	4.283191	169.7247	1.78E-07	7.03E-06	1
10404_at	10404	ENSG0000(	PGCP	plasma glu	peptidase	I	20	-1.47071	-1.30303	-1.48655	-0.94336	-1.47653	5.291663	113.7344	7.73E-07	1.76E-05	1
387521_at	387521	ENSG0000(	TMEM189	transmembrane protei		I	33	-1.33622	-1.21506	-1.29344	-0.84436	-1.24567	7.772516	122.9544	5.82E-07	1.47E-05	1
64776_at	64776	ENSG0000(	C11orf1	chromosome 11 open		I	11	-1.38643	-1.25263	-1.27283	-0.99517	-1.24553	8.632309	129.6874	4.78E-07	1.29E-05	1
5351_at	5351	ENSG0000(	PLOD1	procollage	Secreted	I	22	-1.55084	-1.2835	-1.34493	-1.00893	-1.19635	8.042343	34.82117	5.48E-05	0.000354	1
83746_at	83746	ENSG0000(	L3MBTL2	l(3)mbt-like 2 (Drosopł		I	11	-1.42193	-1.3296	-1.41205	-1.03315	-1.25566	5.963691	54.23778	1.13E-05	0.000108	1
4814_at	4814	ENSG0000(	NINJ1	ninjurin 1		I	11	-1.02164	-1.26923	-1.31551	-0.87267	-1.23947	7.072529	54.44779	1.12E-05	0.000107	1
79415_at	79415	ENSG0000(	C17orf62	chromosome 17 open		I	11	-0.99327	-1.1288	-1.37426	-1.02125	-1.20319	8.692485	103.5315	1.09E-06	2.19E-05	1
10126_at	10126	ENSG0000(	DNAL4	dynein, axonemal, ligh		I	19	-1.0402	-1.06762	-1.29798	-0.93214	-1.08892	4.581838	59.97178	7.90E-06	8.32E-05	1
54939_at	54939	ENSG0000(	COMMD4	COMM domain contai		I	10	-0.97566	-1.10276	-1.29357	-1.01031	-1.05234	8.379399	121.103	6.15E-07	1.52E-05	1
54093_at	54093	ENSG0000(	SETD4	SET domain containing		I	22	-1.05569	-1.22202	-1.14378	-0.86531	-1.03176	6.228343	52.18777	1.30E-05	0.00012	1
79447_at	79447	ENSG0000(	C16orf53	chromosome 16 open		I	21	-1.04358	-1.08363	-1.00052	-0.8954	-1.04005	7.315715	80.53507	2.72E-06	3.99E-05	1
373156_at	373156	ENSG0000(	GSTK1	glutathione S-transfera		I	11	-1.0457	-1.03254	-1.0999	-0.88194	-1.048	9.486377	116.1536	7.16E-07	1.67E-05	1
112770_at	112770	ENSG0000(	C1orf85	chromosome 1 open re		I	40	-1.11784	-1.16182	-0.93822	-0.96012	-1.02157	7.683277	115.6266	7.28E-07	1.69E-05	1
10467_at	10467	ENSG0000(	ZNHIT1	zinc finger, HIT-type cc		I	9	-1.15114	-1.16114	-1.01348	-0.90013	-1.09458	10.06381	116.8859	7.00E-07	1.64E-05	1
25873_at	25873	ENSG0000(	RPL36	ribosomal protein L36		I	17	-1.19856	-1.14259	-1.01592	-0.9372	-1.15699	8.147556	88.8527	1.90E-06	3.11E-05	1
6171_at	6171	ENSG0000(	RPL41	ribosomal protein L41		I	11	-1.1922	-1.1437	-1.28981	-0.98821	-0.92756	9.174893	58.95527	8.40E-06	8.70E-05	1
1938_at	1938	ENSG0000(	EEF2	eukaryotic	GTPase	I	77	-1.23955	-1.15297	-1.30124	-1.06018	-0.97263	11.91947	137.0147	3.91E-07	1.12E-05	1
10437_at	10437	ENSG0000(	IFI30	interferon, gamma-ind		I	11	-1.30151	-1.09667	-1.30591	-0.93477	-1.06598	9.851323	33.86988	6.04E-05	0.00038	1
161882_at	161882	ENSG0000(	ZFPM1	zinc finger protein, mu		I	11	-1.28189	-1.22815	-1.3108	-0.89818	-1.08756	5.342036	79.26331	2.88E-06	4.16E-05	1
1652_at	1652	ENSG0000(	DDT	D-dopachrome tautom		I	8	-1.33234	-1.13214	-1.22543	-1.06741	-0.97792	10.82736	92.63215	1.64E-06	2.84E-05	1
8079_at	8079	ENSG0000(	MLF2	myeloid leukemia facto		I	9	-1.28278	-1.24602	-1.23063	-1.07124	-1.04367	8.059611	76.75835	3.24E-06	4.50E-05	1
140707_at	140707	ENSG0000(	BRI3BP	BRI3 binding protein		I	10	-1.36664	-1.2043	-1.15066	-1.09061	-1.02536	5.747869	88.93292	1.90E-06	3.11E-05	1
55164_at	55164	ENSG0000(	SHQ1	SHQ1 homolog (S. cere		I	30	-1.35498	-1.28538	-1.17961	-1.05441	-1.01616	6.944766	159.9057	2.22E-07	8.02E-06	1
10095_at	10095	ENSG0000(	ARPC1B	actin related protein 2,		I	11	-1.15074	-1.27569	-1.21009	-1.07102	-1.10599	11.16047	132.1813	4.46E-07	1.23E-05	1
284129_at	284129	ENSG0000(	SLC26A11	solute carri	transporte	NI	10	-1.16853	-1.17776	-1.15684	-1.00569	-1.05568	5.379583	71.39992	4.21E-06	5.37E-05	1
80011_at	80011	ENSG0000(	FAM192A	family with sequence s		I	24	-1.16651	-1.18642	-1.20258	-1.07764	-1.05476	8.02647	118.2603	6.71E-07	1.60E-05	1
3959_at	3959	ENSG0000(	LGALS3BP	lectin, gala	Secreted	I	11	-1.26	-1.16763	-1.07127	-0.97357	-1.17622	9.598908	131.595	4.54E-07	1.24E-05	1
5510_at	5510	ENSG0000(	PPP1R7	protein phosphatase 1		I	20	-1.22338	-1.18796	-1.13347	-0.99545	-1.17326	8.531521	136.8027	3.93E-07	1.12E-05	1
2806_at	2806	ENSG0000(	GOT2	glutamic-oxaloacetic tr		I	42	-1.17983	-1.19077	-1.1428	-1.09917	-1.151	9.463929	145.7642	3.12E-07	9.71E-06	1
10421_at	10421	ENSG0000(	CD2BP2	CD2 (cytoplasmic tail) I		I	21	-1.1446	-1.17093	-1.15691	-1.07619	-1.22165	7.558817	124.4327	5.57E-07	1.42E-05	1
3588_at	3588	ENSG0000(	IL10RB	interleukin 10 receptor		I	20	-1.23514	-1.22228	-1.19471	-1.01073	-1.12208	7.981292	121.8915	6.00E-07	1.50E-05	1
7169_at	7169	ENSG0000(	TPM2	tropomyosin 2 (beta)		I	28	-1.21477	-1.24468	-1.20932	-0.97602	-1.22894	6.621869	40.5826	3.19E-05	0.000234	1
51200_at	51200	ENSG0000(	CPA4	carboxypep	peptidase	I	33	-0.79072	-1.35582	-1.10766	-1.33857	-1.26049	6.066071	29.60311	9.67E-05	0.00055	1
1745_at	1745	ENSG0000(	DLX1	distal-less homeobox 1		I	21	-0.79352	-1.42498	-1.18146	-1.0816	-1.39701	4.603193	80.32092	2.75E-06	4.01E-05	1
7128_at	7128	ENSG0000(	TNFAIP3	tumor necr	peptidase	I	22	-0.71463	-1.23373	-1.29733	-1.19043	-1.12934	7.001	59.89972	7.93E-06	8.35E-05	1
54331_at	54331	ENSG0000(	GNG2	guanine nucleotide bir		I	33	-0.66507	-1.42488	-1.34128	-1.082	-1.1094	4.250474	150.704	2.76E-07	8.97E-06	1
196513_at	196513	ENSG0000(	DCP1B	DCP1 decapping enzyn		I	9	-0.79853	-1.45326	-1.28208	-1.08858	-1.04599	5.386136	42.16417	2.78E-05	0.000211	1
414_at	414	ENSG0000(	ARSD	arylsulfatase D		NI	21	-0.76443	-1.02875	-1.38522	-1.10389	-0.91146	4.393079	32.73507	6.80E-05	0.000417	1
6621_at	6621	ENSG0000(	SNAPC4	small nucle	Secreted	I	48	-0.87607	-1.03205	-1.4375	-1.03988	-1.02049	7.277475	90.6373	1.77E-06	2.99E-05	1
54865_at	54865	ENSG0000(	GPATCH4	G patch domain contai		I	20	-0.94123	-1.05434	-1.45368	-1.10222	-0.9453	8.407757	121.8666	6.01E-07	1.50E-05	1
10436_at	10436	NA	EMG1	EMG1 nucleolar protei		I	20	-1.10161	-1.08374	-1.23083	-1.22657	-0.98973	10.15782	74.90319	3.54E-06	4.77E-05	1
9570_at	9570	ENSG0000(	GOSR2	golgi SNAP	transporte	I	41	-1.00825	-1.07321	-1.31382	-1.20011	-1.05497	5.965395	124.7668	5.51E-07	1.42E-05	1
25778_at	25778	ENSG0000(	DSTYK	dual serine kinase		I	65	-1.0565	-1.15029	-1.32073	-1.20289	-1.10874	6.023372	151.1457	2.73E-07	8.95E-06	1

25880_at	25880	ENSG0000(	TMEM186	transmembrane protei	I	11	-0.96772	-1.09433	-1.15854	-1.01051	-0.97609	7.88409	129.0542	4.87E-07	1.30E-05	1
80020_at	80020	ENSG0000(	FOXRED2	FAD-dependent oxidor	I	41	-0.88573	-1.10323	-1.24581	-1.03952	-0.9366	7.746604	112.5561	8.03E-07	1.81E-05	1
79663_at	79663	ENSG0000(	HSPBAP1	HSPB (heat shock 27kD	NI	19	-0.8653	-1.21741	-1.09075	-1.15431	-0.94629	4.935192	71.55071	4.18E-06	5.34E-05	1
283951_at	283951	ENSG0000(	C16orf91	chromosome 16 open	I	11	-0.89994	-1.20863	-1.18179	-1.12079	-1.02737	9.042469	129.728	4.78E-07	1.29E-05	1
84752_at	84752	ENSG0000(	B3GNT9	UDP-GlcNAc:betaGal b	NI	11	-0.85088	-1.15125	-1.04678	-0.6278	-1.15723	4.75843	34.30289	5.77E-05	0.000368	1
128439_at	128439	NA	SNHG11	small nucleolar RNA hc	NI	17	-0.80382	-1.37883	-1.18994	-0.67046	-1.24804	6.24979	58.68887	8.54E-06	8.79E-05	1
387787_at	387787	ENSG0000(	LIPT2	lipoyl(octanoyl) transfe	NI	10	-0.96427	-1.42209	-1.11355	-0.75431	-1.09119	4.5948	17.71194	0.000559	0.002235	1
5476_at	5476	ENSG0000(	CTSA	cathepsin / peptidase	I	21	-0.99072	-1.07816	-0.9816	-0.95682	-1.16995	9.909964	119.7391	6.41E-07	1.55E-05	1
90871_at	90871	ENSG0000(	C9orf123	chromosome 9 open re	I	36	-0.97561	-1.00516	-0.87704	-0.94176	-1.16736	4.593218	72.11995	4.06E-06	5.23E-05	1
84269_at	84269	ENSG0000(	CHCHD5	coiled-coil-helix-coiled	I	11	-0.99454	-1.06067	-0.80961	-1.03104	-1.16784	6.261441	60.7492	7.54E-06	8.03E-05	1
283377_at	283377	ENSG0000(	SPRYD4	SPRY domain containir	I	21	-1.0119	-1.22661	-0.8751	-1.09854	-1.337	5.761232	113.8707	7.70E-07	1.76E-05	1
7106_at	7106	ENSG0000(	TSPAN4	tetraspanin 4	I	39	-0.89826	-1.12897	-0.8532	-1.03269	-1.37594	9.261917	115.1083	7.40E-07	1.71E-05	1
80381_at	80381	ENSG0000(	CD276	CD276 molecule	I	22	-0.92323	-1.08751	-0.97285	-1.00576	-1.42506	5.942438	101.7219	1.16E-06	2.27E-05	1
9016_at	9016	ENSG0000(	SLC25A14	solute carri transporter	I	14	-0.79727	-1.16727	-0.86358	-1.04812	-1.12704	6.658414	35.53329	5.10E-05	0.000335	1
22994_at	22994	ENSG0000(	AZI1	5-azacytidine induced	NI	9	-0.84206	-1.25577	-0.81959	-1.09622	-1.14252	5.741604	44.49533	2.30E-05	0.000182	1
7091_at	7091	ENSG0000(	TLE4	transducin-like enhanc	NI	30	-0.96266	-1.2828	-0.89262	-1.23235	-1.07774	4.210652	108.6801	9.13E-07	1.98E-05	1
1174_at	1174	ENSG0000(	AP1S1	adaptor-related protei	I	20	-1.03811	-1.21797	-0.9444	-1.23317	-1.04674	8.262207	84.40897	2.29E-06	3.58E-05	1
57461_at	57461	ENSG0000(	ISY1	ISY1 splicing factor hor	I	18	-1.03043	-1.17767	-0.95591	-1.11708	-0.93498	8.882849	110.5849	8.57E-07	1.89E-05	1
6778_at	6778	ENSG0000(	STAT6	signal trans transporter	I	42	-0.98327	-1.2745	-1.0014	-1.04348	-1.02308	5.210268	87.70724	1.99E-06	3.23E-05	1
9054_at	9054	ENSG0000(	NFS1	NFS1 nitro; Secreted	I	34	-1.05444	-1.28538	-0.95481	-1.04228	-1.02978	7.13901	94.23419	1.54E-06	2.70E-05	1
