# Supplementary material for: Endothelial Protein C Receptor Function in Murine and Human Breast Cancer Development
Source: PLoS One. 2013 Apr 9;8(4):e61071. doi: 10.1371/journal.pone.0061071 (PMC3621887; doi:10.1371/journal.pone.0061071)
Supplement: Table S2 — Pathway and gene signature analysis of differentially regulated genes in EPCR+ and EPCR− subpopulations of MDA-MB-231mfp cells. (PDF) [file pone.0061071.s005.pdf]

Table S2: Microarray data analysis.

A Pathway analysis: The clustered differentially expressed genes between $EPCR^+$ versus $EPCR^-$ samples (see Table S1) were used as input to Ingenuity IPA. The tables below show the top 5 scoring Canonical Pathways identified for each of the 10 branches with a cutoff of p $\leq$ 0.05 (1.3).

Branch 1 (upregulated genes in $EPCR^+$ cells): Top 5 Canonical Pathways

Ingenuity Canonical Pathways	-log(p-value)	Ratio	Molecules
Fcγ Receptor-mediated Phagocytosis in Macrophages and Monocytes	2.1	0.0588	MYO5A, TLN2, ARPC1B, FCGR2A, VAV3, PRKD1
Cardiac β-adrenergic Signaling	1.92	0.0455	PRKACB, GNG11, PPP1R7, GNG2, CACNA1A, MPPE1, PKIG
HER-2 Signaling in Breast Cancer	1.86	0.0625	CCNE1, ERBB3, PIK3R4, ITGB5, PRKD1
Intrinsic Prothrombin Activation Pathway	1.82	0.0938	F12, PROS1, COL3A1
Huntington's Disease Signaling	1.68	0.0381	GNG11, POLR2C, HSPA1L, CDK5, GNG2, PIK3R4, GOSR2, SNCA, PRKD1

Branch 2 (upregulated genes in $EPCR^+$ cells): Top 5 Canonical Pathways

Ingenuity Canonical Pathways	-log(p-value)	Ratio	Molecules
Cdc42 Signaling	4.94	0.0506	MYLK, MYL9, HLA-DMA, WIPF1, HLA-DMB, HLA-DPB1, HLA-DRB5, HLA-DPA1, ITGA4
Antigen Presentation Pathway	4.79	0.125	HLA-DMA, HLA-DMB, HLA-DPB1, HLA-DRB5, HLA-DPA1
Cytotoxic T Lymphocyte-mediated Apoptosis of Target Cells	3.18	0.0588	HLA-DMA, HLA-DMB, HLA-DPB1, HLA-DRB5, HLA-DPA1
Allograft Rejection Signaling	3.13	0.0526	HLA-DMA, HLA-DMB, HLA-DPB1, HLA-DRB5, HLA-DPA1
OX40 Signaling Pathway	3.06	0.0532	HLA-DMA, HLA-DMB, HLA-DPB1, HLA-DRB5, HLA-DPA1

Branch 3 (upregulated genes in $EPCR^+$ cells): Top 5 Canonical Pathways

Ingenuity Canonical Pathways	-log(p-value)	Ratio	Molecules
Antigen Presentation Pathway	3.74	0.075	HLA-DRA, CD74, HLA-F
Complement System	2.36	0.0571	C1R, C3
B Cell Development	2.31	0.0556	IL7R, HLA-DRA
Atherosclerosis Signaling	2.3	0.0233	COL1A2, VCAM1, MMP3
Graft-versus-Host Disease Signaling	2.08	0.04	HLA-DRA, HLA-F

Branch 4 (upregulated genes in $EPCR^+$ cells): Top 5 Canonical Pathways

Ingenuity Canonical Pathways	-log(p-value)	Ratio	Molecules
B Cell Development	3.73	0.0833	HLA-DQA1, HLA-DRB1, HLA-DQB1
Graft-versus-Host Disease Signaling	3.38	0.06	HLA-DQA1, HLA-DRB1, HLA-DQB1
Autoimmune Thyroid Disease Signaling	3.35	0.0492	HLA-DQA1, HLA-DRB1, HLA-DQB1
Nur77 Signaling in T Lymphocytes	3.15	0.0476	HLA-DQA1, HLA-DRB1, HLA-DQB1
Calcium-induced T Lymphocyte Apoptosis	3	0.0429	HLA-DQA1, HLA-DRB1, HLA-DQB1

Branch 5 (upregulated genes in EPCR^{+} cells): Top 5 Canonical Pathways

Ingenuity Canonical Pathways	-log(p-value)	Ratio	Molecules
Huntington's Disease Signaling	3.68	0.072	CAPN5, PIK3C2B, PIK3CA, POLR2D, GLS, DNM3, CREB3L4, GPAA1, GNG7, EP300, TAF9B, CTSD, GNB3, ARFIP2, PRKCD, PLCB1, STX16
Role of NFAT in Cardiac Hypertrophy	2.55	0.0619	MAP2K6, PIK3C2B, PIK3CA, ITPR2, MAPK12, MAPK11, GNG7, EP300, RCAN1, GNB3, PRKCD, PLCB1, ADCY7
Role of Tissue Factor in Cancer	2.34	0.0789	PIK3C2B, PIK3CA, ARRB2, ITGAV, PLCB1, MAPK12, CSF2, MAPK11, PDXP
Propanoate Metabolism	2.25	0.0492	ALDH1B1, ACADVL, PCCA, SUCLG2, ALDH1A3, ACSS1
CCR3 Signaling in Eosinophils	2.21	0.0714	PIK3C2B, PIK3CA, GNB3, ITPR2, PRKCD, PLCB1, MAPK12, MAPK11, GNG7

Branch 6 (upregulated genes in EPCR^{+} cells): No results.

Branch 7 (upregulated genes in EPCR^{-} cells): Top 5 Canonical Pathways

Ingenuity Canonical Pathways	-log(p-value)	Ratio	Molecules
Hypoxia Signaling in the Cardiovascular System	2.39	0.0462	UBE2B, CREB1, UBE2D1
Breast Cancer Regulation by Stathmin1	1.8	0.0192	TUBA1A, CAMK1, CDKN1B, PPP2R5E
ATM Signaling	1.48	0.0339	SMC3, CREB1
Nucleotide Sugars Metabolism	1.41	0.0154	GLCE
Pathogenesis of Multiple Sclerosis	1.35	0.111	CXCL11

Branch 8 (upregulated genes in EPCR^{-} cells): Top 5 Canonical Pathways

Ingenuity Canonical Pathways	-log(p-value)	Ratio	Molecules
Molecular Mechanisms of Cancer	6.09	0.0899	MAP2K4, RAP2B, RAP2A, BAD, BMP2, NFKBIE, TAB2, HRAS, HIF1A, FZD1, E2F3, CCND1, TGFBR2, PTK2 (includes EG:14083), CASP6, GNA15, RHOD, E2F5, FZD2, LRP5, CDKN2D, PRKAR2A, ARHGEF17, RHOJ, BAK1, BCL2L1, FZD8, FOS, MAPK14, CCND2, IRS1, CDKN1A, PRKAG2, ARHGEF18
O-Glycan Biosynthesis	5.65	0.2	GALNT5, GALNTL4, GALNT14, ST6GALNAC5, GALNT1, C1GALT1, GALNT6, GALNT10, FUT4
IGF-1 Signaling	4.47	0.132	RPS6KB1, BAD, SOCS2, SOCS6, PRKAR2A, HRAS, IGFBP7, GRB10, PTK2 (includes EG:14083), FOS, IRS1, PRKAG2, IRS2, CYR61
ILK Signaling	3.8	0.099	MAP2K4, MYH10, FLNB, PPP2CA, BMP2, PPP2R5D, PPP2R3B, RHOJ, HIF1A, CCND1, PGF, PTEN, PTK2 (includes EG:14083), FOS, RHOD, IRS1, SNAI2, PPP1R12A, IRS2
CDK5 Signaling	3.77	0.128	PPP1CC, LAMA5, PPP2CA, PPP2R5D, EGR1, ITGA2, PRKAR2A, PRKAG2, PPP1R12A, HRAS, PPP2R3B, NGF

Branch 9 (upregulated genes in EPCR^{-} cells): **Top Canonical Pathways**

Ingenuity Canonical Pathways	-log(p-value)	Ratio	Molecules
FXR/RXR Activation	2.2	0.0297	IL18 (includes EG:16173), FBP1, SLCO1B3

Ingenuity Canonical Pathways	-log(p-value)	Ratio	Molecules
Hypoxia Signaling in the Cardiovascular System	1.5	0.0308	EDN1, UBE2E2
PXR/RXR Activation	1.45	0.0227	IGFBP1 (includes EG:16006), SLCO1B3
Leukocyte Extravasation Signaling	1.3	0.0151	CXCR4, CYBA, NCF2

Branch 10 (upregulated genes in EPCR$^-$ cells): **Top 5 Canonical Pathways**

Ingenuity Canonical Pathways	-log(p-value)	Ratio	Molecules
Role of Tissue Factor in Cancer	3.76	0.0702	FYN, CTGF, CXCL1, HBEGF, MAPK13, F3, EGFR, FGF5
Hepatic Fibrosis / Hepatic Stellate Cell Activation	3.03	0.0544	IGFBP4, IL1A, CTGF, FGFR1, IGFBP3, IL6, PDGFC, EGFR
Coagulation System	2.82	0.105	THBD, SERPINE1, F3, PLAT
Nitrogen Metabolism	2.77	0.0333	MARC2, LOXL1, CA12, CTH
HMGB1 Signaling	2.63	0.06	IL1A, MAPK13, SERPINE1, RHOF, FNBP1, PLAT

B Ingenuity analysis of gene signatures defining subpopulations of skin cancer stem cells regulated by TGF-β and integrin signaling: Gene lists from Schober M, *et al*. (2011) *PNAS* 108:10544-10549 with more than average 1.5-fold changes between CD34$^+$ vs. CD34$^-$ skin carcinoma populations deficient in TGF-β receptor II (TGFBR2) or focal adhesion kinase (FAK) were included in the analysis. Gene lists with up- and down-regulation were considered separately (cutoff of $p \leq 0.05$ (1.3)).

Branch 1	-log(p-value)	Ratio	Molecules
TGFBR2-KO upregulated	2.05	0.0413	TRIP13, ID2, TGFBI, SLC14A1, FAM129A, ADA, BLM, CENPH, ESCO2

Branch 2	-log(p-value)	Ratio	Molecules
TGFBR2-KO downregulated	3.03	0.0346	COL16A1, FAM176B, WIPF1, FN1, ZBTB20, BAMBI, CSGALNACT1, GBP2 (includes EG:14469), SMARCD3

Branch 3	-log(p-value)	Ratio	Molecules
TGFBR2-KO downregulated	4.24	0.0231	VCAM1, SPP1 (includes EG:20750), C3, KCNJ15, NUPR1, ARHGEF25
FAK-KO upregulated	3.1	0.027	COL5A2, VCAM1, SPP1 (includes EG:20750), C3

Branch 4	-log(p-value)	Ratio	Molecules
FAK-KO upregulated	5.29	0.0405	CCL2, HLA-DQA1, MGP, POSTN, HLA-DRB1, GPNMB
TGFBR2-KO upregulated	1.66	0.0138	ACP5, S100A3, HLA-DQA1
TGFBR2-KO downregulated	1.44	0.0115	MGP, HLA-DRB1, GPNMB

Branch 5	-log(p-value)	Ratio	Molecules
TGFBR2-KO upregulated	2.45	0.0642	MAP2K6, CENPM, SRD5A1, SPC25 (includes EG:100144563), TTK, NCAPD2, DONSON, NEK2, DSCC1, SHMT1, RAD51C, EXO1 (includes EG:26909), E2F8, TK1

Branch 8	-log(p-value)	Ratio	Molecules
TGFBR2-KO downregulated	3.04	0.0769	SOX4, FHL1 (includes EG:14199), MMP14, FAM43A, ANGPTL2, SLC35F3, FZD1, NFKBIZ, AXL, PLXND1, RNASE4, SEPP1, TGM2, TGFBR2, LIFR, LGMN, RGS10, SLC9A3R2, HEBP2, KLF2
FAK-KO upregulated	1.6	0.0743	SEPP1, TGM2, CH25H, CCND2, NFKBIE, FAM43A, CHKA, CD14, RHOJ, NFKBIZ, CCDC80

Branch 9	-log(p-value)	Ratio	Molecules
TGFBR2-KO downregulated	5.19	0.0308	EDN1, CXCR4, RGCC, ZEB1, EFEMP1, CXCL2, INHBB, MECOM
FAK-KO upregulated	3.45	0.0338	CXCR4, CYBA, RGCC, EFEMP1, CXCL2

Branch 10	-log(p-value)	Ratio	Molecules
TGFBR2-KO downregulated	5.22	0.0577	FCGRT, FYN, RGS2 (includes EG:19735), LAPTM5, IFI27, CXCL1, SLIT2, ARHGDIB, RRAGD, COL5A1, FSTL1, TTC28, CYP4V2, SERPINE1, PLAC8
FAK-KO upregulated	3.02	0.0541	COL5A1, ITGB2, FSTL1, RGS2 (includes EG:19735), LAPTM5, LOXL1, CXCL1, PLAC8
FAK-KO downregulated	1.76	0.133	FHIT, GNAI1

C Test for enrichment of stem cell and EMT signatures: The individual branches were analyzed through the use of IPA (Ingenuity® Systems, www.ingenuity.com) for enrichment of the 40- and 380-gene embryonic stem cell-like signatures, and 654-gene PRC2 signature described by Ben-Porath *et al*. (2008) *Nature Genetics* 40:499-507. Results with a p value of 0.05 (1.3) are shown. We found a significant representation of targets for the Polycomb Repressive Complex 2 (PRC2) that are silenced in embryonic stem cells in Branch 3 of genes upregulated in $EPCR^+$ cells, whereas the transcripts of the signature of human embryonic stem cells (hESC 380) were represented in cluster 7 and 8 of genes upregulated in $EPCR^-$ cells. The recently identified mammary stem cell marker and developmental driver, Sox9 (Guo W *et al.* (2012) *Cell* 148: 1015-1028) was differentially expressed at the mRNA, but not the protein level and appeared to be rather regulated by post-translational mechanisms (see Supplementary Fig. 2D). A 64 gene EMT signature (Anastassiou D, et al. (2011) "Human cancer cells express Slug-based epithelial-

mesenchymal transition gene expression signature obtained in vivo." *BMC Cancer* 11:529) was also represented in both EPCR$^+$ and EPCR$^-$ subpopulations (cut-off, $p \leq 0.05$).

**Branch 3**	**-log(p-value)**	**Ratio**	**Molecules**
Anastassiou: EMT signature	1.85	0.0317	COL1A2, COL5A2
Ben-Porath: PRC2 654	1.61	0.00776	OLIG2, HOXB6, DNAJC22, IRX5, HOXC4

**Branch 4**	**-log(p-value)**	**Ratio**	**Molecules**
Anastassiou: EMT signature	3.02	0.0476	POSTN, SPARC, FAP (includes EG:14089)

**Branch 7**	**-log(p-value)**	**Ratio**	**Molecules**
Ben-Porath: hESC 380	2.74	0.0185	HELLS, BMPR1A, GINS4, MAT2A, WDHD1, FRAT2, DSG2

**Branch 8**	**-log(p-value)**	**Ratio**	**Molecules**
Ben-Porath: hESC 380	1.41	0.058	PLA2G16, DEK, DLGAP5, PODXL, SORL1, CHST7, POLE2, HMGA1, SLC38A1, KLHL7, SPRY4, MRS2 (includes EG:380836), USP44, GMNN, JARID2, DNAJB6, TMPO, HOMER1, NFYB, UNG, BUB3 (includes EG:12237), HMGB3

**Branch 10**	**-log(p-value)**	**Ratio**	**Molecules**
Anastassiou: EMT signature	1.94	0.0635	COL5A1, LOX (includes EG:16948), COL6A3, CDH11
