# Supplementary material for: Endothelial Protein C Receptor Function in Murine and Human Breast Cancer Development
Source: PLoS One. 2013 Apr 9;8(4):e61071. doi: 10.1371/journal.pone.0061071 (PMC3621887; doi:10.1371/journal.pone.0061071)
Supplement: Table S3 — Primers used for RT-PCR analysis of gene expression by cell fractions of murine PyMT tumors. (PDF) [file pone.0061071.s006.pdf]

Table S3: Primers used for RT-PCR analysis of PyMT murine tumor samples

	Forward	Reverse
β actin	5’ACGGCCAGGTCAATCACTATTG	5’CCAAGAAGGAAGGCTGGAAAA
CD11c	5’CCGTCTGAGTACCCGGGCCAT	5’TCCTCCACACAGGCCGGGAG
EPCR	5’CTGGTGTGGCCGTGGGCATC	5’TGGGGGAGTCTGTTTGGCGTCA
F7	5’CCGTCTCCCCGTAGCTGCCT	5’TGCGGCACAATTCACGTGTCCT
F10	5’TTCCGGATGAACGTGGCCCCT	5’ATGCGTGCGTCCAAAACCGCT
PAR1	5’TGCTGTCTTCCCGCGTCCCT	5’GAGCGGGGGTTCACCGTAGC
PAR2	5’TGCAGCCGGACCGAGAACCT	5’AGCCTGGTTCTACCGGAACCCC
Protein C	5’CCGCATCCCTTTGGTTGCTCGA	5’TGCCTGCACACAGCATGTTCTCC
TF	5’TCAAGCACGGGAAAGAAAAC	5’CTGCTTCCTGGGCTATTTTG
ST14	5’CTGACCGGCGACATCCTGGC	5’TGTTGGGCGGGTAGTGGCCT
